# Reversible, Chemically Triggered Water Retention in Disulfide Cross-Linked Synthetic Conserved Anterior Mucus Proteins (SCAMPs)

**DOI:** 10.1021/acs.macromol.6c00640

**Published:** 2026-07-31

**Authors:** Manuel A. Lema, Chengyu Sun, Milan A. Shlain, Farhana M. Khan, Keidy L. Matos, Seungri Kim, Vick Tan, Monil S. Patel, Xi Chen, Adam B. Braunschweig

**Affiliations:** † Advanced Science Research Center at the Graduate Center, 14772 The City University of New York, 85 St. Nicholas Terrace, New York, New York 10031, United States; ‡ Department of Chemistry and Biochemistry, Hunter College, 695 Park Ave, New York, New York 10065, United States; § The PhD program in Chemistry, Graduate Center of the City University of New York, 365 fifth Ave, New York, New York 10016, United States; ∥ Nomi Materials Corp, 500 White Plains Rd., Tarrytown, New York 10591, United States; ⊥ Department of Chemical Engineering, The City College of New York, 275 Convent Avenue, New York, New York 10031, United States; # The PhD Program in Physics, The Graduate Center of the City University of New York, 365 fifth Avenue, New York, New York 10016, United States; ∇ The PhD program in Biochemistry, Graduate Center of the City University of New York, 365 fifth Ave, New York, New York 10016, United States

## Abstract

Water-responsive (WR) materials change their morphology and properties in response to variations in relative humidity (RH). Myriad biological processes, including lubrication, adhesion, and ion exchange, rely upon mucus hydrogels, the most prevalent, natural, WR material, and there is substantial interest in mucus hydrogels for biomedical and materials applications. The WR properties of mucus hydrogels arise from the structure of mucins, proteins with *O*-linked glycans on serine/threonine residues and cysteine (Cys)-mediated cross-linking. However, natural mucuses present significant challenges for materials and biomedical use because of their heterogeneity, instability, and limited scalability. As such, there is a need for synthetic materials that can recreate the structures and WR properties of their natural counterparts. We report a series of polymers, poly­(β-Gal-Thr)*_m_*-*r*-poly­(Cys-H)*_n_*, that are inspired by conserved anterior mucus proteins (CAMPs) found in snail mucus and that possess properties —including water adsorption, stiffness, and effective work of adhesion—that are similar to natural mucus hydrogels and that are mediated by reversible, biomimetic disulfide cross-linking. The polymerization conditions—involving the ring opening of *N*-carboxyanhydride monomers to form glycosylated polypeptides—enables independent control over polymer length (*m* + *n*) and monomer ratio (*m:n*). The polymers were characterized by ^1^H NMR spectroscopy, mass spectrometry, and gel permeation chromatography, and all of the data are consistent with the proposed structures. The tunable mechanical and WR properties were measured using atomic force microscopy (AFM) and dynamic vapor sorption (DVS). Notably, as RH increases, the poly­(β-Gal-Thr)*_m_*-*r*-poly­(Cys-H)*_n_* polymers absorb water, with poly­(β-Gal-Thr)_78_-*r*-poly­(Cys-H)_4_ doubling in mass with hysteresis between the absorption and desorption profiles, indicative of a porous structure. Cross-linking to form poly­(β-Gal-Thr)*_m_*-*r*-poly­(Cys-D)*_n_* substantially decreases the water adsorption of the polymers, but the water adsorption can be reestablished by reducing the disulfide cross-links. These SCAMPs represent a significant step toward mimicking the complex structures and WR properties of natural mucus using synthetic analogs, and this work has implications for the design and implementation of synthetic mucus in biomaterials, biotechnology, and medicine.

## Graphical abstract

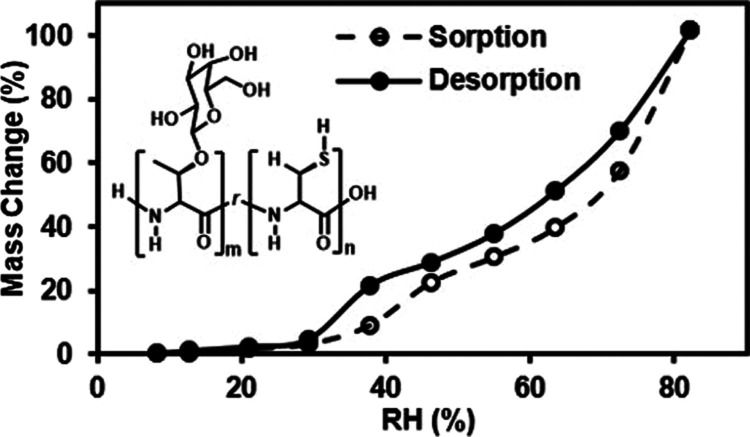

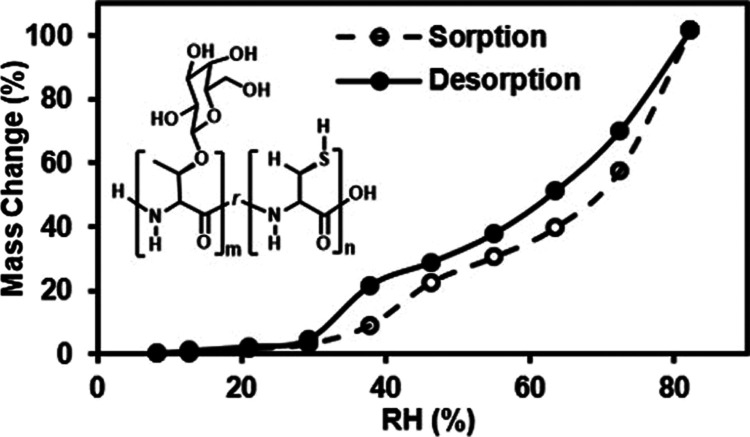

## Introduction

Mucus hydrogels^1^ are water-responsive (WR) materials found throughout the animal kingdom that are used as lubricants, adhesives, and physical barriers.^2,3^ The advantageous WR, adhesive, and tribological properties of mucus hydrogels are attributed to mucins, high-molecular weight proteins where glycans are *O*-linked to polypeptide chains via serine (Ser) and threonine (Thr) residues.^1^ The interplay of mucin chains with other mucin chains and mucin chains with H_2_O influences the viscosity and elasticity of the mucuses, which has consequences for adhesive strength, lubricity, and permissibility to chemicals, proteins, and microbes.^4^ Mucins can form polymer networks as a result of cross-links between adjacent chains in the form of covalent disulfide bridges between Cys residues or by forming noncovalent, Ca^2+^-mediated ion bridges between sugars.^5–7^ The highly glycosylated mucin proteins increase the H_2_O solubility of other components within mucus, and mucus can form gels that are ∼95 wt % H_2_O.^8^ There are many examples in nature where the interactions between mucus and H_2_O are directly related to their unique biological roles. Mucus relies on the retention of H_2_O to create a gel-like layer to protect the epithelial surface of the gut from harmful bacteria and maintain a healthy and balanced microbiome, while controlling the ability to absorb nutrients.^9^ This mucus coating in the gut can contract to expel H_2_O and in turn, change the diffusion of nutrients into the epithelia.^10^ The stomach mucus layer also acts as a diffusion barrier for luminal HCl, thereby protecting the surface epithelial cells. *Helicobacter pylori* changes the pH of the mucus, which alters mucus H_2_O content and in turn, morphology, viscosity, and lubricity, so that the bacteria can tunnel through these mucus barriers and cause ulcers.^11,12^ Meanwhile, the mucus layer of the eye works as a barrier that protects the ocular surface from parasites, allergies, and infections or dry eye disease that can reduce H_2_O content in the mucus layer, lead to inflammation, and damage to the ocular surface.^13,14^

Because mucuses fill so many essential functions in nature, there are substantial current efforts to explore animal-sourced mucuses for biomedical applications,^15^ including for scar/wound healing,^16,2,17,18^ eye lubrication,^19^ anti-inflammation,^20^ drug delivery,^21^ coating for implants,^22^ articular lubrication,^23^ ion and gas exchange membranes,^24^ adhesives,^25^ and cosmetics.^26^ Despite this ample current interest in natural mucus for meeting biotechnological needs for WR materials, the intrinsic properties of these animal-sourced biomaterials make them unsuitable for many of the envisioned applications. For example, animal-sourced mucuses are heterogeneous mixtures of proteins, ions, and lipids, they degrade upon purification,^27^ have batch-to-batch variability,^28,29^ can contain parasites,^30^ and the animals that produce many of the mucuses of interest may not be amenable to scalable and ethical farming.^29^

As a result of the compelling material and biological properties of mucus as well as the challenges inherent to working with natural mucuses, there is now substantial interest in developing synthetic mucins—artificial molecules that seek to recreate the structures and/or properties of their natural counterparts.^31–37^ Currently, synthetic mucins are being explored in the context of drug delivery,^38–40^ H_2_O purification,^41^ cell growth media,^42^ dermal patches,^43^ cosmetics,^44,45^ eye droplets,^40,46,47^ wound healing,^48^ lubrication,^49,50^ and deicing.^51^ These synthetic mucins include polymers with non-natural backbones, such as polyacrylamide,^52^ poly-*N*-isopropylacrylamide,^53^ poly­(ethylene glycol),^54^ or polylactic acid,^55^ which can contain cross-linking that is driven by biotin–streptavidin bonding^56^ or hydrophobic interactions.^57,58^ Researchers have also incorporated Cys into synthetic mucins to increase the viscosity of a polymer by subsequently forming disulfide bridges between polymer chains. For example, Cys has been incorporated into poly­(acrylic acid)-Cys,^59^ deacetylated gellan gum-Cys,^60^ poly­(acrylic acid)-cysteamine,^61^ poly­(methacrylic acid)-Cys,^62^ and poly­(acrylic acid)-homoCys,^63^ chitosan-Cys,^64^ and alginate-Cys^65^ to make materials that mimic some aspects of the properties, but not the structures—particularly the highly glycosylated peptide backbone—of mucins. Meanwhile, the ring-opening polymerization reaction of *N*-carboxyanhydrides (NCAs) provides synthetic polymers with polypeptide backbones, narrow polydispersity indices (*Đ*), and has been deployed successfully to polymerize glycosylated NCA monomers to form synthetic mucins.^34–36,66^

However, none of these synthetic mucin polymers combine all three essential structural elements of mucins that are likely responsible for their WR behavior—the polypeptide backbone, Ser- or Thr-linked *O* -glycans, and Cys residues that are capable of forming reversible disulfide cross-links—nor have the WR properties of these biomimetic polymers been investigated despite the knowledge that a key characteristic of any synthetic materials CAMPs that seek to mimic the advantageous properties of natural mucuses should have potent and dynamic interactions with H_2_O. In an effort to create synthetic polymers whose structures contain the three essential elements of the water-responsive proteins in mucus, here we report a series of polymers—poly­(β*-*Gal-Thr)*_m_*-*r*-poly­(Cys-H)*_n_* —that are random copolymers of a galactose-functionalized threonine NCA monomer—β-AcO-Gal-Thr-NCA—and a *t*-Butyl protected Cys-NCA—*t-*But-Cys-NCA. Rather than being inspired by the block copolymer structure of mucins, these polymers recreate the random copolymer structure of conserved anterior mollusk proteins (CAMPs) that are found in snail mucus^67^ and that are likely responsible for the WR properties of these materials. The synthesis of these synthetic CAMPs (SCAMPs) was optimized so that both degree of polymerization (***X̅***_***n***_), which is equal to *m* + *n*, and ratio of monomers (*m:n*) could be controlled independently. The structures of these polymers were studied by ^1^H NMR spectroscopy, mass spectrometry, and gel permeation chromatography (GPC), and subjected to an Ellman’s assay to quantify thiol incorporation, and all data are consistent with the proposed structures. The Young’s modulus (*E*), effective work of adhesion (*W*), and H_2_O sorption isotherms were examined. These studies confirm that ***X̅***_***n***_ and *m:n* were controllable, disulfide cross-links could be reversibly formed and broken, and that each of the polymers had structure-dependent H_2_O adsorption and WR properties. These results demonstrate that bioinspired materials that reproduce the three salient elements of natural mucins—a polypeptide backbone, *O-*glycosylation, and reversible disulfide cross-links—can also possess reversible WR properties similar to those of natural mucuses. These tunable SCAMPs are an important step toward the development of glycopolypeptide materials for addressing opportunities in biomaterials, biotechnology, and medicine.

## Materials and Methods

### Materials

All reagents and starting materials were purchased from Sigma-Aldrich, TCI, or VWR and used without further purification unless otherwise noted. 1,2,3,4,6-Penta-O-acetyl-β-d-galactopyranose was purchased from BioSynth Inc. (United Kingdom, MT01060). β-AcO-Gal-Thr-NCA was prepared following previously reported protocols.^66^ Although the synthesis of *t*-But-Cys-NCA has been reported previously,^68^ a new synthesis was developed herein. Thin-layer chromatography was carried out using aluminum sheets precoated with silica gel 60 (EMD 40–60 mm, 230–400 mesh with 254 nm dye). Silica gel (Silicycle, 60–200 μm, 70–230 mesh, 60Å) was used for column chromatography. All solvents were used without any further purification unless otherwise noted, and all reactions were carried out under Ar atmosphere using standard Schlenk techniques. THF was dried using sodium hydride (NaH) and benzophenone as the indicator and heating at reflux. Molecular sieves (Alfa Aesar, L05454) were activated in a furnace at 350 °C under an Ar atmosphere for 24 h. Deuterated solvents were purchased from Cambridge Isotope Laboratories Inc. and used as received.

## Methods

### Polymerization and Purification

Each polymerization solution was prepared inside a glovebox using the following general procedure, and details of individual polymerization reactions are given in Table S1. β-AcO-Gal-Thr-NCA was dissolved in dry THF to prepare a 0.1 M solution, and then the desired amount of *t-*But-Cys-NCA was added to the reaction flask. The initiator solution (0.1 M in THF) was added to the Schlenk flask. The reaction vessel was sealed, removed from the glovebox, and heated at 50 °C under Ar. The reaction was monitored by removing 10 μL aliquots and diluting in DMF/DMSO [1:1], and 0.3 mL of the aliquot was injected into the gel permeation chromatography (GPC) instrument. Polymerization progress and monomer consumption were monitored by GPC analysis, and reactions were considered complete when no further monomer consumption was observed. Upon completion of the reaction (no change in *M_n_*), the solvent (THF) was removed under reduced pressure. The reaction mixture was subjected first to the deprotection of the *t*-butyl thiol groups, then the acetate groups by dissolving in H_2_O (10 mL) with 0.5% w/w dithiothreitol (DTT) and stirring for 12 h. MeOH (10 mL) was added to the solution, followed by the addition of K_2_CO_3_ (0.02 mol, 4.0 g), and the mixture was stirred for 2 days. The deprotected polymer was purified by membrane dialysis (*M_w_* cutoff: 3.5 kDa), lyophilized to remove H_2_O, and characterized by mass spectroscopy matrix-assisted laser desorption ionization–time-of-flight (MALDI-ToF), ^1^H NMR, and GPC.

### Determination of Thiol-Incorporation by Ellman’s Assay

Ellman’s reagent (5,5′-dithio-bis­(2-nitrobenzoic acid), 5 mg, 0.0126 mmol) was dissolved in 10 mL of the buffer solution pH 8 (1 mM EDTA, 0.1 M sodium phosphate, pH 8) to prepare a solution of 1.26 mM. Five mg/mL (0.5% w/v) of poly­(β-Gal-Thr)*_m_*-*r*-poly­(Cys-H)*_n_* or poly­(β-Gal-Thr)*_m_*-*r*-poly­(Cys-D)*_n_* was dissolved in 1 mL of buffer solution. Prior to analysis, the polymer solutions were sonicated until complete dissolution was achieved. The mixture (polymers and Ellman’s reagents) was sonicated for 15 min and added to a cuvette (10 mm path length), and the absorbance spectrum was then taken of these solutions (JASCO V-660 UV–vis/NIR spectrophotometer).

### Scanning Electron Microscopy and Energy-Dispersive X-ray Analysis

Samples were mounted on conductive aluminum stubs (Product No. 16120, Ted Pella, Inc.) and sputter-coated with 5 nm layer of gold using a Leica EM ACE600 sputterer. Imaging was performed on a FEI Helios Nanolab 660 focused ion beam–scanning electron microscope (FIB-SEM) with HT of 5 kV, current of 6.3, 13, and 25 pA with an Everhart-Thornley detector. Energy-dispersive X-ray spectroscopy mapping was collected with a Silicon Drift Detectors at HT of 10 kV and current of 1.6 nA. Data were collected and analyzed using AZtec v6.0 software.^69^

### Water Sorption Isotherm Measurement

Water sorption isotherms of poly­(β-Gal-Thr)*_m_*-*r*-poly­(Cys-H)*_n_* and poly­(β-Gal-Thr)*_m_*-*r*-poly­(Cys-D)*_n_* were measured using dynamic vapor sorption (DVS) Intrinsic system (Surface Measurement Systems) at 25 °C. Approximate ∼ 1–4 mg of poly­(β-Gal-Thr)*_m_*-*r*-poly­(Cys-H)*_n_* or poly­(β-Gal-Thr)*_m_*-*r*-poly­(Cys-D)*_n_* was loaded onto the ultrasensitive microbalance of the DVS instrument. Each sample underwent 3 consecutive adsorption/desorption cycles between 8% and 82% RH in 10% increments. In every cycle, RH was increased from 5–90% and decreased back to 5% with 10% RH increments.^70^ Prior to each subsequent RH step, equilibration was defined as either a mass change rate (d*m*/d*t*) below 0.05% mg/min sustained for 5 consecutive mins or a maximum hold time of 20 min.^71^ The mass percent vapor sorption (% VS) at each RH was calculated as eq 1:

1 $$\%\mathrm{VS}=\frac{\left(m-m_{8\%\mathrm{RH}}\right)}{m_{8\%\mathrm{RH}}}\times 100\%$$

where *m* is the sample mass at a given RH and *m*_8%RH_ is the reference mass of each polymer at 8% RH.

### Characterization of *E*

*E* of polymer samples was characterized by atomic force microscopy (AFM) nanoindentation using a customized Multimode 8 AFM (Bruker) operated at 25 °C and 50% RH. Prior to AFM measurements, 2 μL polymer solution (20 mg/mL) was drop-cast onto a silicon wafer and dried overnight at room temperature. The local RH was controlled by adjusting the flow rates of humidified and dry air and was allowed to stabilize at the target value for at least 10 min before measurements. The RH was monitored using a HIH-4021 humidity sensor (Honeywell). AFM nanoindentation measurements were subsequently performed using a NCHV probe (Bruker), with a calibrated spring constant of 55.3 N/m. Force–distance curves were acquired at an indentation rate of 1 Hz. For each location, indentation measurements were repeated three times to ensure reproducibility, and at least three different locations were tested for each sample.^72,73^ The obtained force–distance curves were analyzed using a Hertzian contact model to extract the *E* of the polymer (eq 2).

2 $$F^{2/3}={\left(\frac{4}{3}\frac{E}{{\left(1-v\right)}^{2}}\sqrt{R}\right)}^{2/3}d$$

where *F* is the indentation force, *v* is the Poisson’s ratio (estimated to be 0.3), *R* is the probe radius (8 nm), and *d* is the indentation depth.^74,75^

### Characterization of *W*

*W* was estimated from the area enclosed by the approach and retraction curves in the force–distance curves, and then normalized by the corresponding tip–sample contact area (*A*) to account for variations in indentation depth and contact geometry (eq 3).

3 $$W=\frac{\oint F\left(d\right)dd}{A}$$

where ∮*F*(*d*)­d*d* is the enclosed area of the force–distance curve, corresponding to the mechanical energy dissipated during one loading–unloading cycle. *A* was approximated using the Hertzian contact during loading, *A* = π*Rd*_max_, where *R* is the probe radius and *d*_max_ is the maximum indentation depth. Note that *W* shows a dissipative, nonequilibrium work of adhesion rather than a purely thermodynamic surface energy. It captures energy losses arising from viscoelastic dissipation within the polymer–water network, as well as additional contributions from kinetically limited bond rupture and molecular rearrangements.^76–78^

## Discussion

### Polymer Design

The majority of information on mucus comes from the study of mammalian mucuses, where the predominant, WR proteins are mucins, such as MUC2, that are characterized by *O*-glycan-rich central domains that are flanked at the termini by regions rich in Cys, like von Willebrand factors (Figure 1A).^1,79,80^ The mucus of the garden snail Cornu aspersum, which is currently used in cosmetics^15,81^ and explored in the context of biotechnology,^81,82^ does not contain mucin proteins, rather it contains CAMPs, where Cys are interspersed throughout the proteins, including in the highly glycosylated domains (Figure 1B).^67^ Given the interest in and utility of *C. aspersum* mucus,^15,67^ as well as the accessible synthesis of a bioinspired random copolymer, we selected snail proteins with mucin-like domains, such as CAMP1, CAMP3, and Snail4,^67^ as our design inspiration for the WR SCAMPs described herein.

**Figure 1:**
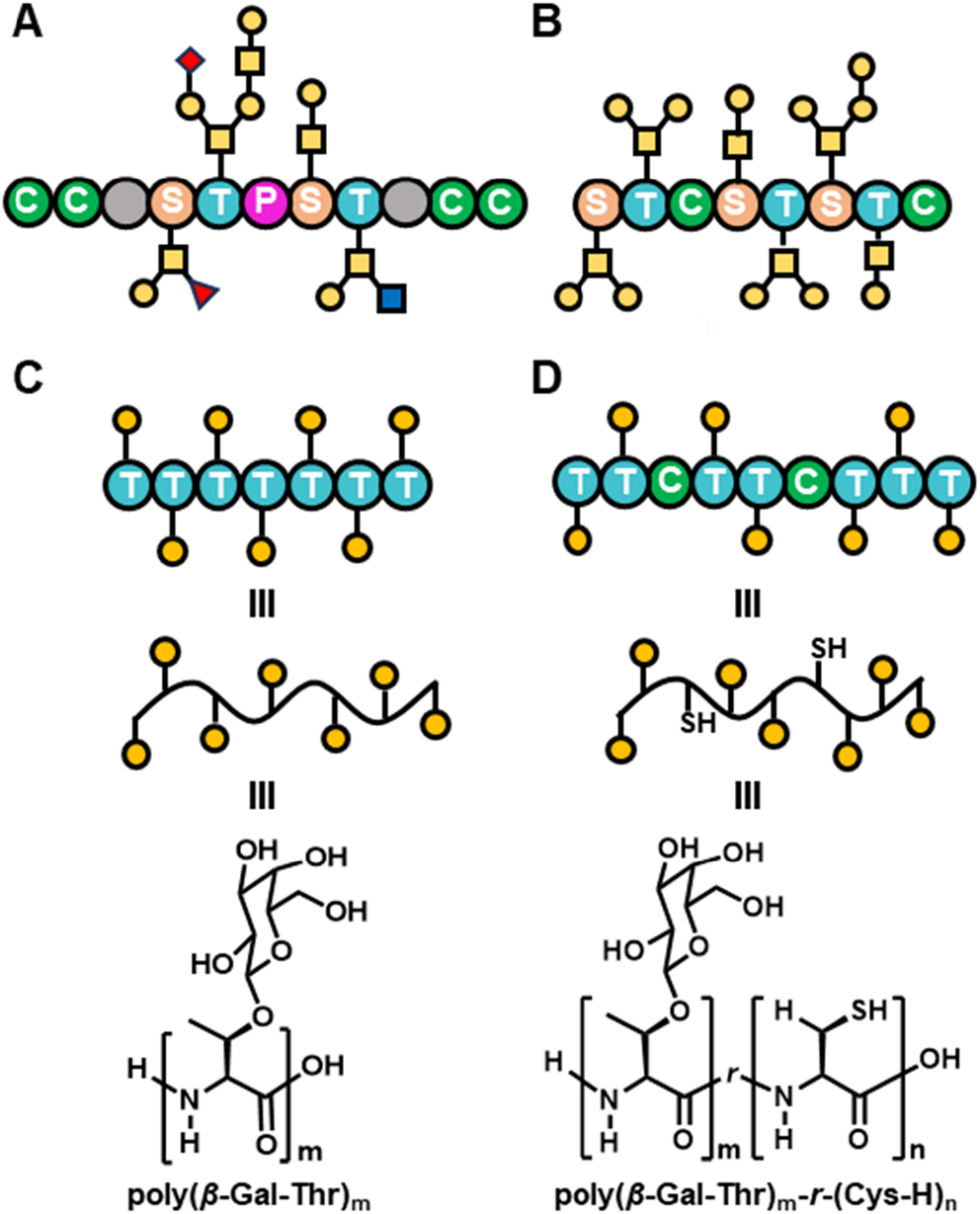
(A) Representation of a mammalian mucin, characterized by Cys-rich terminal domains and a highly *O*-glycosylated central domain. (B) Representation of snail CAMPs, where Cys residues are interspersed within highly *O*-glycosylated domains. (C) Poly­(β-Gal-Thr)*_m_*. Yellow circles represent *O*-linked β-galactose and black line represents the polypeptide backbone. (D) Synthetic CAMP (SCAMP) poly­(β-Gal-Thr)*_m_*-*r*-(Cys-H)*_n_*. −SH indicates cysteine thiols.

Previously, we had reported^66^ the structure of poly­(β-Gal-Thr)*_m_* (Figure 1C), a bioinspired polymer which is intended to mimic aspects of the *O*-glycosylated domains of mucin and CAMP proteins—particularly the Thr-rich peptide backbone and the β-linked Gal residues, which are the most common terminal monosaccharides on mammalian mucin and snail mucin-like protein *O-*glycans. Remarkably, even this simple structure is able to reproduce many of the salient properties of mucins, including interfacial gelation and shear thinning^83^ and formation of lubricating films.^50^ These poly­(β-Gal-Thr)*_m_* polymers, however, lack the critical Cys residues that are involved in interchain cross-linking, which limits their ability to mimic the full range of properties found in natural mucuses. In an effort to form biomimetic SCAMPs whose structures are inspired by the structures of snail CAMPs with H_2_O adsorption and WR behavior approaching those of their natural counterparts, we elaborate the structure of poly­(β-Gal-Thr)*_m_* by incorporating randomly Cys residues that could form disulfide cross-links into the polypeptide backbone, resulting in poly­(β-Gal-Thr)*_m_*-*r*-poly­(Cys-H)*_n_* (Figure 1D). In this nomenclature the “poly­(Cys-H)” indicates polymers where the thiols have not been cross-linked, and “poly­(Cys-D)” indicates polymers that have been cross-linked into disulfide bridges.

## Monomer and Polymer Synthesis

These SCAMPs are formed (Scheme 1) by the ring-opening copolymerization of two NCA monomers—β-AcO-Gal-Thr-NCA, a monomer that creates galactosylated Thr in the polypeptide backbone and whose synthesis we have previously reported^66^—and *t-*But-Cys-NCA, a previously reported^84^ monomer that incorporates as cysteine into the polymer backbone, and which contains a *t*-butyl protecting group on the thiols to prevent interference of unprotected thiols with the anionic propagation mechanism of the polymerization. To copolymerize these monomers, we built upon the conditions we reported for the synthesis of poly­(β-AcO-Gal-Thr)*_m_*,^66^ which involve LiHMDS as an initiator and HFAB as a catalyst, and whose benefits include high yield, *Đ* < 1.2, good control over ***X̅***_***n***_, and the avoidance of toxic heavy metal catalysts.

**Scheme 1:**
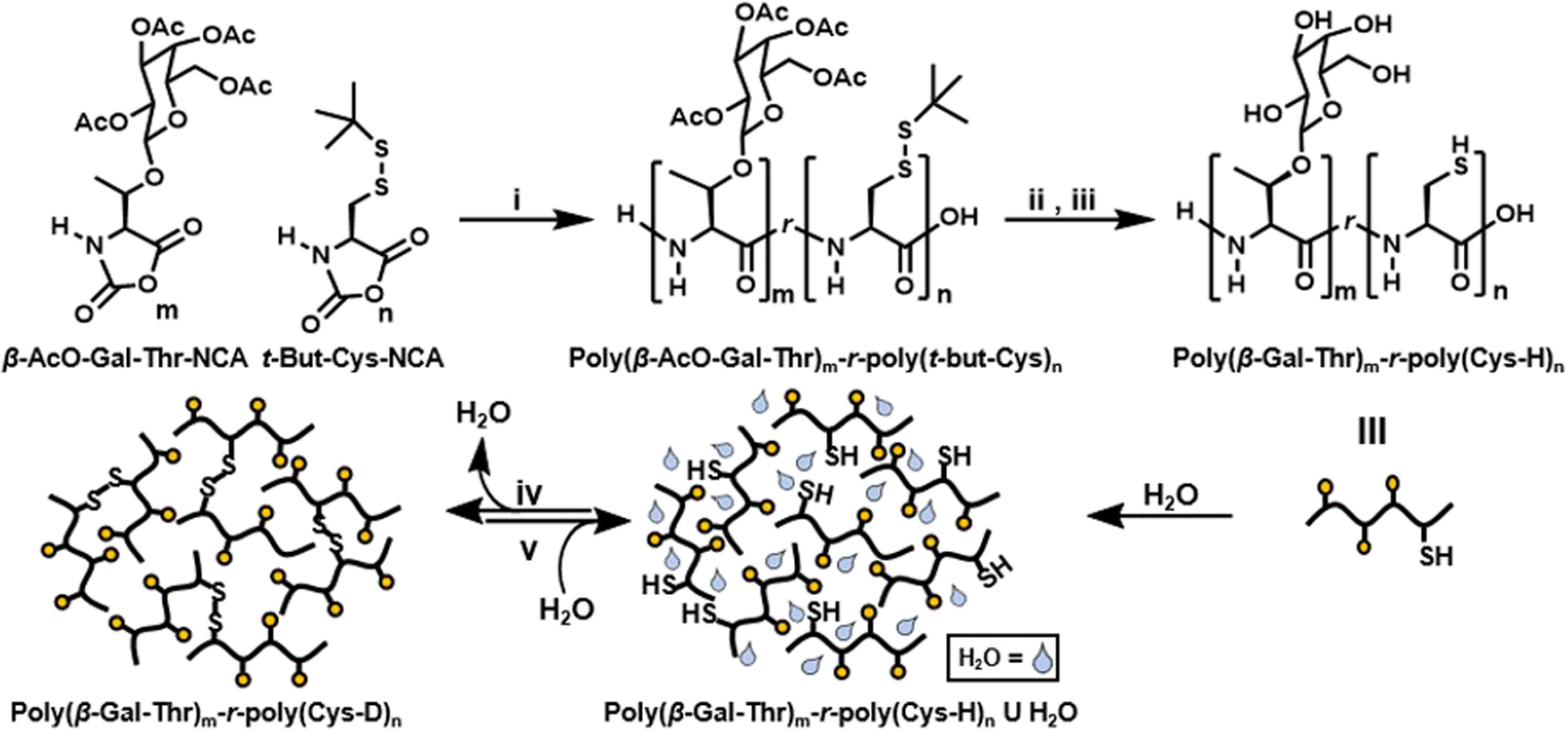
Preparation of SCAMPs poly­(β-Gal-Thr)*_m_*-*r*-poly­(Cys-H)*_n_* *via* the Ring-Opening Random Copolymerization of the *N*-carboxyanhydride (NCA) Monomers β-AcO-Gal-Thr-NCA and *t-*But-Cys-NCA and Reversible Disulfide Cross-Linking and H_2_O uptake^a^

a (i) LiHMDS, THF, 50 °C, 144 h. (ii) DTT, H_2_O, pH 10, 10 h, 71%. (iii) K_2_CO_3_, H_2_O/MeOH, 48 h. (iv) H_2_O_2_, KI, 25 °C, 24 h. (v) DTBA, H_2_O, pH 7, 24 h.

The generic polymerization conditions for forming poly­(β-Gal-Thr)*_m_*-*r*-poly­(Cys-H)*_n_* are as follows: 0.1 M solutions in THF of β-AcO-Gal-Thr-NCA and *t-*But-Cys-NCA were prepared. The solutions were transferred to a Schlenk reaction flask in ratios determined by the target *m:n*. The initiator, in a 0.1 M solution in THF, was added to the reaction in a monomer:initiator ratio ((*m+n)*:i) dependent upon the target ***X̅***_***n***_. The reaction vessel was heated under Ar and stirred vigorously. The reaction was monitored by removing periodically 10 μL aliquots, diluting them in 0.5 mL 1:1 DMF/DMSO, and analyzing them by GPC to determine monomer consumption, *M_n_*, *M_w_*, *Đ*, and ***X̅***_***n***_. When the reaction was observed to have ceased, the THF was evaporated under reduced pressure, then the protecting groups were removed in a one-pot, two-step reaction involving dithiothreitol (DTT) (3% w/v) in H_2_O for 24 h to remove the *t-*butyl protecting groups, followed by the removal of the acetate groups in K_2_CO_3_-saturated MeOH for 24 h, to provide poly­(β-Gal-Thr)*_m_*-*r*-poly­(Cys-H)*_n_*. The reaction mixture was dialyzed (*M_w_* cutoff = 3.5 kDa) to remove the initiator and unreacted monomer, lyophilized, and characterized by ^1^H NMR spectroscopy (Figure 2A), matrix-assisted laser desorption ionization–time-of-flight (MALDI-ToF) mass spectrometry (Figure 2B), GPC (Figure 3), and an Ellman’s assay to quantify the average number of thiols in each polymer chain.

**Figure 2:**
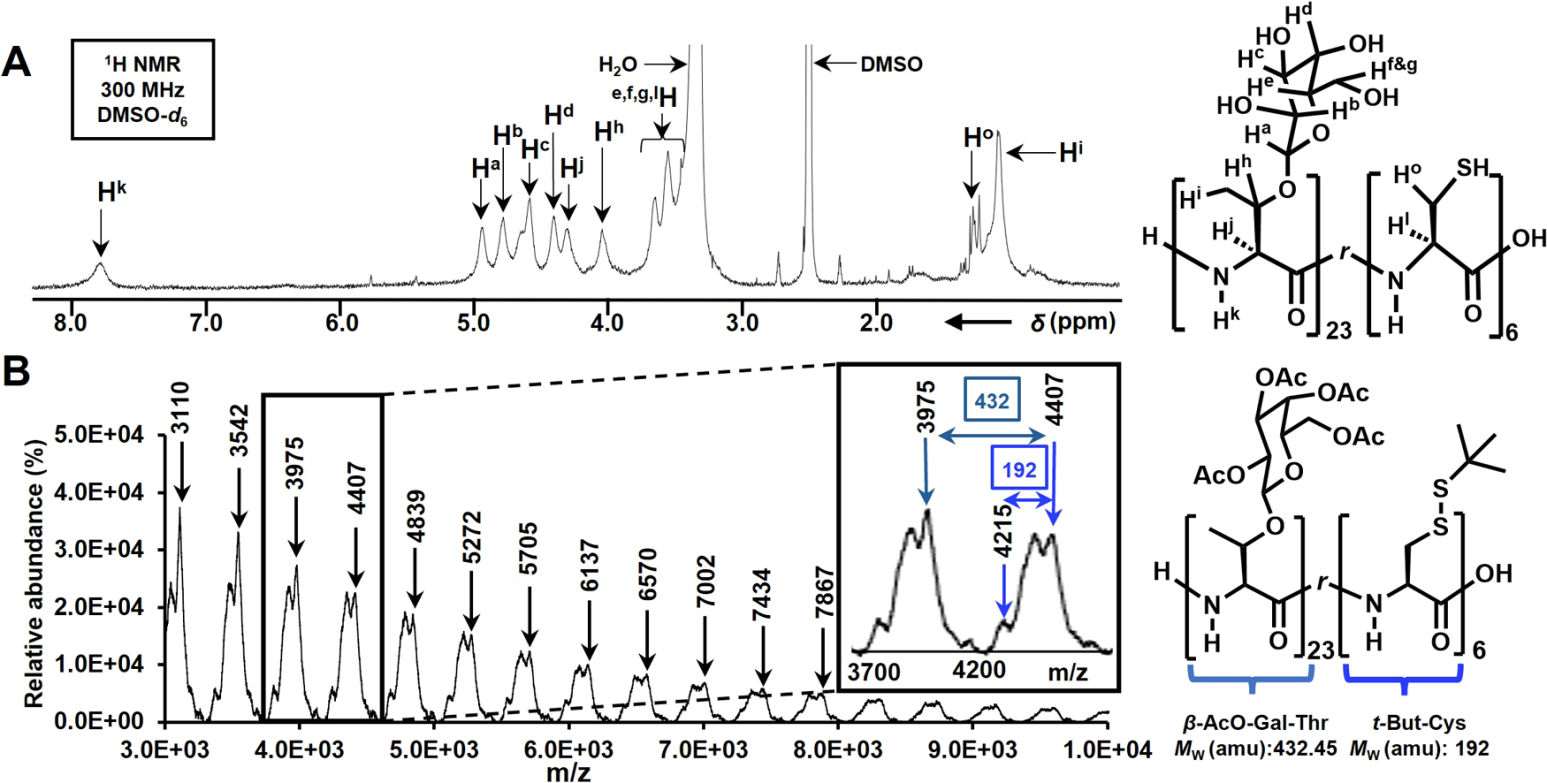
(A) ^1^H NMR of poly­(β-Gal-Thr)_23_-*r*-poly­(Cys-H)_6_ (DMSO–D6, 300 MHz). (B) Matrix-assisted laser desorption ionization time-of-flight mass spectrometry of poly­(β-AcO-Gal-Thr)_23_-*r*-poly­(*t-*But-Cys)_6_.

**Figure 3:**
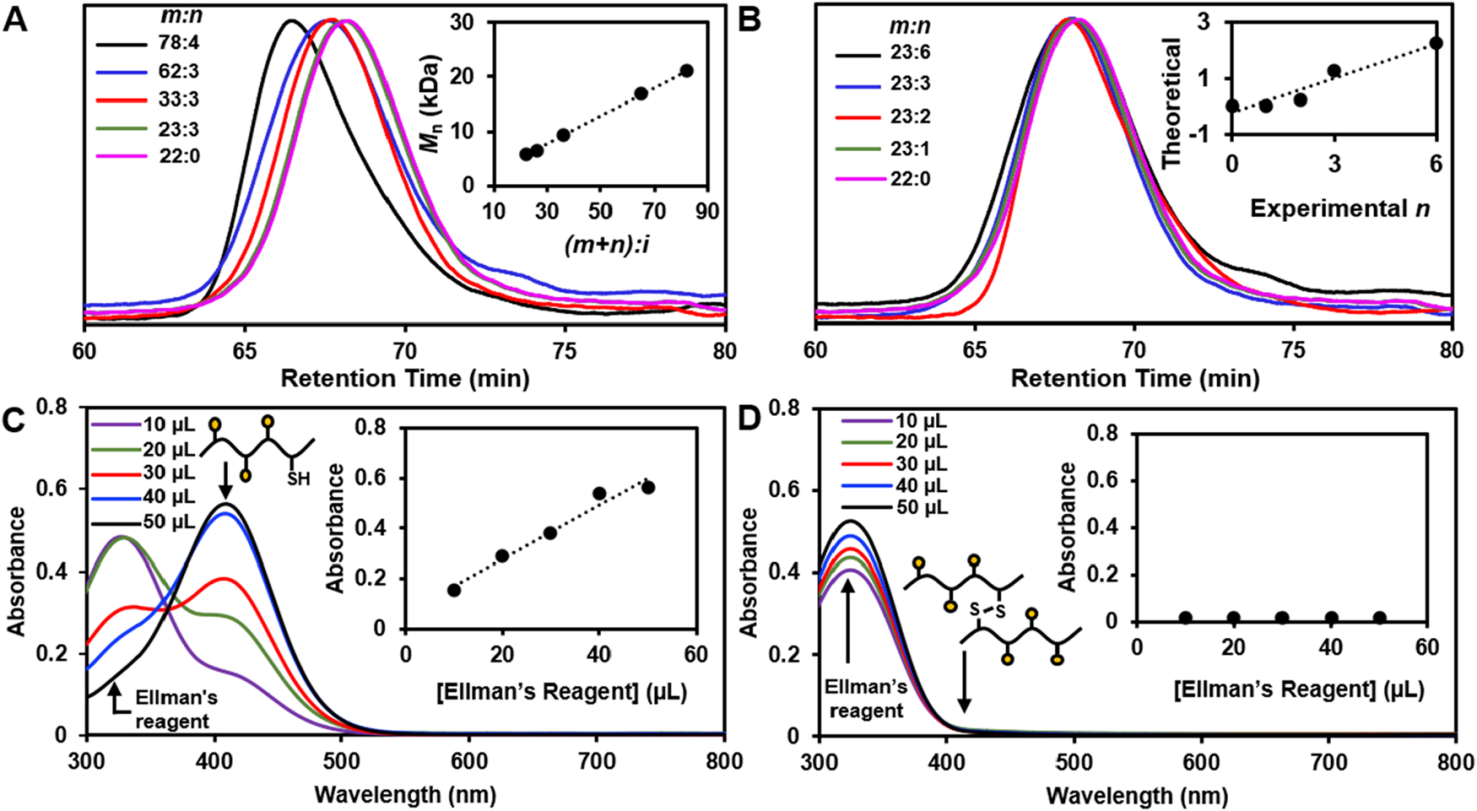
(A) GPC characterization of poly­(β-Gal-Thr)*_m_*-*r*-poly­(Cys-H)*_n_*, where [β-AcO-Gal-Thr-NCA] is varied and [*t-*But-Cys-NCA] and [LiHMDS] remain constant. Inset: *R* ^2^ = 0.94. (B) GPC characterization of poly­(β-Gal-Thr)*_m_*-*r*-poly­(Cys-H)*_n_*, where [β-AcO-Gal-Thr-NCA] and [LiHMDS] are constant and [*t-*But-Cys-NCA] is varied. Inset: *R* ^2^ = 0.95. (C) UV–vis spectra of Ellman’s assay analysis for poly­(β-Gal-Thr)_23_-*r*-poly­(Cys-H)_6_. Peak intensities at 412 nm correlate to [thiol]. (D) UV–vis spectra of Ellman’s assay analysis for poly­(β-Gal-Thr)_23_-*r*-poly­(Cys-D)_6_. Absence of peaks as 412 nm indicates the absence of free thiols in the cross-linked polymers..

^1^H NMR (Figure 2A) spectroscopic analysis of poly­(β-Gal-Thr)_23_-*r*-poly­(Cys-H)_6_ was used to confirm the polymer structure and determine ***X̅***_***n***_ and *m:n*, and this same analysis was applied to all polymers. The peaks at 3.6, 4.1, 4.4, 4.6, 4.8, and 4.9 ppm correspond to galactoside protons H^e,f,g^ and H^j^, H^d^, H^c^, H^b^, and H^a^, respectively. The peaks at 1.1 and 4 ppm (H^i^ and H^j^) correspond to the Thr methylene protons, and the peak at 1.26 ppm (H^o^) corresponds to the Cys methylene protons, confirming the presence of the glycan, the polypeptide backbone, and Cys in the polymer. The absence of peaks corresponding to *t-*butyl (1.3 ppm) and AcO groups (2.0 ppm) confirms the successful removal of both of the protecting groups from the polymer. *M_n_* obtained by end group analysis, involving the determination of the ratios of the relative integration of H^i^ and H^o^, provided the *m:n* of 23:6 and an ***X̅***_***n***_ of 6.71 kDa (Figure S12), and this value of *M_n_* is in agreement with the 6.71 kDa obtained by GPC (Figure S12).

To further confirm the *m:n* ratio that was determined from ^1^H NMR analysis and to confirm that the separate elements of the polymer were linked covalently, MALDI-ToF mass spectrometry was performed (Figure 2B) on poly­(β-AcO-Gal-Thr)*_m_*-*r*-poly­(*t*-But-Cys)*_n_* rather than poly­(β-Gal-Thr)*_m_*-*r*-poly­(-Cys-H)*_n_* because only the former was soluble in CHCl_3_, CH_2_Cl_2_, and MeOH, which are compatible with the MALDI matrix. In the mass spectra, a periodic series of peaks, characteristic of polymer mass spectra, were observed. In the separation of peaks, distances corresponding to the exact masses of the β-AcO-Gal-Thr and *t-*But-Cys repeat units—432 and 192 *m*/*z*, respectively—were observed, which is characteristic of random copolymers.^85^ The compositional analysis supports successful copolymer formation with tunable cysteine incorporation; however, the present data do not directly establish whether a random sequence distribution exists along the polymer backbone. The MALDI-TOF spectra were analyzed according to the method of Nuwaysir et al.^86,87^ These data provide a quantitative value for the ratio of the two monomers, which can be multiplied by the *M_n_* determined from GPC to determine *m:n*. For poly­(β-AcO-Gal-Thr)_23_-*r*-poly­(*t*-But-Cys)_6_, this analysis resulted in an *m:n* of 23:6, which is in agreement with the *m:n* determined by ^1^H NMR analysis.

Conditions for the ring-opening polymerization of β-AcO-Gal-Thr-NCA and *t-*But-Cys-NCA were investigated to explore the effects of catalyst, initiator, temperature (*T*), solvent, and time (*t*) on yields, ***X̅***_***n***_, *M_n_*, *m:n*, and *Đ* (Table 1). Conditions were selected in an effort to avoid halogenated solvents and complex catalysts that contain heavy metals, require their own multistep syntheses, or that are highly O_2_ or H_2_O sensitive. Following previously established, optimized polymerization conditions,^66^ the first conditions explored involved LiHMDS as the initiator in the presence of 1,3-bis­(1,1,1,3,3,3-hexafluoro-2-hydroxypropyl)­benzene (HFAB) as a catalyst. Under these conditions, the polymerization took 8 days to complete with a yield of 78% (Table 1, **Entry 1**).^66^ Subsequently, *t*-butyl acetoacetate (TBAA) in CH_2_Cl_2_ was tested because it can polymerize NCAs in the presence of H_2_O and can form polypeptides with variable *M_n_* and narrow *Đ*, with and without the HFAB as a cocatalyst.^88^ Under these conditions, the reaction time decreased to 6 days with a 56% yield with HFAB, and 3 days with a 45% yield without HFAB (Table 1, **Entry 2** and **3**). However, when the scale of the monomer was increased from 50 mg to 400 mg and *t-*But-Cys-NCA was added, the *t* to reach reaction completion increased to 6 days with a 76% yield (Table 1, **Entry 4**). Despite the acceptable yields and *t*, TBAA remained bound to the polymers following dialysis and required halogenated solvents (Table 1, **Entry 5**), and so reactions conditions involving TBAA were not pursued further. To eliminate halogenated solvents in the polymerizations, dry THF was examined as the reaction solvent with LiHMDS as the catalyst, and this reaction provided the desired polymers (Table 1, **Entry 6**), albeit at low yields (23%). Upon increasing *T* from 25 to 50 °C (Table 1, **Entry 7**), at a 100 mg monomer scale, a significant amount of monomer remained unreacted, which accounts for the low yields. HFAB was added as a catalyst (Table 1, **Entry 8**), which decreased the reaction time to 96 h and increased the yields to 74%. When the reaction was run on a larger scale (2 g) in the absence of HFAB, the monomer was fully consumed, and the polymer was obtained with a yield of 65% (Table 1, **Entry 9**). This difference in yields at small vs large scales can likely be attributed to the outsized effect of trace H_2_O and O_2_ contamination on small-scale reactions. The resulting polymers were obtained over a range of *M_n_*, compositions, and yields, demonstrating the viability of Cys incorporation into SCAMP architectures as well as the limitations of the polymerization chemistry. Following these studies, we selected LiHMDS at 50 °C in THF as standard conditions for all subsequent polymerizations (Table 1, **Entries 9–14**).

**Table 1:** Reaction Conditions Tested for the Preparation of poly­(β-Gal-Thr)*_m_*-*r*-(Cys-H)*_n_* by the Ring-Opening Copolymerization of β-AcO-Gal-Thr-NCA and *t-*But-Cys-NCA

**entry**	^a^i**nitiator**	^b^ **mass (g)**	^a^ **catalyst**	^c^ **solvent**	***T* (°C)**	**time (h)**	**theoretical** ***m.n.i***	**experimental** ***m:n***	**deprotected** ***M**_**n**_* **(kDa)**	^d^ ***M***_***w***_ ***/M**_**n**_* **(*Đ*)**	^e^ *X̅_n_*	^f^y**ield (%)**
1	LiHMDS	0.05	HFAB	CH_2_CI_2_	25	192	25:0:1		6.10	1.22	23	78
2	TBAA	0.05	HFAB	CH2CI2	25	144	25:0:1		5.33	1.61	20	56
3	TBAA	0.05		CH_2_CI_2_	25	72	25:0:1		5.22	1.42	20	45
4	TBAA	040		CH2CI2	25	144	25:0.025:0:1	22:1	5.63	1.35	23	67
5	TBAA	0.05		MeCN	70	192	25:1.25:1					
6	LiHMDS	0.10		THF	25	144	25:0:1		3.69	1.21	14	23
7	LiHMDS	0 10		THF	50	144	25:0:1		22 9	117	87	32
8	LiHMDS	0.10	HFAB	THF	50	96	25:2.5:1	23:6	6.72	1.14	29	74
9	LiHMDS	2.0		THF	50	144	25:0.025:1	23:1	5.35	1.25	24	65
10	LiHMDS	2.0		THF	50	144	25:1:1	23:2	6.18	1.26	25	69
11	LiHMDS	2.0		THF	50	144	25:1.25:1	23:3	6.37	1.21	26	67
12	LiHMDS	2 0		THF	50	144	25:2 5:1	23:6	6 71	1.25	29	71
13	LiHMDS	2.0		THF	50	144	50:2.5:1	33:3	9.02	1.18	36	63
14	LiHMDS	2.0		THF	50	216	75:2.5:1	62:3	16.7	1.23	65	56
15	LiHMDS	2.0		THF	50	288	100:2.5:1	78:4	209	1.21	82	73

a Reaction conditions for the polymers: [initiator] = 0.1 M. [catalyst] = 0.1 M.

b Mass of β-AcO-Gal-Thr-NCA used in the reaction.

c THF was dried over sodium and benzophenone, CH_2_Cl_2_ and MeCN were dried with molecular sieves, freeze–pump–thaw, and purged with Ar.

d *M_w_*, *M_n_*, yields, and *Đ* determined for deprotected polymers, poly­(β-Gal-Thr)*_m_*-*r*-(Cys-H)*_n_*, by GPC (Calibration standard: ReadyCal-Dextran, (82–87) *M*p: 0.18–298 kDa 1:1 DMF: DMSO, 0.25 mL/min, 60 °C).

e *X̅_n_* was determined by dividing the *M_n_* of the polymer by the molecular weight of the repeating unit.

f Reactions were stopped after no further growth was observed when aliquots were periodically removed and analyzed by GPC.

To explore the ability of the optimized polymerization conditions to create polymers with tailored *m:n*, a library of polymers was prepared by systematically varying *m:n:i*. Initially, [β-AcO-Gal-Thr-NCA] in the polymerization was varied, while holding [*t-*But-Cys-NCA] and [LiHMDS] constant, with *m:n:i (β*-AcO-Gal-Thr-NCA: *t*-But-Cys-NCA: LiHMDS) of 22:0:1, 23:3:1, 33:3:1, 62:3:1, and 78:3:1 (Figure 3A). The GPC data show that *M_n_* increases linearly with increasing (*m+n*):*i*, with *M_n_* from 6.37 kDa – 20.9 kDa, demonstrating the ability to control precisely the *M_n_* of the poly­(β-Gal-Thr)*_m_*-*r*-poly­(Cys-H)*_n_*. The resulting *m:n* were 22:0, 23:3, 33:3, 62:3, and 78:4, respectively. The plot of *M_n_* to (*m+n):i* was linear with an *R* ^2^ = 0.94. In the next set of polymers, [*t*-But-Cys-NCA] in the polymerization was varied, while holding [β-AcO-Gal-Thr-NCA] and [LiHMDS] constant, with *m:n:i* of 22:0:1, 23:1:1, 23:2:1, 23:3:1, and 23:6:1 (Figure 3B). The resulting polymers had *m:n* of 22:0, 23:1, 23:2, 23:3, and 23:6, and a plot of theoretical:experimental *m:n* was linear with an *R* ^2^ value of 0.95. The theoretical and experimental *m:n* ratios were unequal because of differences in the relative rates of reactivity of the two monomers, with *t*-But-Cys-NCA reacting approximately 3 times the rate of β-AcO-Gal-Thr-NCA.^66,89^ The slow relative reactivity of β-AcO-Gal-Thr-NCA has been previously attributed to the steric hindrance caused by the galactoside resting over the electrophilic position of the glycosylated monomer.^66^

Conditions for reversibly cross-linking the polymers by forming disulfide bridges were developed, and the success of these reactions were confirmed using Ellman’s assay.^90^ ^,^ ^91^ In the Ellman’s assay of poly­(β-Gal-Thr)*_m_*-*r*-poly­(Cys-H)*_n_*, the peak at 412 nm in the UV–vis spectrum, which increases with increasing polymer concentration, confirms the presence of thiols in the polymers (Figure 3C). From the intensity of the peaks, the number of thiols per chain can be calculated, and the values obtained from the Ellman’s assay agree well with the values obtained by ^1^H NMR, GPC, and MALDI-TOF. To form the disulfide bridges between chains, poly­(β-Gal-Thr)*_m_*-*r*-poly­(Cys-H)*_n_* was oxidized to form poly­(β-Gal-Thr)*_m_*-*r*-poly­(Cys-D)*_n_* by dissolving the polymers in H_2_O_2_ and KI, and stirring at 25 °C for 24 h. The polymers were isolated by dialysis (cutoff = 3.5 kDa), then lyophilized to provide a white powder that was analyzed by the Ellman’s assay. The absence of peaks at 412 nm in the UV–vis spectrum (Figure 3D) suggests that all Cys thiols were converted to disulfide cross-links. To determine the reversibility of the disulfide cross-links, the reducing agent dithiobutylamine^92^ (DTBA) (5 wt %) was added to a sample of poly­(β-Gal-Thr)_23_-*r*-poly­(Cys-D)_6_ that was dissolved in H_2_O and stirring for 24 h. The reduced polymers were obtained as powders following dialysis and lyophilization. The Ellman’s assay confirmed the presence of thiols, with the quantity in good agreement with the original sample of poly­(β-Gal-Thr)_23_-*r*-poly­(Cys-H)_6_ (Figure S29), verifying the ability to quantitatively reverse the disulfide cross-links. While the Ellman’s assay reveals the formation of disulfide bonds, these measurements do not distinguish between intermolecular and intramolecular cross-links, quantify network homogeneity, or determine the topology of the oxidized polymer network.

The H_2_O sorption isotherms of poly­(β-Gal-Thr)*_m_*-*r*-poly­(Cys-H)*_n_* and poly­(β-Gal-Thr)*_m_*-*r*-poly­(Cys-D)*_n_* were measured to investigate the WR properties of these polymers and to determine how Cys, *M_n_*, and cross-linking affect H_2_O uptake, the polymer network, swelling/deswelling processes, kinetic trapping, and changes in intermolecular interactions during hydration and dehydration.^93^ Consequently, the hysteresis observed here is interpreted as evidence of differences in water–polymer interactions and structural rearrangements during sorption rather than definitive proof of pore structure.^93^ For the poly­(β-Gal-Thr)*_m_*-*r*-poly­(Cys-H-D)*_n_*, the effect of *M_n_* on H_2_O adsorption/desorption was monitored for polymers with *m:n* = 22:0, 23:3, 33:3, 62:3, and 78:4 as RH changes between 8 and 82% (Figure 4A), and observed maximum H_2_O uptake at 82% RH is 22.7, 29.1, 35.2, 62.6, and 101.0%, respectively. These observations indicate that H_2_O adsorption capabilities increase with *M_n_*. Interestingly, hysteresis between adsorption and desorption curves appears in all polymers, revealing the porous nature of the SCAMPs.^93^ Note that the poly­(β-Gal-Thr)_78_-*r*-poly­(Cys-H)_4_ with the highest *M*_n_ exhibits the greatest hysteresis between ∼27% RH and 82% RH, indicating high porosity and a broad pore size distribution. To examine the effect of cross-linking on water-uptake behavior, disulfide cross-linking was introduced to generate poly­(β-Gal-Thr)*_m_*-*r*-poly­(Cys-D)*_n_*. Cross-linking led to a pronounced reduction of H_2_O adsorption across all polymers (Figure 4B), with H_2_O uptake at 82% RH of 22.7, 22.5, 21.2, 6.2, and 0.3%, respectively. In comparison, the non-cross-linked poly­(β-Gal-Thr)_78_-*r*-poly­(Cys-H)_4_ exhibits an H_2_O uptake of 101.0% at 82% RH, whereas the cross-linked polymer poly­(β-Gal-Thr)_78_-*r*-poly­(Cys-D)_4_ shows only a 0.3% mass change under the same conditions. These results demonstrate that disulfide cross-linking is associated with a substantial reduction in water uptake and suppression of sorption hysteresis. While these observations are consistent with reduced polymer mobility, restricted swelling, and altered network organization following cross-linking, the DVS and SEM data do not directly establish the specific structural features responsible for the observed water-uptake behavior.^71^

**Figure 4:**
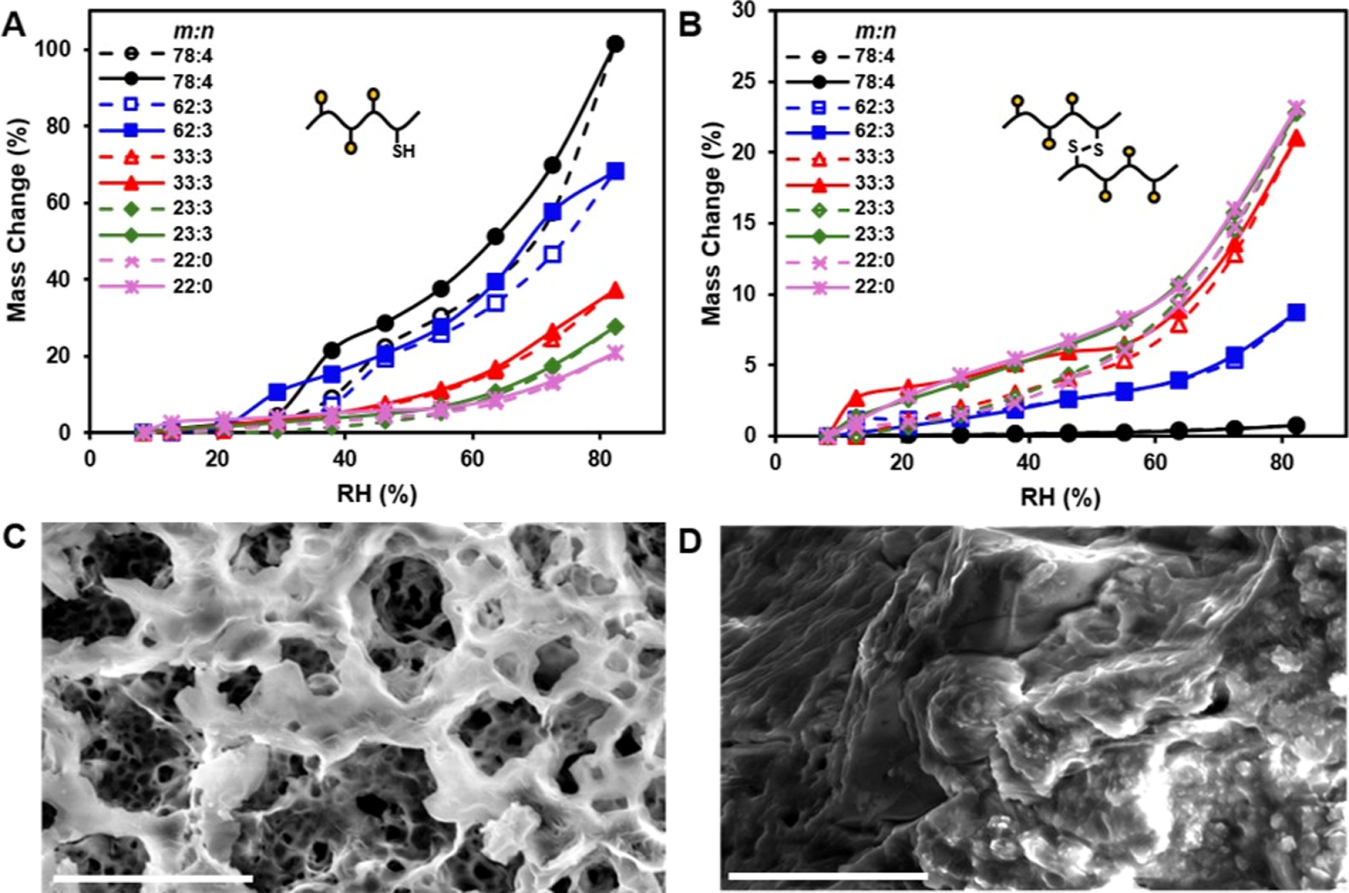
DVS and SEM analysis of SCAMPs. (A-B) Water sorption isotherms of poly­(β-Gal-Thr)*_m_*-*r*-poly­(Cys-H)*_n_* (A) and poly­(β-Gal-Thr)*_m_*-*r*-poly­(Cys-D)*_n_*. The dashed lines represent adsorption and the solid lines represent desorption. (C–D) SEM images of poly­(β-Gal-Thr)_78_-*r*-poly­(Cys-H)_4_ (C) and poly­(β-Gal-Thr)_78_-*r*-poly­(Cys-D)_4_ (D). Scale bar is 20 μm.

To test the reversibility of the disulfide cross-links and the ability to reestablish the H_2_O sorption profile, the cross-linked polymer, poly­(β-Gal-Thr)_23_-*r*-poly­(Cys-D)_6_, was reduced using 0.5 wt % of DTBA for 24 h at 25 °C. Ellman’s reagent analysis was performed to determine the [SH] (Figure S33), which showed that the thiols could be recovered quantitatively. DVS for the poly­(β-Gal-Thr)_23_-*r*-poly­(Cys-H)_6_ after the oxidation–reduction cycle was taken to determine the ability of the regenerated polymer to absorb H_2_O. The H_2_O adsorption capacity (29.9%) was identical to the polymer prior to cross-linking, confirming that these polymers have chemically triggered, reversible changes to their H_2_O sorption profiles. The substantial decrease in water uptake following oxidation likely reflects disulfide formation and accompanying changes in polymer organization and morphology. While reduction restored accessible thiols and in turn, swelling behavior, these measurements do not interrogate whether the original morphology was reestablished within the polymer network.

Scanning electron microscopy (SEM) images of poly­(β-Gal-Thr)_78_-*r*-poly­(Cys-H)_4_ and poly­(β-Gal-Thr)_78_-*r*-poly­(Cys-D)_4_ reveal differences in their microstructural morphology that are consistent with their respective DVS behaviors. Poly­(β-Gal-Thr)_78_-*r*-poly­(Cys-H)_4_ exhibits a highly porous morphology, characterized by interconnected voids (Figure 4C). Such an open microstructure would be expected to absorb and retain significant amounts of H_2_O as the highly glycosylated pores can trap^83^ H_2_O and form H-bonds between H_2_O and the matrix. In contrast, the cross-linked poly­(β-Gal-Thr)_78_-*r*-poly­(Cys-D)_4_ (Figure 4D) has a denser and more compact microstructure. This compressed structure may limit the H_2_O retention and reduce the swelling, which is in agreement with the substantially reduced H_2_O uptake and reversible hydration behavior observed in the DVS measurements.

The *E* of poly­(β-Gal-Thr)*_m_*-*r*-poly­(Cys-H)*_n_* and poly­(β-Gal-Thr)*_m_*-*r*-poly­(Cys-D)*_n_* were measured to evaluate how disulfide cross-linking and ***X̅***_***n***_ affect the stiffness of the polymers. The effect of non-cross-linked and cross-linked thiols on *E* was evaluated by holding *m* constant and varying *n* (*m:n* = 22:0 23:1, 23:2, 23:3 and 23:6) at 50% RH (Figure 5A and Table 2). For the non-cross-linked polymers_,_ the *E* values are 4.7, 0.7, 0.5, 1.8, and 0.4 GPa, respectively. In the case of cross-linked polymers, the *E* values are 4.7, 6.7, 2.2, 2.8, and 6.6 GPa, respectively. It is clear that the formation of disulfide bonds increases *E*, although *E* does not increase monotonically with *n*. Subsequently, the effect of ***X̅***_***n***_ on *E* was evaluated on poly­(β-Gal-Thr)*_m_*-*r*-poly­(Cys-H)*_n_* and poly­(β-Gal-Thr)*_m_*-*r*-poly­(Cys-D)*_n_* with polymers *m:n* = 22:0, 23:3, 33:3, 62:3, and 78:4 (Figure 5B and Table 2), and *E* values for both non-cross-linked (4.7, 1.8, 1.1, 7.1, 7.1, and 0.9 GPa, respectively) and cross-linked polymers (4.7, 2.8, 2.9, 7.5, and 4.8 GPa, respectively) were determined. In both cases, *E* is greater for the cross-linked form, which makes the polymers are stiffer and more rigid, and therefore more resistant to elastic deformations. These data reveal that cross-linked polymers have a higher *E* that complex relationships exist between *m:n*, *m+n*, and *E*, and that *E* can be tuned by varying *m:n* and *m+n*.

**Figure 5:**
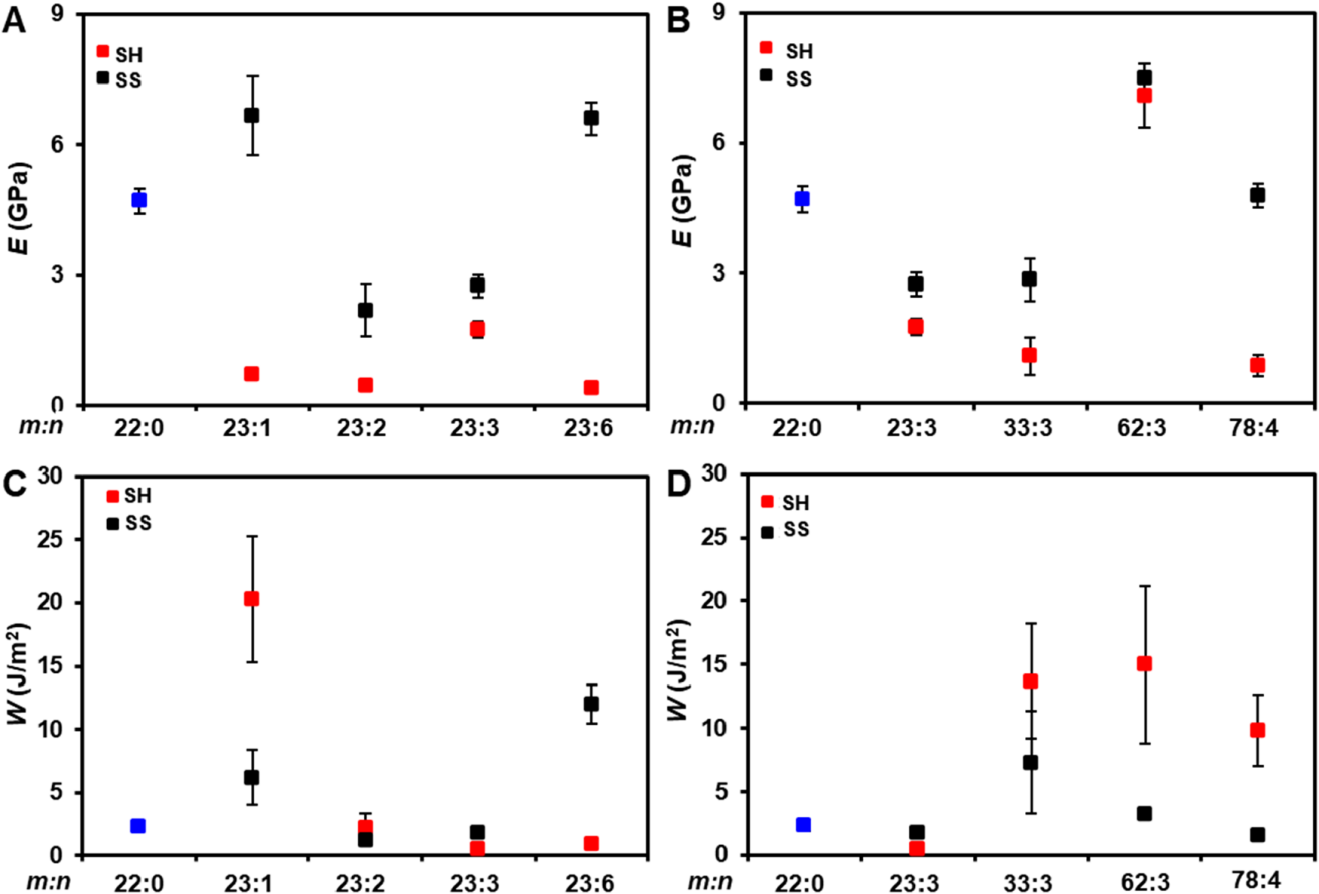
Young’s modulus (*E*) and effective work of adhesion (*W*) of poly­(β-Gal-Thr)*_m_*-*r*-poly­(Cys-H)*_n_* and poly­(β-Gal-Thr)*_m_*-*r*-poly­(Cys-D)*_n_* at 50% RH. (A) *E* for poly­(β-Gal-Thr)*_m_*-*r*-poly­(Cys-H)*_n_* (red) and poly­(β-Gal-Thr)*_m_*-*r*-poly­(Cys-D)*_n_* (black) with constant *m*, varying *n* and poly­(β-Gal-Thr)_22_ (blue). (B) *E* for poly­(β-Gal-Thr)*_m_*-*r*-poly­(Cys-H)*_n_* (red) and poly­(β-Gal-Thr)*_m_*-*r*-poly­(Cys-D)*_n_* (black) with constant *n*, varying *m* and poly­(β-Gal-Thr)_22_ (blue). (C) *W* for poly­(β-Gal-Thr)*_m_*-*r*-poly­(Cys-H)*_n_* (red) and poly­(β-Gal-Thr)*_m_*-*r*-poly­(Cys-D)*_n_* (black) with constant *m*, varying *n* and poly­(β-Gal-Thr)_22_ (blue). (D) *W* for poly­(β-Gal-Thr)*_m_*-*r*-poly­(Cys-H)*_n_* (red) and poly­(β-Gal-Thr)*_m_*-*r*-poly­(Cys-D)*_n_* (black) with constant *n*, varying *m* and poly­(β-Gal-Thr)_22_ (blue).

**Table 2:** Comparison of Maximum H_2_O (82% RH), *E* (50% RH) and *W* (50% RH) for Poly­(β-Gal-Thr)*_m_*-*r*-(Cys-H)*_n_* (SH) and poly­(β-Gal-Thr)*_m_*-*r*-(Cys-D)*_n_* (SS) at 50% RH

**SH**						**SS**				
***m:n***	**DVS 82% RH**	**hysteresis at 38% RH**	**hysteresis** ^a^ **72% RH**	***E*** ^b^ **(GPa)**	***W*** ^b^ (J/m^**2**^ **)**	**DVS 82% RH**	**hysteresis** ^a^ **at 38% RH**	**hysteresis** ^a^ **at 72% RH**	***E*** ^b^ **(GPa)**	***W*** ^b^ (J/m^**2**^ **)**
22:0	**22.7**	**2.11**	**0.750**	**4.70**	**2.34**	**22.7**	**2.11**	**0.750**	**4.70**	**2.34**
23:1	**25.4**	**3.43**	**2.01**	**0.730**	**20.3**	**21.7**	**1.58**	**1.21**	**6.67**	**6.18**
23:2	**27.7**	**2.43**	**1.86**	**0.470**	**2.20**	**22.5**	**2.32**	**1.17**	**2.20**	**1.20**
23:3	**29.0**	**2.37**	**0.807**	**1.76**	**0.500**	**20.3**	**1.70**	**0.959**	**2.75**	**1.80**
23:6	**26.1**	**1.75**	**1.88**	**0.400**	**0.330**	**18.7**	**1.56**	**0.694**	**6.60**	**12.0**
33:3	**35.2**	**0.413**	**2.26**	**1.10**	**13.7**	**21.2**	**3.07**	**1.25**	**2.86**	**7.30**
62:3	**62.6**	**8.14**	**11.1**	**7.10**	**15.0**	**6.19**	**0.145**	**0.324**	**7.50**	**3.20**
78:4	**101**	**12.5**	**12.5**	**0.370**	**9.30**	**0.332**	**0.034**	**0.026**	**4.80**	**1.52**
23:6	**28.8** ^c^	**0.972** ^c^	**7.47** ^c^							

a Hysteresis units are (J/m^2^).

b AFM Measurements: *E* of the polymers was measured using (AFM) nanoindentation using a customized Multimode 8 AFM (Bruker) operated at 25 °C and 50% RH. Prior to AFM measurements, 2 μL polymer solution (20 mg/mL) was drop-cast onto a silicon wafer and dried overnight at room temperature. AFM nanoindentation measurements were subsequently performed using NCHV probe (Bruker), with a calibrated spring constant of 55.3 N/m. *W* was determined from the area enclosed by the approach and retraction curves in the force–distance curves, and then normalized by the corresponding tip–sample contact area (A) to account for variations in indentation depth and contact geometry, $W=\frac{\oint F\left(d\right)dd}{A}$ . The *W* shows a dissipative, nonequilibrium work of adhesion rather than purely thermodynamic surface energy.

c Measurements after reduction (reversibility) (*E* and *W* were not retaken).

The effect of cross-linked and non-cross-linked thiol on *W* was evaluated by holding constant *m* and varying *n* (22:0, 23:1, 23:2, 23:3, and 23:6), and these data are shown in Figure 5C and summarized in Table 2. The values of *W* for the non-cross-linked polymers are 2.3, 20.3, 2.2, 0.5, and 0.9 J/m^2^, respectively, whereas the corresponding values for the cross-linked polymers are 2.34, 6.18, 1.20, 1.18, and 12.0 J/m^2^, respectively. As ***X̅***_***n***_ was increased (*m:n* 22:0, 23:3, 33:3, 62:3 and 78:4), the non-cross-linked polymers exhibited values of 2.3, 0.5, 13.7, 15.0, and 9.8 J/m^2^, respectively. After the formation of disulfide bonds, the corresponding values were 2.3, 1.8, 7.3, 3.2, and 1.5 J/m^2^, respectively. In both cases, we found that *W* is higher for the non-cross-linked polymers, and the dependence on thiol content and chain length is nonmonotonic.

Considering these nonmonotonic trends together with the structural data suggests that *W* is governed by multiple coupled factors rather than a single structural parameter. For the non-cross-linked polymers, the highest *W* is observed at *m:n* = 23:1 (20.3 J/m^2^), indicating enhanced energy dissipation during the contact cycle. This behavior may be related to the combined effects of higher H_2_O uptake and a more deformable polymer network, which together influence energy dissipation during loading and unloading. In contrast, *W* is substantially reduced for most cross-linked polymers compared to their non-cross-linked counterparts, which is consistent with a more constrained network and a limited amount of adsorbed H_2_O in the former. Overall, these results suggest that variations in *W* are the result of the counterbalancing effects of polymer composition, cross-linking state, and interfacial hydration.^94–97^

## Conclusions

In conclusion, we have developed synthetic protocols for polymers that contains the three salient features of natural mucin polymers – a polypeptide backbone, terminal β-galactosides, and Cys residues. The H_2_O vapor sorption isotherm, *E*, and *W* of these poly­(β-Gal-Thr)*_m_*-*r*-poly­(Cys-H/Cys-D)*_n_* SCAMPs polymers are strongly dependent upon *m:n*, *m+n*, and cross-linking. Poly­(β-Gal-Thr)*_m_*-*r*-poly­(Cys-H)*_n_* exhibit high porosity, flexibility, and significant H_2_O uptake that increases with *M_n_*, reflecting their ability to form H-bonds and trap H_2_O within an open, interconnected network. In contrast, the disulfide cross-linked polymers, poly­(β-Gal-Thr)*_m_*-*r*-poly­(Cys-D)*_n_*, possess a dense, compact morphology with restricted pore accessibility, resulting in markedly reduced H_2_O sorption. The tunable WR and mechanical properties of this platform provides the flexibility to adapt these SCAMPs toward applications, such as drug delivery and cosmetics, that have different WR demands. While these examples illustrate the potential utility of SCAMPs across a broad range of applications, the present work focuses on establishing fundamental structure–property relationships in cysteine-containing SCAMPs, and does not directly evaluate performance in any specific application.

## Supplementary Material



## Notes

Macromolecules, including materials, synthetic procedures, supplementary tables, MALDI-TOF mass spectra, ^1^H and ^13^C NMR spectra, and GPC spectra. NMR, SEC, GPC, and other analytical data supporting the findings of this study are available from the corresponding author upon reasonable request (PDF)
