# Supplementary material for: Reversible, Chemically Triggered Water Retention in Disulfide Cross-Linked Synthetic Conserved Anterior Mucus Proteins (SCAMPs)
Source: Macromolecules. 2026 Jul 31;59(15):8607–19. doi: 10.1021/acs.macromol.6c00640 (PMC13472211; doi:10.1021/acs.macromol.6c00640)
Supplement: Supplementary file 1 [file ma6c00640_si_002.pdf]

Supporting Information

Reversible, chemically triggered water retention in disulfide cross-linked synthetic conserved anterior mucus proteins (SCAMPs)

Manuel A. Lema[a,b,c,d], Chengyu Sun[a,e], Milan A. Shlain[a,b,c], Farhana M. Khan[a,d], Keidy L. Matos[a,b,c.d], Seungri Kim[a,e], Vick Tan[a], Monil S. Patel[a,b], Xi Chen[a,c,e,f], Adam B. Braunschweig[a,b,c,d,g*]

[a] M. A. Lema, C. Sun, M. A. Shlain, F.M. Khan, K. L. Matos, S. Kim, V. Tan, M. S. Patel, Prof. X. Chen, Prof. A. B. Braunschweig
Advanced Science Research Center at the Graduate Center
The City University of New York
85 St. Nicholas Terrace, New York, NY, 10031, USA

[b] M. A. Lema, M. A. Shlain, K. L. Matos, M. S. Patel. Prof. A. B. Braunschweig
Department of Chemistry and Biochemistry
Hunter College
695 Park Ave, New York, NY, 10065, USA

[c] M. A. Lema, M. A. Shlain, K. L. Matos, Prof. X. Chen, Prof. A. B. Braunschweig,
The PhD program in Chemistry
Graduate Center of the City University of New York
365 5th Ave, New York, NY, 10016, USA

[d] M. A. Lema, Prof. A. B. Braunschweig
Nomi Materials Corp
500 White Plains Rd., Tarrytown, NY 10591

[e] C. Sun, S. Kim, Prof. X. Chen
Department of Chemical Engineering
The City College of New York
275 Convent Avenue, New York, NY 10031, USA

[f] Prof. X. Chen
The PhD Program in Physics
The Graduate Center of the City University of New York
365 5th Avenue, New York, NY 10016, USA

[g] Prof. A. B. Braunschweig
The PhD program in Biochemistry
Graduate Center of the City University of New York

365 5th Ave, New York, NY, 10016, USA

Email: abraunschweig@gc.cuny.edu

Table of Contents

1. Monomer synthesis and characterization ………………..………………………………..S4
2. Polymer synthesis and characterization ……………………………………………………S6
3. Determination of thiol-incorporation by Ellman's assay ………………………….………S27
4. Scanning electron microscopy and energy-dispersive X-ray analysis…..………………S37
5. Dynamic vapor sorption…………………..…………………………………………….....S53
6. Determination of Young's modulus and effective work of adhesion of polymers …......S86
7. References …………….…………..……………………………………………….. S101

1. Monomer synthesis and characterization

General methods. All reagents and starting materials were purchased from Sigma-Aldrich, TCI, or VWR and used without further purification unless otherwise noted. 1,2,3,4,6-penta-O-acetyl-β-D-galactopyranose was purchased from BioSynth Inc. (United Kingdom, MT01060). *β*-AcO-Gal-Thr-NCA was prepared following previously reported protocols.[1] Although the synthesis of *t*-But-Cys-NCA has been reported previously,[2] a new synthesis was developed herein. Thin-layer chromatography was carried out using aluminum sheets precoated with silica gel 60 (EMD 40 - 60 mm, 230 - 400 mesh with 254 nm dye). Silica gel (Silicycle, 60-200 μm, 70-230 mesh, 60Å) was used for column chromatography. All solvents were used without any further purification unless otherwise noted, and all reactions were carried out under Ar atmosphere using standard Schlenk techniques. THF was dried using sodium hydride with benzophenone as the indicator and heating at reflux. Molecular sieves (Alfa Aesar, L05454) were activated in a furnace at 350 °C under an Ar atmosphere for 24 h. Deuterated solvents were purchased from Cambridge Isotope Laboratories Inc. and used as received. ^{1}H NMR spectra were obtained on a Bruker 300 MHz spectrometer. All chemical shifts are reported in *δ* units (ppm) using the solvent residual signal as an internal standard. The following abbreviations are used for signal multiplicities: *s*, singlet; *br s*, broad singlet; *d*, doublet; *t*, triplet; *q*, quartet; *m*, multiplet; *dd*, doublet of doublets; *ddd*, doublet of doublets of doublets. High-resolution electrospray ionization mass spectra were obtained on Agilent Q-TOF system.

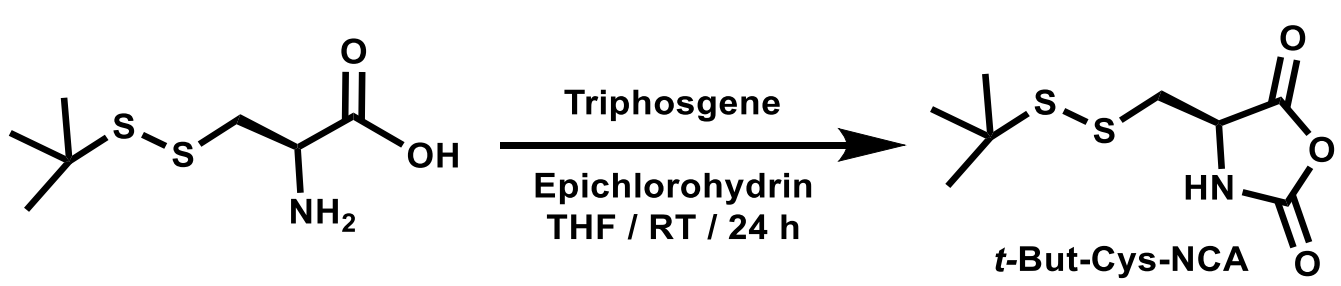

Scheme S1. Synthesis of *t*-But-Cys-NCA.

Synthesis of *S*-tert-butyl-mercapto-L-Cysteine-NCA (*t*-But-Cys-NCA): *S*-*t*-But-mercapto-L-Cysteine (5.00 g, 0.0239 mol, 1.00 eq) and epichlorohydrin (0.0445 mol, 4.12 g, 4.00 eq) were dissolved into THF (125 mL). Triphosgene (3.96 g, 0.0133 mol, 3.00 eq) was added to the stirring solution. The mixture was stirred for 24 h at room temperature under Ar atmosphere. The mixture was then diluted with EtOAc (100 mL) and washed with cold H_2O (2 x 100 mL). The organic phase was washed with cold saturated $NaHCO_3$ (aq) (2 X 100 mL) and cold saturated NaCl (aq) (1 X 100 mL). The organic layer was then dried with Na_2SO_4 (anhydrous), and the solvent was removed under reduced pressure. The residue was dissolved in CH_2Cl_2 (100 mL), stirred for 30 min, and filtered through celite. The solvent was evaporated under reduced pressure, and the residue was dissolved in $CHCl_3$ (5 mL). Et_2O was added to precipitate the product, and the resulting white solid was recrystallized in $CHCl_3$ to provide *t*-But-Cys-NCA as a white solid (2.92 g, 52.0 %). ^{1}H NMR (300 MHz, CD_2Cl_2) δ 6.27 (*s*, 1H), 4.75 (*ddd*, *J* = 8.9, 3.4, 0.9 Hz, 1H), 3.25 (*dd*, *J* = 14.1, 3.4 Hz, 1H), 2.98 (*dd*, *J* = 14.1, 8.9 Hz, 1H), 1.40 (*s*, 9H). ^{13}C NMR (75 MHz, CD_2Cl_2) δ 57.29, 54.15, 53.79, 53.43, 53.07, 52.71, 48.97, 40.85, 29.55. This data matches with the previously reported spectrum.[2]

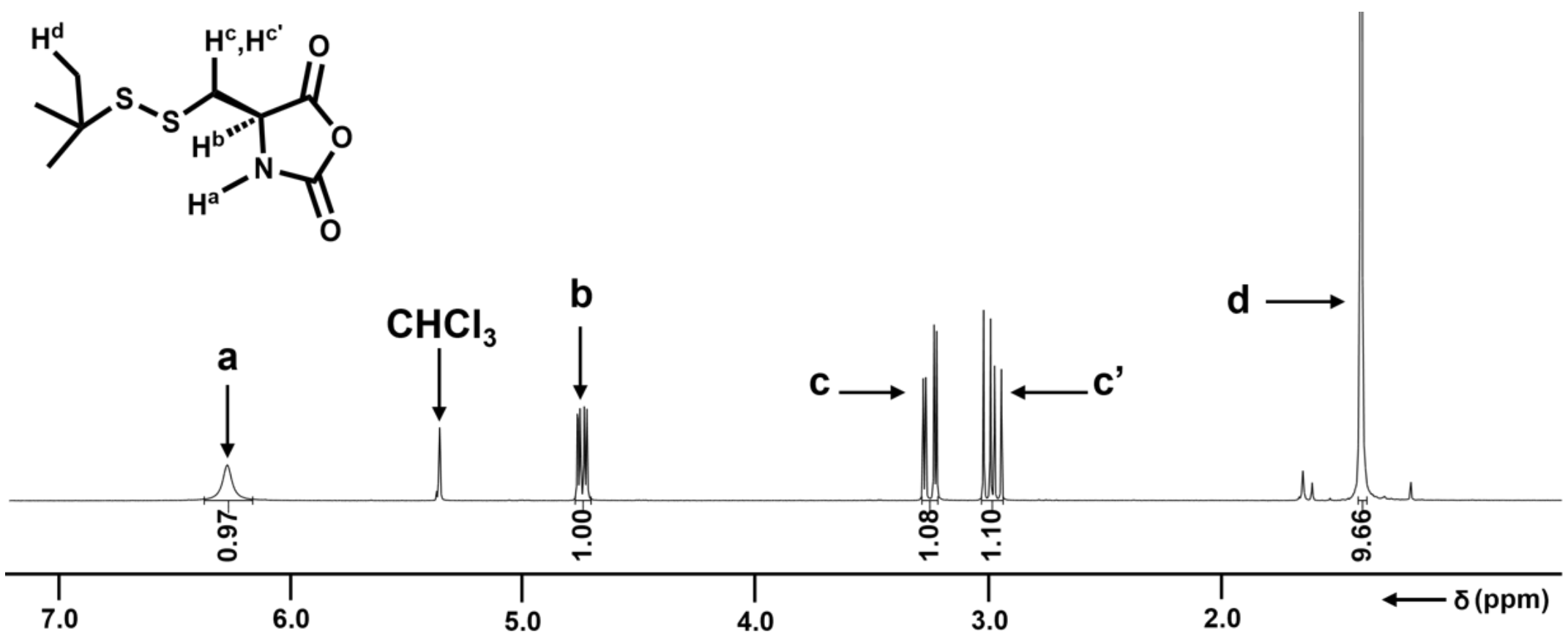

Figure S2. ^{1}H NMR of *t*-But-Cys-NCA (300 MHz, $CDCl_3$).

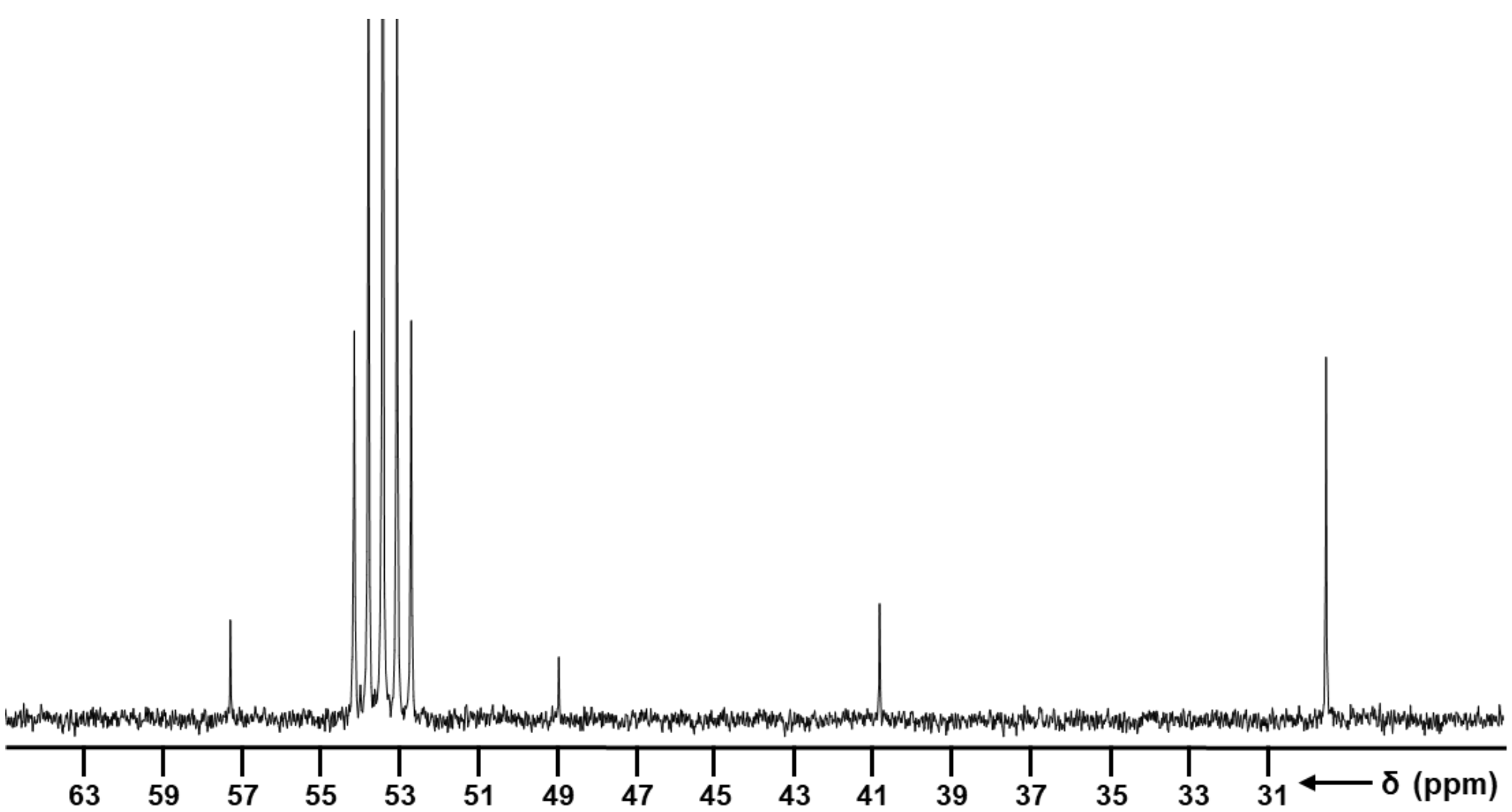

Figure S3. ^{13}C NMR of *t*-But-Cys-NCA (300 MHz, $CDCl_3$).

2. Polymer synthesis and characterization

General methods. All reagents and starting materials were purchased from Aldrich, TCI, or VWR and used without further purification unless otherwise noted. Poly(*β*-AcO-Gal-Thr)$_m$ was prepared following previously reported protocols[1]. The solvents for the polymerizations were freshly dried with NaH and benzophenone as an indicator. LiHMDS was purified by sublimation within 24 h of the polymerization.[3] ^{1}H NMR spectra were obtained on a Bruker 300 MHz spectrometer. MALDI-TOF mass spectrometry of poly(*β*-AcO-Gal-Thr)$_m$-*r*-poly(*t*-But-Cys-H)$_n$ were taken on a Bruker Autoflex Speed TOF-TOF. For the matrix-assisted laser desorption ionization–time of flight (MALDI-ToF) mass spectrometry, 50 µL of 3 mg/mL solution of polymers was mixed with 1 mL of 10 % 2,5-dihydroxybenzoic acid (DHB) in CH_2Cl_2. Gel-permeation chromatography (GPC) was performed by diluting a 60 µL aliquot from the reaction vessel into 0.5 mL of the mobile phase (1:1 DMF:DMSO). The sample was passed through a 0.45 µm PTFE filter (VWR 28145-493), and 50 µL was injected into the GPC instrument. The GPC (Shimadzu) is comprised of CBM-40 controller, DGU-405 degassing unit, 2x LC-20AR pumps, a CTO-40C column oven and the following columns: Shodex™ OHpak[4], SB-G-6B (No. B2080109), SB-803-HQ (No. L2070430), SB-806-HQ (No. L2070220). Molecular weight analysis was conducted with in-line RID-20A refractive index detector (Shimadzu). Samples were analyzed at a flow rate of 0.25 mL/min at 60 °C. The method was calibrated using Dextran 0.18 – 298 kDa ReadyCal™ standard, polymer standard service (PSS:PSS-PULKITR1H), **Figure S4**). The calibration curve of the calibration standards was fitted with a 7th order polynomial + hyperbolic curve model[5] (**Figure S5**). Results were analyzed using LabSolutions GPC Postrun analysis software (Shimadzu).

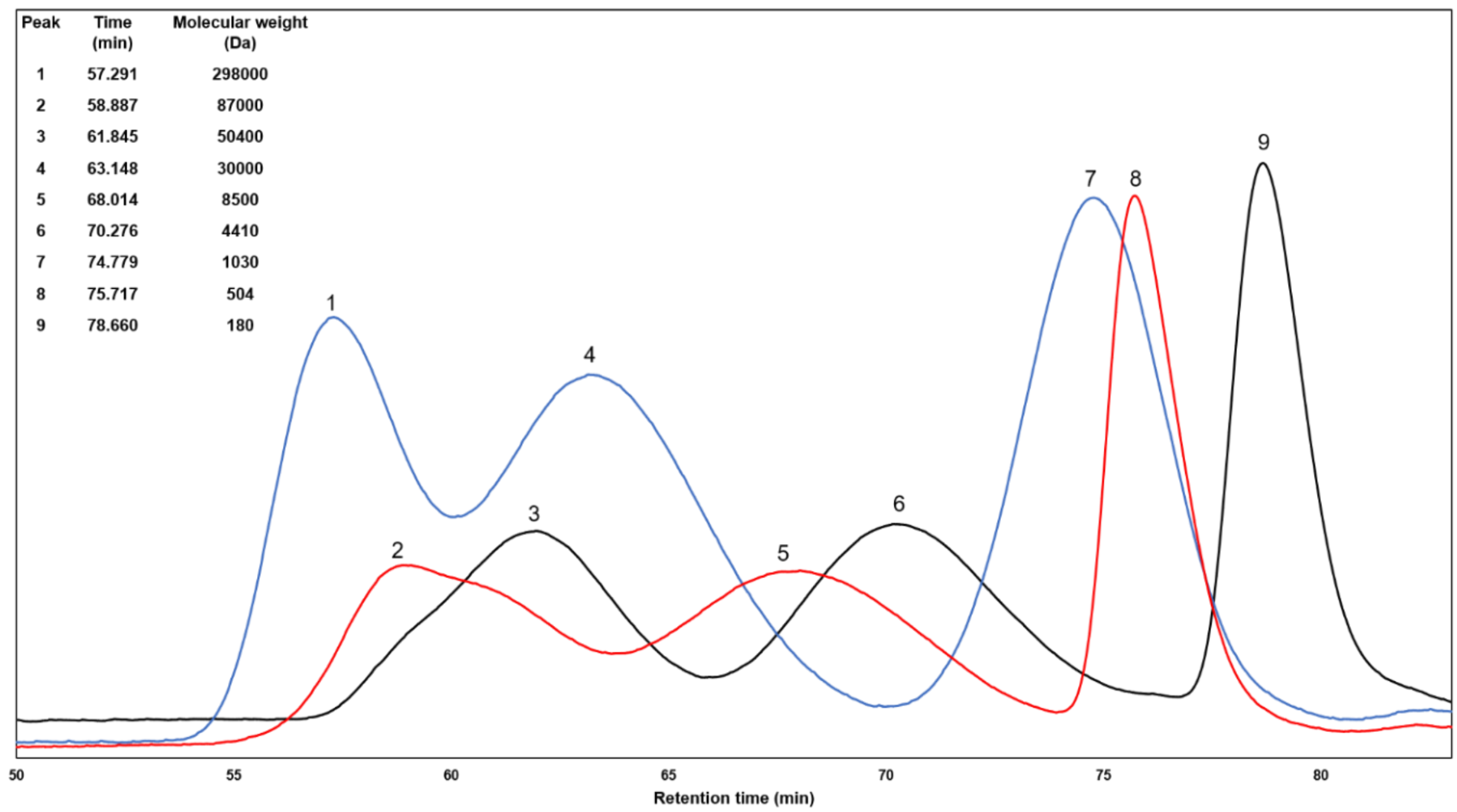

Figure S4. GPC spectra for the Dextran 0.18 – 298 kDa ReadyCal™ standard calibration standards. Standard A (blue line) corresponds to sample with 298 kDa (peak 1), 30.0 kDa (peak 4) and 1.03 kDa (peak 7). Standard B (red line) corresponds to sample with 87.0 kDa (peak 2), 8.50 kDa (peak 5) and 0.50 kDa (peak 8). Standard C (black line) corresponds to sample with 50.4 kDa (peak 3), 4.41 kDa (peak 6) and 0.18 kDa (peak 9).

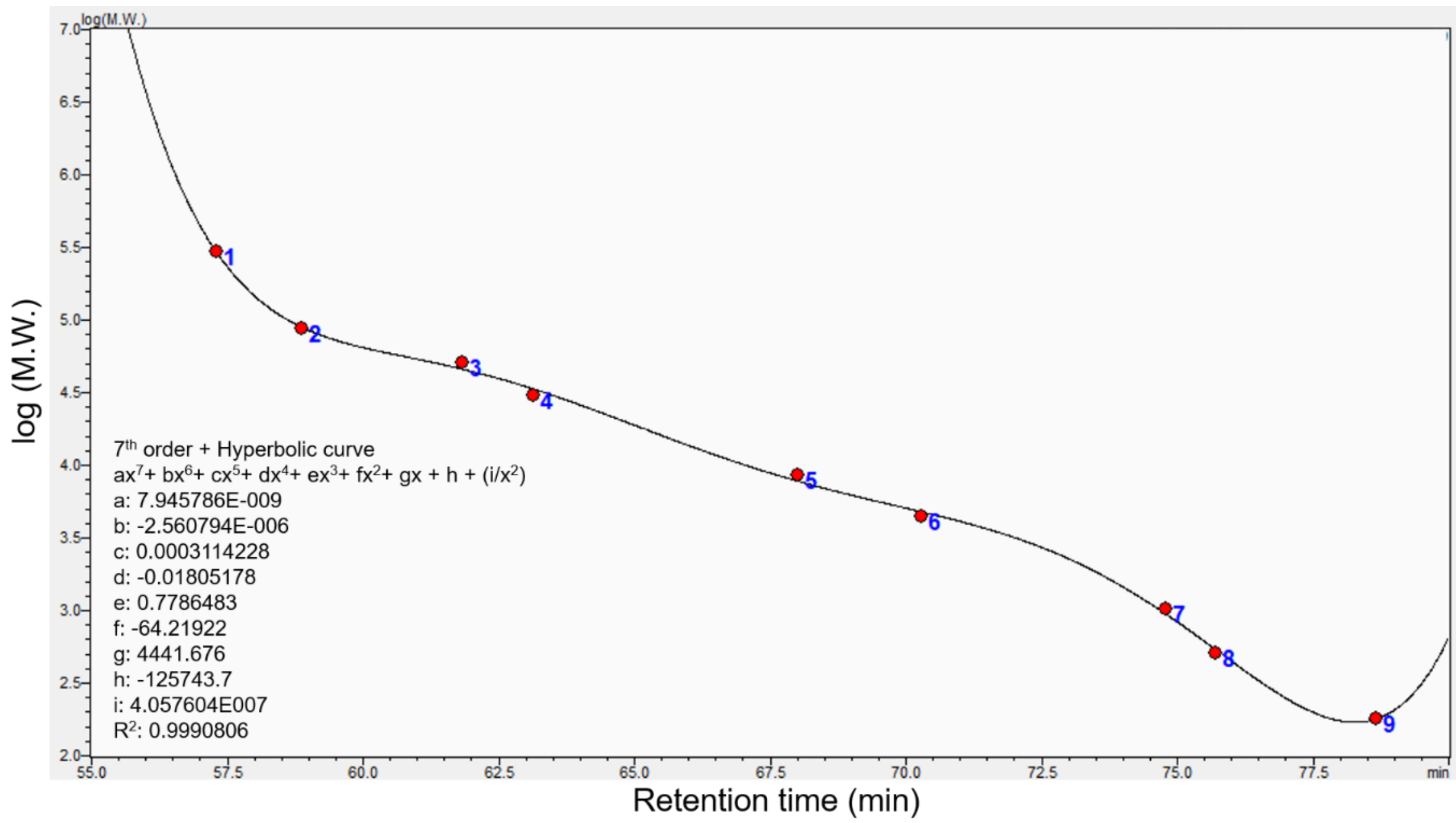

Figure S5. Calibration curve for the GPC calibration standards (ReadyCal™-Dextran, M_w: 0.18 – 298 kDa).

General Polymerization Procedure for Poly(*β*-Gal-Thr)$_m$-*r*-poly(Cys-H)$_n$

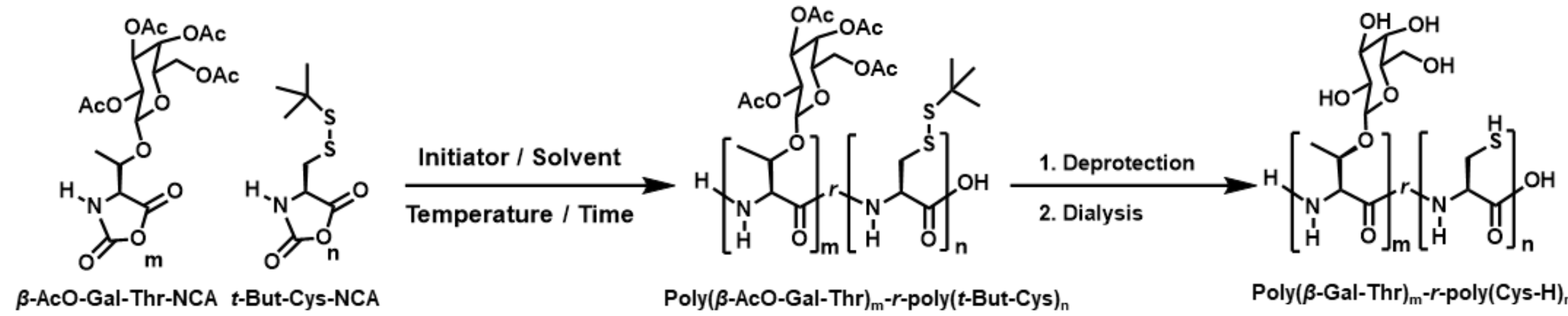

Each polymerization solution was prepared inside a glove box using the following general procedure, and details of individual polymerization reactions are given in **Table S1**. *β*-AcO-Gal-Thr-NCA was dissolved in dry THF to prepare a 0.1 M solution, and then the desired amount of *t*-But-Cys-NCA was added to the reaction flask. The initiator solution (0.1 M in THF) was added to the Schlenk flask. The reaction vessel was sealed, removed from the glove box, and heated at 50 °C under Ar. The reaction was monitored by removing 10 μL aliquots and diluting in DMF/DMSO [1:1], and 0.3 mL of the aliquot was injected into the gel permeation chromatography (GPC) instrument. Polymerization progress and monomer consumption were monitored by GPC analysis, and reactions were considered complete when no further monomer consumption was observed. Upon completion of the reaction (no change in M_n), the solvent (THF) was removed under reduced pressure. The reaction mixture was subjected first to the deprotection of the *t*-butyl thiol groups, then the acetate groups by dissolving in H_2O (10 mL) with 0.5% w/w dithiothreitol (DTT) and stirring for 12 h. MeOH (10 mL) was added to the solution, followed by the addition of K_2CO_3 (0.02 mol, 4.0 g), and the mixture was stirred for 2 days. The deprotected polymer was purified by membrane dialysis (M_w cutoff: 3.5 KDa), lyophilized to remove H_2O, and characterized by mass spectroscopy matrix-assisted laser desorption ionization–time of flight (MALDI-ToF), ^{1}H NMR, and GPC

Table S1. Reaction conditions for the polymerization of *β*-AcO-Gal-Thr-NCA and *t*-But-Cys-NCA.

Entry	[a]Initiator	Mass (mg)	Catalyst	[b]Solvent	T (°C)	Time (h)	Theoretical m:n:i	Experimental m:n	[e]Protected M_n (kDa)	[f]Deprotected M_n (kDa)	[f]M_w/M_n (Đ)	$\bar{X}_n$[f]	[g]Yield %
1	LiHMDS	50	HFAB	CH_2Cl_2	25	192	25:0:1	---	9.21	6.10	1.22	23	78
2	TBAA	50	HFAB	CH_2Cl_2	25	144	25:0:1	---	7.97	5.33	1.61	20	56
3	TBAA	50	---	CH_2Cl_2	25	72	25:0:1	---	7.57	5.22	1.42	20	45
4	TBAA	50	---	MeCN	70	192	25:1.25:1	---	---	---	---	---	---
5	TBAA	50	---	MeCN	0	192	25:1.25:1	---	---	---	---	---	---
6	TBAA	50	---	MeCN	25	192	25:1.25:1	---	---	---	---	---	---
7	TBAA	50	---	MeCN	25	192	25:1.25:1	---	---	---	---	---	---
8	TBAA	400	---	CH_2Cl_2	25	144	25:0.025:1	22:1	9.29	5.63	1.35	23	67
9	TBAA	400	---	CH_2Cl_2	25	144	25:1:1	22:2	10.3	6.24	1.33	24	70
10	TBAA	400	---	CH_2Cl_2	25	144	25:1.25:1	23:3	10.5	6.46	1.31	26	72
11	TBAA	400	---	CH_2Cl_2	25	144	25:2.5:1	23:6	11.2	6.66	1.25	29	71
12	TBAA	50	---	CH_2Cl_2	25	144	50:5:1	34:6	15.9	9.85	1.40	40	68
13	TBAA	50	---	CH_2Cl_2	25	168	75:0:1	---	27.2	16.6	1.32	63	77
14	TBAA	50	---	CH_2Cl_2	25	192	100:0:1	---	29.3	17.9	1.38	68	59
15	LiHMDS	100	---	THF	25	144	25:0:1	---	7.10	3.69	1.21	14	23
16	LiHMDS	100	HFAB	THF	25	96	25:2.5:1	23:6	11.4	6.72	1.14	29	74
17		100	$Co(PMe_3)_4$	THF	25	144	25:0:1	---	---	--	---	---	---
18		100	$Co(PMe_3)_4$	THF	50	144	25:0:1	---	41.1	22.7	1.03	86	10
19	LiHMDS	2000	---	THF	50	144	25:0.025:1	23:1	9.31	5.35	1.25	24	65
20	LiHMDS	2000	---	THF	50	144	25:1:1	23:2	9.89	6.18	1.26	25	69
21	LiHMDS	2000	---	THF	50	144	25:1.25:1	23:3	10.3	6.37	1.21	26	67
22	LiHMDS	2000	---	THF	50	144	25:2.5:1	23:6	10.9	6.71	1.25	29	71
23	LiHMDS	2000	---	THF	50	144	50:2.5:1	33:3	14.8	9.02	1.18	36	63
24	LiHMDS	2000	---	THF	50	216	75:2.5:1	62:3	27.4	16.7	1.23	65	56
25	LiHMDS	2000	---	THF	50	288	100:2.5:1	78:4	34.5	20.9	1.21	82	73

[a][Initiator] = 0.1M (LiHMDS (lithium bis(trimethylsilyl)amide), TBAA (*t*-But acetoacetate)), [Catalyst] = 0.1 M HFAB (1,3-bis(2-hydroxyhexafluoroisopropyl)benzene), $Co(PMe_3)_4$ (tetrakis(trimethylphosphane)cobalt(0)). [b]Dry (molecular sieves, freeze-pump-thaw, and purging), [c]Reflux, [d]Closed system, [e]M_w, M_n, M_w/M_n (***Đ***), and $\bar{X}_n$ determined on the protected polymers, poly(*β*-AcO-Gal-Thr) and poly(*β*-AcO-Gal-Thr)$_m$-*r*-poly(*t*-But-Cys)$_n$, by GPC (Calibration standard: ReadyCal™-Dextran, M_w: 0.18 – 298 kDa). [f]M_w, M_n, M_w/M_n (***Đ***), and $\bar{X}_n$ determined on the deprotected polymer, poly(*β*-Gal-Thr)$_n$ and poly(*β*-Gal-Thr)$_n$-*r*-poly(Cys-H)$_m$ by GPC (Calibration standard: ReadyCal™-Dextran, M_w: 0.18 – 298 kDa). [g]Yields % are reported for the deprotected polymers.

^{1}H NMR, GPC, and MALDI-TOF MS Characterization

General methods. All ^{1}H NMR were taken using DMSO-d6 purchased from Cambridge Isotope Laboratories (Cat. No. 101322-296). The GPC was taken after lyophilization. For GPC analysis, the powder polymer was dissolved in 0.5 mL of DMF:DMSO [1:1], and the sample was injected into the GPC instrument. Matrix-assisted laser desorption ionization–time of flight (MALDI-ToF) mass spectrometry samples were prepared by dissolving 50 μL of 3 mg/mL solution of polymers and mixing with 1 mL of 10 % 2,5-dihydroxybenzoic acid (DHB) in CH_2Cl_2.

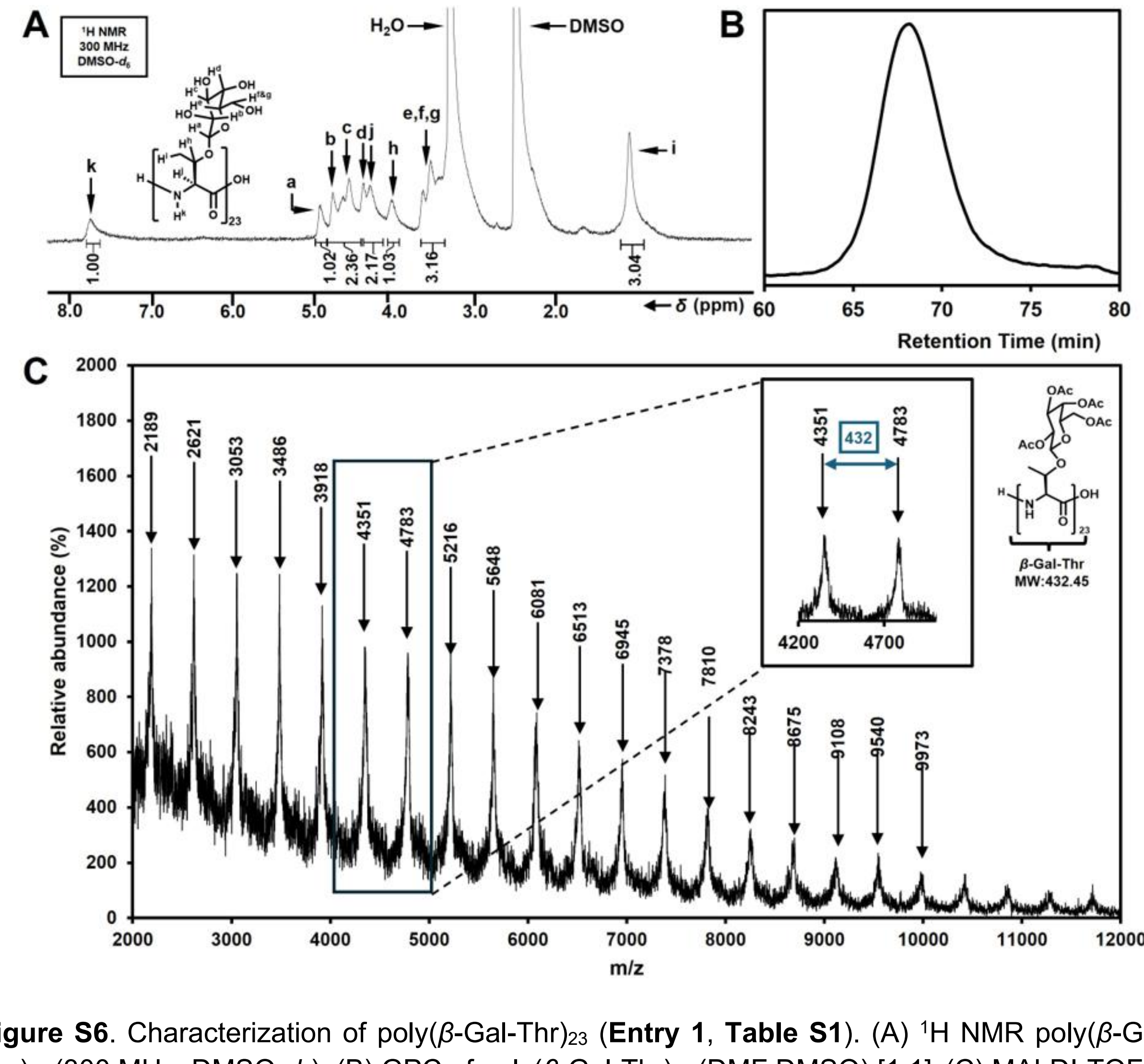

Figure S6. Characterization of poly(β-Gal-Thr)$_{23}$ (**Entry 1**, **Table S1**). (A) ^{1}H NMR poly(β-Gal-Thr)$_{23}$ (300 MHz, DMSO-d_6). (B) GPC of poly(β-Gal-Thr)$_{23}$ (DMF:DMSO) [1:1]. (C) MALDI-TOF of poly(β-AcO-Gal-Thr)$_{23}$.

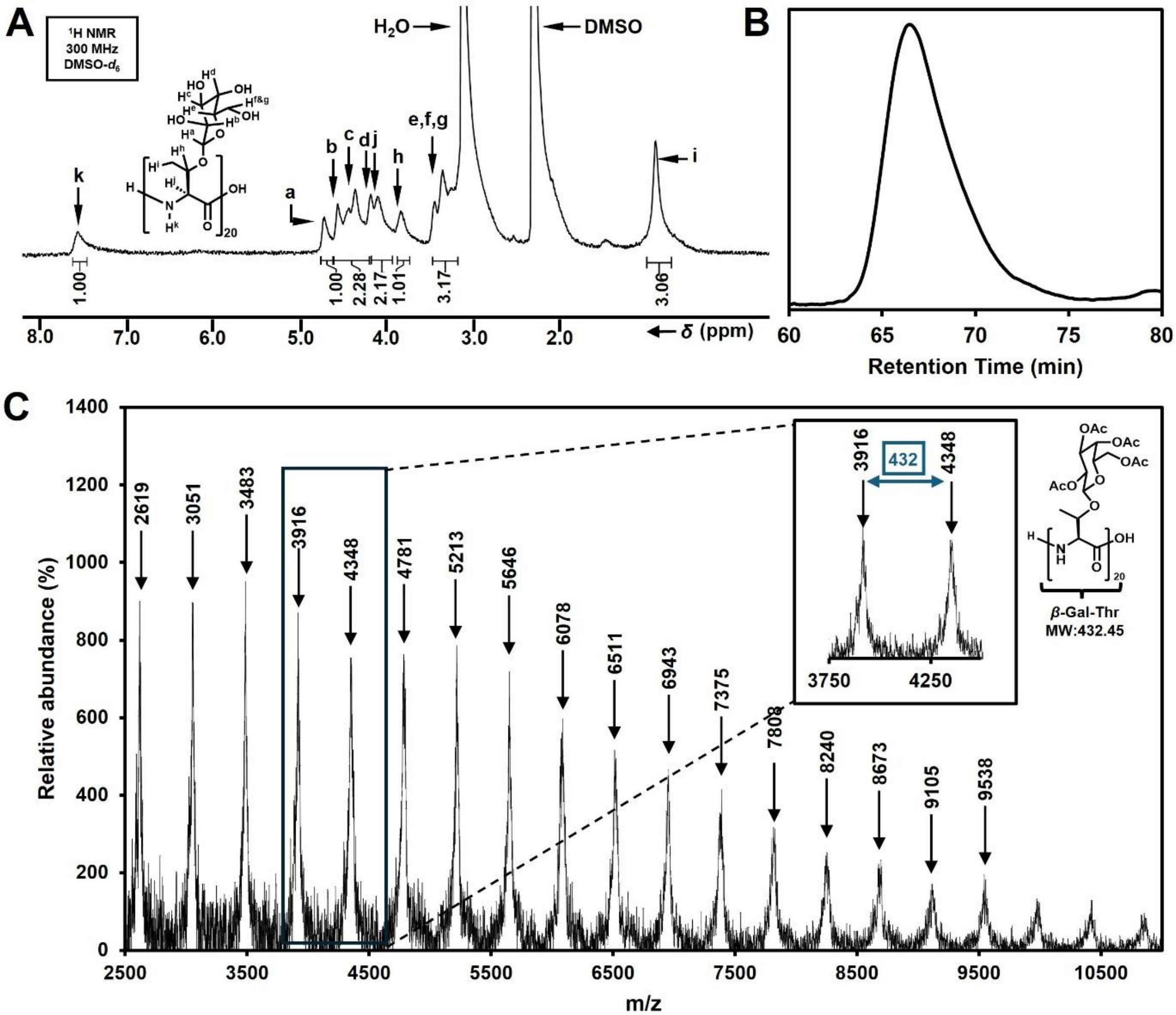

Figure S7. Characterization of poly(β-Gal-Thr)$_{20}$ (**Entry 2, Table S1**). (A) ^{1}H NMR poly(β-Gal-Thr)$_{20}$ (300 MHz, DMSO-d_6). (B) GPC of poly(β-Gal-Thr)$_{20}$ (DMF:DMSO) [1:1]. (C) MALDI-TOF of poly(β-AcO-Gal-Thr)$_{20}$.

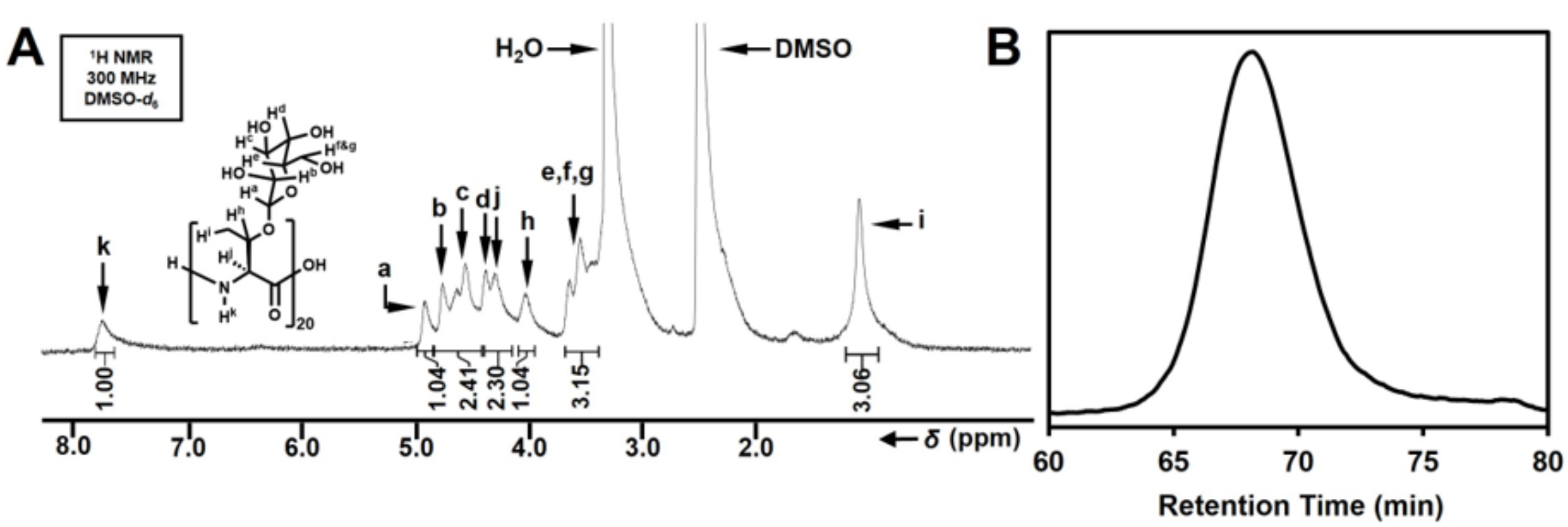

Figure S8. Characterization of poly(β-Gal-Thr)$_{20}$ (**Entry 3, Table S1**). (A) ^{1}H NMR poly(β-Gal-Thr)$_{20}$ (300 MHz, DMSO-d_6). (B) GPC of poly(β-Gal-Thr)$_{20}$ (DMF:DMSO) [1:1].

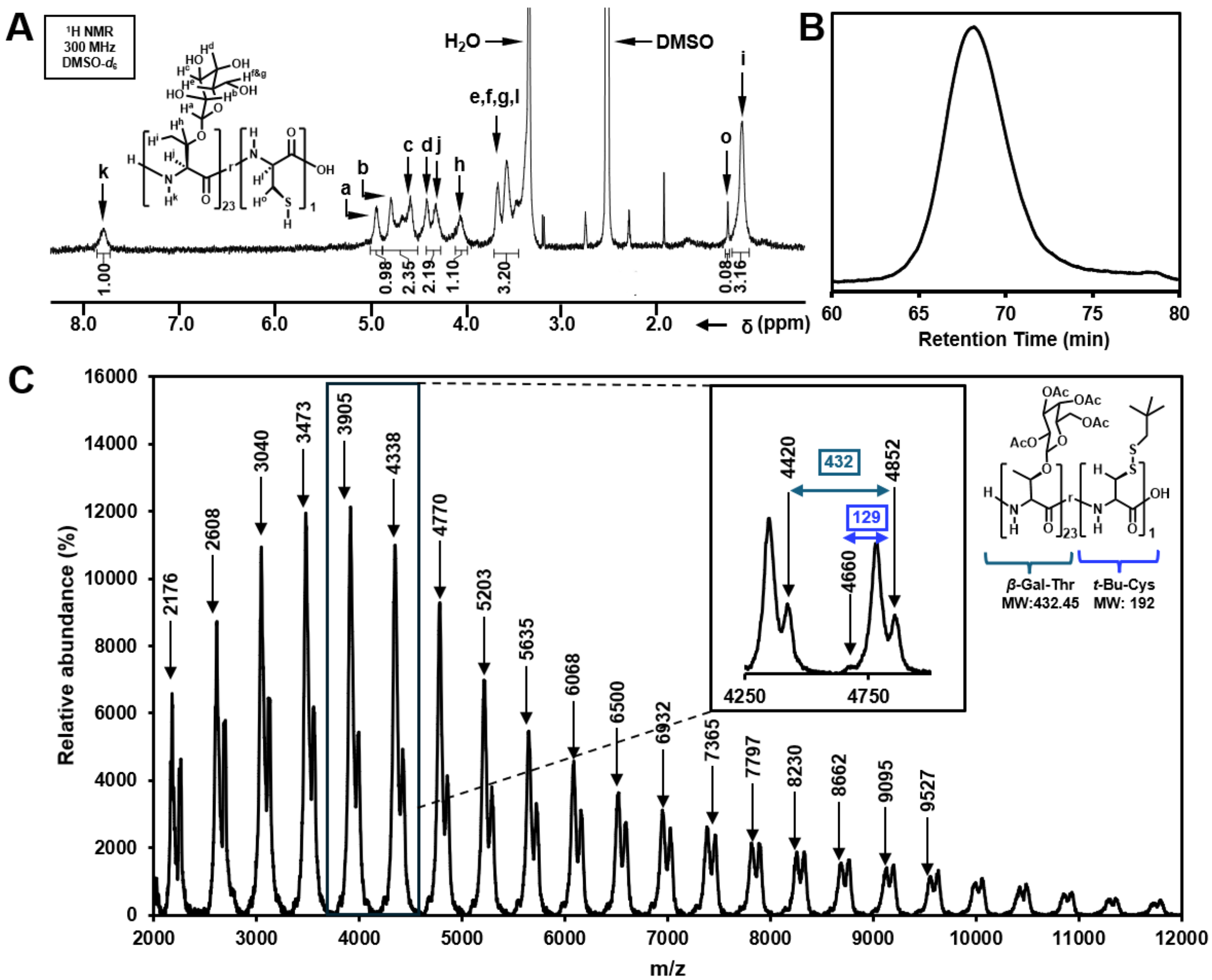

Figure S9. Characterization of poly(β-Gal-Thr)$_{23}$-*r*-poly(Cys-H)$_1$ (**Entry 8, Table S1**). (A) ^{1}H NMR poly(β-Gal-Thr)$_{23}$-*r*-poly(Cys-H)$_1$ (300 MHz, DMSO-d_6). (B) GPC of poly(β-Gal-Thr)$_{23}$-*r*-poly(Cys-H)$_1$ (DMF:DMSO) [1:1]. (C) MALDI-TOF of poly(β-AcO-Gal-Thr)$_{23}$-*r*-poly(*t*-But-Cys)$_1$.

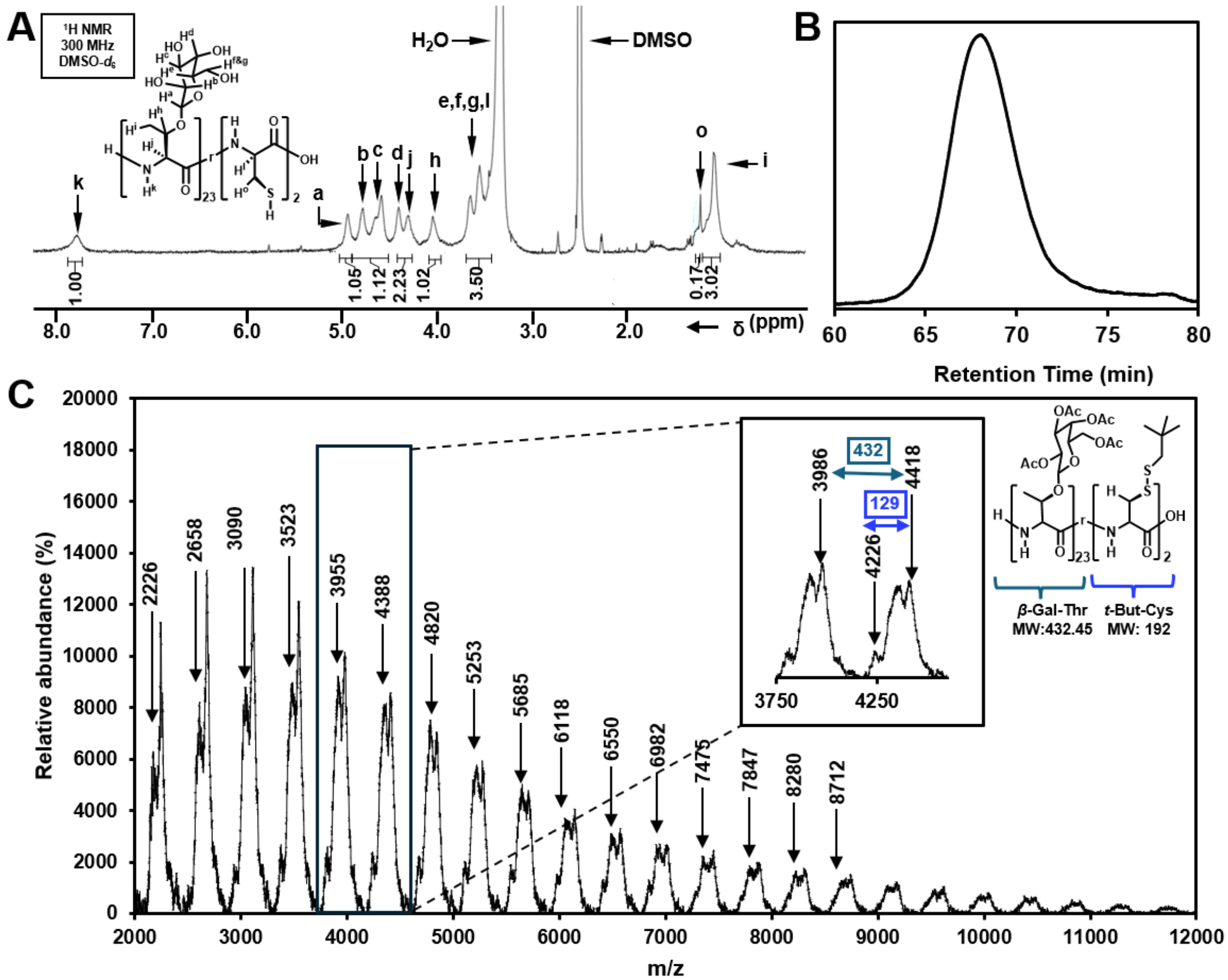

Figure S10. Characterization of poly(β-Gal-Thr)$_{23}$-*r*-poly(Cys-H)$_2$ (**Entry 9**, **Table S1**). (A) ^{1}H NMR poly(β-Gal-Thr)$_{23}$-*r*-poly(Cys-H)$_2$ (300 MHz, DMSO-d_6). (B) GPC of poly(β-Gal-Thr)$_{23}$-*r*-poly(Cys-H)$_2$ (DMF:DMSO) [1:1]. (C) MALDI-TOF of poly(β-AcO-Gal-Thr)$_{23}$-*r*-poly(*t*-But-Cys)$_2$.

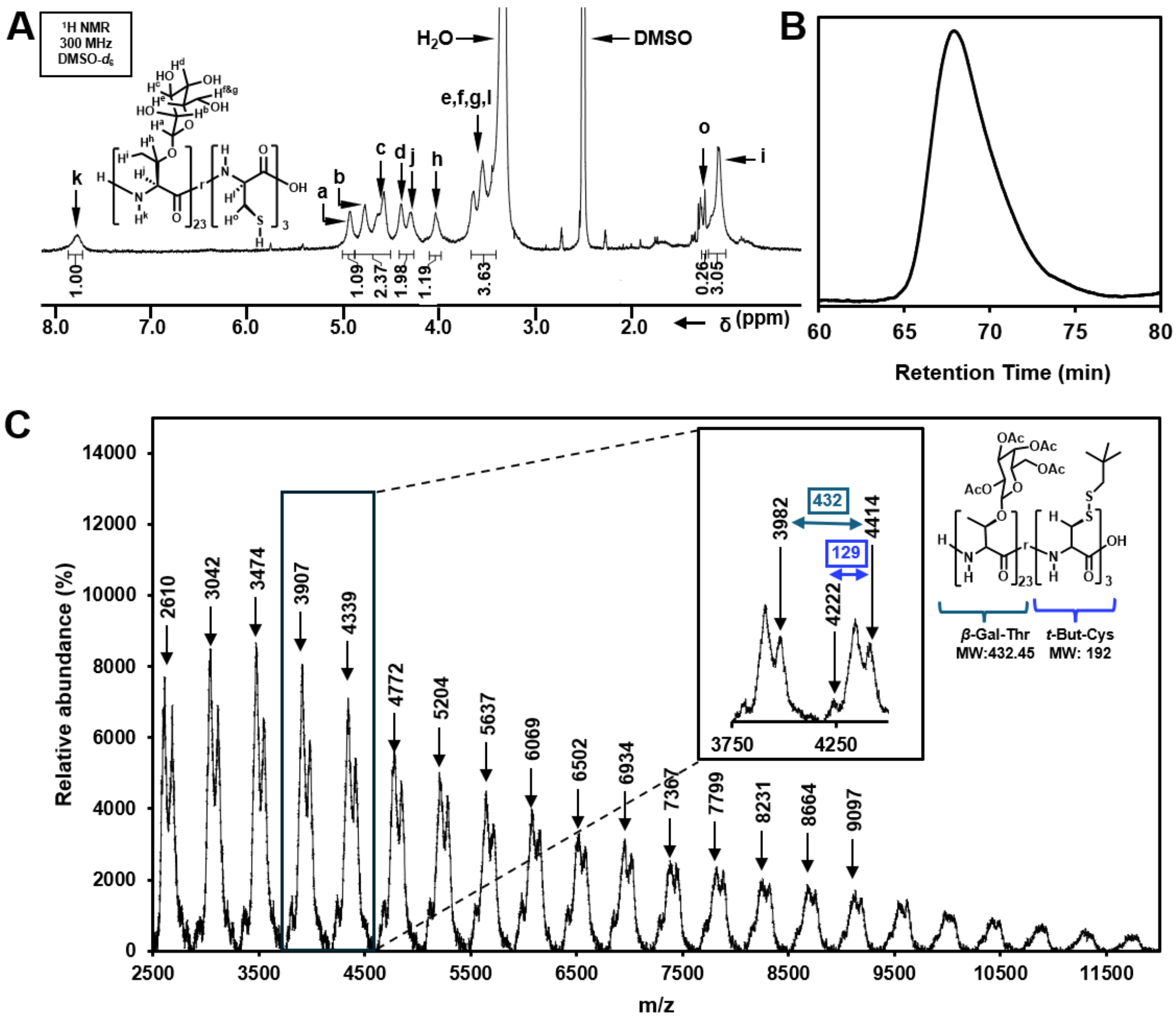

Figure S11. Characterization of poly(β-Gal-Thr)$_{23}$-*r*-poly(Cys-H)$_3$ (**Entry 10, Table S1**). (A) ^{1}H NMR poly(β-Gal-Thr)$_{23}$-*r*-poly(Cys-H)$_3$ (300 MHz, DMSO-d_6). (B) GPC of poly(β-Gal-Thr)$_{23}$-*r*-poly(Cys-H)$_3$ (DMF:DMSO) [1:1]. (C) MALDI-TOF of Poly(β-AcO-Gal-Thr)$_{23}$-*r*-poly(*t*-But-Cys)$_3$.

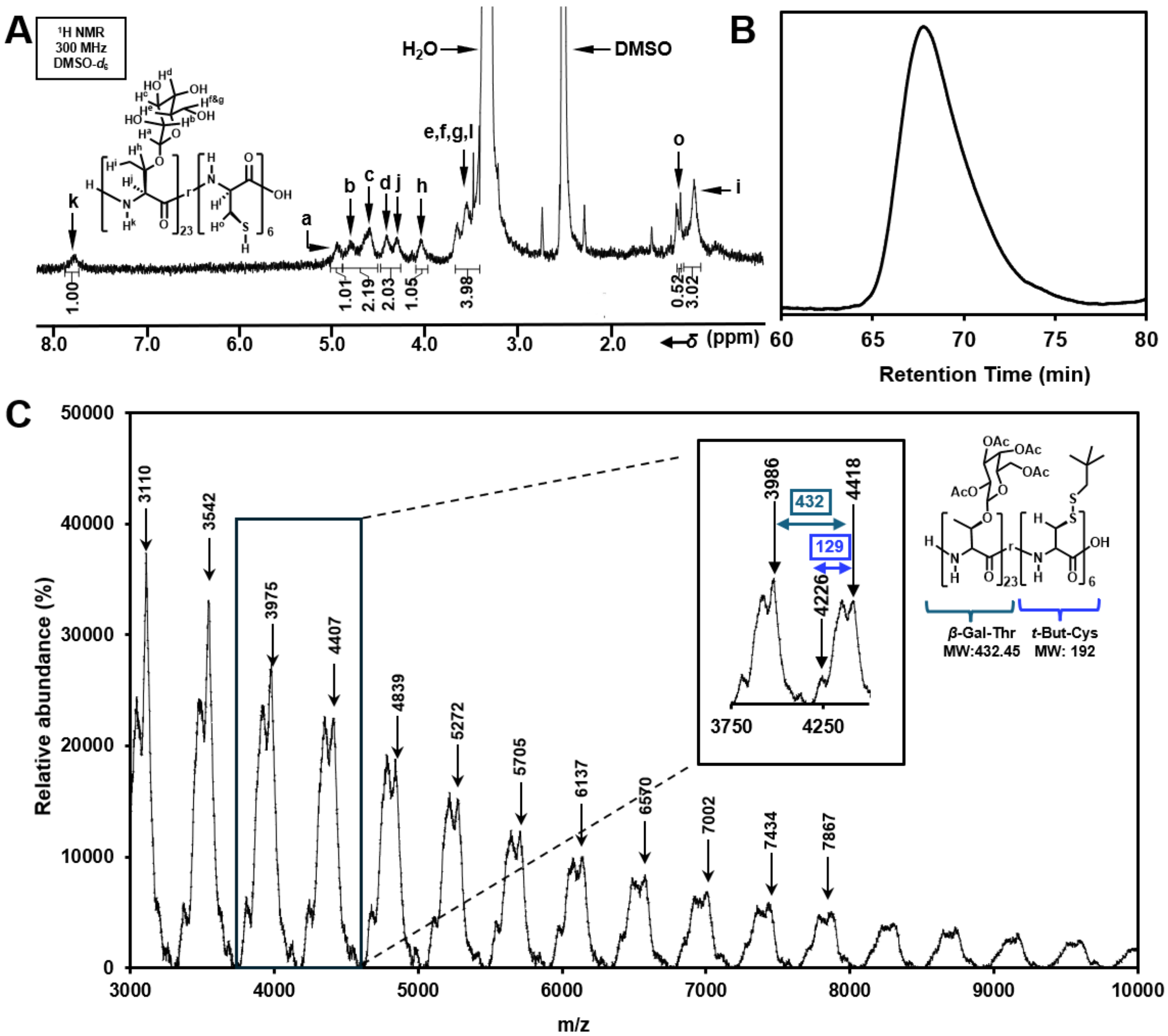

Figure S12. Characterization of poly(β-Gal-Thr)$_{23}$-*r*-poly(Cys-H)$_6$ (**Entre 11, Table S1**). (A) ^{1}H NMR poly(β-Gal-Thr)$_{23}$-*r*-poly(Cys-H)$_6$ (300 MHz, DMSO-d_6). (B) GPC of poly(β-Gal-Thr)$_{23}$-*r*-poly(Cys-H)$_6$ (DMF:DMSO) [1:1]. (C) MALDI-TOF of Poly(β-AcO-Gal-Thr)$_{23}$-*r*-poly(*t*-But-Cys)$_6$.

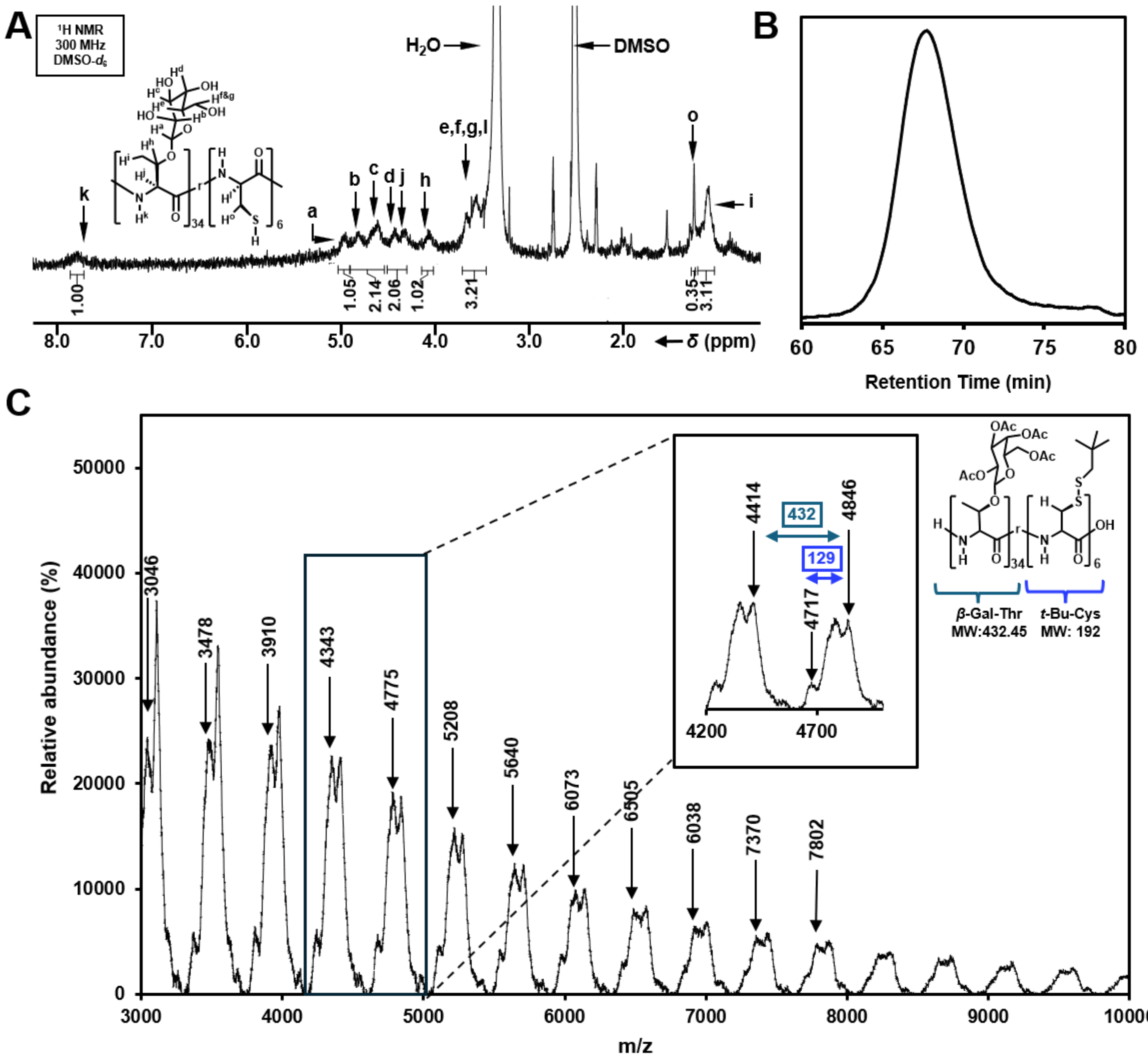

Figure S13. Characterization of Poly(β-Gal-Thr)$_{34}$-*r*-poly(Cys-H)$_6$ (**Entry 12, Table S1**). (A) ^{1}H NMR poly(β-Gal-Thr)$_{34}$-*r*-poly(Cys-H)$_6$ (300 MHz, DMSO-d_6). (B) GPC of poly(β-Gal-Thr)$_{34}$-*r*-poly(Cys-H)$_6$ (DMF:DMSO) [1:1]. (C) MALDI-TOF of Poly(β-AcO-Gal-Thr)$_{34}$-*r*-poly(*t*-But-Cys)$_6$.

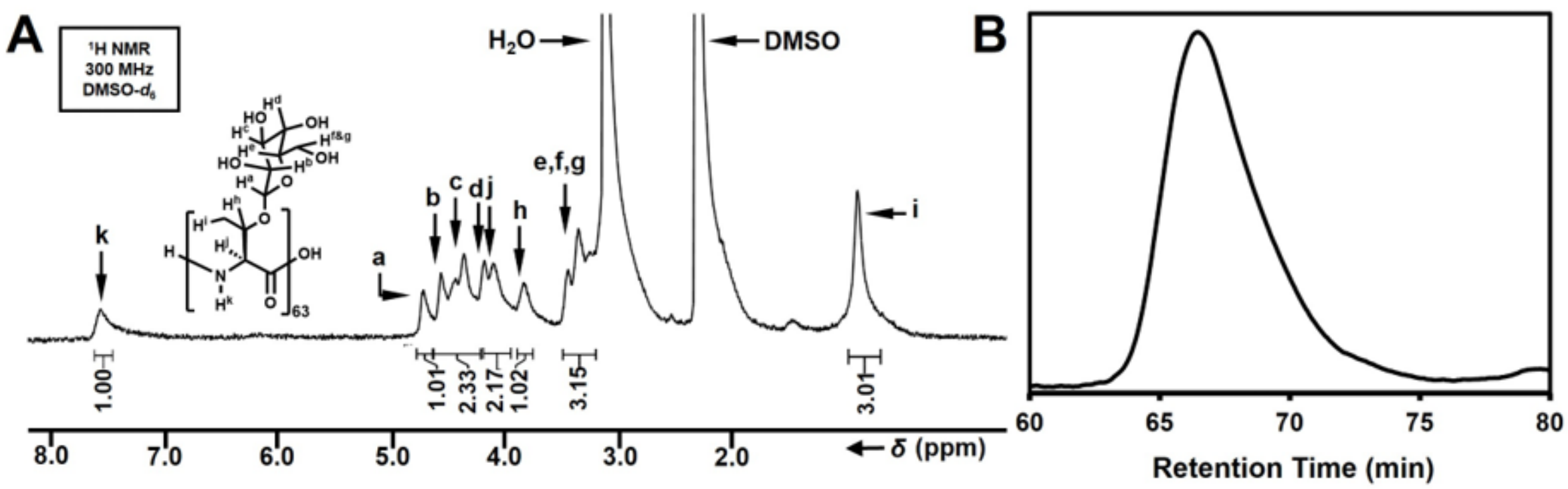

Figure S14. Characterization of poly(β-Gal-Thr)$_{63}$ (**Entry 13, Table S1**). (A) ^{1}H NMR poly(β-Gal-Thr)$_{63}$ (300 MHz, DMSO-d_6). (B) GPC of Poly(β-Gal-Thr)$_{63}$ (DMF:DMSO) [1:1]. (C) MALDI-TOF of poly(β-AcO-Gal-Thr)$_{63}$.

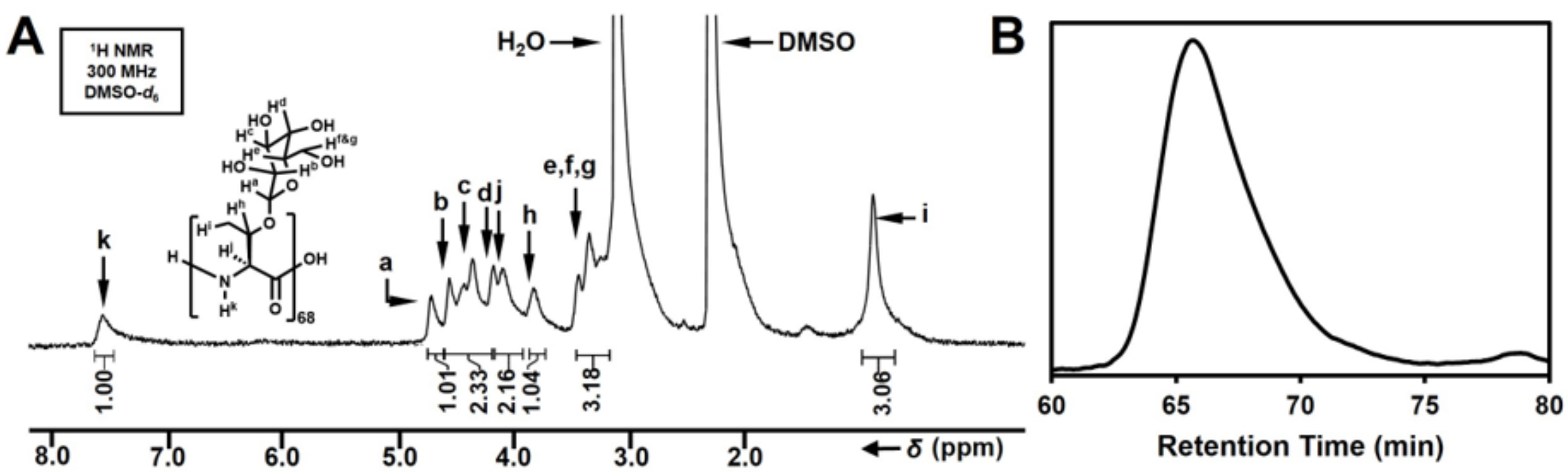

Figure S15. Characterization of poly(β-Gal-Thr)$_{68}$ (**Entry 14, Table S1**). (A) ^{1}H NMR poly(β-Gal-Thr)$_{68}$ (300 MHz, DMSO-d_6). (B) GPC of Poly(β-Gal-Thr)$_{68}$ (DMF:DMSO) [1:1]. (C) MALDI-TOF of poly(β-AcO-Gal-Thr)$_{68}$.

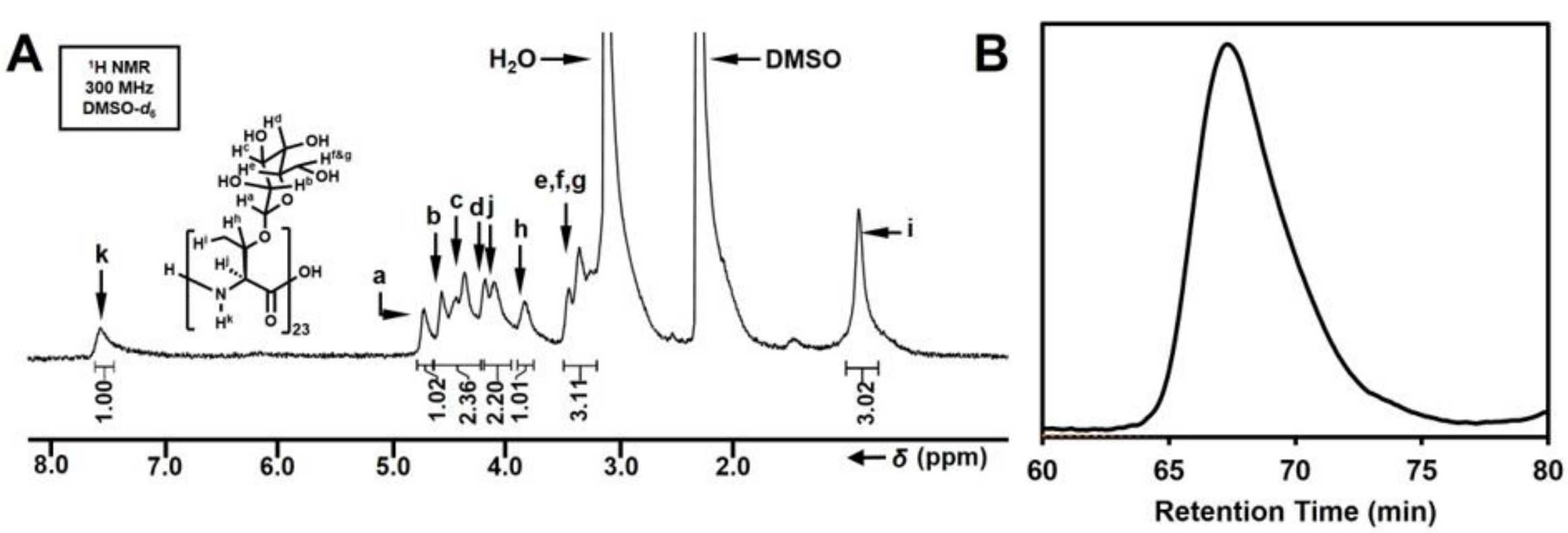

Figure S16. Characterization of poly(β-Gal-Thr)$_{23}$ (**Entry 15, Table S1**). (A) ^{1}H NMR Poly(β-Gal-Thr)$_{23}$ (300 MHz, DMSO-d_6). (B) GPC of Poly(β-Gal-Thr)$_{23}$ (DMF:DMSO) [1:1].

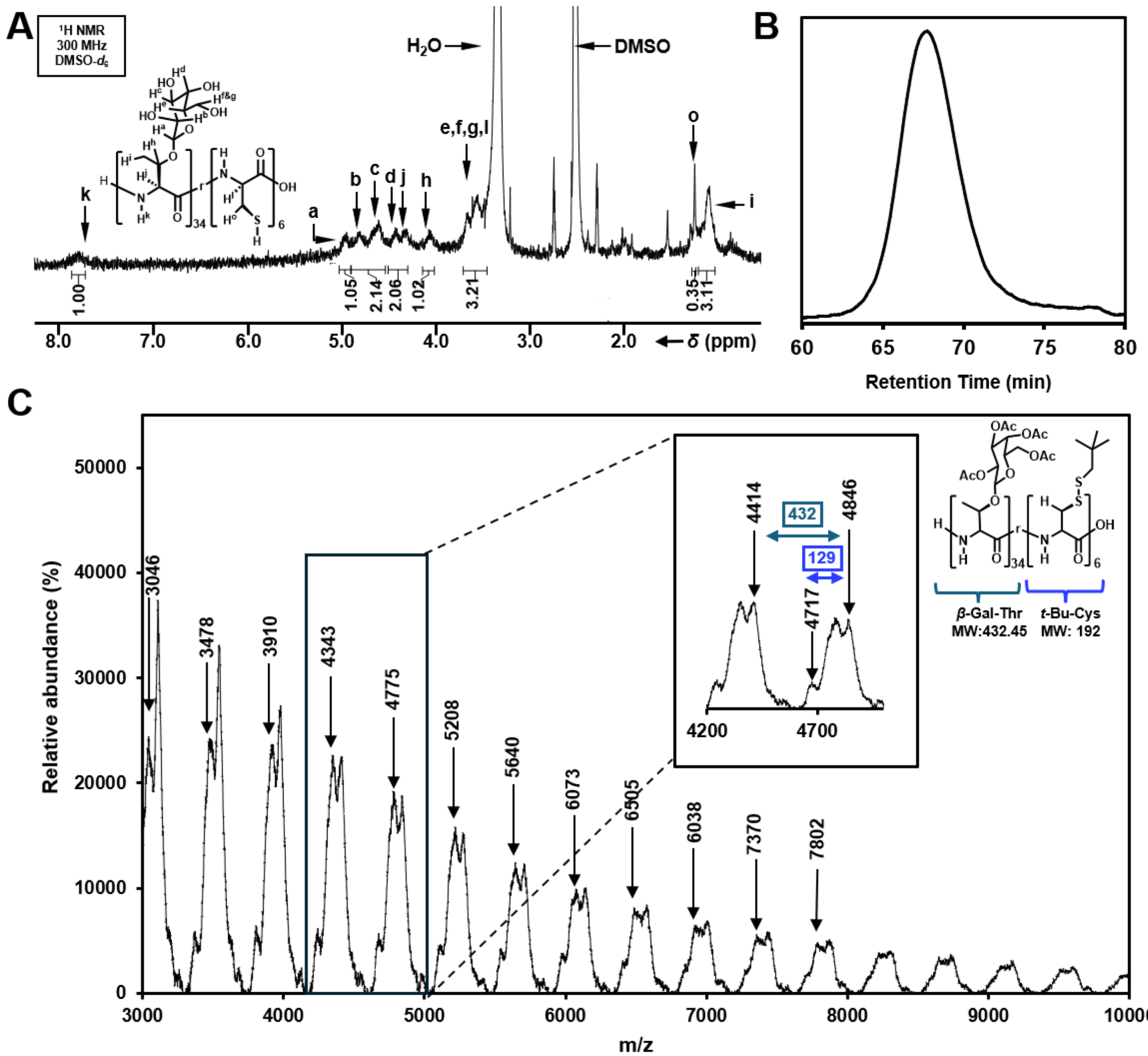

Figure S17. Characterization of poly(β-Gal-Thr)$_{23}$-*r*-poly(Cys-H)$_6$ (**Entry 16, Table S1**). (A) ^{1}H NMR Poly(β-Gal-Thr)$_{23}$-*r*-poly(Cys-H)$_6$ (300 MHz, DMSO-d_6). (B) GPC of poly(β-Gal-Thr)$_{23}$-*r*-poly(Cys-H)$_6$ (DMF:DMSO) [1:1]. **(C)** MALDI-TOF of Poly(β-AcO-Gal-Thr)$_{23}$-*r*-poly(*t*-But-Cys)$_6$.

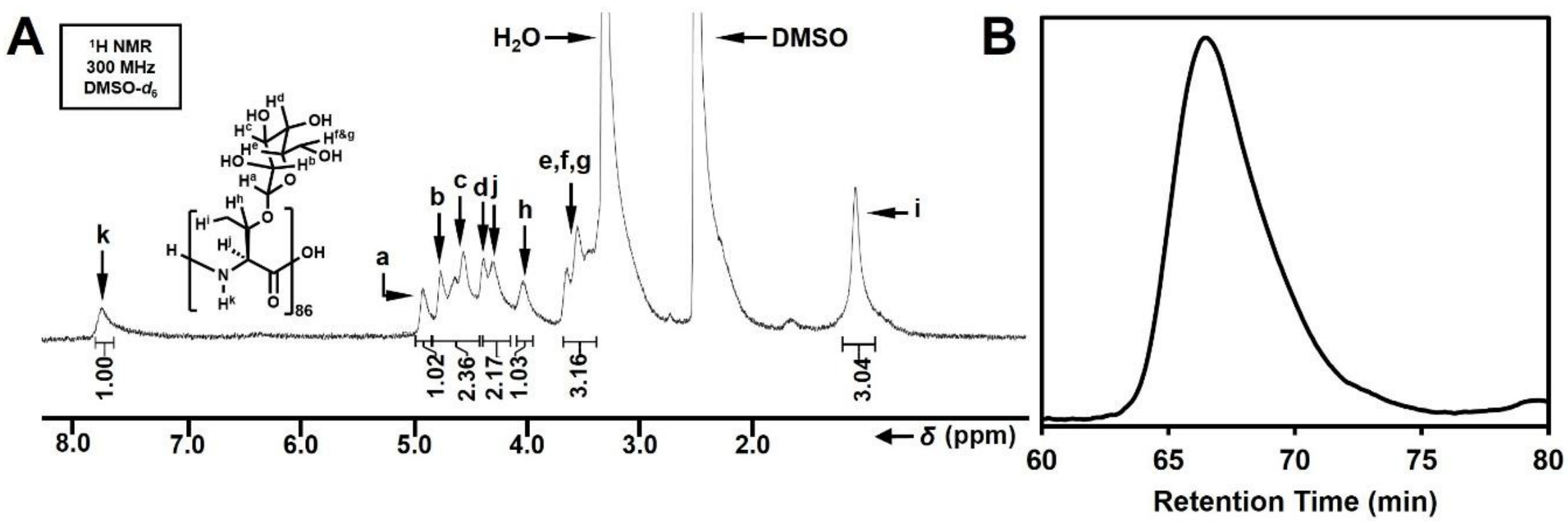

Figure S18. Characterization of poly(β-Gal-Thr)$_{86}$ (**Entry 18, Table S1**). (A) ^{1}H NMR poly(β-Gal-Thr)$_{86}$ (300 MHz, DMSO-d_6). (B) GPC of poly(β-Gal-Thr)$_{86}$ (DMF:DMSO) [1:1].

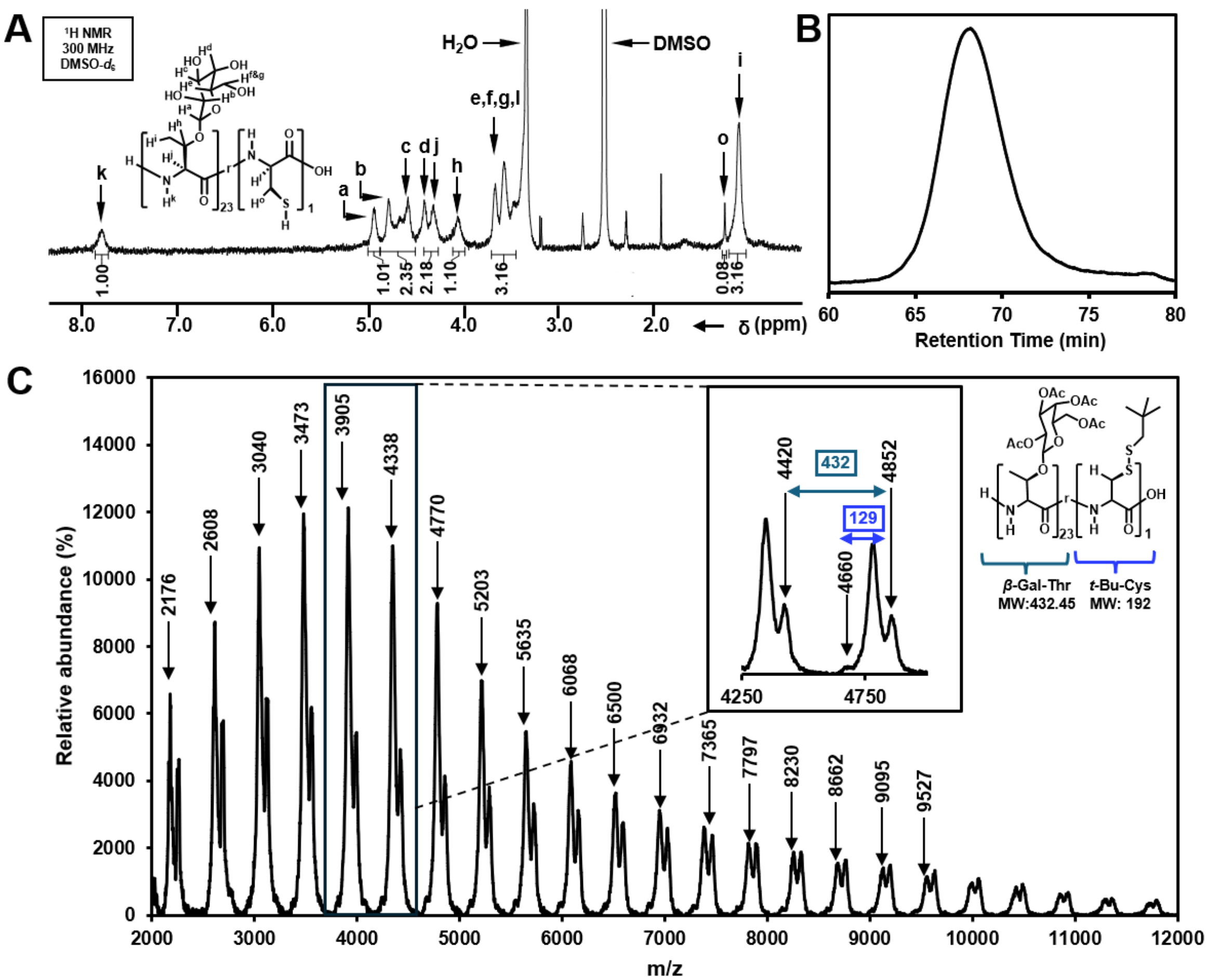

Figure S19. Characterization of poly(β-Gal-Thr)$_{23}$-*r*-poly(Cys-H)$_1$ (**Entry 19, Table S1**). (A) ^{1}H NMR Poly(β-Gal-Thr)$_{23}$-*r*-poly(Cys-H)$_1$ (300 MHz, DMSO-d_6). (B) GPC of poly(β-Gal-Thr)$_{23}$-*r*-poly(Cys-H)$_1$ (DMF:DMSO). (C) MALDI-TOF of poly(β-AcO-Gal-Thr)$_{23}$-*r*-poly(*t*-But-Cys)$_1$.

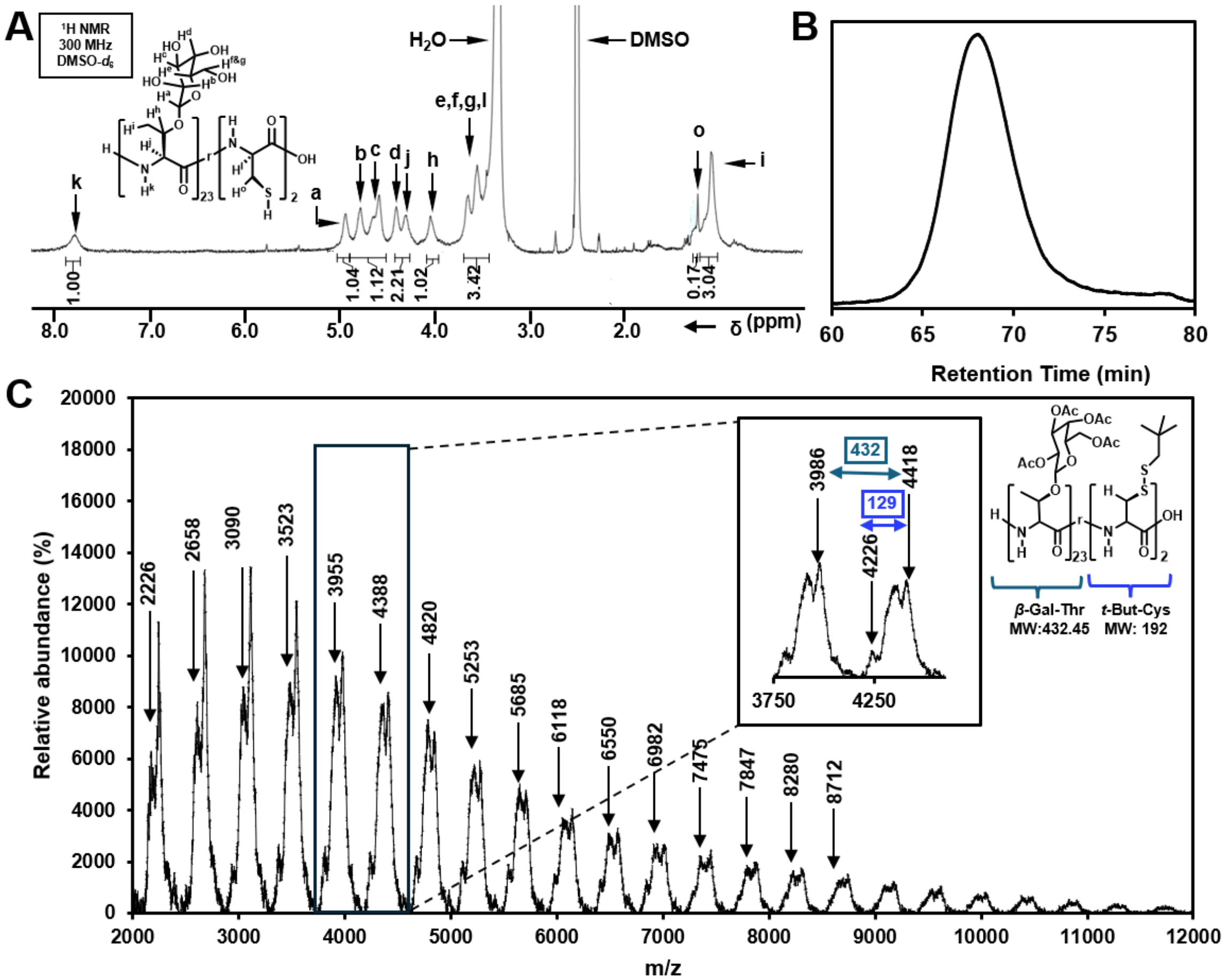

Figure S20. Characterization of poly(β-Gal-Thr)$_{23}$-*r*-poly(Cys-H)$_2$ (**Entry 20, Table S1**). (A) ^{1}H NMR poly(β-Gal-Thr)$_{23}$-*r*-poly(Cys-H)$_2$ (300 MHz, DMSO-d_6). (B) GPC of poly(β-Gal-Thr)$_{23}$-*r*-poly(Cys-H)$_2$ (DMF:DMSO) [1:1]. (C) MALDI-TOF of poly(β-AcO-Gal-Thr)$_{23}$-*r*-poly(*t*-But-Cys)$_2$.

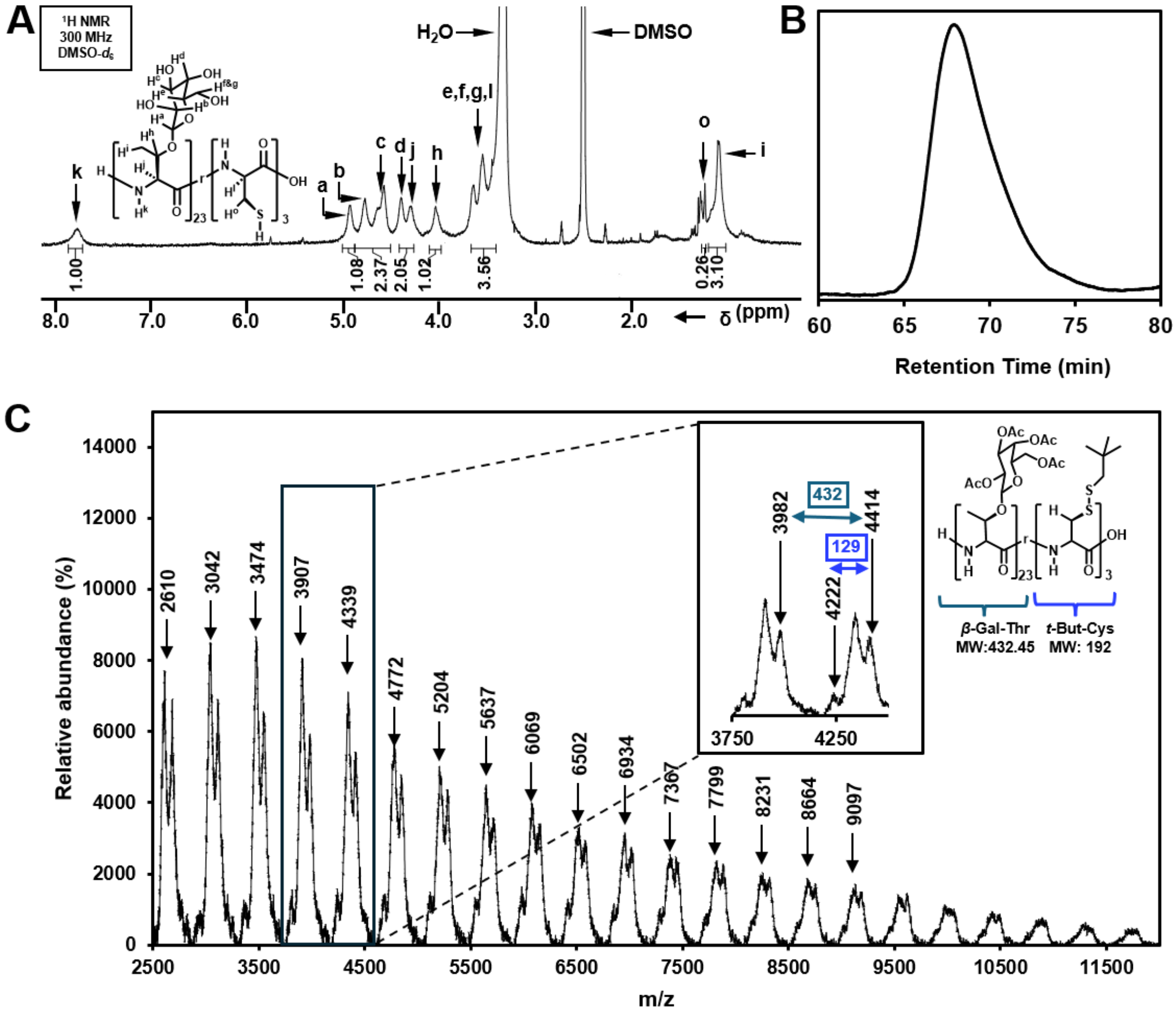

Figure S21. Characterization of poly(β-Gal-Thr)$_{23}$-*r*-poly(Cys-H)$_3$ (**Entry 21, Table S1**). (A) ^{1}H NMR poly(β-Gal-Thr)$_{23}$-*r*-poly(Cys-H)$_3$ (300 MHz, DMSO-d_6). (B) GPC of poly(β-Gal-Thr)$_{23}$-*r*-poly(Cys-H)$_3$ (DMF:DMSO) [1:1]. (C) MALDI-TOF of poly(β-AcO-Gal-Thr)$_{23}$-*r*-poly(*t*-But-Cys)$_3$.

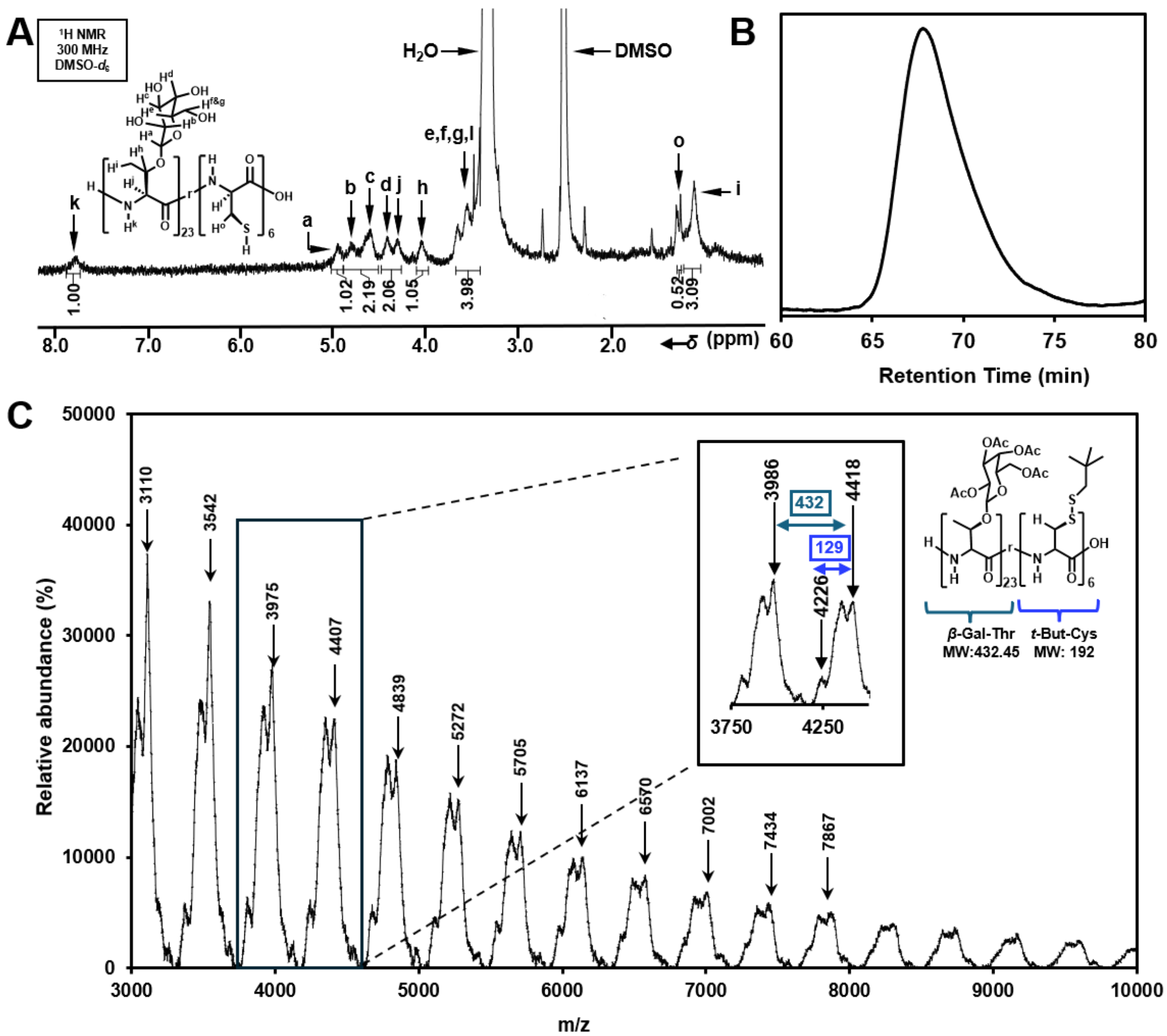

Figure S22. Characterization of poly(β-Gal-Thr)$_{23}$-*r*-poly(Cys-H)$_6$ (**Entry 22,Table S1**). (A) ^{1}H NMR poly(β-Gal-Thr)$_{23}$-*r*-poly(Cys-H)$_6$ (300 MHz, DMSO-d_6). (B) GPC of poly(β-Gal-Thr)$_{23}$-*r*-poly(Cys-H)$_6$ (DMF:DMSO) [1:1] (C) MALDI-TOF of poly(β-AcO-Gal-Thr)$_{23}$-*r*-poly(*t*-But-Cys)$_6$.

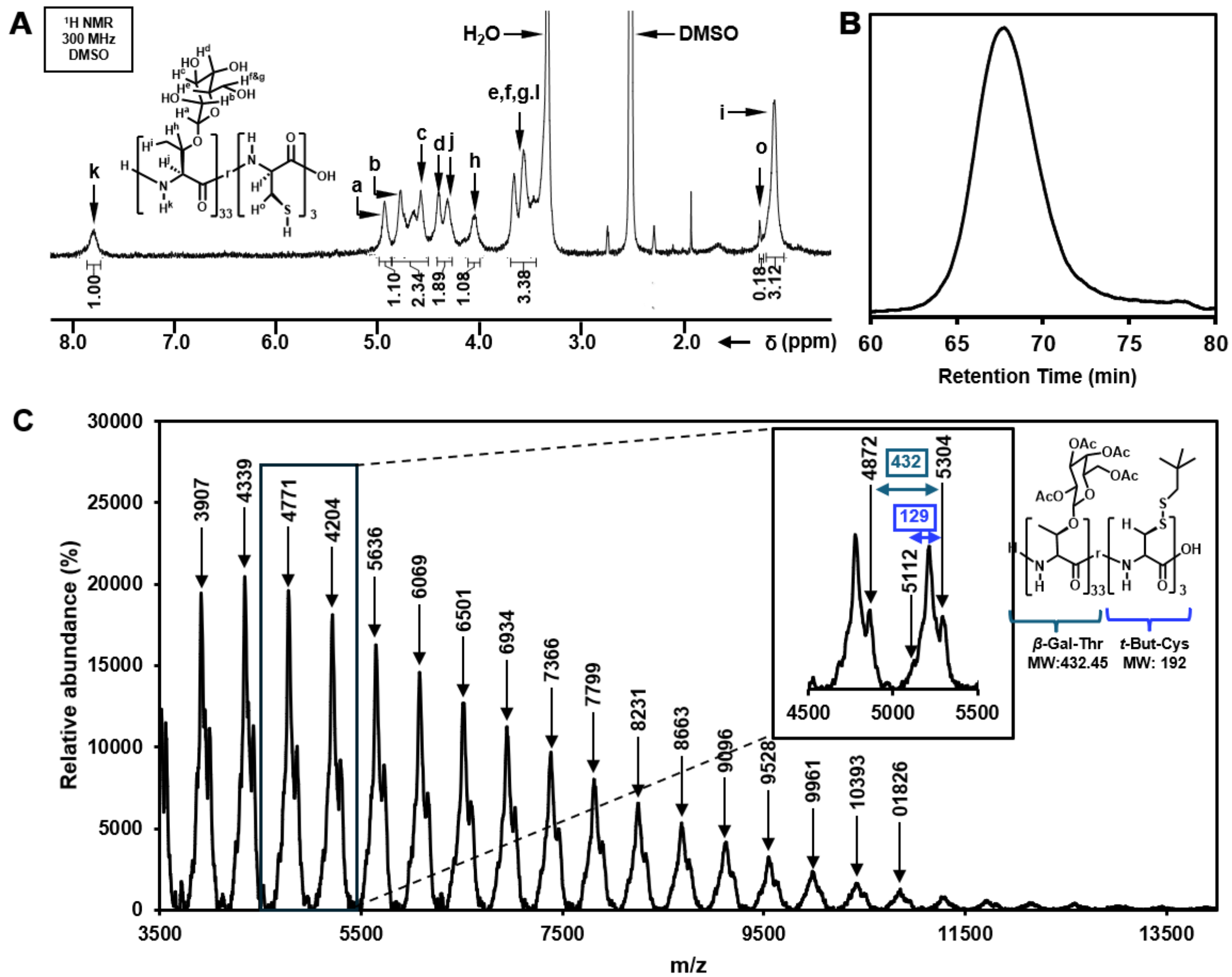

Figure S23. Characterization of poly(β-Gal-Thr)$_{33}$-*r*-poly(Cys-H)$_3$ (**Entry 23, Table S1**) (A) ^{1}H NMR poly(β-Gal-Thr)$_{33}$-*r*-poly(Cys-H)$_3$ (300 MHz, DMSO-d_6). (B) GPC of poly(β-Gal-Thr)$_{33}$-*r*-poly(Cys-H)$_3$ (DMF:DMSO) [1:1]. (C) MALDI-TOF of poly(β-AcO-Gal-Thr)$_{33}$-*r*-poly(*t*-But-Cys)$_3$.

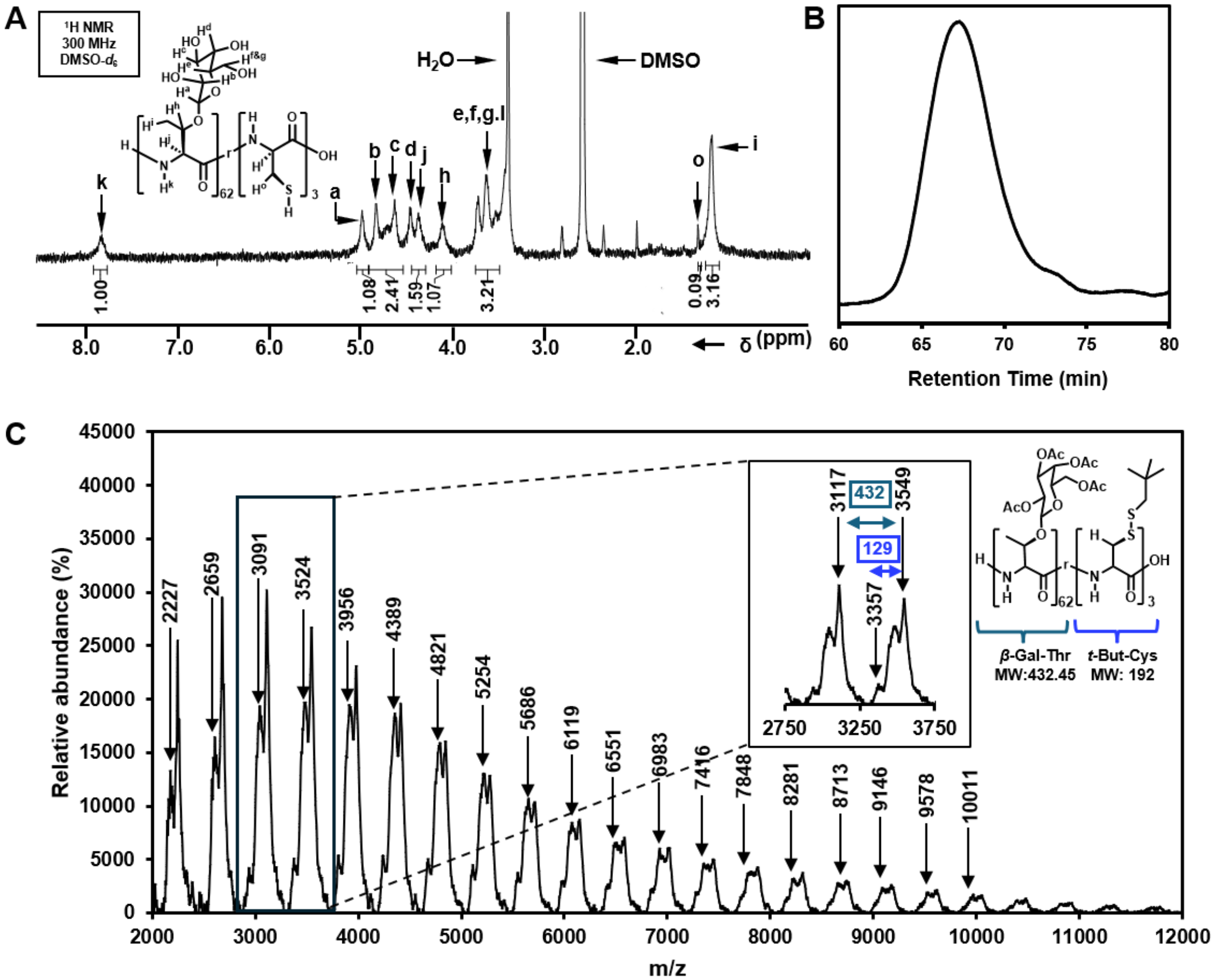

Figure S24. Characterization of poly(β-Gal-Thr)$_{62}$-*r*-poly(Cys-H)$_3$ (**Entry 24, Table S1**). (A) ^{1}H NMR poly(β-Gal-Thr)$_{62}$-*r*-poly(Cys-H)$_3$ (300 MHz, DMSO-*d*$_6$). (B) GPC of poly(β-Gal-Thr)$_{62}$-*r*-poly(Cys-H)$_3$ (DMF:DMSO) [1:1]. (C) MALDI-TOF of poly(β-AcO-Gal-Thr)$_{62}$-*r*-poly(*t*-But-Cys)$_3$.

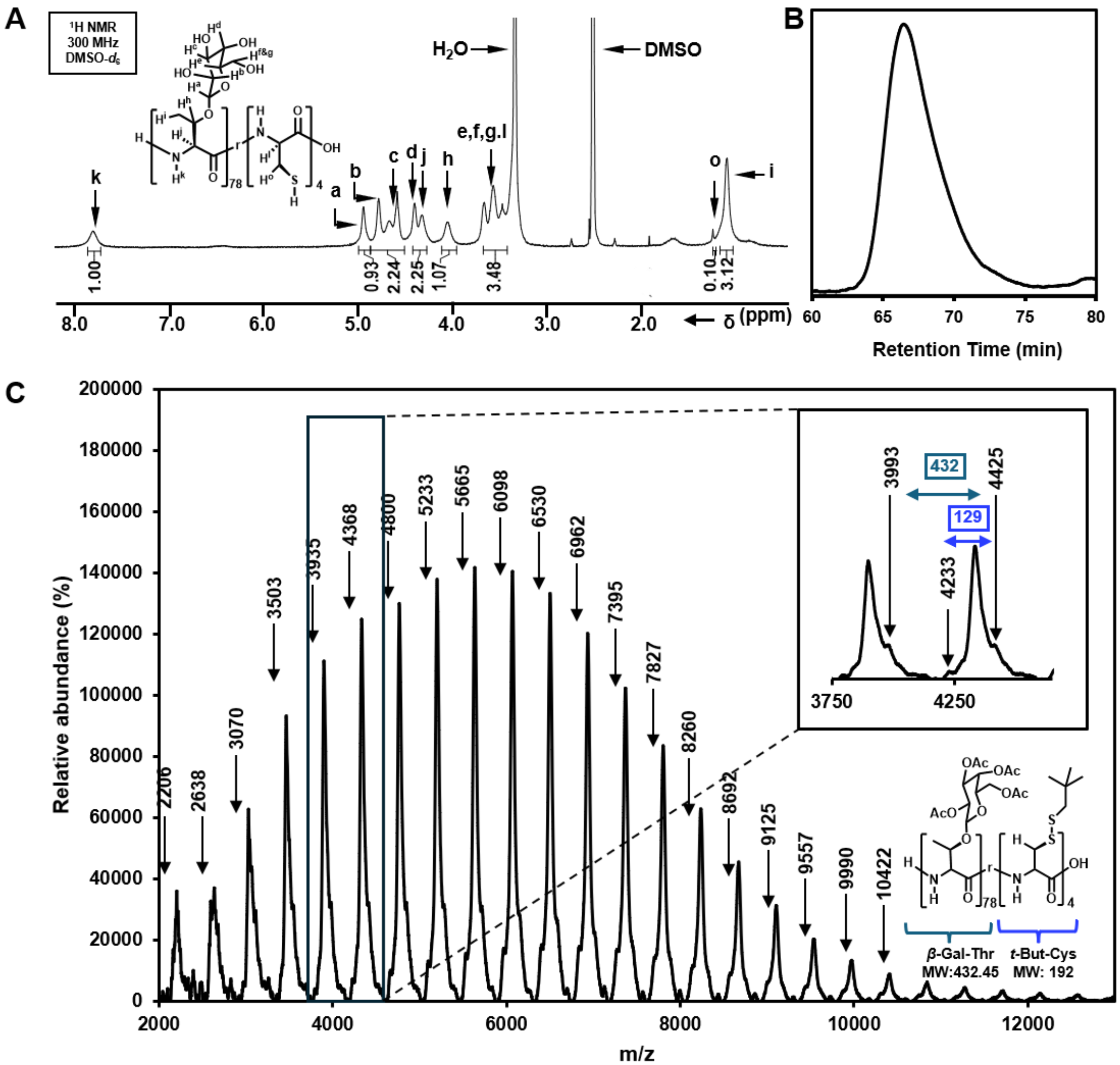

Figure S25. Characterization of poly(β-Gal-Thr)$_{78}$-*r*-poly(Cys-H)$_4$ (**Entry 25, Table S1**). (A) ^{1}H NMR poly(β-Gal-Thr)$_{78}$-*r*-poly(Cys-H)$_4$ (300 MHz, DMSO-d_6). (B) GPC of Poly(β-Gal-Thr)$_{78}$-*r*-poly(Cys-H)$_4$ (DMF:DMSO) [1:1]. **(C)** MALDI-TOF of poly(β-AcO-Gal-Thr)$_{78}$-*r*-poly(*t*-But-Cys)$_4$.

3. Determination of thiol-incorporation by Ellman's assay

Ellman's Reagent Assay for Poly(*β*-Gal-Thr)$_m$-*r*-poly(Cys-D)$_n$ and Poly(*β*-Gal-Thr)$_m$-*r*-poly(Cys-H)$_n$

General methods. Ellman's reagent (5,5′-dithio-bis-(2-nitrobenzoic acid) was purchased at Sigma. Ellman's reagent (5 mg, 0.0126 mmol) was dissolved in 10 mL of the buffer solution pH 8 (EDTA / Sodium Phosphate) to prepare a solution of (1.26 mM). 5 mg/mL (0.5%w/v) poly(*β*-Gal-Thr)$_m$-*r*-poly(Cys-H)$_n$ and poly(*β*-Gal-Thr)$_m$-*r*-poly(Cys-D)$_n$ were dissolved 1 mL of buffer solution. The Ellman's reagents were used at different concentrations for the confirmation of SH present in the polymers. Prior to analysis, the polymer solutions were sonicated until complete dissolution was achieved. The mixture (polymers and Ellman's reagents) was sonicated for 15 min, then added to a cuvette (10 mm path length), and the absorbance spectrum of the solution was recorded. Ultraviolet-Visible (UV-Vis) spectroscopy was utilized to measure absorbance of the poly(*β*-Gal-Thr)$_m$-*r*-poly(Cys-H)$_n$ and poly(*β*-Gal-Thr)$_m$-*r*-poly(Cys-D)$_n$ using a JASCO V-660 JASCO UV-Vis/NIR Spectrophotometer. To determine the absorbance of the polymer, first the buffer was prepared using ethylenediaminetetraacetic acid (EDTA) (1 mM) and sodium phosphate (Na_3PO_4) (0.1 M) to pH 8. The buffer pH was measured using a pH meter (Jenway, Model:3510) for accuracy. 5 mg of Ellman's reagent was prepared by dissolving 5,5′-dithio-bis-(2-nitrobenzoic acid in10 mL of the EDTA / sodium phosphate buffer. The Ellman's reagent solution was covered with aluminum foil and sonicated for 20 min to homogeneity. The poly(*β*-Gal-Thr)$_m$-*r*-poly(Cys-H)$_n$ and poly(*β*-Gal-Thr)$_m$-*r*-poly(Cys-D)$_n$ were prepared in 1 mL of the buffer solution (EDTA / Sodium Phosphate, pH 8) to the desired polymer concentration. The polymers were sonicated for one week for full solubility of the polymer. The mixture (polymers and Ellman's reagents) was sonicated for 15 min and added to a cuvette (10 mm pathlength), and the absorbance spectrum was then taken of these solution.

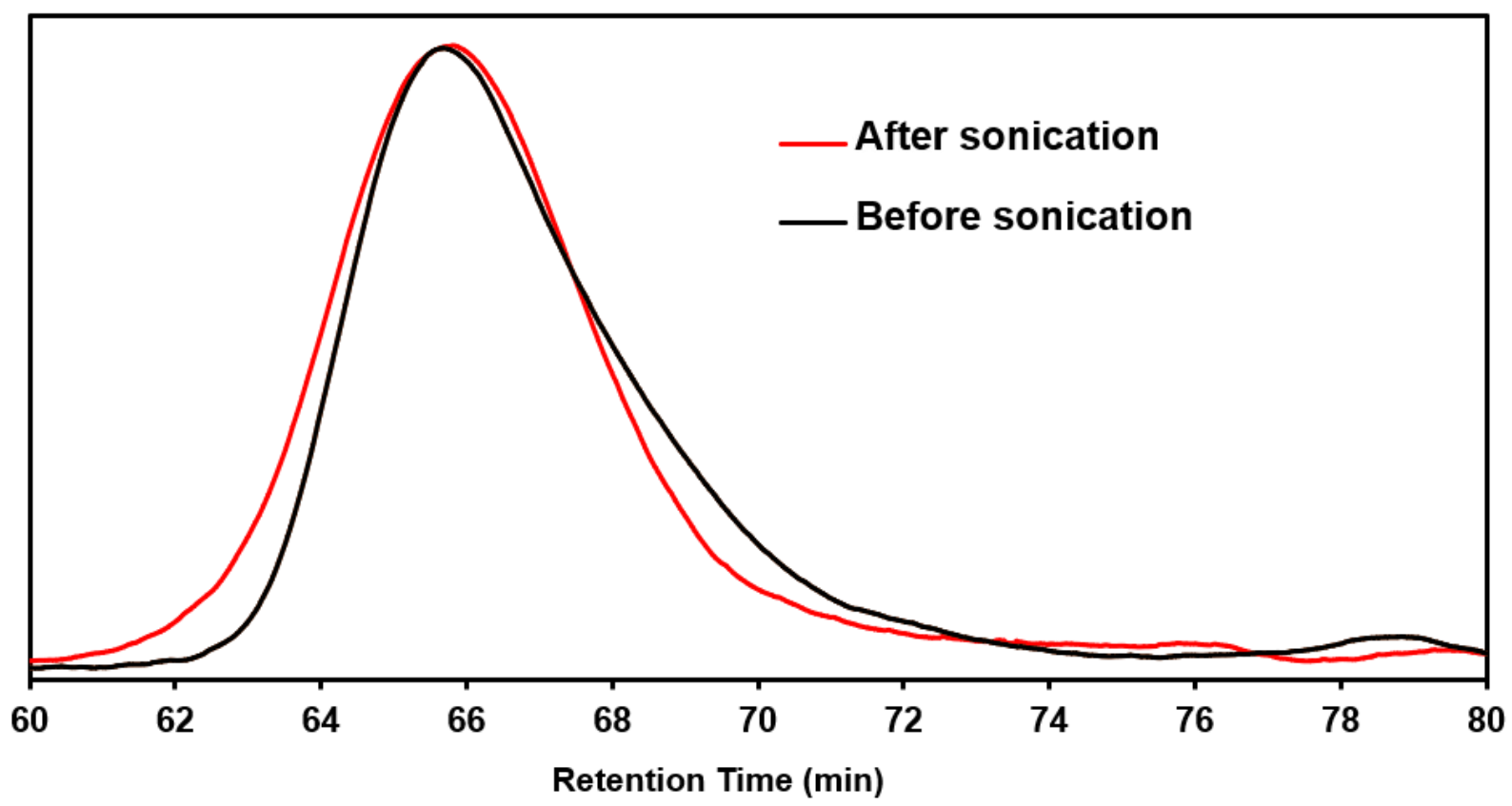

Figure S26. GPC of poly(β-Gal-Thr)$_{78}$-*r*-poly(Cys-H)$_4$ (Red line) after sonication (168 h) compared to before sonication (Black line).

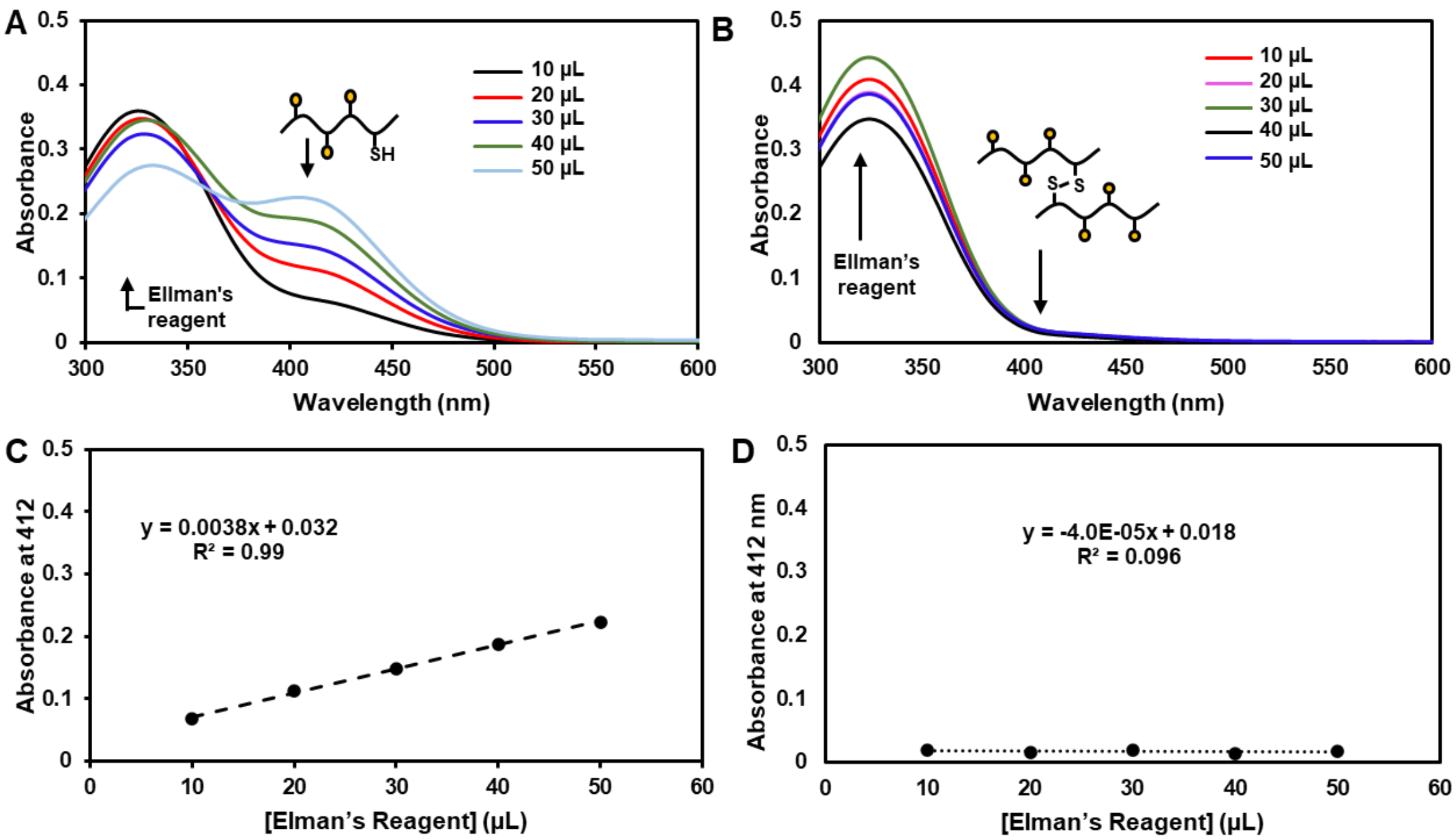

Figure S27. Ellman's assay characterization of poly(β-Gal-Thr)$_{23}$-*r*-poly(Cys-H)$_1$ and poly(β-Gal-Thr)$_{23}$-*r*-poly(Cys-D)$_1$. (A) UV-Vis characterization of poly(β-Gal-Thr)$_{23}$-*r*-poly(Cys-H)$_1$ with the titration of 50 µL of a 0.05 mM M solution of Ellman's reagent. (B) UV-Vis characterization of poly(β-Gal-Thr)$_{23}$-*r*-poly(Cys-D)$_1$ with the titration of 50 µL of a 0.05 mM solution of Ellman's reagent. (C) Absorbance vs concentration plot of poly(β-Gal-Thr)$_{23}$-*r*-poly(Cys-H)$_1$ with the titration of 50 µL of a 0.05 mM solution of Ellman's reagent. (D) Absorbance vs concentration plot poly(β-Gal-Thr)$_{23}$-*r*-poly(Cys-D)$_1$ with the titration of 50 µL of a 0.05 mM solution of Ellman's reagent.

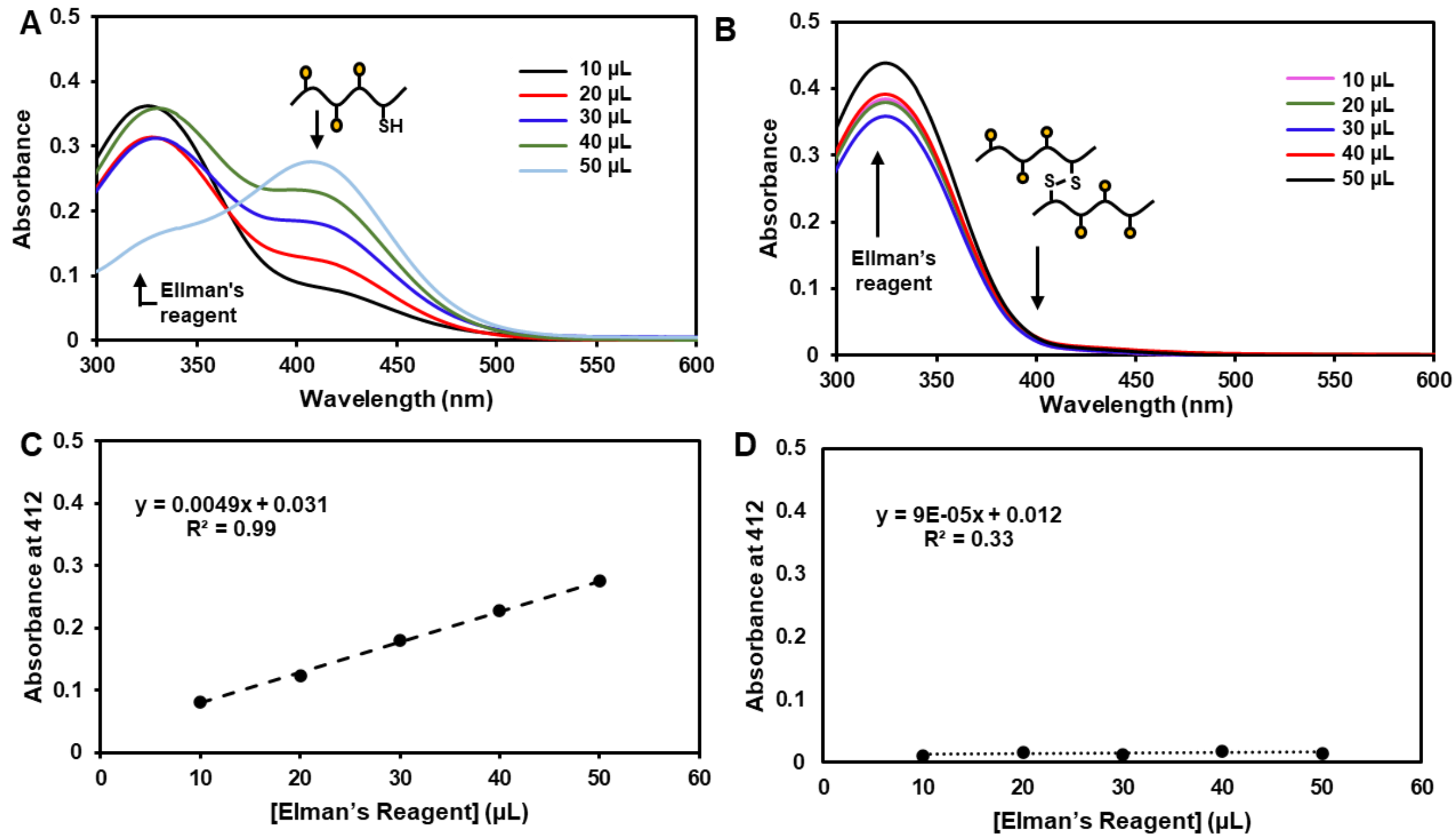

Figure S28. Ellman's assay characterization of poly(β-Gal-Thr)$_{23}$-*r*-poly(Cys-H)$_2$ and poly(β-Gal-Thr)$_{23}$-*r*-poly(Cys-D)$_2$. (A) UV-Vis characterization of poly(β-Gal-Thr)$_{23}$-*r*-poly(Cys-H)$_2$ with the titration of 50 µL of a 0.05 mM solution of Ellman's reagent. (B) UV-Vis characterization of poly(β-Gal-Thr)$_{23}$-*r*-poly(Cys-D)$_2$ with the titration of 50 µL of a 0.05 mM solution of Ellman's reagent.. (C) Absorbance vs concentration plot of poly(β-Gal-Thr)$_{23}$-*r*-poly(Cys-H)$_2$ with the titration of 50 µL of a 0.05 mM solution of Ellman's reagent. (D) Absorbance vs concentration plot poly(β-Gal-Thr)$_{23}$-*r*-poly(Cys-D)$_2$ with the titration of 50 µL of a 0.05 mM solution of Ellman's reagent.

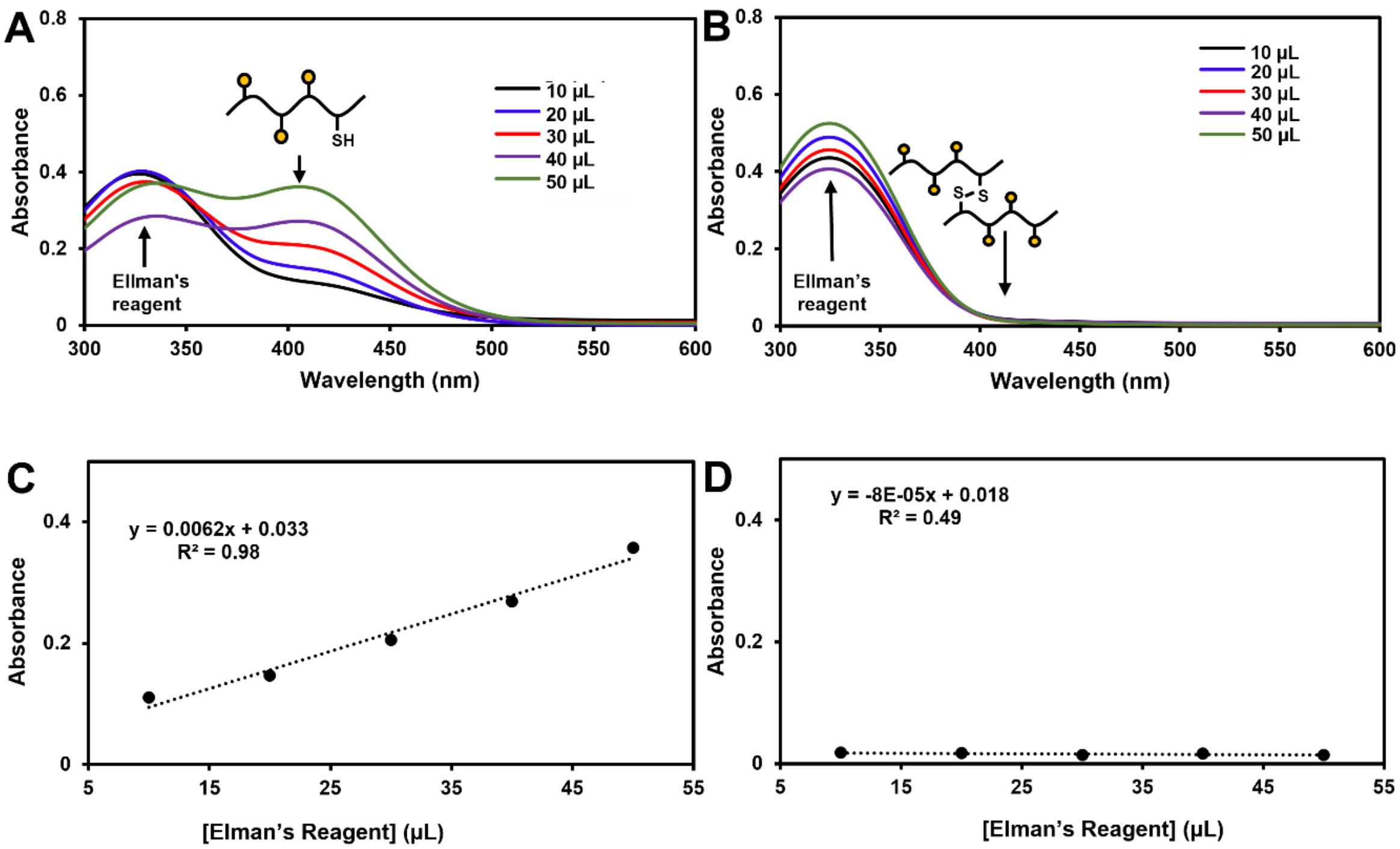

Figure S29. Ellman's assay characterization of poly(β-Gal-Thr)$_{23}$-*r*-poly(Cys-H)$_3$ and poly(β-Gal-Thr)$_{23}$-*r*-poly(Cys-D)$_3$. (A) UV-Vis characterization of poly(β-Gal-Thr)$_{23}$-*r*-poly(Cys-H)$_3$ with the titration of 50 µL of a 0.05 mM solution of Ellman's reagent. (B) UV-Vis characterization of poly(β-Gal-Thr)$_{23}$-*r*-poly(Cys-D)$_3$ with the titration of 50 µL of a 0.05 mM solution of Ellman's reagent.. (C) Absorbance vs concentration plot of poly(β-Gal-Thr)$_{23}$-*r*-poly(Cys-H)$_1$ with the titration of 50 µL of a 0.05 mM solution of Ellman's reagent. (D) Absorbance vs concentration plot poly(β-Gal-Thr)$_{23}$-*r*-poly(Cys-D)$_3$ with the titration of 50 µL of a 0.05 mM solution of Ellman's reagent.

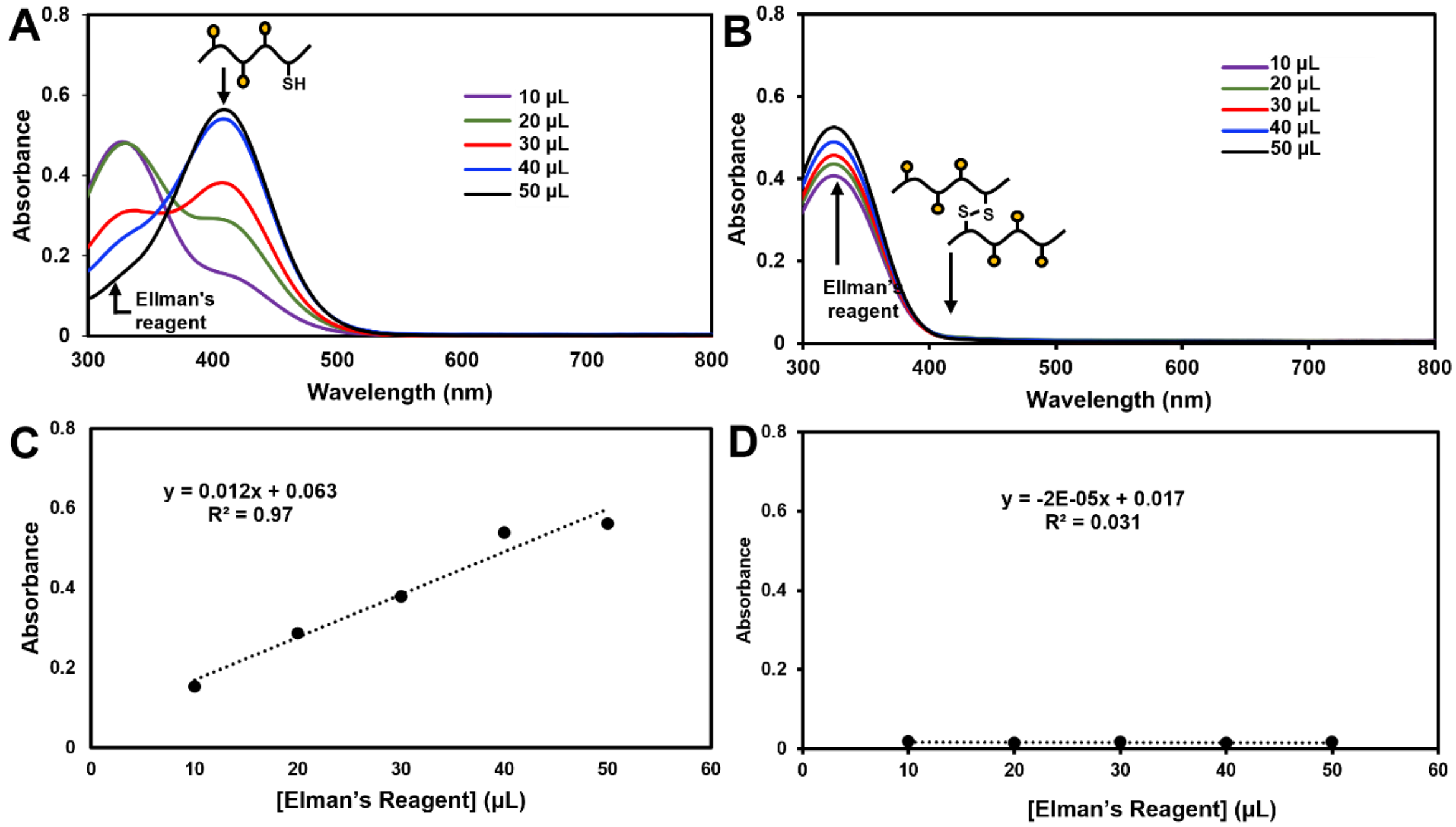

Figure S30. Ellman's assay characterization of poly(β-Gal-Thr)$_{23}$-*r*-poly(Cys-H)$_6$ and poly(β-Gal-Thr)$_{23}$-*r*-poly(Cys-D)$_6$. (A) UV-Vis characterization of poly(β-Gal-Thr)$_{23}$-*r*-poly(Cys-H)$_6$ with the titration of 50 µL of a 0.05 mM solution of Ellman's reagent. (B) UV-Vis characterization of poly(β-Gal-Thr)$_{23}$-*r*-poly(Cys-D)$_6$ with the titration of 50 µL of a 0.05 mM solution of Ellman's reagent.. (C) Absorbance vs concentration plot of poly(β-Gal-Thr)$_{23}$-*r*-poly(Cys-H)$_6$ with the titration of 50 µL of a 0.05 mM solution of Ellman's reagent. (D) Absorbance vs concentration plot poly(β-Gal-Thr)$_{23}$-*r*-poly(Cys-D)$_6$ with the titration of 50 µL of a 0.05 mM solution of Ellman's reagent.

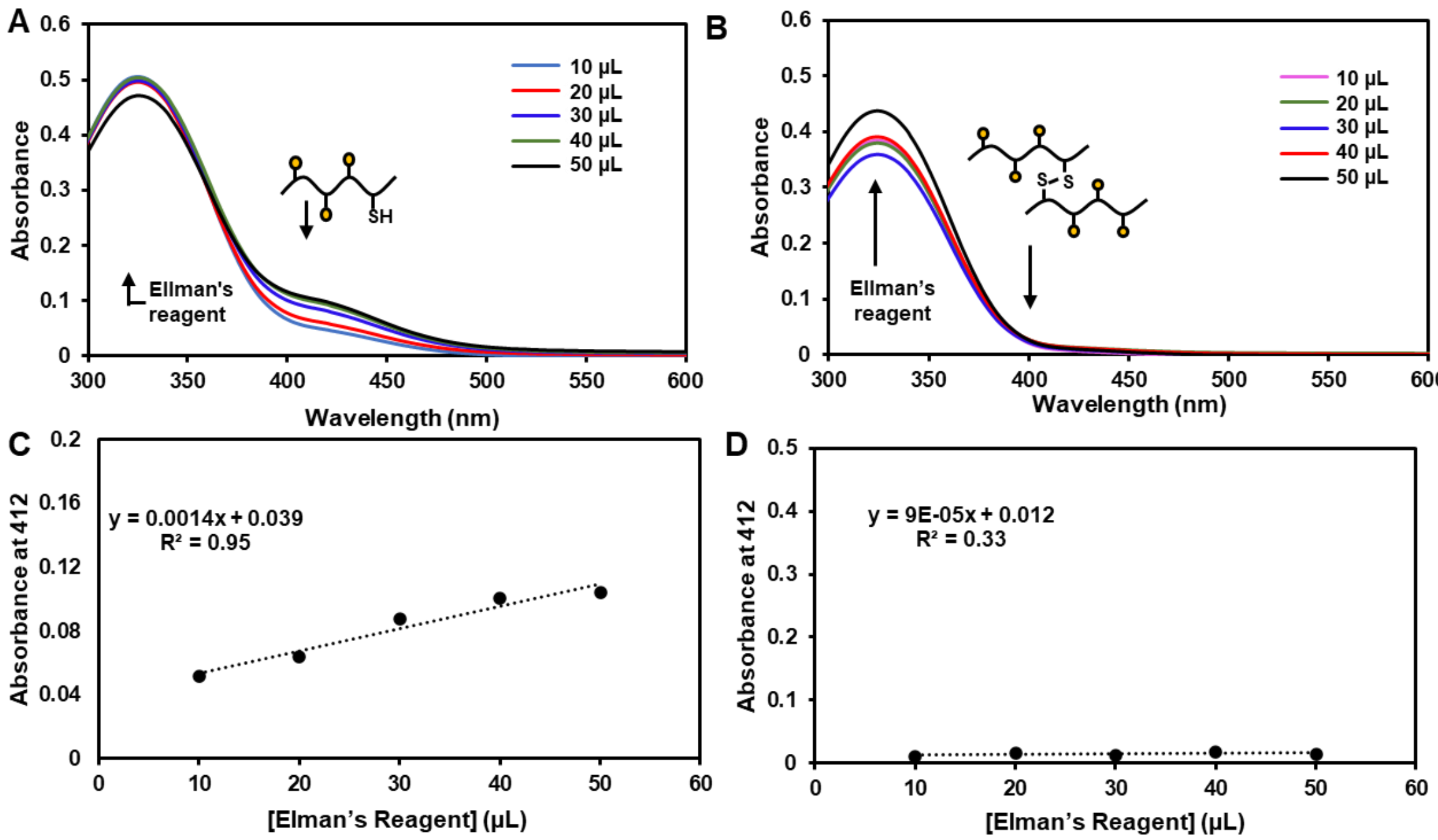

Figure S31. Ellman's assay characterization of poly(β-Gal-Thr)$_{33}$-*r*-poly(Cys-H)$_3$ and poly(β-Gal-Thr)$_{33}$-*r*-poly(Cys-D)$_3$. (A) UV-Vis characterization of poly(β-Gal-Thr)$_{33}$-*r*-poly(Cys-H)$_3$ with the titration of 50 µL of a 0.05 mM solution of Ellman's reagent. (B) UV-Vis characterization of poly(β-Gal-Thr)$_{33}$-*r*-poly(Cys-D)$_3$ with the titration of 50 µL of a 0.05 mM solution of Ellman's reagent. (C) Absorbance vs concentration plot of poly(β-Gal-Thr)$_{33}$-*r*-poly(Cys-H)$_3$ with the titration of 50 µL of a 0.05 mM solution of Ellman's reagent. (D) Absorbance vs concentration plot poly(β-Gal-Thr)$_{33}$-*r*-poly(Cys-D)$_3$ with the titration of 50 µL of a 0.05 mM solution of Ellman's reagent.

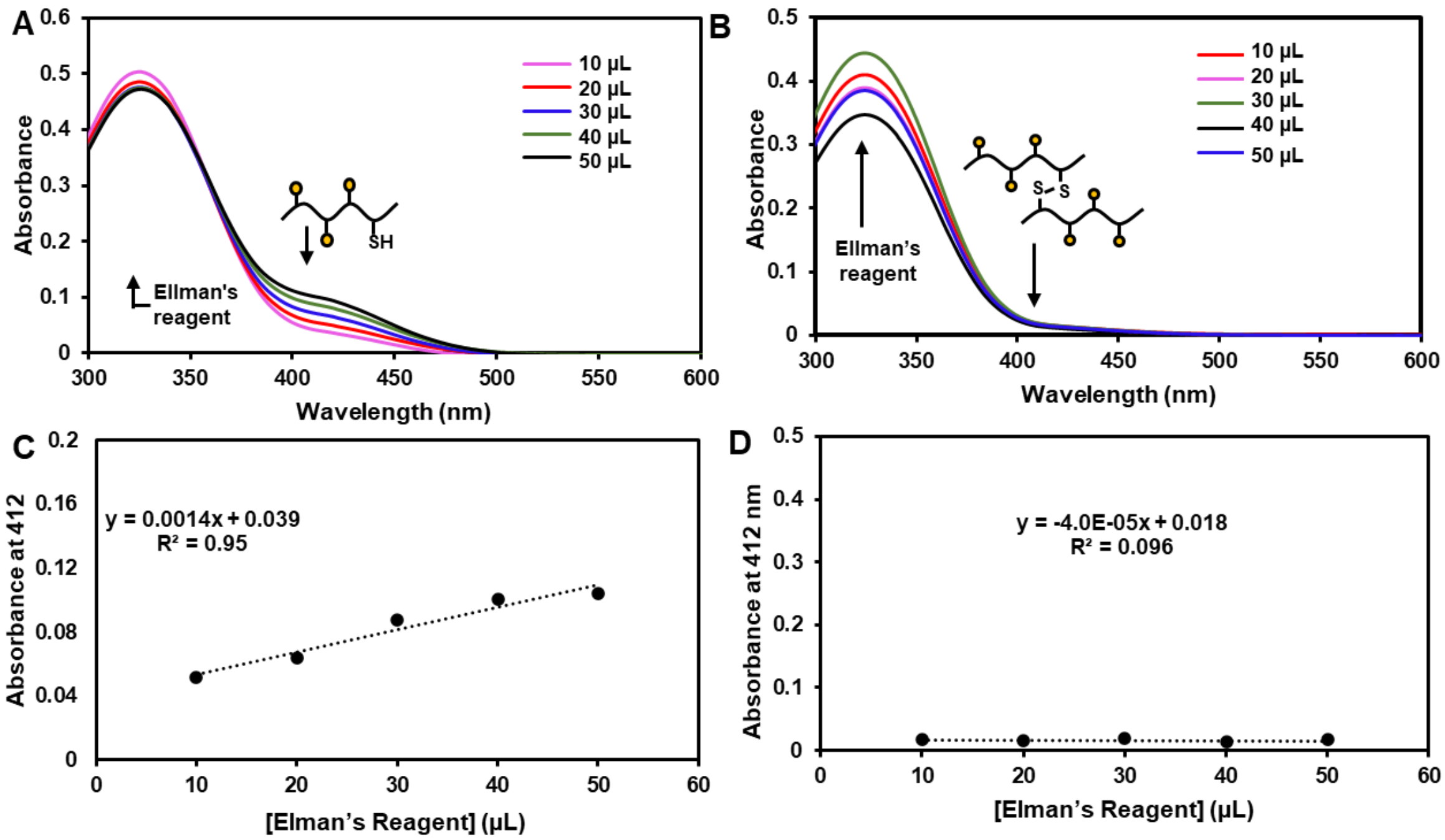

Figure S32. Ellman's assay characterization of poly(β-Gal-Thr)$_{62}$-*r*-poly(Cys-H)$_3$ and poly(β-Gal-Thr)$_{62}$-*r*-poly(Cys-D)$_3$. (A) UV-Vis characterization of poly(β-Gal-Thr)$_{62}$-*r*-poly(Cys-H)$_3$ with the titration of 50 µL of a 0.05 mM solution of Ellman's reagent. (B) UV-Vis characterization of poly(β-Gal-Thr)$_{62}$-*r*-poly(Cys-D)$_3$ with the titration of 50 µL of a 0.05 mM solution of Ellman's reagent. (C) Absorbance vs concentration plot of poly(β-Gal-Thr)$_{62}$-*r*-poly(Cys-H)$_3$ with the titration of 50 µL of a 0.05 mM solution of Ellman's reagent. (D) Absorbance vs concentration plot poly(β-Gal-Thr)$_{62}$-*r*-poly(Cys-D)$_3$ with the titration of 50 µL of a 0.05 mM solution of Ellman's reagent.

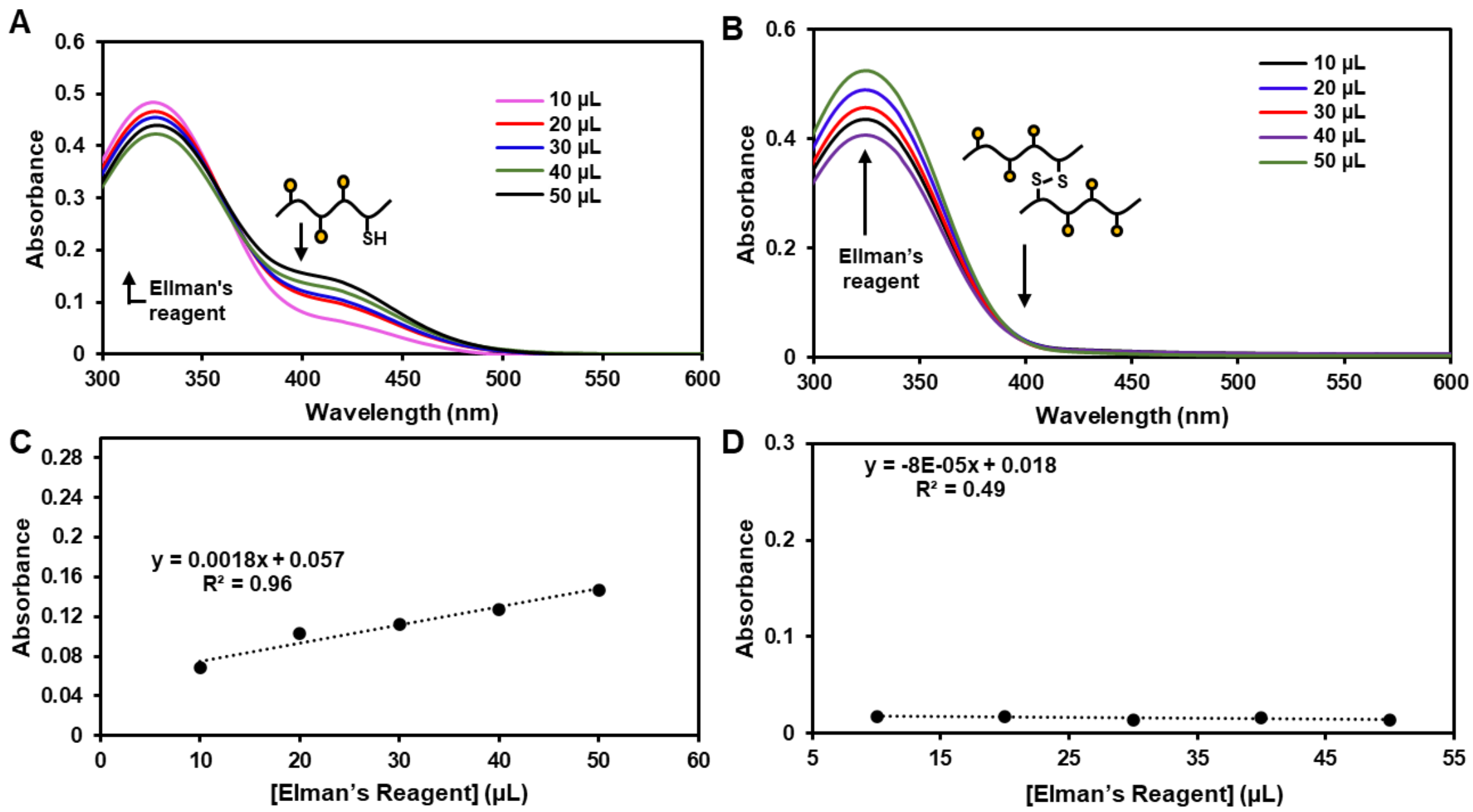

Figure S33. Ellman's assay characterization of poly(β-Gal-Thr)$_{78}$-*r*-poly(Cys-H)$_4$ and poly(β-Gal-Thr)$_{78}$-*r*-poly(Cys-D)$_4$. (A) UV-Vis characterization of poly(β-Gal-Thr)$_{78}$-*r*-poly(Cys-H)$_4$ with the titration of 50 µL of a 0.05 mM solution of Ellman's reagent. (B) UV-Vis characterization of poly(β-Gal-Thr)$_{78}$-*r*-poly(Cys-D)$_4$ with the titration of 50 µL of a 0.05 mM solution of Ellman's reagent.. (C) Absorbance vs concentration plot of poly(β-Gal-Thr)$_{78}$-*r*-poly(Cys-H)$_4$ with the titration of 50 µL of a 0.05 mM solution of Ellman's reagent. (D) Absorbance vs concentration plot poly(β-Gal-Thr)$_{78}$-*r*-poly(Cys-D)$_4$ with the titration of 50 µL of a 0.05 mM solution of Ellman's reagent.

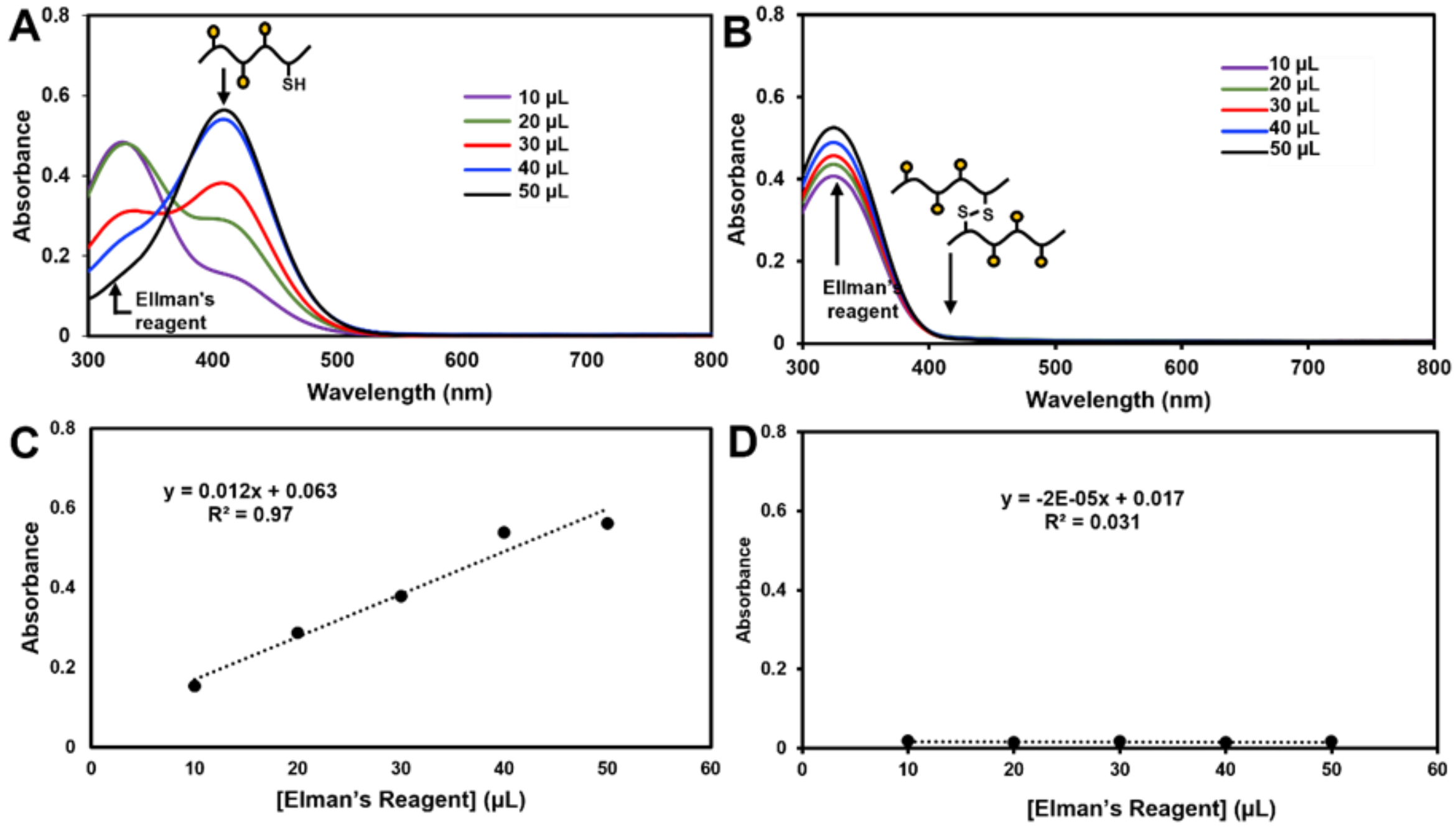

Figure S34. Ellman's reagent reversibility characterization of poly(β-Gal-Thr)$_{23}$-*r*-poly(Cys-H)$_1$ and poly(β-Gal-Thr)$_{23}$-*r*-poly(Cys-D)$_1$. (A) UV-Vis characterization of poly(β-Gal-Thr)$_{23}$-*r*-poly(Cys-H)$_1$ with the titration of 50 µL of a 0.05 mM solution of Ellman's reagent. (B) UV-Vis characterization of poly(β-Gal-Thr)$_{23}$-*r*-poly(Cys-D)$_1$ with the titration of 50 µL of a 0.05 mM solution of Ellman's reagent. (C) Absorbance vs concentration plot of poly(β-Gal-Thr)$_{23}$-*r*-poly(Cys-H)$_1$ with the titration of 50 µL of a 0.05 mM solution of Ellman's reagent. (D) Absorbance vs concentration plot poly(β-Gal-Thr)$_{23}$-*r*-poly(Cys-D)$_1$ with the titration of 50 µL of a 0.05 mM solution of Ellman's reagent.

4. Scanning Electron Microscopy and Energy-Dispersive X-ray (EDX) Analysis

General methods. The poly(β-Gal-Thr)$_m$-*r*-poly(Cys-H)$_n$ and poly(β-Gal-Thr)$_m$-*r*-poly(Cys-D)$_n$ were prepared by freezing in liquid nitrogen followed by lyophilization prior to imaging. Consequently, the observed morphologies are representative of the polymers' dried state and may not fully reflect the native hydrated network structure. Then the polymers were placed on a double-sided tape to keep the polymers set in a specific place and mounted to a double on conductive aluminum stubs (Product No. 16120, Ted Pella, Inc.) and sputter-coated with 5 nm layer of gold using a Leica EM ACE600. Imaging was performed on a FEI Helios Nanolab 660 focused ion beam–scanning electron microscope (FIB-SEM) with HT of 5 kV, current of 6.3, 13 and 25 pA with ETD (Everhart-Thornley) detector. EDX (energy-dispersive X-ray spectroscopy) mapping was collected with a Silicon Drift Detectors (SDD) at HT of 10 kV and current of 1.6 nA. Data was collected and analyzed using AZtec v6.0 software.[6]

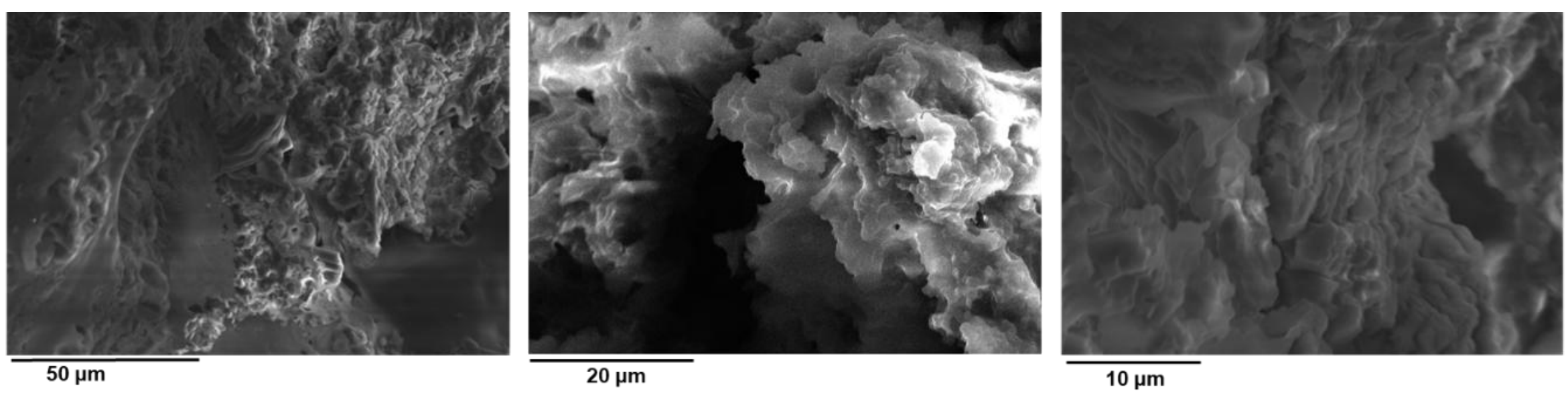

Figure S35. SEM image of poly(β-Gal-Thr)$_{23}$. From left to right Magnification 500X, 2000X, 3500X.

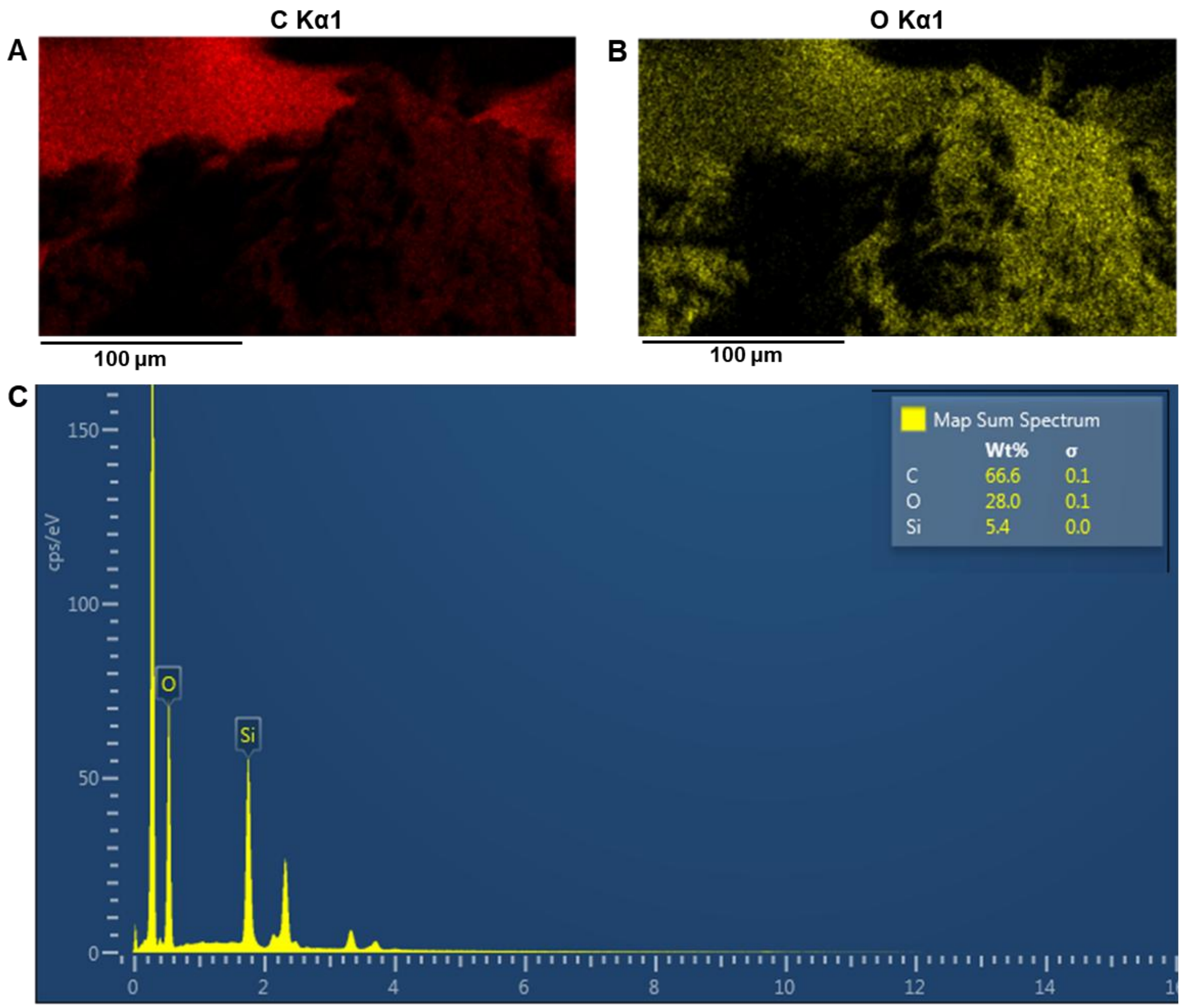

Figure S36. EDX analysis of poly(*β*-Gal-Thr)$_{23}$. (A) Localization of detected Carbon. Scale bar shown. (B) Localization of detected Oxygen. Scale bar shown. (C) EDX Spectra showing elemental composition of poly(*β*-Gal-Thr)$_{23}$.

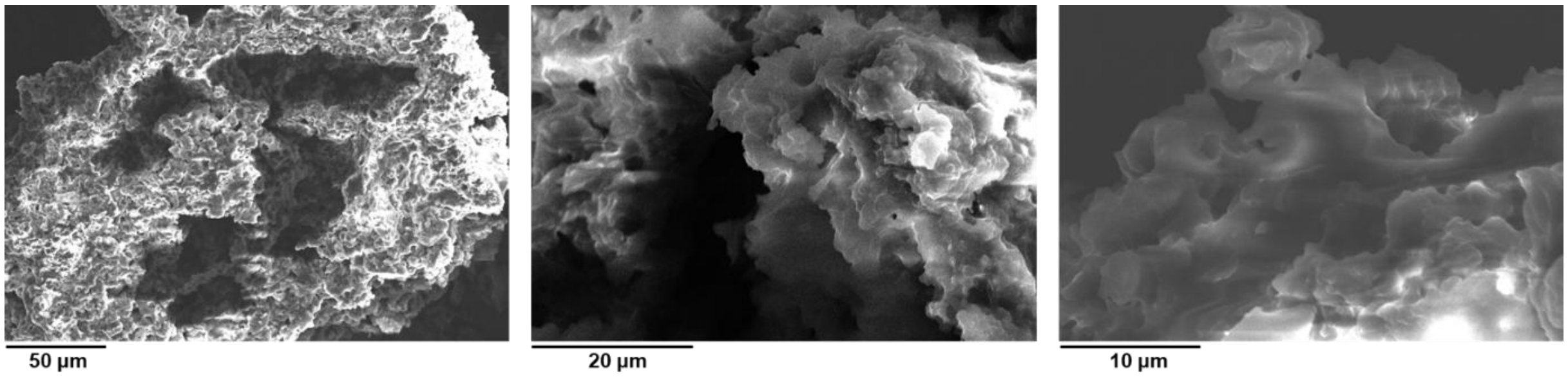

Figure S37. SEM image of poly(β-Gal-Thr)$_{23}$-*r*-poly(Cys-H)$_1$. From left to right Magnification 500X, 2000X, 3500X.

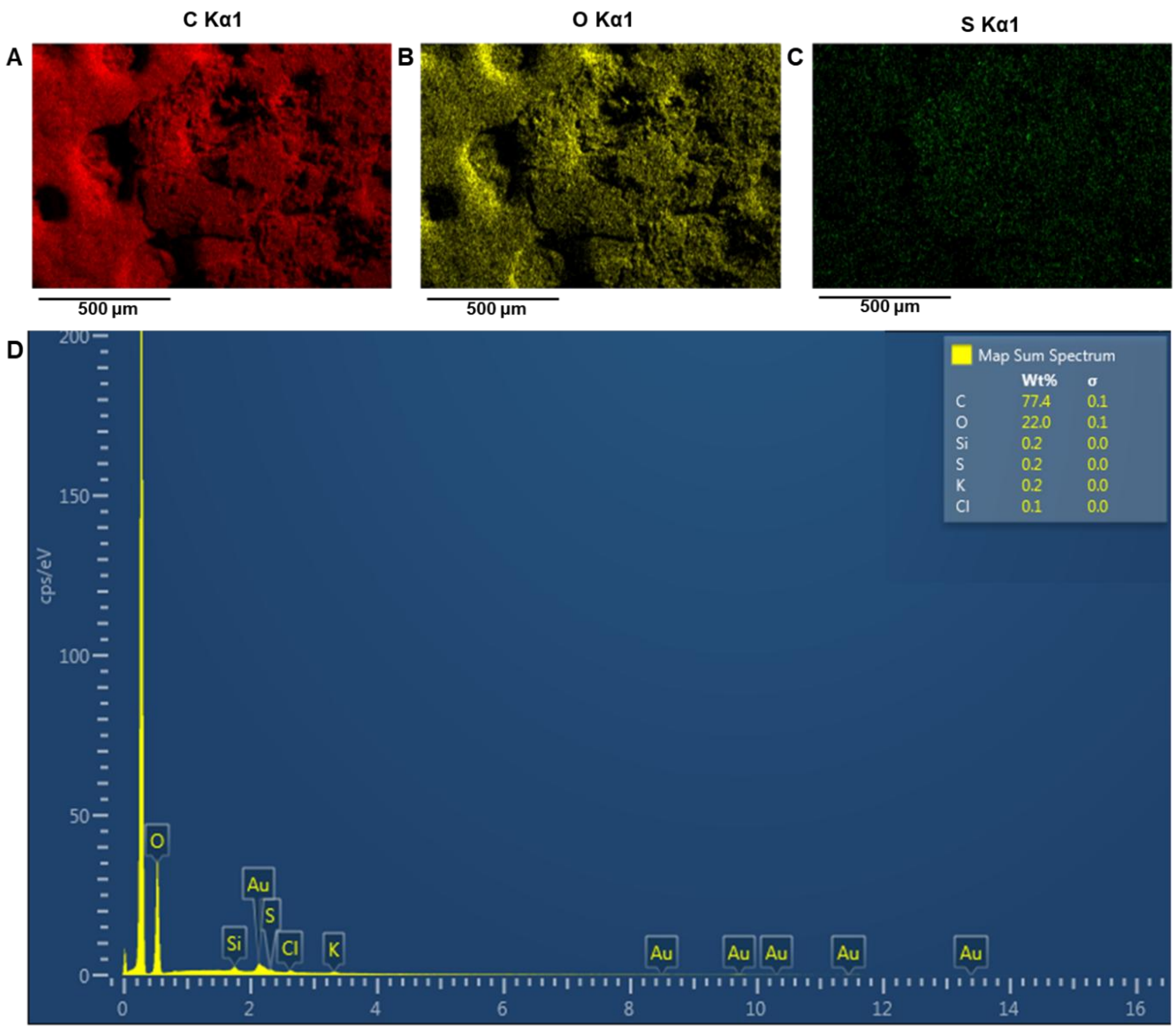

Figure S38. EDX analysis of poly(β-Gal-Thr)$_{23}$-*r*-poly(Cys-H)$_1$. (A) Localization of detected Carbon. Scale bar shown. (B) Localization of detected Oxygen. Scale bar shown. (C) Localization of detected Sulfur. Scale bar shown. (D) EDX Spectra showing elemental composition of poly(β-Gal-Thr)$_{23}$-*r*-poly(Cys-H)$_1$.

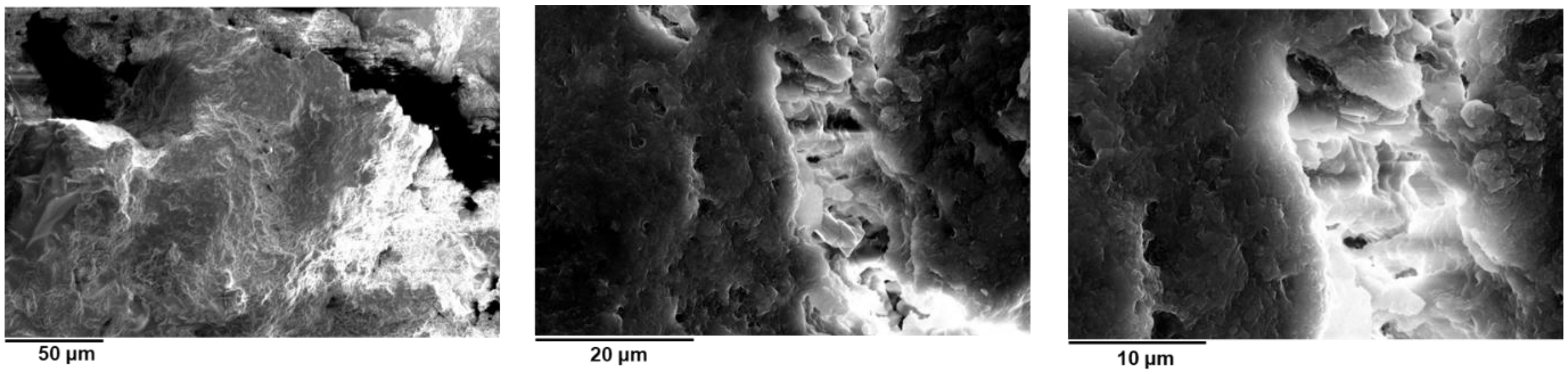

Figure S39. SEM image of poly(β-Gal-Thr)$_{23}$-r-poly(Cys-D)$_1$. From left to right Magnification 500X, 2000X, 3500X.

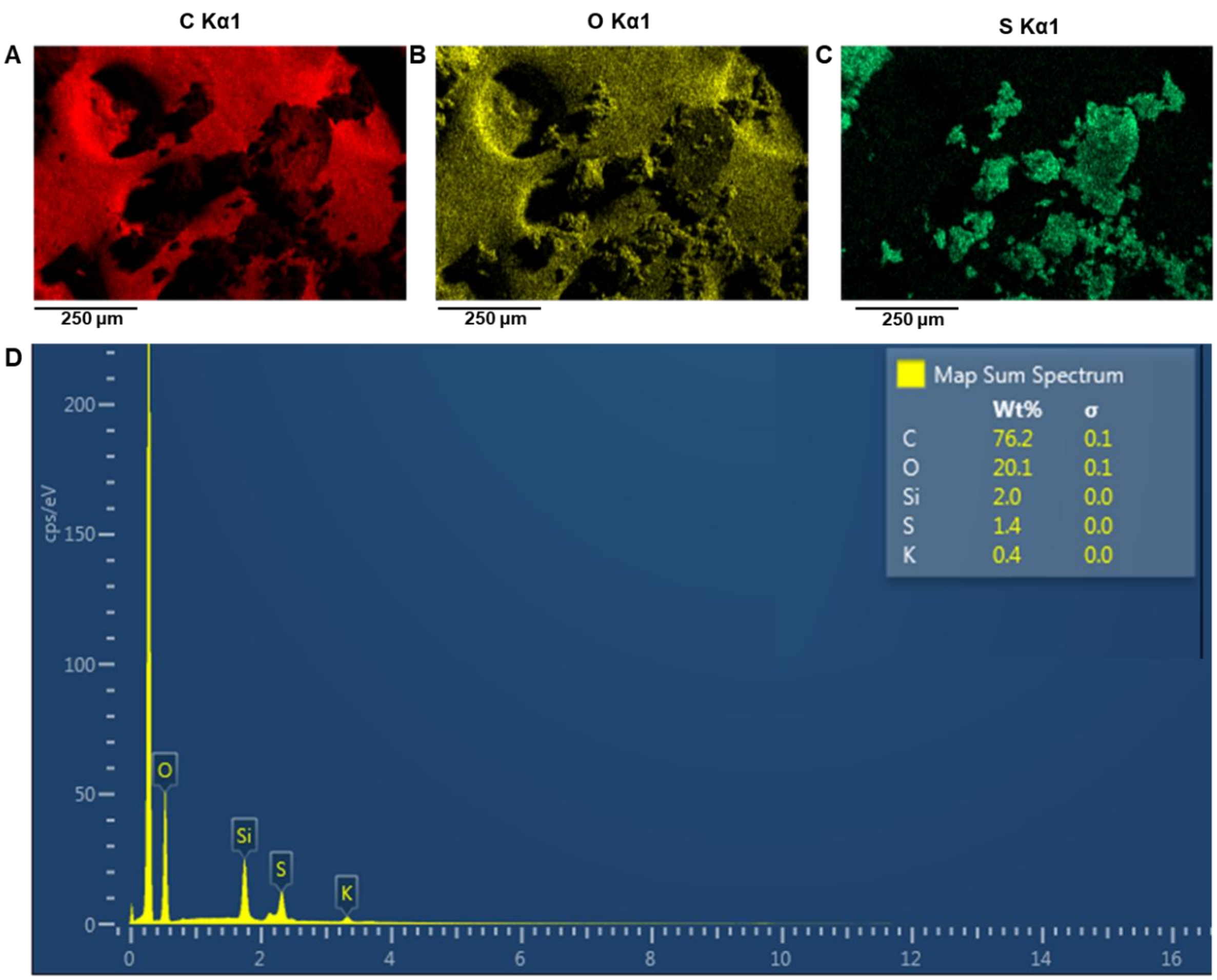

Figure S40. EDX analysis of poly(β-Gal-Thr)$_{23}$-r-poly(Cys-D)$_1$. (A) Localization of detected Carbon. Scale bar shown. (B) Localization of detected Oxygen. Scale bar shown. (C) Localization of detected Sulfur. Scale bar shown. (D) EDX Spectra showing elemental composition of poly(β-Gal-Thr)$_{23}$-r-poly(Cys-D)$_1$.

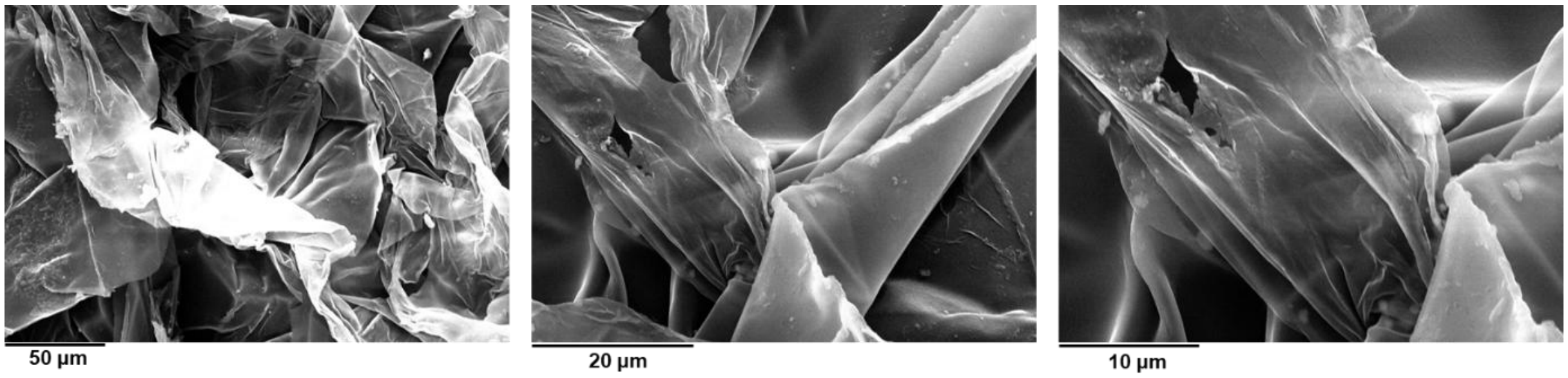

Figure S41. SEM image of poly(β-Gal-Thr)$_{23}$-*r*-poly(Cys-H)$_2$. From left to right Magnification 500X, 2000X, 3500X.

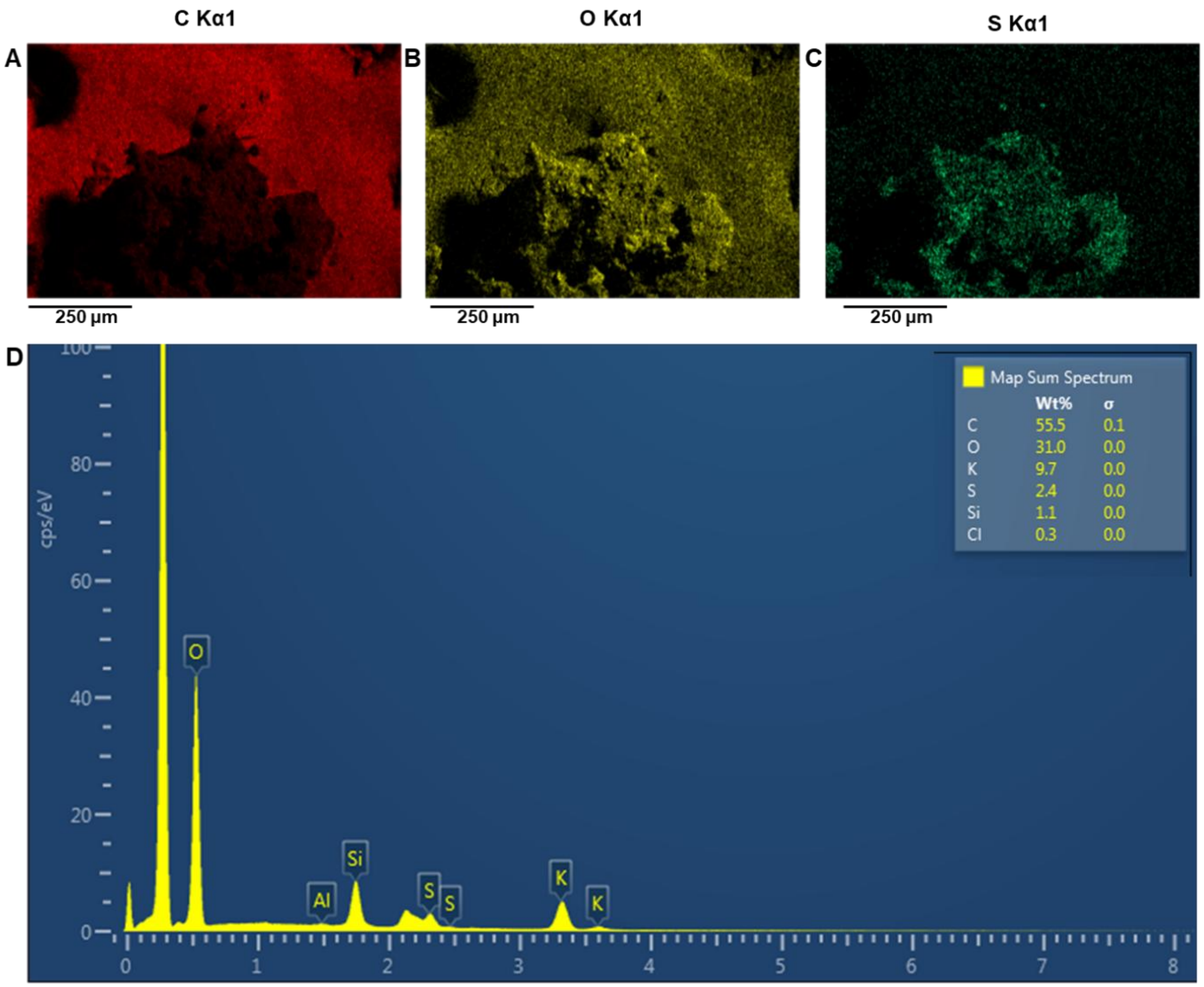

Figure S42. EDX of poly(β-Gal-Thr)$_{23}$-*r*-poly(Cys-H)$_2$. (A) Localization of detected Carbon. Scale bar shown. (B) Localization of detected Oxygen. Scale bar shown. (C) Localization of detected Sulfur. Scale bar shown. (D) EDX Spectra showing elemental composition of poly(β-Gal-Thr)$_{23}$-*r*-poly(Cys-H)$_2$.

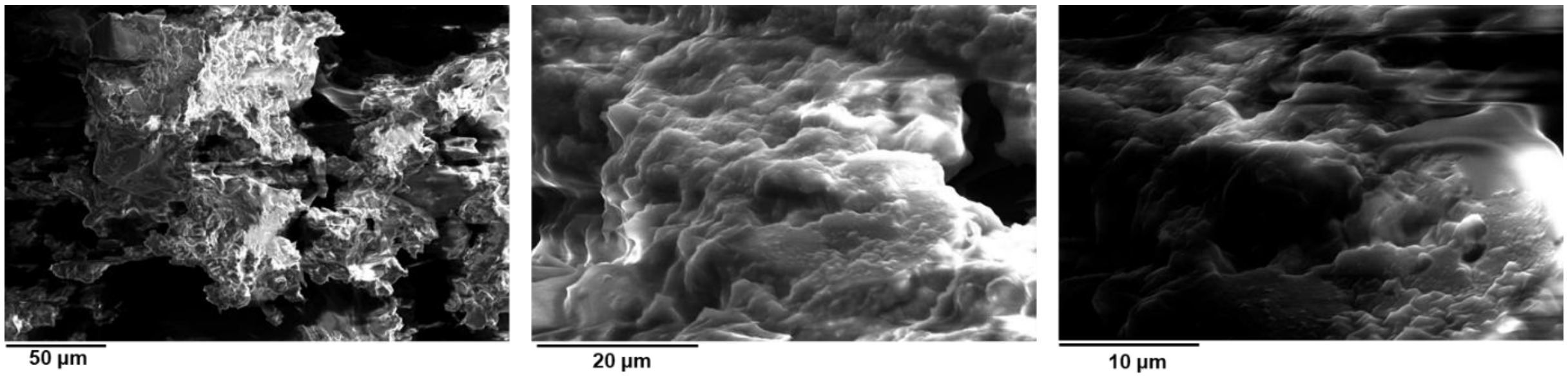

Figure S43. SEM image of poly(β-Gal-Thr)$_{23}$-*r*-poly(Cys-D)$_2$. From left to right Magnification 500X, 2000X, 3500X.

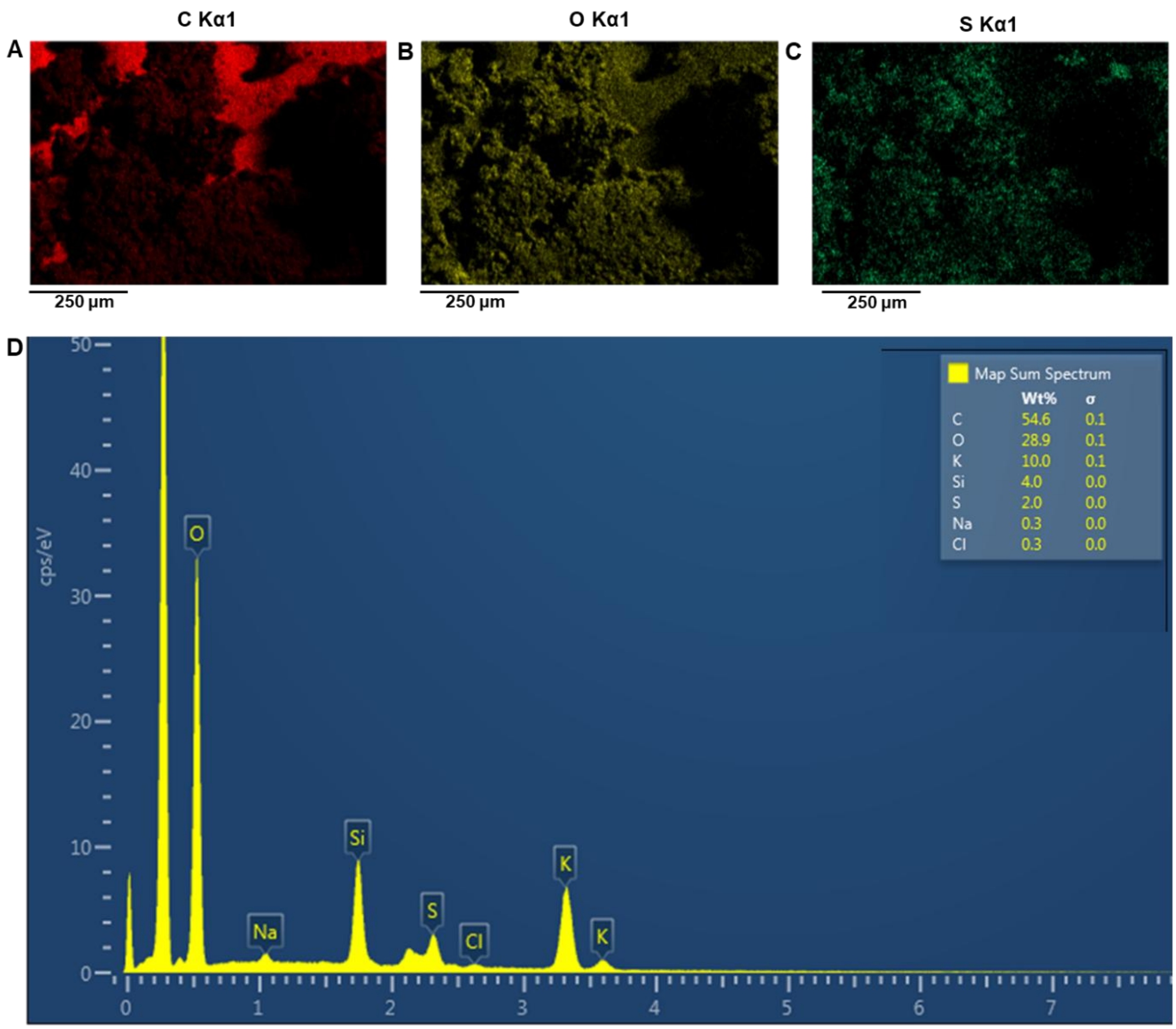

Figure S44. EDX analysis of poly(β-Gal-Thr)$_{23}$-*r*-poly(Cys-D)$_2$. (A) Localization of detected Carbon. Scale bar shown. (B) Localization of detected Oxygen. Scale bar shown. (C) Localization of detected Sulfur. Scale bar shown. (D) EDX Spectra showing elemental composition of poly(β-Gal-Thr)$_{23}$-*r*-poly(Cys-D)$_2$.

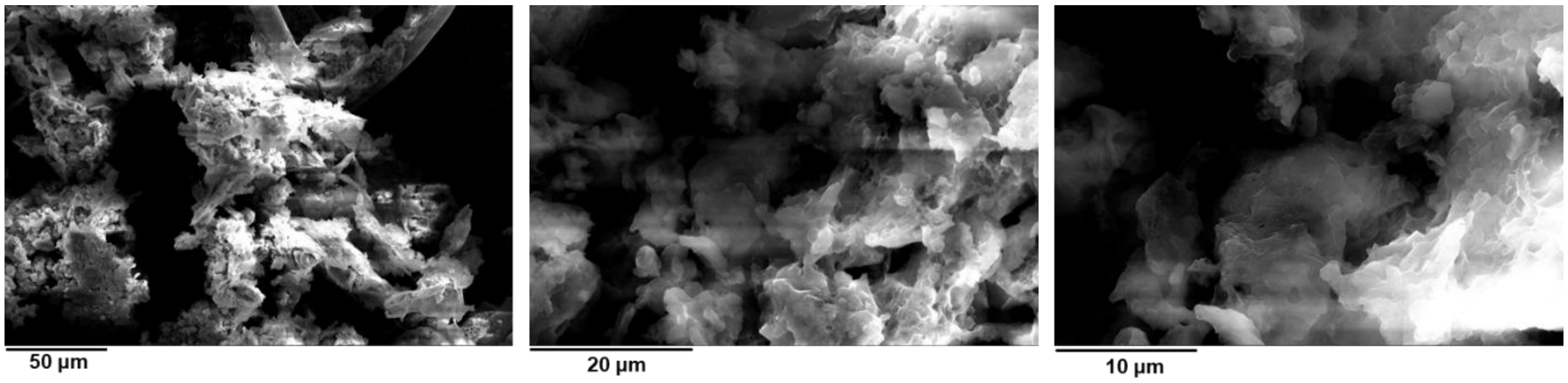

Figure S45. SEM image of poly(β-Gal-Thr)$_{23}$-*r*-poly(Cys-H)$_3$. From left to right Magnification 500X, 2000X, 3500X.

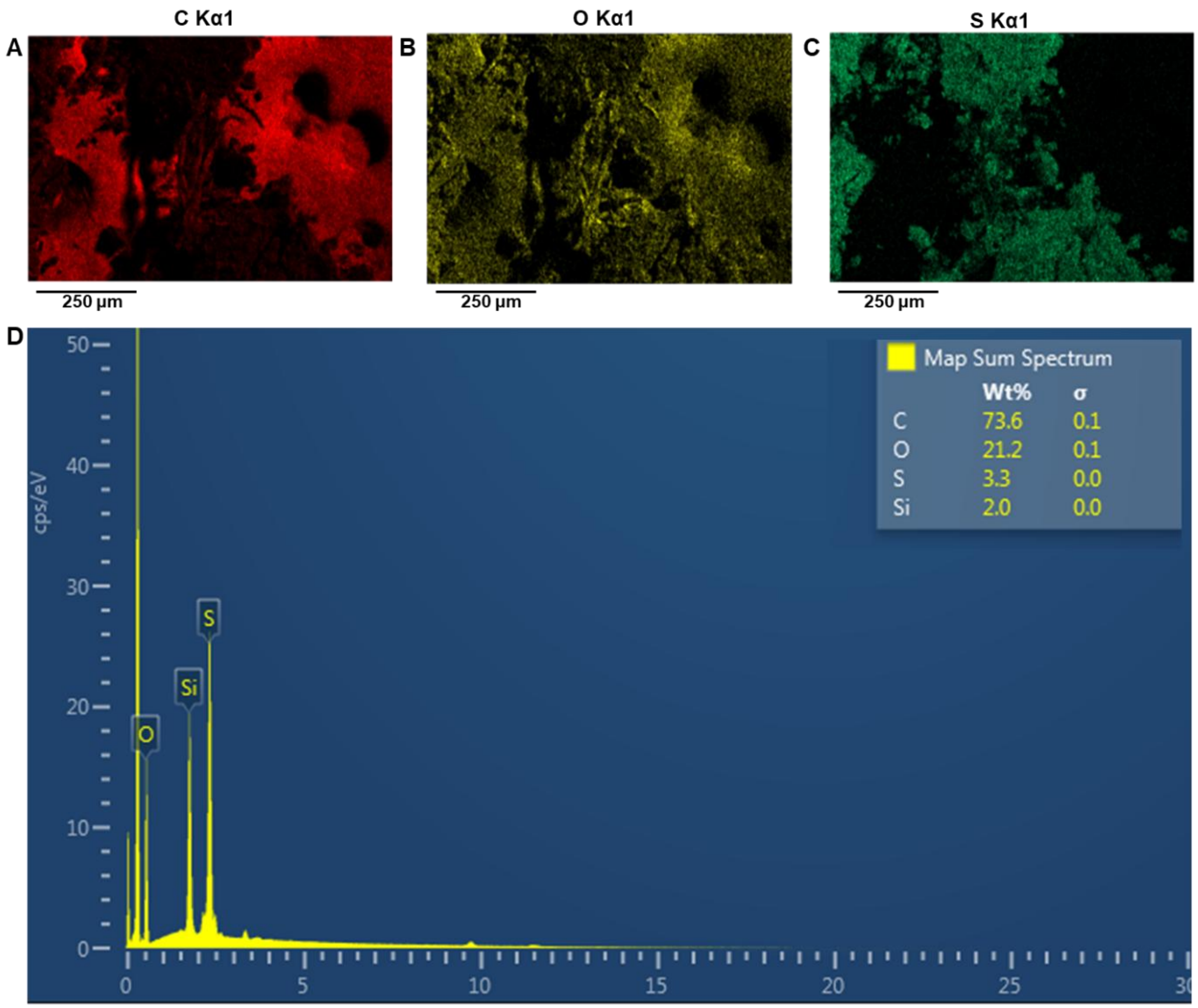

Figure S46. EDX analysis of poly(β-Gal-Thr)$_{23}$-*r*-poly(Cys-H)$_3$. (A) Localization of detected Carbon. Scale bar shown. (B) Localization of detected Oxygen. Scale bar shown. (C) Localization of detected Sulfur. Scale bar shown. (D) EDX Spectra showing elemental composition of poly(β-Gal-Thr)$_{23}$-*r*-poly(Cys-H)$_3$.

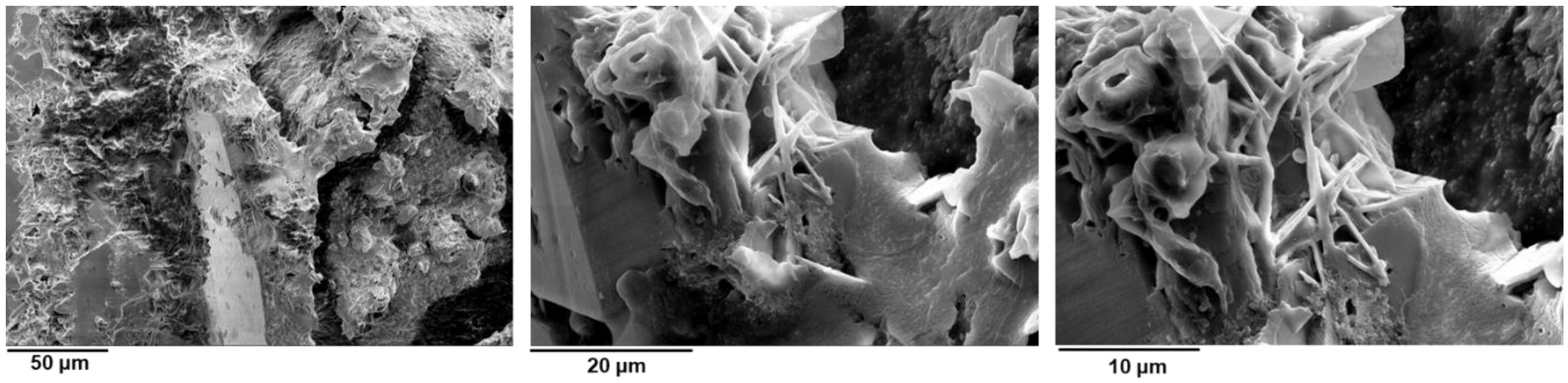

Figure S47. SEM image of poly(β-Gal-Thr)$_{23}$-*r*-poly(Cys-D)$_3$. From left to right Magnification 500X, 2000X, 3500X.

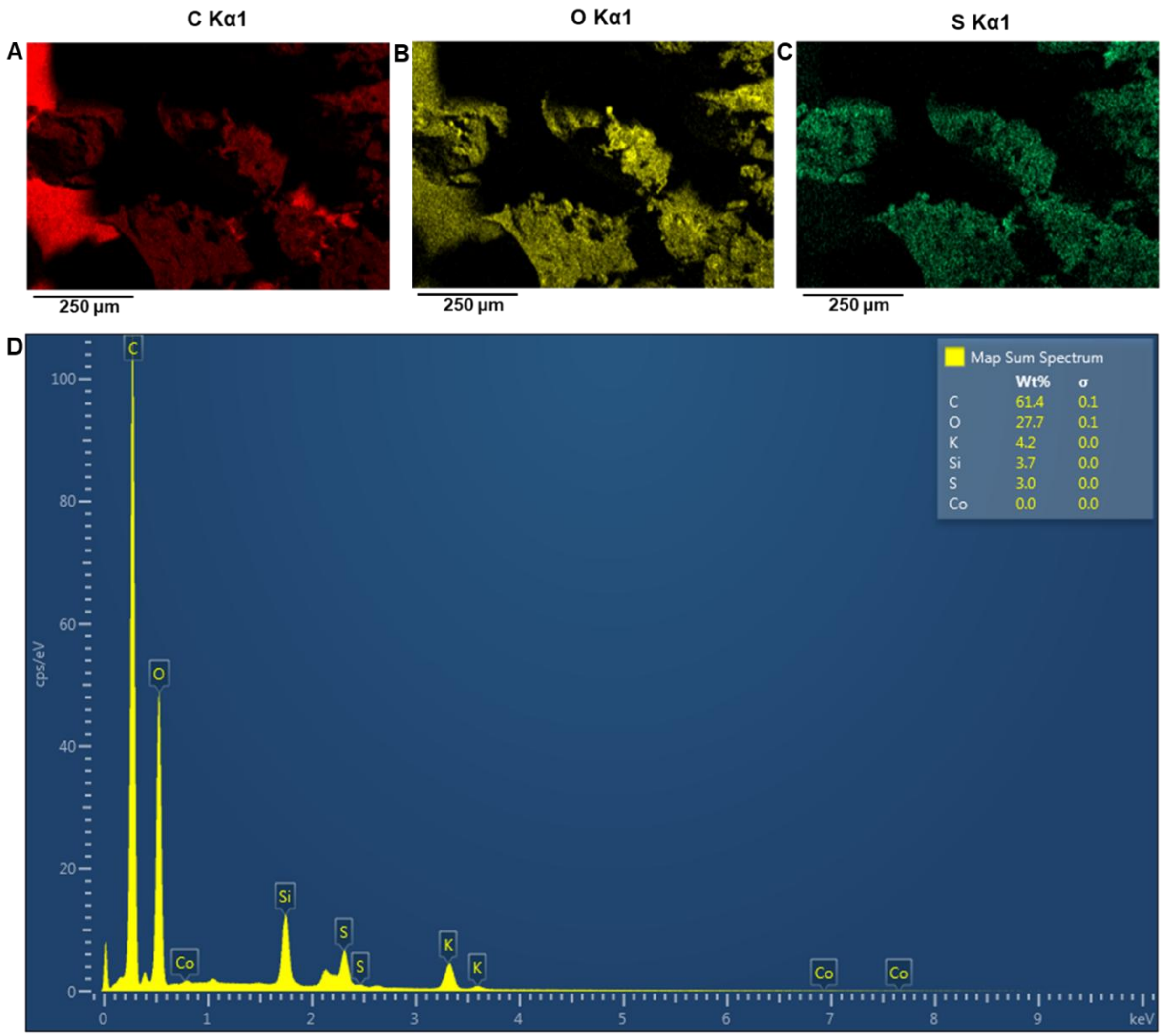

Figure S48. EDX of poly(β-Gal-Thr)$_{23}$-*r*-poly(Cys-D)$_3$. (A) Localization of detected Carbon. Scale bar shown. (B) Localization of detected Oxygen. Scale bar shown. (C) Localization of detected Sulfur. Scale bar shown. (D) EDX Spectra showing elemental composition of poly(β-Gal-Thr)$_{23}$-*r*-poly(Cys-D)$_3$.

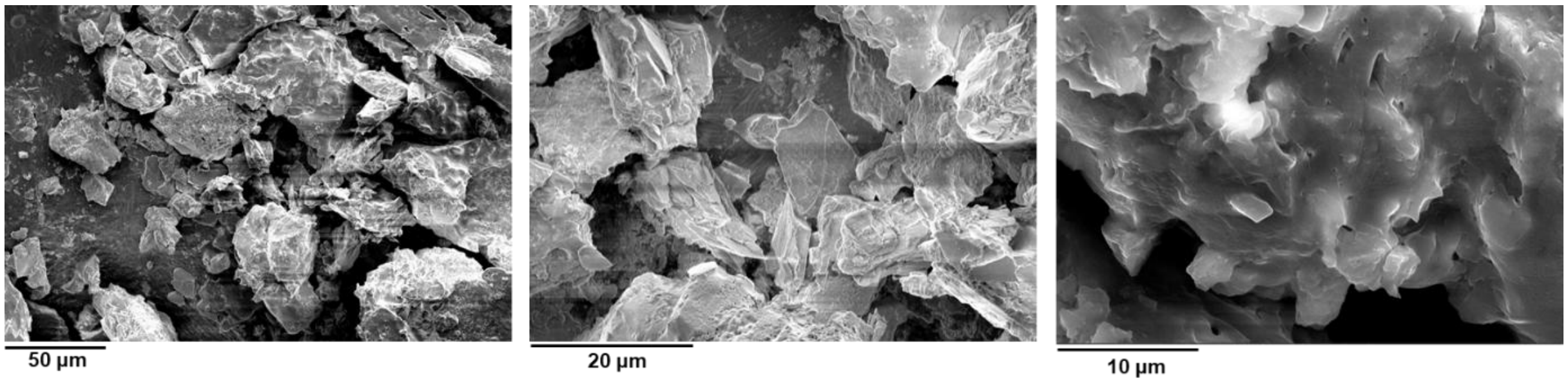

Figure S49. SEM image of poly(β-Gal-Thr)$_{23}$-*r*-poly(Cys-H)$_6$. From left to right Magnification 500X, 2000X 3500X.

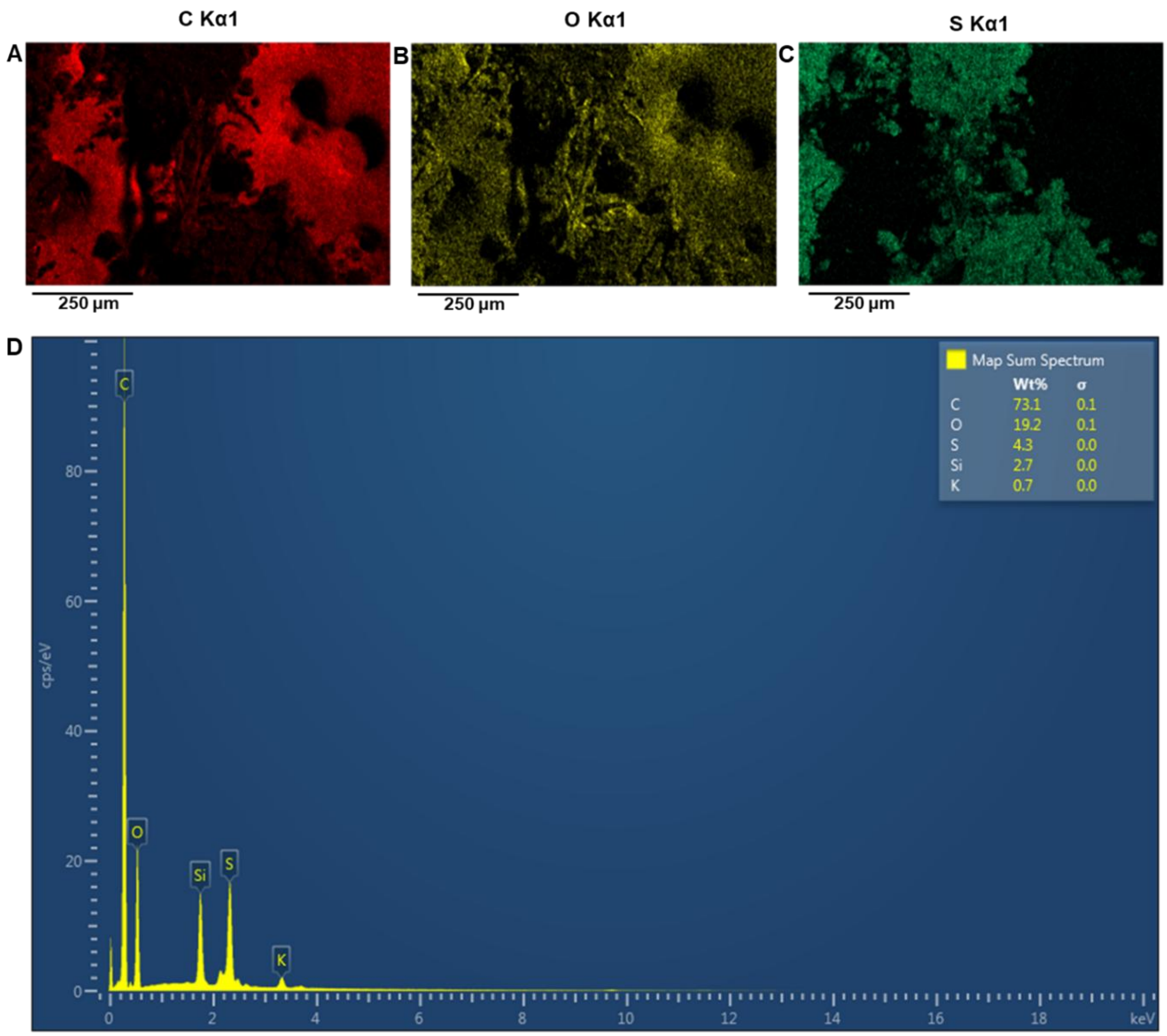

Figure S50. EDX analysis of poly(β-Gal-Thr)$_{23}$-*r*-poly(Cys-H)$_6$. (A) Localization of detected Carbon. Scale bar shown. (B) Localization of detected Oxygen. Scale bar shown. (C) Localization of detected Sulfur. Scale bar shown. (D) EDX Spectra showing elemental composition of poly(β-Gal-Thr)$_{23}$-*r*-poly(Cys-H)$_6$.

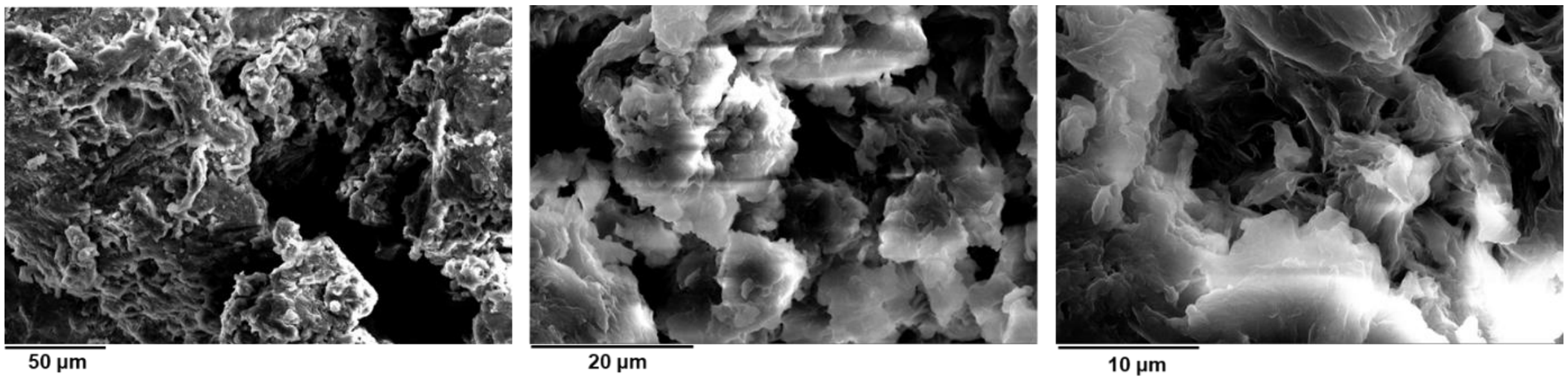

Figure S51. SEM image of poly(β-Gal-Thr)$_{23}$-*r*-poly(Cys-D)$_{6}$. From left to right Magnification 500X, 2000X, 3500X.

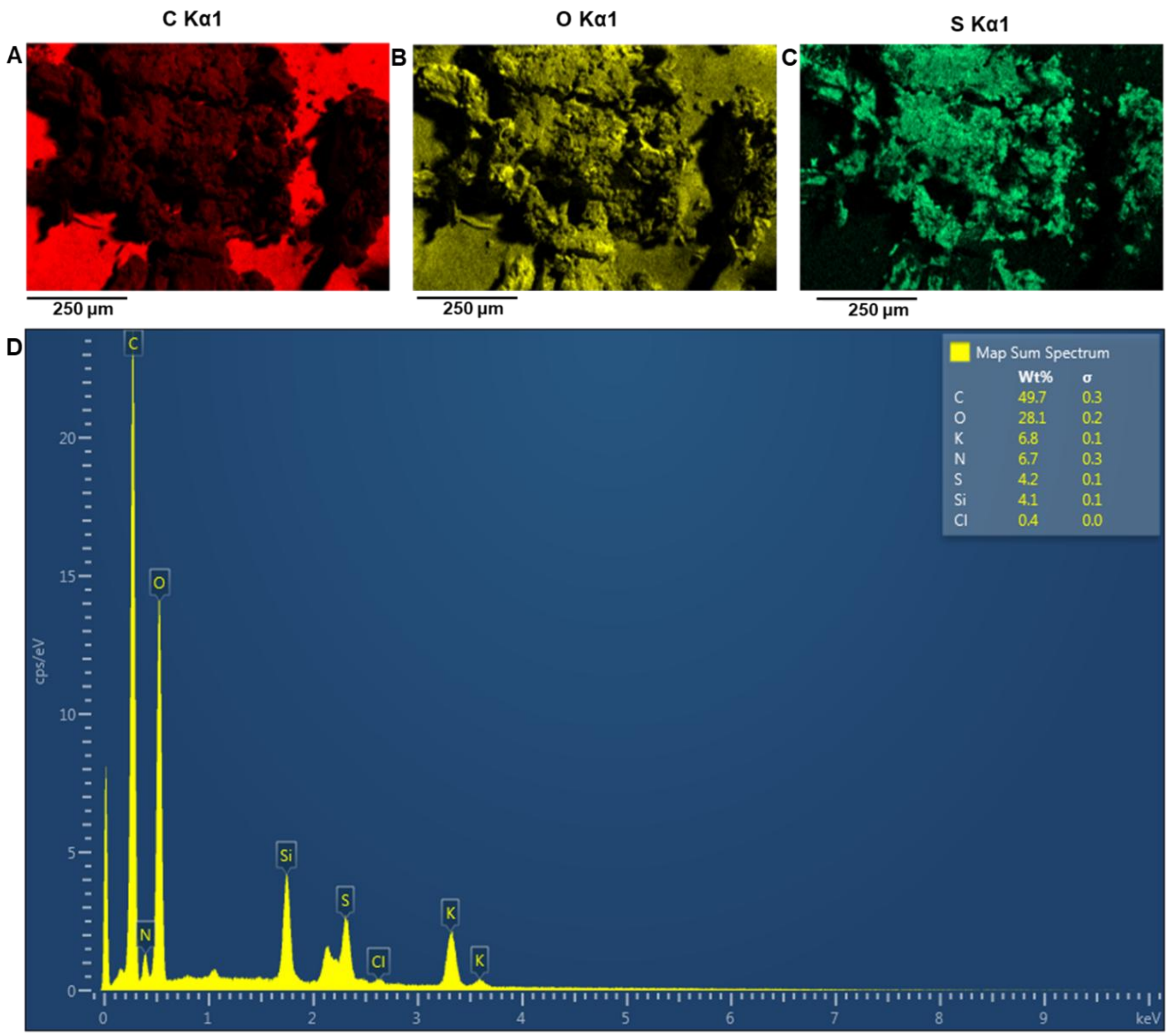

Figure S52. EDX analysis of poly(β-Gal-Thr)$_{23}$-*r*-poly(Cys-D)$_{6}$. (A) Localization of detected Carbon. Scale bar shown. (B) Localization of detected Oxygen. Scale bar shown. (C) Localization of detected Sulfur. Scale bar shown. (D) EDX Spectra showing elemental composition of poly(β-Gal-Thr)$_{23}$-*r*-poly(Cys-D)$_{6}$.

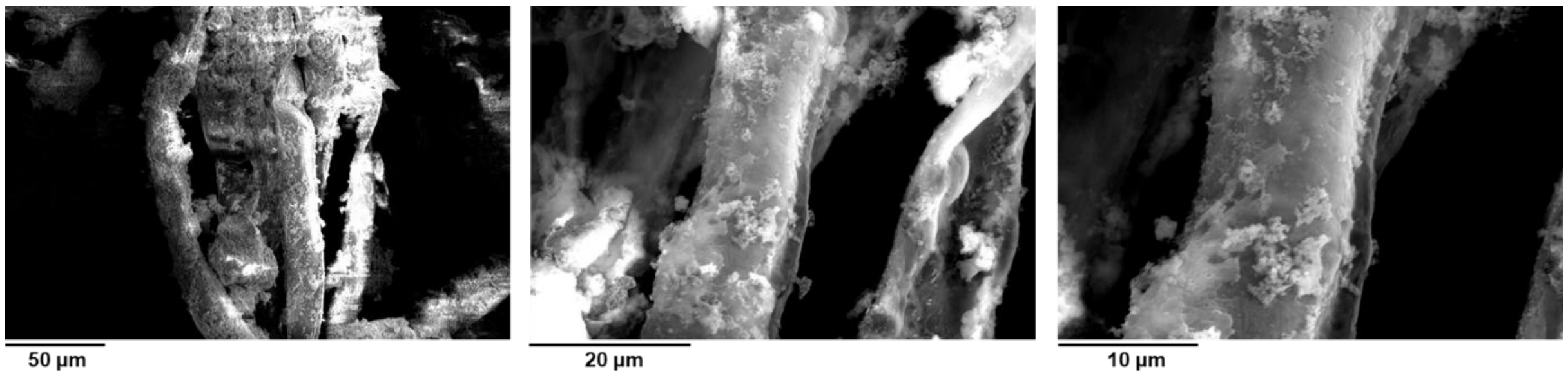

Figure S53. SEM image of poly(β-Gal-Thr)$_{33}$-*r*-poly(Cys-H)$_{3}$. From left to right Magnification 500X, 2000X, 3500X.

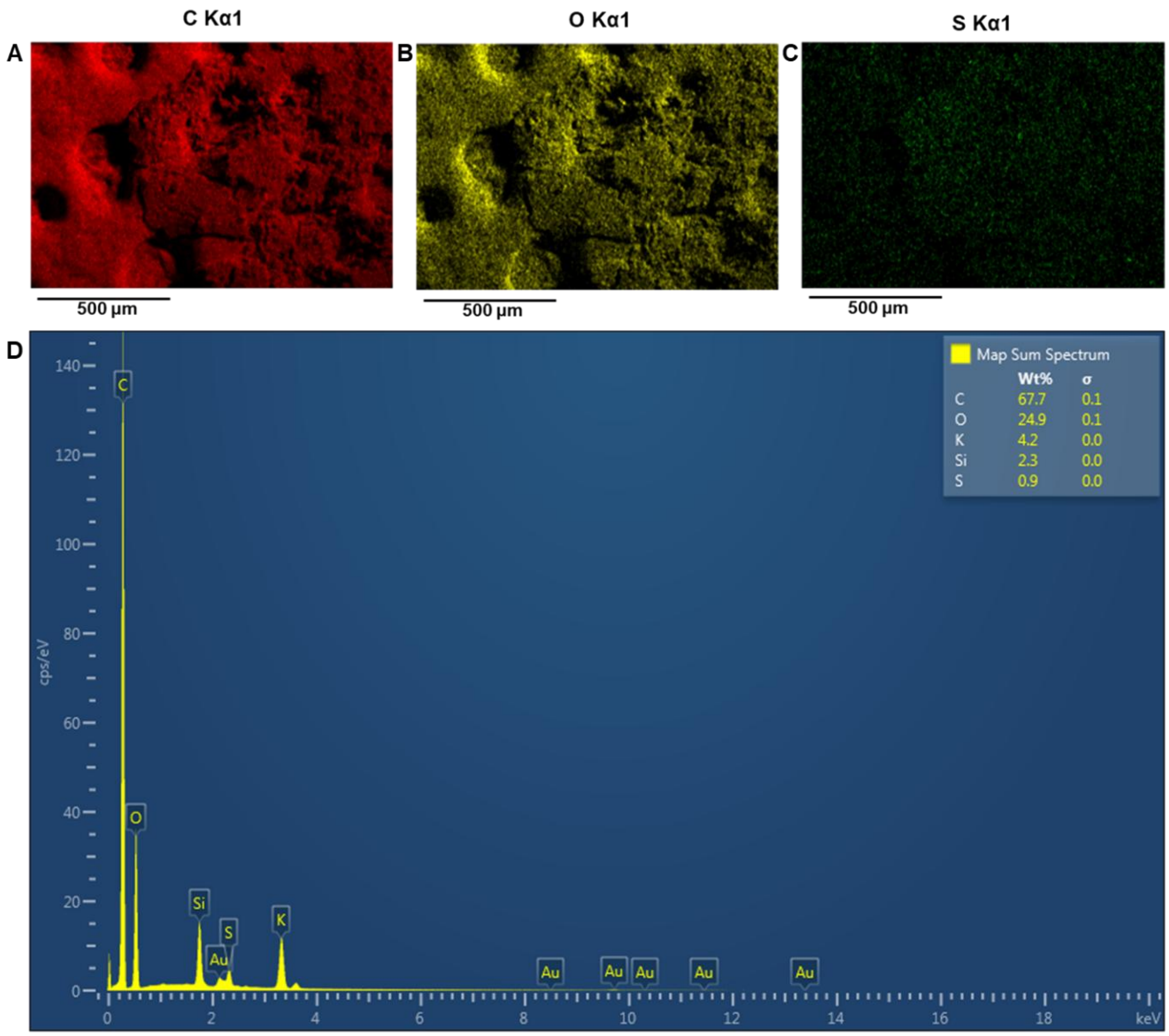

Figure S54. EDX analysis of poly(β-Gal-Thr)$_{33}$-*r*-poly(Cys-H)$_{3}$. (A) Localization of detected Carbon. Scale bar shown. (B) Localization of detected Oxygen. Scale bar shown. (C) Localization of detected Sulfur. Scale bar shown. (D) EDX Spectra showing elemental composition of poly(β-Gal-Thr)$_{33}$-*r*-poly(Cys-H)$_{3}$.

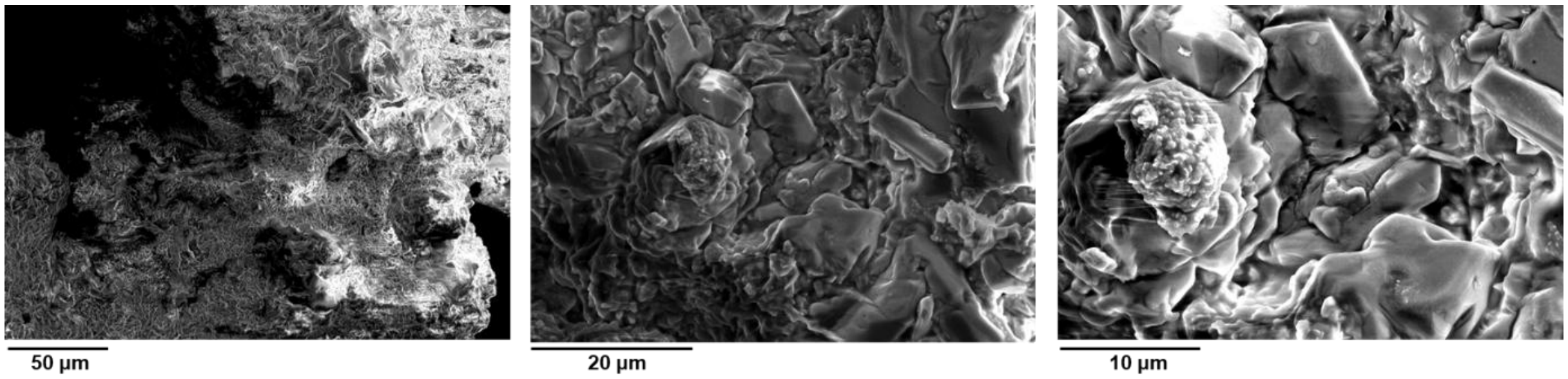

Figure S55. SEM image for poly(β-Gal-Thr)$_{33}$-*r*-poly(Cys-D)$_3$. From left to right Magnification 500X, 2000X, 3500X.

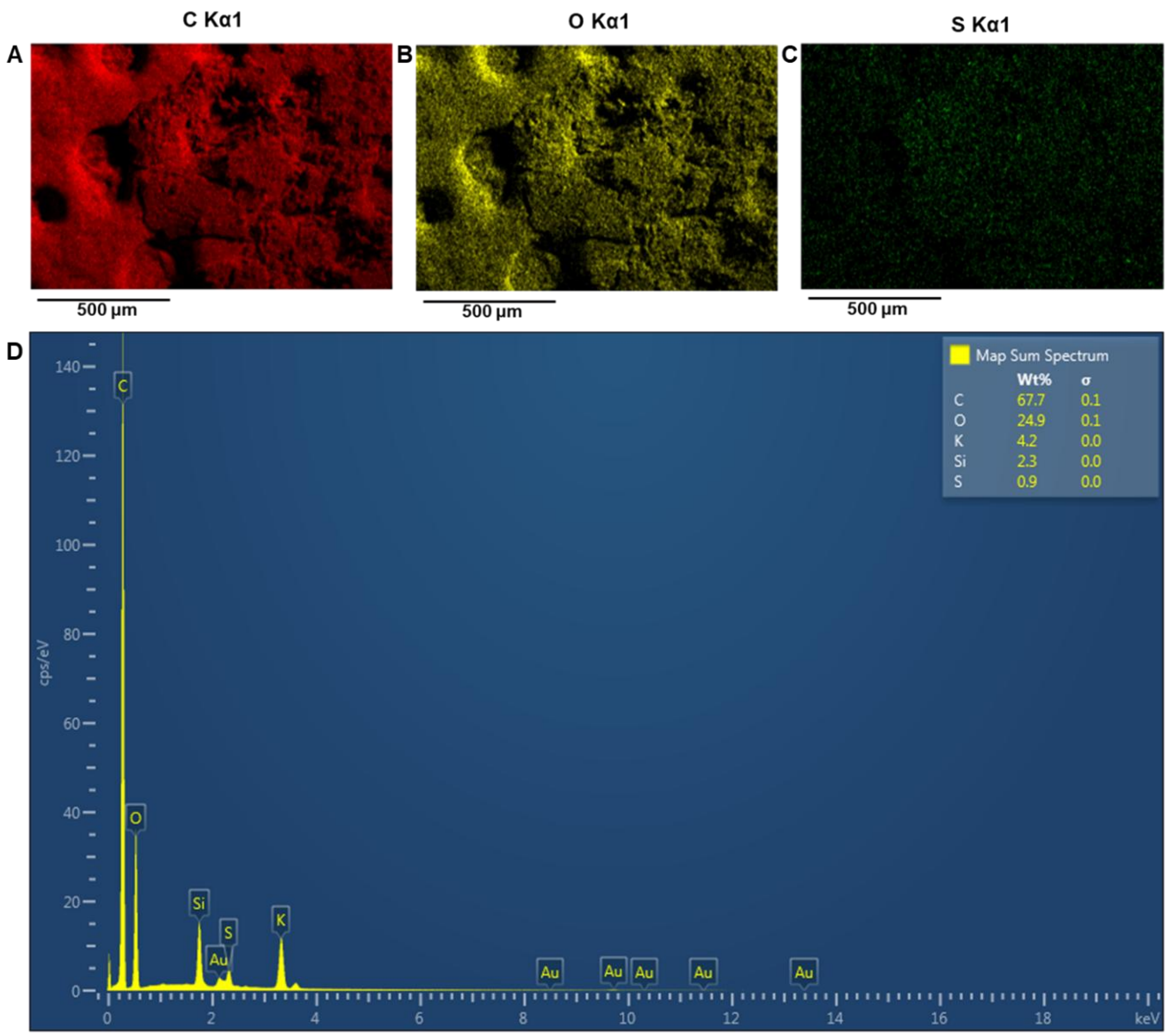

Figure S56. EDX analysis of poly(β-Gal-Thr)$_{33}$-*r*-poly(Cys-D)$_3$. (A) Localization of detected Carbon. Scale bar shown. (B) Localization of detected Oxygen. Scale bar shown. (C) Localization of detected Sulfur. Scale bar shown. (D) EDX Spectra showing elemental composition of poly(β-Gal-Thr)$_{33}$-*r*-poly(Cys-D)$_3$.

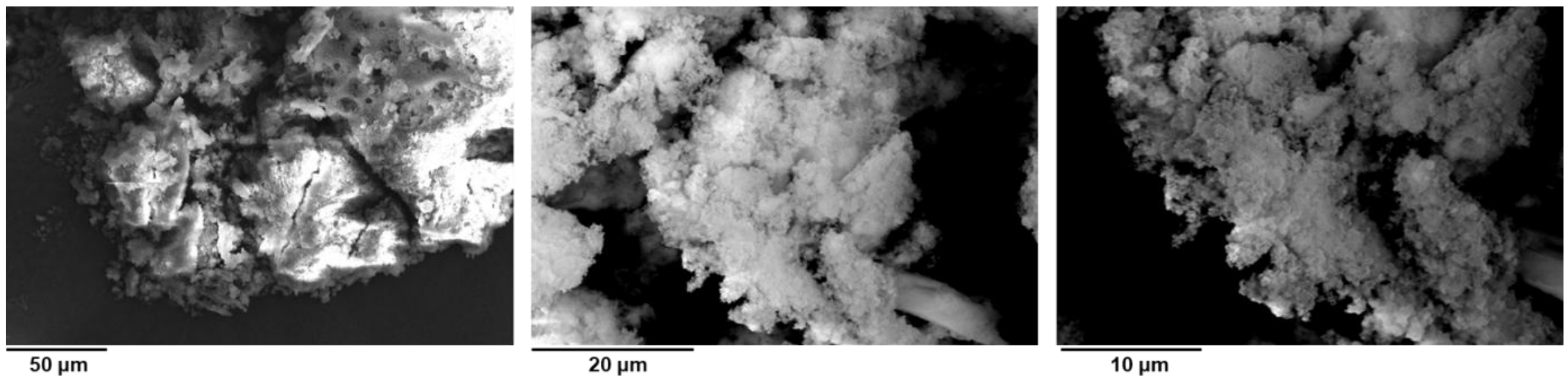

Figure S57. SEM image for Poly(β-Gal-Thr)$_{63}$-*r*-poly(Cys-H)$_{3}$. From left to right Magnification 500X, 2000X, 3500X.

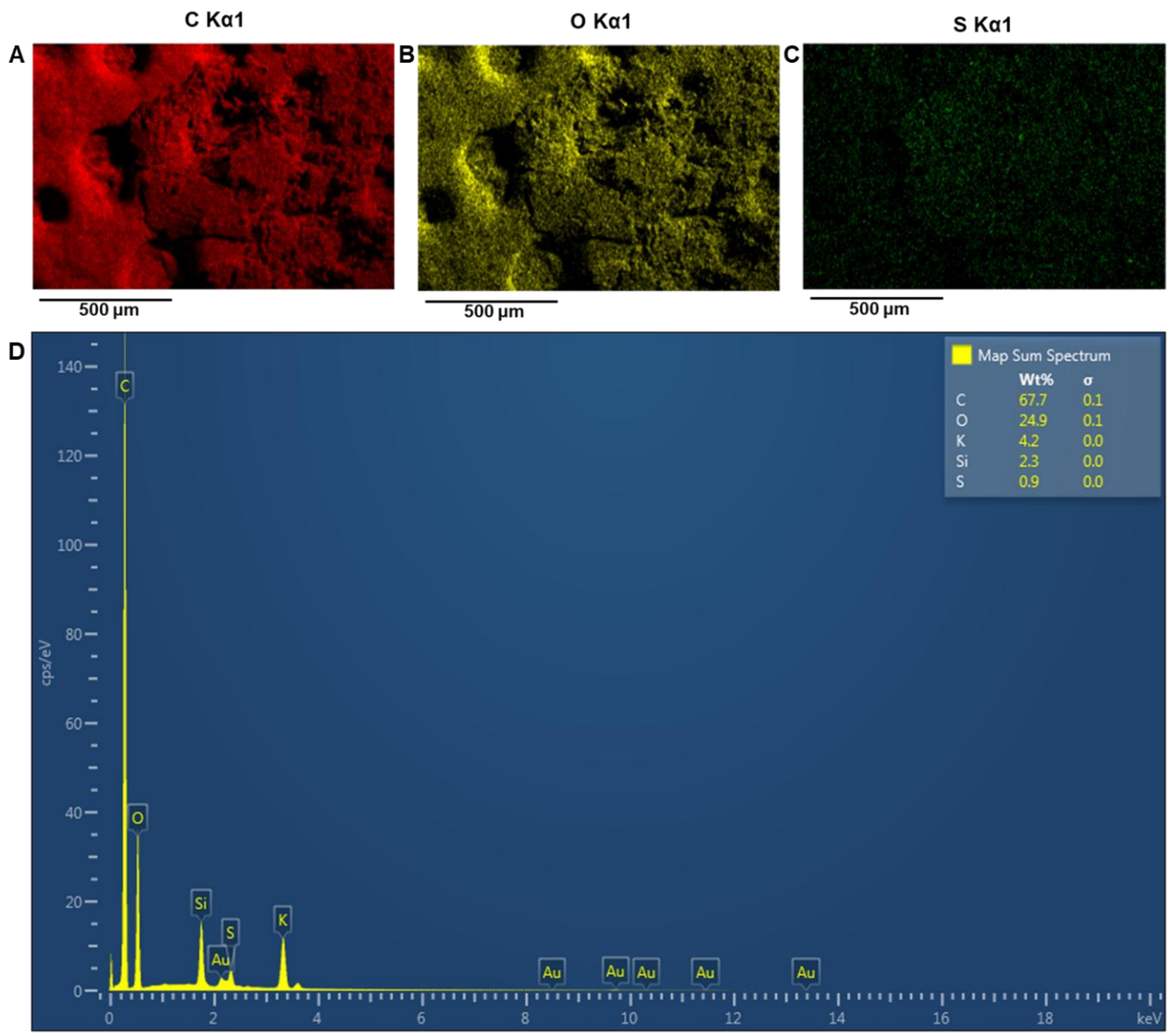

Figure S58. EDX analysis of poly(β-Gal-Thr)$_{63}$-*r*-poly(Cys-H)$_{3}$. (A) Localization of detected Carbon. Scale bar shown. (B) Localization of detected Oxygen. Scale bar shown. (C) Localization of detected Sulfur. Scale bar shown. (D) EDX Spectra showing elemental composition of poly(β-Gal-Thr)$_{63}$-*r*-poly(Cys-H)$_{3}$.

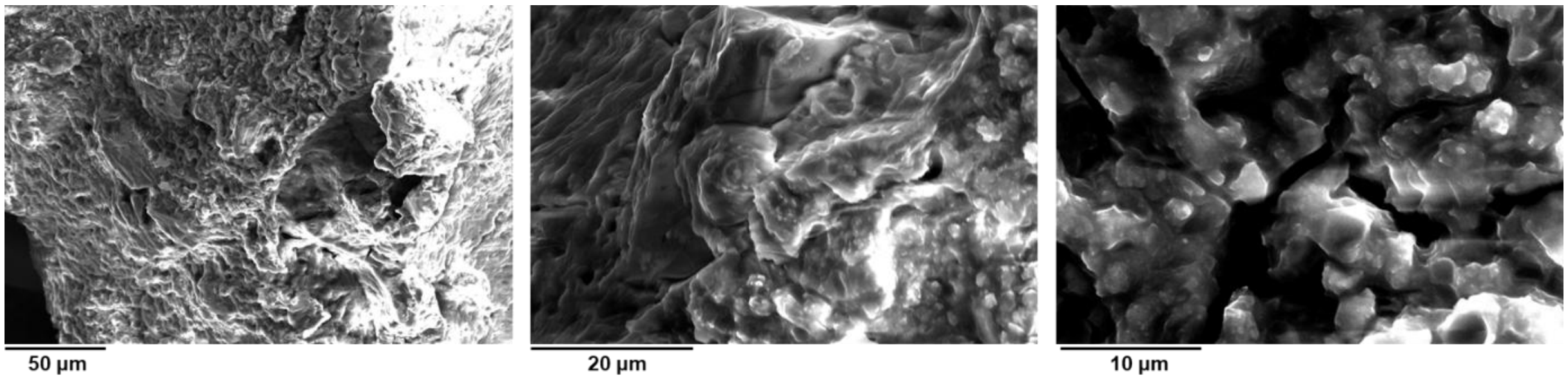

Figure S59. SEM image for poly(β-Gal-Thr)$_{63}$-*r*-poly(Cys-D)$_3$. From left to right Magnification 500X, 2000X, 3500X.

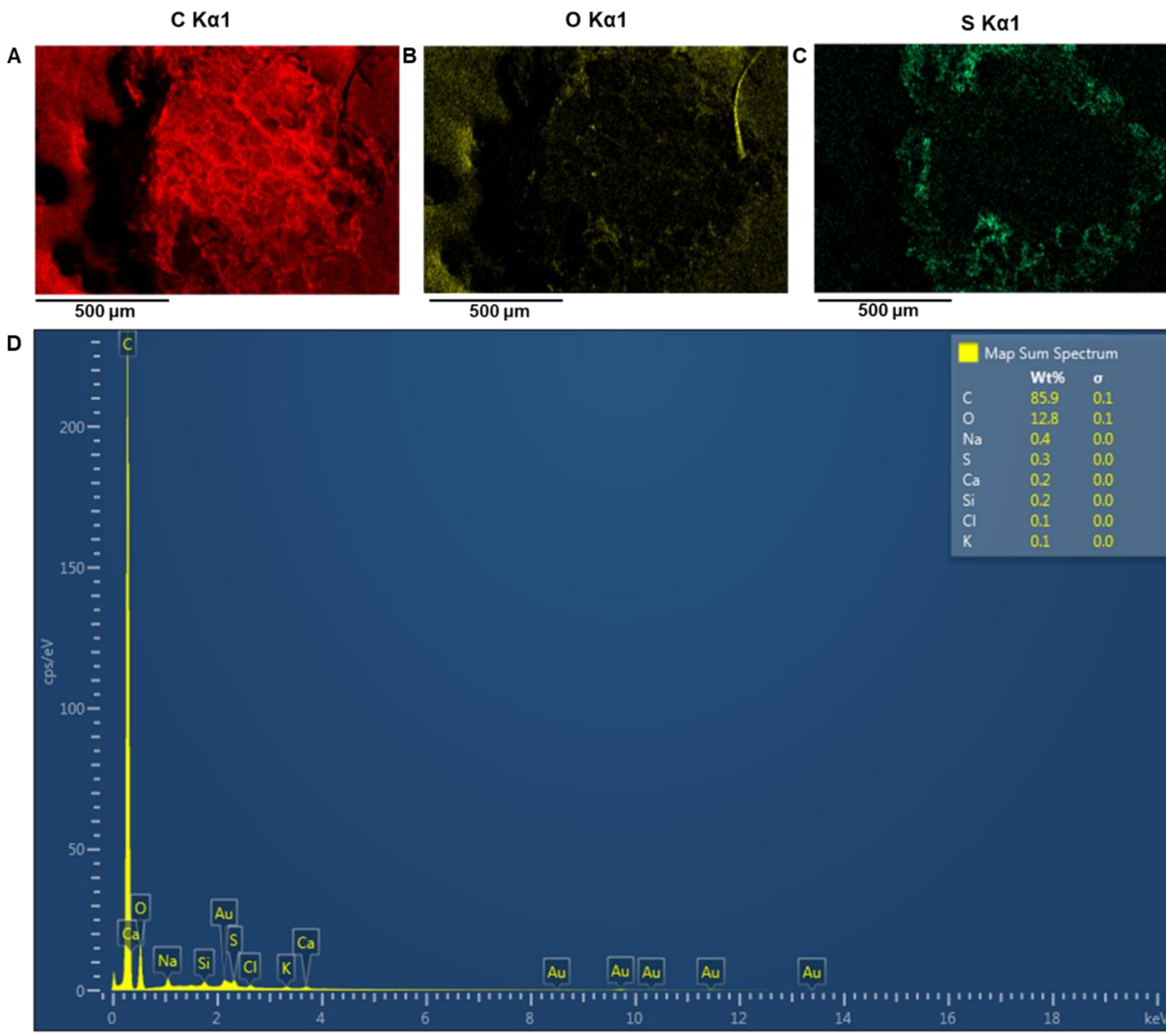

Figure S60. EDX analysis of poly(β-Gal-Thr)$_{63}$-*r*-poly(Cys-D)$_3$. (A) Localization of detected Carbon. Scale bar shown. (B) Localization of detected Oxygen. Scale bar shown. (C) Localization of detected Sulfur. Scale bar shown. (D) EDX Spectra showing elemental composition of poly(β-Gal-Thr)$_{63}$-*r*-poly(Cys-D)$_3$.

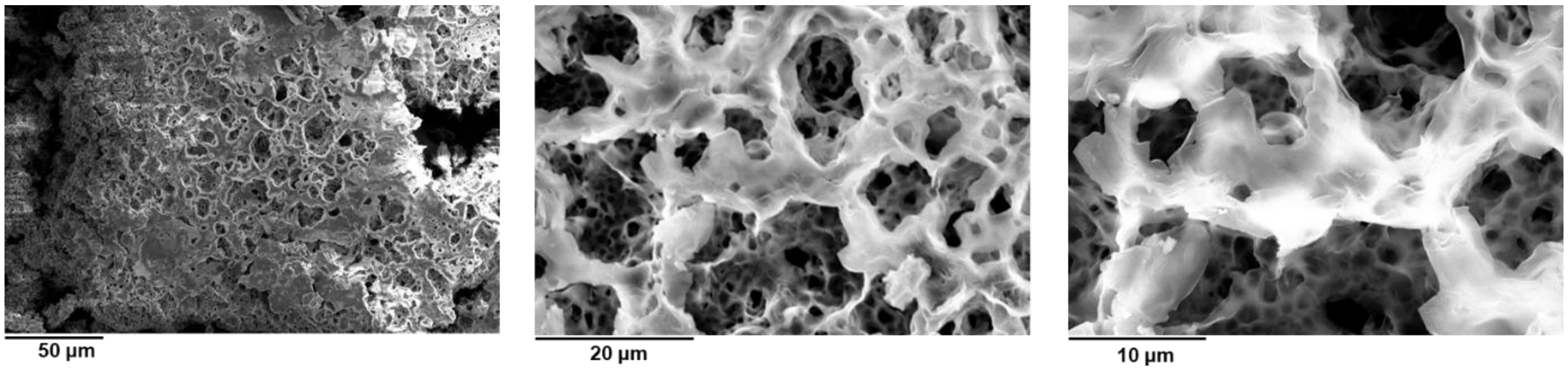

Figure S61. SEM image for poly(β-Gal-Thr)$_{78}$-*r*-poly(Cys-H)$_4$**.** From left to right Magnification 500X, 2000X, 3500X.

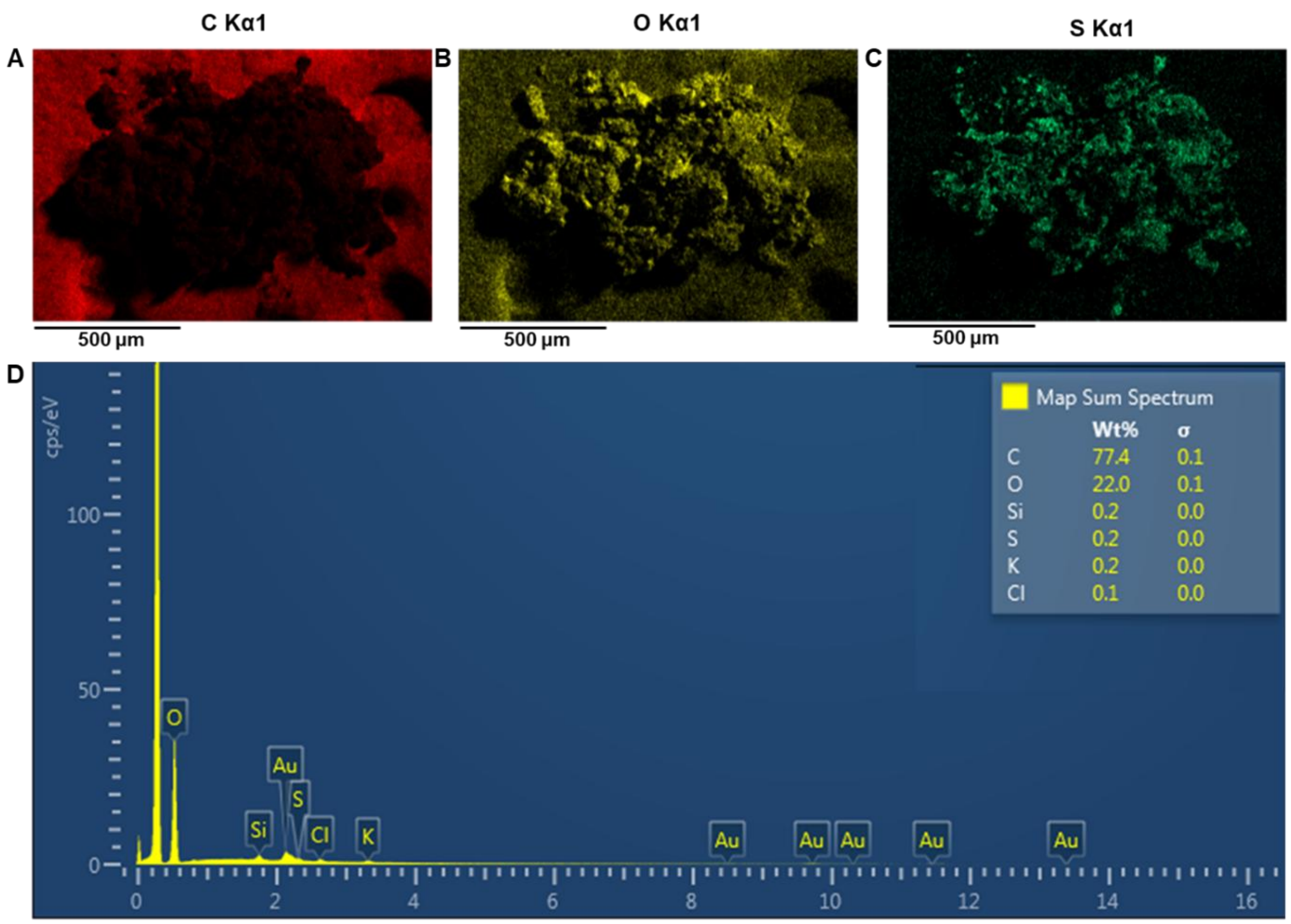

Figure S62. EDX analysis of poly(β-Gal-Thr)$_{78}$-*r*-poly(Cys-H)$_4$. (A) Localization of detected Carbon. Scale bar shown. (B) Localization of detected Oxygen. Scale bar shown. (C) Localization of detected Sulfur. Scale bar shown. (D) EDX Spectra showing elemental composition of poly(β-Gal-Thr)$_{78}$-*r*-poly(Cys-H)$_4$.

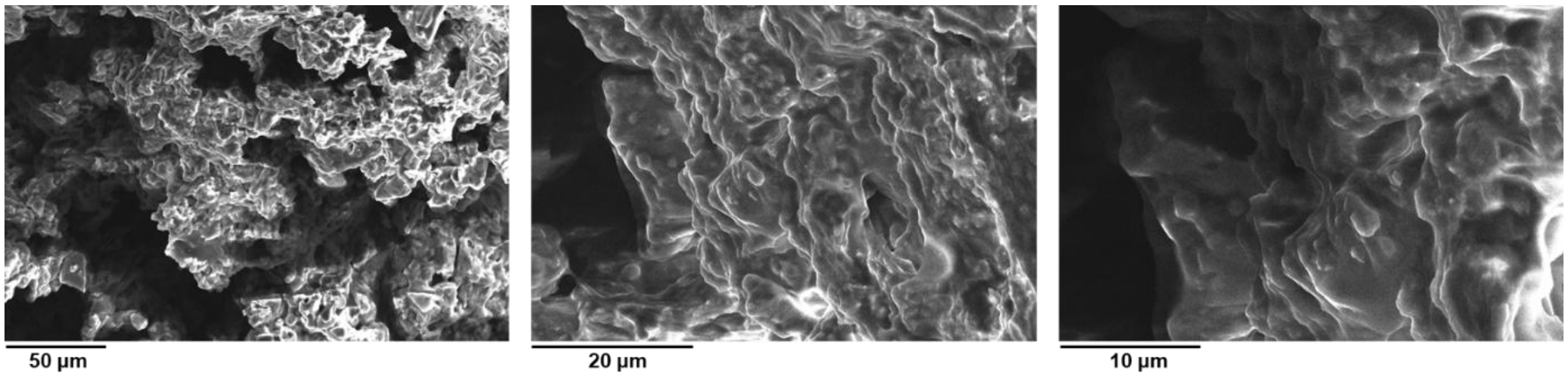

Figure S63. SEM image for poly(β-Gal-Thr)$_{78}$-*r*-poly(Cys-D)$_{4}$. From left to right Magnification 500X, 2000X, 3500X.

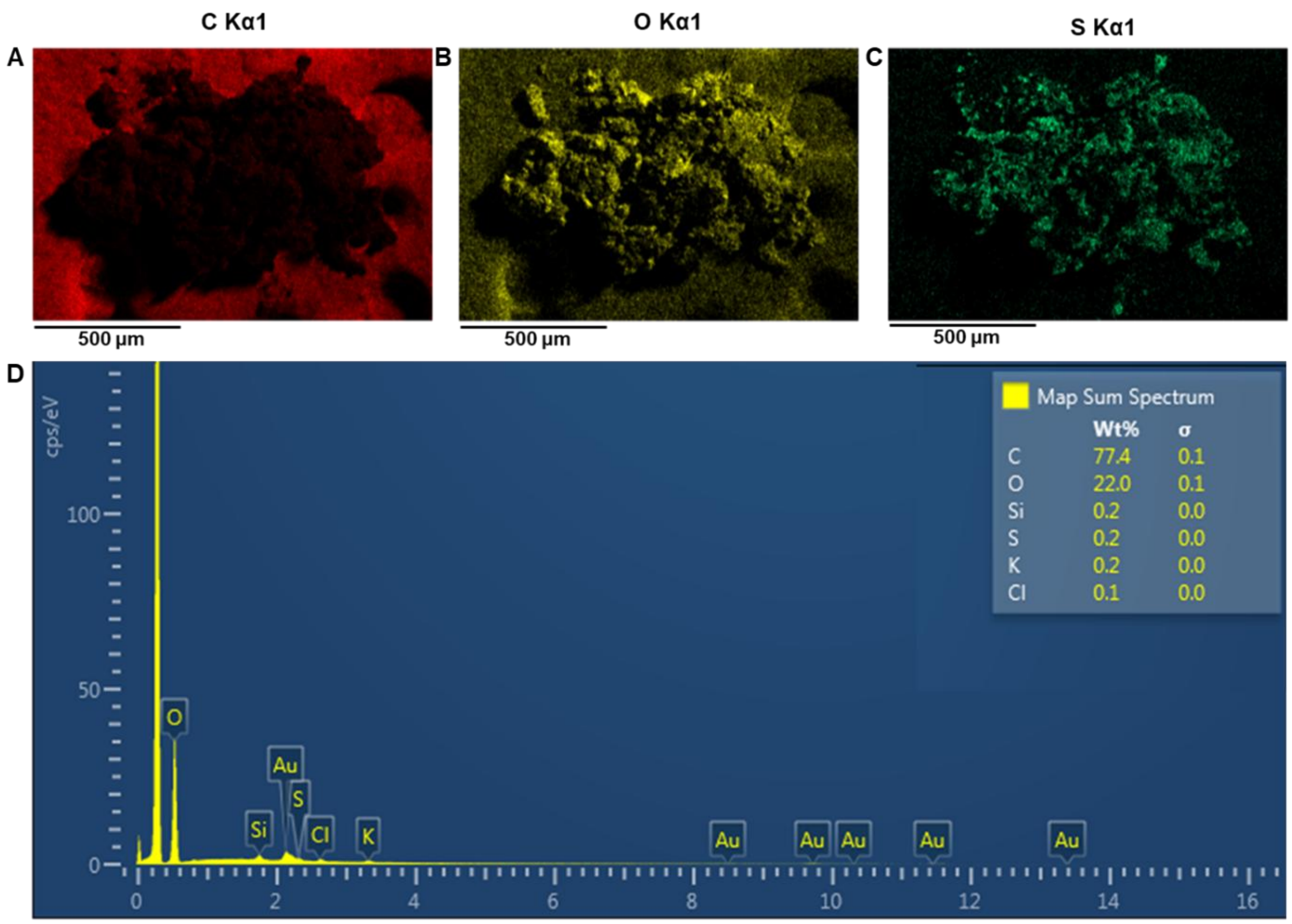

Figure S64. EDX analysis of poly(β-Gal-Thr)$_{78}$-*r*-poly(Cys-D)$_{4}$. (A) Localization of detected Carbon. Scale bar shown. (B) Localization of detected Oxygen. Scale bar shown. (C) Localization of detected Sulfur. Scale bar shown. (D) EDX Spectra showing elemental composition of poly(β-Gal-Thr)$_{78}$-*r*-poly(Cys-D)$_{4}$.

5. Dynamic vapor sorption of poly(*β*-Gal-Thr)$_m$-*r*-poly(Cys-H)$_n$ and poly(*β*-Gal-Thr)$_m$-*r*-poly(Cys-D)$_n$

General methods. Water sorption isotherms of poly(*β*-Gal-Thr)$_m$-*r*-poly(Cys-H)$_n$ and poly(*β*-Gal-Thr)$_m$-*r*-poly(Cys-D)$_n$ were measured using dynamic vapor sorption (DVS) conducted using a DVS Intrinsic system (Surface Measurement Systems) at 25 °C. Approximately ~1-4 mg of poly(*β*-Gal-Thr)$_m$-*r*-poly(Cys-H)$_n$ or poly(*β*-Gal-Thr)$_m$-*r*-poly(Cys-D)$_n$ was loaded onto the ultra-sensitive microbalance of the DVS instrument. Each sample underwent 2-3 consecutive sorption-desorption cycles between 8 % and 82 % RH. In every cycle, RH was increased from 8 % - 82 % and decreased back to 5 % in 10 % RH increments.[7] Prior to each subsequent RH step, equilibration was defined as either a mass change rate (dm/dt) below 0.05% mg/min sustained for 5 consecutive mins or a minimum hold time of 20 mins[8]. The mass percent vapor sorption (%VS) at each RH was calculated as Eq. 1:

$$\%VS = \frac{(m - m_{5\%RH})}{m_{5\%RH}} \times 100\% \quad \text{(Eq. 1)}$$

where m is the sample mass at a given RH and $m_{5\%RH}$ is the reference mass of each polymer at 5% RH.

DVS Isotherm Analysis Report

Meth: Chengyu 8.3-82.3% per 10% 3 cycles 0.05%
Temp: 24.8 °C
MRef: 0.8522 from Mass at end of first stage

	Target	Change In Mass (%) - ref		
	% P/Po	Sorption	Desorption	Hysteresis
Cycle 1	8.3	0.00	0.57	
	12.8	0.81	2.26	1.45
	21.0	1.83	3.57	1.74
	29.3	2.44	4.34	1.90
	37.8	4.53	4.65	0.12
	46.3	5.28	4.40	-0.88
	55.1	6.58	5.09	-1.49
	63.6	8.75	7.39	-1.36
	72.5	13.00	12.24	-0.76
	82.3	20.16	20.16	
Cycle 2	8.3	0.57	1.70	
	12.8	0.88	2.07	1.19
	21.0	1.71	3.20	1.49
	29.3	2.43	3.39	0.96
	37.8	3.60	4.27	0.67
	46.3	4.76	6.17	1.41
	55.1	5.79	6.59	0.81
	63.6	7.56	8.39	0.83
	72.5	12.70	13.17	0.47
	82.3	20.32	20.32	
Cycle 3	8.3	1.70	3.50	
	12.8	1.74	4.39	2.65
	21.0	2.78	5.14	2.36
	29.3	3.67	5.76	2.09
	37.8	4.72	6.83	2.11
	46.3	5.83	7.63	1.80
	55.1	7.05	8.12	1.07
	63.6	9.59	10.57	0.98
	72.5	14.54	15.29	0.75
	82.3	22.73	22.73	

DVS from SMS - World leader in Sorption Science

© Surface Measurement Systems Ltd 1996-2018

Figure S65. DVS Isotherm analysis report for poly(β-Gal-Thr)$_{22}$.

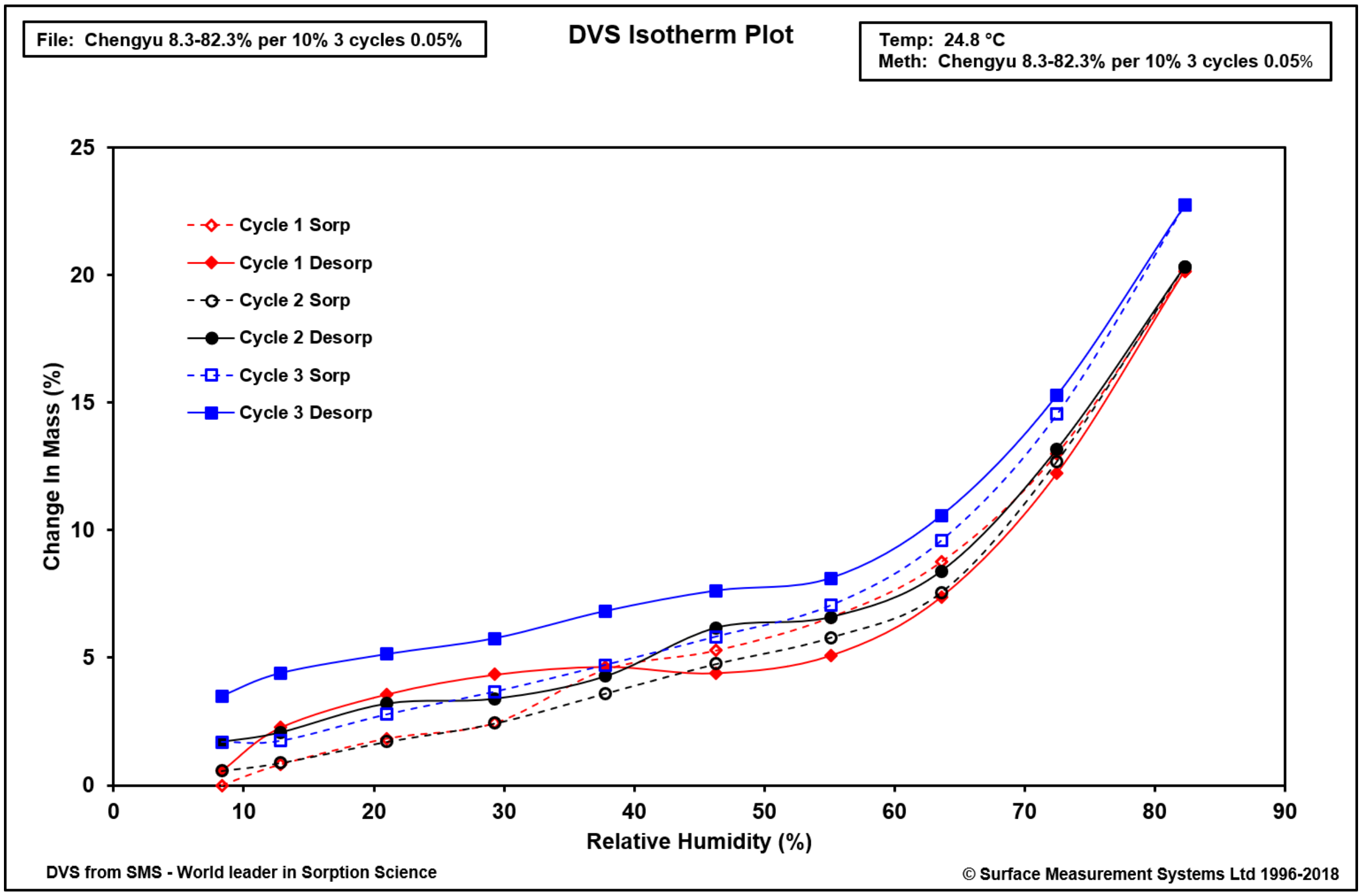

Figure S66. DVS Isotherm plot for poly(*β*-Gal-Thr)$_{22}$.

DVS Isotherm Analysis Report

Meth: Chengyu 8.3-82.3% per 10% 3 cycles 0.05%
Temp: 24.8 °C
MRef: 1.1469 from Mass at end of first stage

	Target	Change In Mass (%) - ref		
	% P/Po	Sorption	Desorption	Hysteresis
Cycle 1	8.3	0.72	1.45	
	12.8	1.46	2.05	0.59
	21.0	2.49	3.64	1.15
	29.3	3.84	5.25	1.41
	37.8	5.58	6.62	1.04
	46.3	7.28	8.30	1.02
	55.1	9.36	9.90	0.54
	63.6	11.59	12.80	1.21
	72.5	16.67	18.21	1.54
	82.3	25.43	25.43	
Cycle 2	8.3	1.45	1.66	
	12.8	1.88	2.35	0.47
	21.0	2.62	3.89	1.27
	29.3	3.83	5.49	1.66
	37.8	5.26	7.01	1.75
	46.3	7.03	8.36	1.33
	55.1	9.22	10.27	1.05
	63.6	12.14	13.31	1.17
	72.5	17.06	18.95	1.89
	82.3	25.44	25.44	
Cycle 3	8.3	1.66	0.00	
	12.8	1.94	1.39	-0.55
	21.0	2.46	2.39	-0.07
	29.3	3.53	3.97	0.44
	37.8	4.63	5.46	0.83
	46.3	5.57	6.92	1.35
	55.1	7.54	8.76	1.22
	63.6	9.90	11.72	1.81
	72.5	14.94	16.86	1.92
	82.3	23.38	23.38	

DVS from SMS - World leader in Sorption Science

© Surface Measurement Systems Ltd 1996-2018

Figure S67. DVS isotherm analysis report for poly(*β*-Gal-Thr)$_{23}$-*r*-poly(Cys-H)$_{1}$.

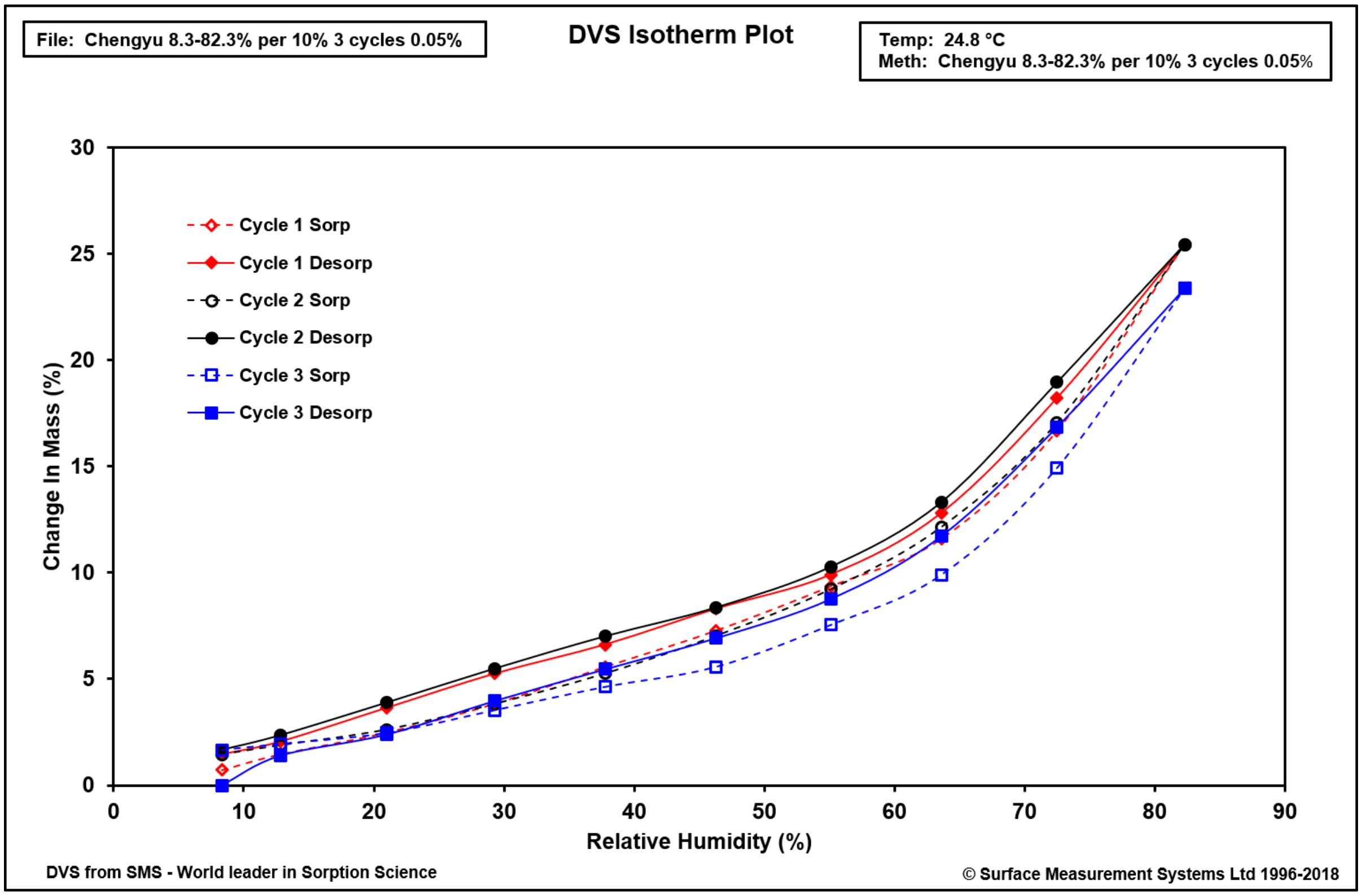

Figure S68. DVS Isotherm plot for poly(β-Gal-Thr)$_{23}$-*r*-poly(Cys-H)$_1$.

DVS Isotherm Analysis Report

Meth: Chengyu 8.3-82.3% per 10% 3 cycles 0.05%
Temp: 24.9 °C
MRef: 1.7112 from Sample Weight

	Target	Change In Mass (%) - ref		
	% P/Po	Sorption	Desorption	Hysteresis
Cycle 1	8.3	0.00	-1.69	
	12.8	0.03	-0.66	-0.69
	21.0	1.40	1.50	0.10
	29.3	2.99	3.54	0.55
	37.8	4.44	5.28	0.84
	46.3	5.63	6.88	1.25
	55.1	6.94	8.46	1.52
	63.6	10.21	11.06	0.85
	72.5	15.22	16.51	1.29
	82.3	22.71	22.71	
Cycle 2	8.3	-1.69	-1.55	
	12.8	-1.06	-0.46	0.60
	21.0	0.29	1.60	1.31
	29.3	1.96	3.55	1.59
	37.8	3.78	5.36	1.58
	46.3	5.66	6.92	1.26
	55.1	7.75	8.50	0.75
	63.6	10.30	10.81	0.51
	72.5	14.78	15.99	1.21
	82.3	21.73	21.73	
Cycle 3	8.3	-1.55	-1.94	
	12.8	-1.05	-0.95	0.10
	21.0	0.37	1.24	0.87
	29.3	2.04	3.31	1.27
	37.8	3.75	5.11	1.36
	46.3	5.65	6.71	1.07
	55.1	7.71	8.19	0.48
	63.6	10.17	10.44	0.27
	72.5	14.34	15.57	1.23
	82.3	21.34	21.34	

DVS from SMS - World leader in Sorption Science

© Surface Measurement Systems Ltd 1996-2018

Figure S69. DVS isotherm analysis report for poly(β-Gal-Thr)$_{23}$-*r*-poly(Cys-D)$_1$.

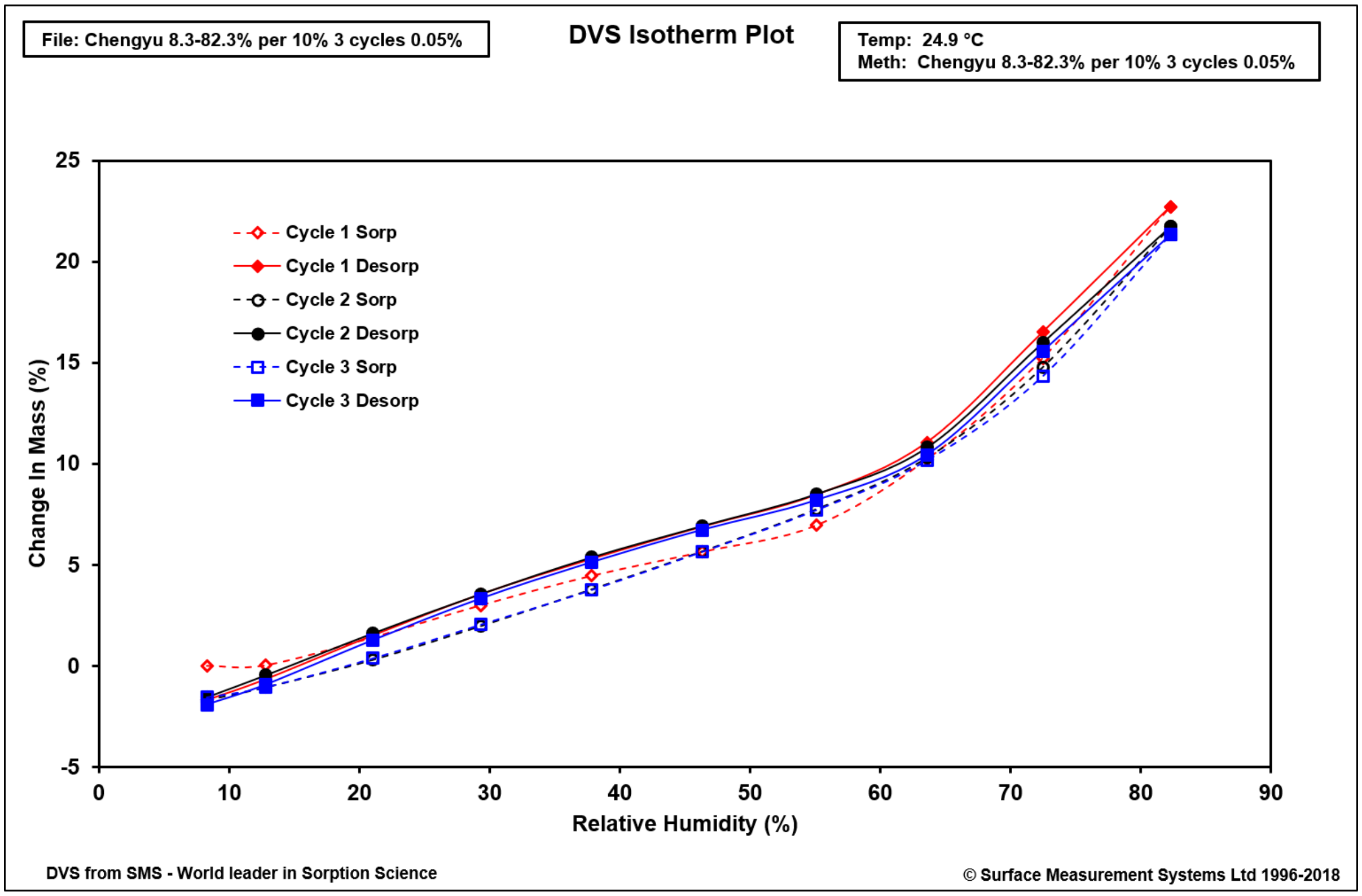

Figure S70. DVS Isotherm plot for poly(β-Gal-Thr)$_{23}$-*r*-poly(Cys-D)$_1$.

DVS Isotherm Analysis Report

Meth: Chengyu 8.3-82.3% per 10% 3 cycles 0.05%
Temp: 24.8 °C
MRef: 0.9533 from Mass at end of first 0.0 P/Po stage

	Target	Change In Mass (%) - ref		
	% P/Po	Sorption	Desorption	Hysteresis
Cycle 1	8.3	-2.51	-0.57	
	12.8	-2.18	-0.02	2.16
	21.0	-1.19	0.71	1.90
	29.3	0.22	1.59	1.37
	37.8	1.65	2.60	0.95
	46.3	3.51	3.94	0.43
	55.1	5.87	6.27	0.40
	63.6	10.04	10.32	0.28
	72.5	16.54	16.95	0.41
	82.3	27.04	27.04	
Cycle 2	8.3	-0.57	0.03	
	12.8	-0.87	0.34	1.21
	21.0	-0.69	1.00	1.69
	29.3	-0.19	2.01	2.20
	37.8	0.83	3.20	2.37
	46.3	2.21	4.32	2.11
	55.1	4.55	6.24	1.69
	63.6	9.22	10.21	0.99
	72.5	16.16	16.97	0.81
	82.3	27.17	27.17	
Cycle 3	8.3	0.03	0.00	
	12.8	0.02	0.15	0.13
	21.0	0.30	1.07	0.77
	29.3	0.41	1.98	1.57
	37.8	1.21	2.86	1.66
	46.3	2.43	4.42	1.98
	55.1	4.78	6.47	1.69
	63.6	9.30	10.28	0.98
	72.5	16.37	17.19	0.82
	82.3	27.13	27.13	

DVS from SMS - World leader in Sorption Science

© Surface Measurement Systems Ltd 1996-2018

Figure S71. DVS isotherm analysis report for poly(β-Gal-Thr)$_{23}$-*r*-poly(Cys-H)$_2$.

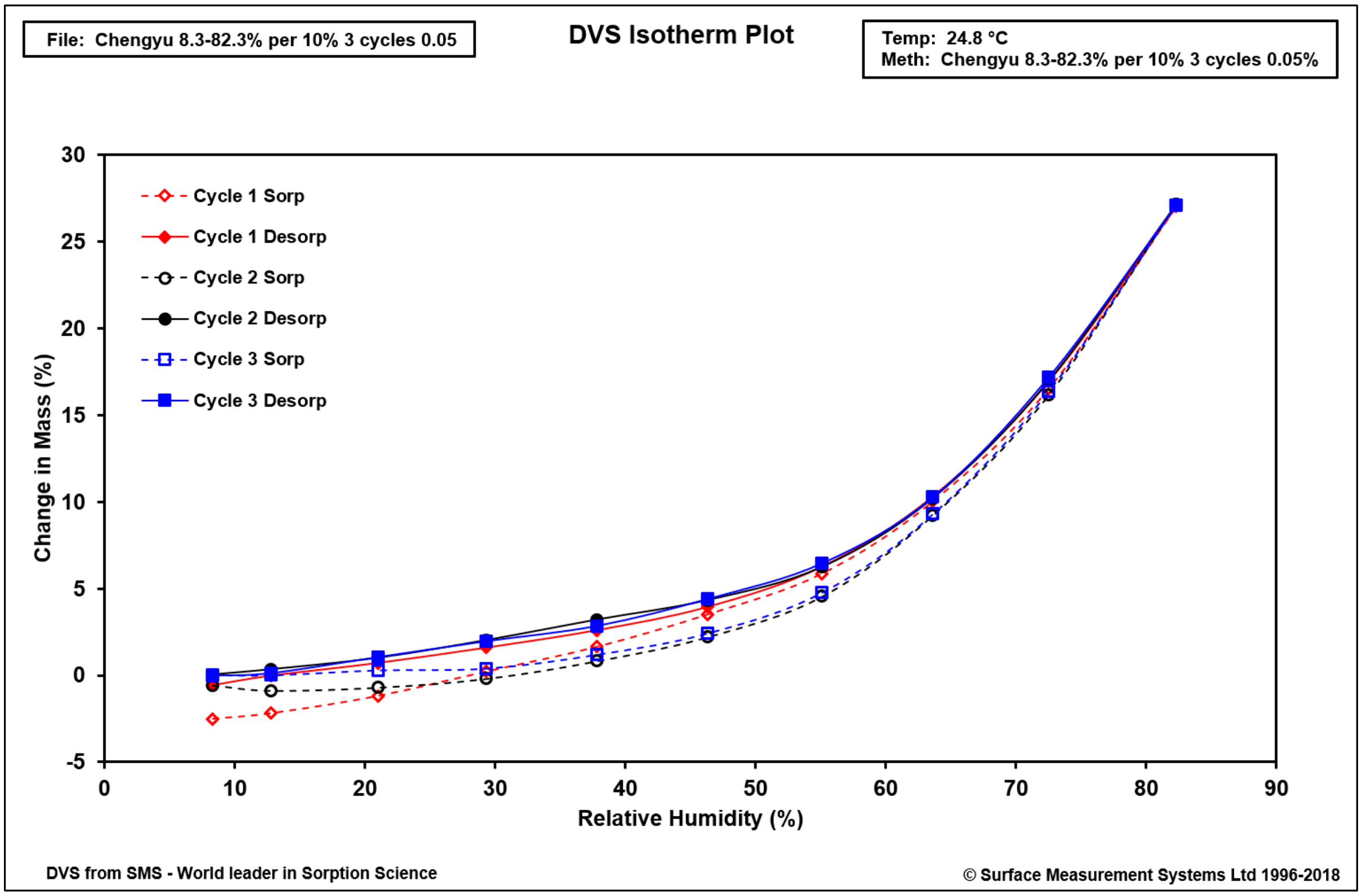

Figure S72. DVS isotherm plot for poly(β-Gal-Thr)$_{23}$-*r*-poly(Cys-H)$_2$.

DVS Isotherm Analysis Report

Meth: Chengyu 8.3-82.3% per 10% 3 cycles 0.05% - Copy
Temp: 24.9 °C
MRef: 1.7112 from Sample Weight

	Target % P/Po	Change In Mass (%) - ref Sorption	Desorption	Hysteresis
Cycle 1	8.3	0.00	-1.69	
	12.8	0.03	-0.66	0.69
	21.0	1.40	1.60	0.21
	29.3	2.99	2.55	0.43
	37.8	5.44	6.28	0.84
	46.3	5.63	6.82	1.19
	55.1	6.94	8.46	1.52
	63.6	11.92	12.06	0.14
	72.5	16.22	17.51	1.29
	82.3	22.92	22.92	
Cycle 2	8.3	-0.28	0.29	
	12.8	0.20	1.06	0.86
	21.0	0.38	2.29	1.91
	29.3	1.24	3.47	2.22
	37.8	2.44	4.76	2.32
	46.3	4.02	6.13	2.11
	55.1	5.97	7.78	1.82
	63.6	9.28	10.45	1.17
	72.5	14.32	15.50	1.18
	82.3	22.54	22.54	
Cycle 3	8.3	-1.55	-1.94	
	12.8	1.03	0.96	0.08
	21.0	0.37	1.24	0.87
	29.3	2.05	3.31	1.26
	37.8	4.75	5.17	0.42
	46.3	5.69	6.55	0.86
	55.1	8.71	9.19	0.48
	63.6	11.17	11.96	0.79
	72.5	14.34	15.57	1.23
	82.3	22.34	22.34	

DVS from SMS - World leader in Sorption Science

© Surface Measurement Systems Ltd 1996-2018

Figure S73. DVS isotherm analysis report for poly(β-Gal-Thr)$_{23}$-*r*-poly(Cys-D)$_2$.

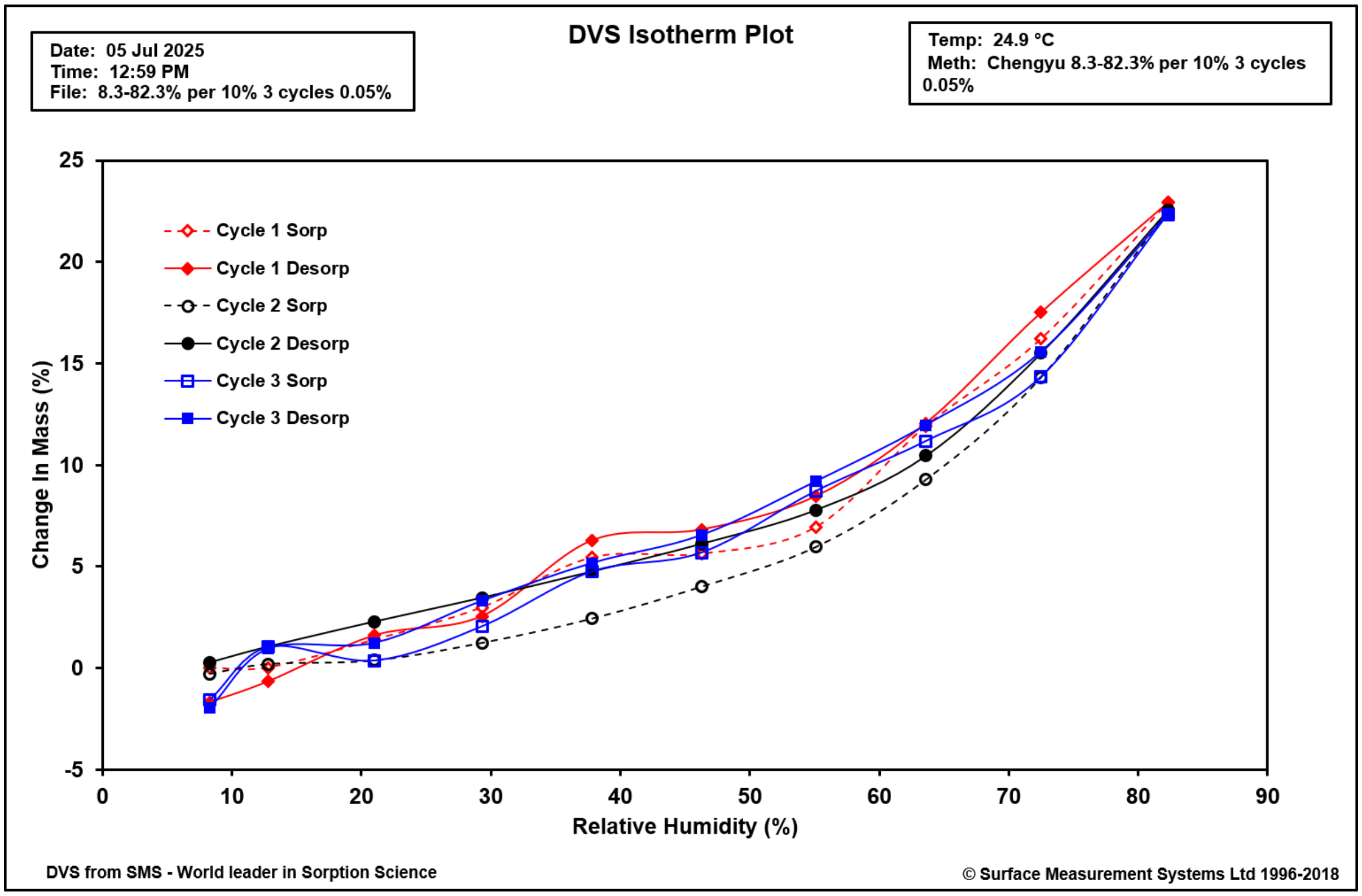

Figure S74. DVS isotherm plot for poly(*β*-Gal-Thr)$_{23}$-*r*-poly(Cys-D)$_2$.

DVS Isotherm Analysis Report

Meth: Chengyu 8-82% per 10% 3 cycles 0.05% - Copy
Temp: 24.8 °C
MRef: 1.2506 from Mass at end of first 0.0 P/Po stage

	Target % P/Po	Change In Mass (%) - ref Sorption	Desorption	Hysteresis
Cycle 1	8.3	-0.98	3.25	
	12.8	-0.91	3.90	4.81
	21.0	0.18	4.91	4.73
	29.3	1.20	6.23	5.03
	37.8	2.62	7.20	4.57
	46.3	4.37	8.64	4.26
	55.1	6.38	9.44	3.05
	63.6	10.52	12.09	1.57
	72.5	17.14	18.10	0.97
	82.3	28.96	28.96	
Cycle 2	8.3	3.25	4.09	
	12.8	3.07	4.88	1.81
	21.0	3.08	5.91	2.83
	29.3	3.15	6.60	3.45
	37.8	3.45	6.88	3.43
	46.3	3.88	8.72	4.84
	55.1	4.91	9.82	4.91
	63.6	9.14	12.75	3.61
	72.5	16.44	18.46	2.02
	82.3	29.09	29.09	
Cycle 3	8.3	4.09	0.00	
	12.8	3.90	0.12	-3.78
	21.0	3.88	1.22	-2.65
	29.3	3.91	3.06	-0.85
	37.8	4.16	3.51	-0.65
	46.3	4.57	4.51	-0.06
	55.1	2.88	7.42	4.54
	63.6	7.64	9.99	2.34
	72.5	14.16	15.86	1.70
	82.3	27.78	27.78	

DVS from SMS - World leader in Sorption Science

© Surface Measurement Systems Ltd 1996-2018

Figure S75. DVS isotherm analysis report for poly(*β*-Gal-Thr)$_{23}$-*r*-poly(Cys-H)$_3$.

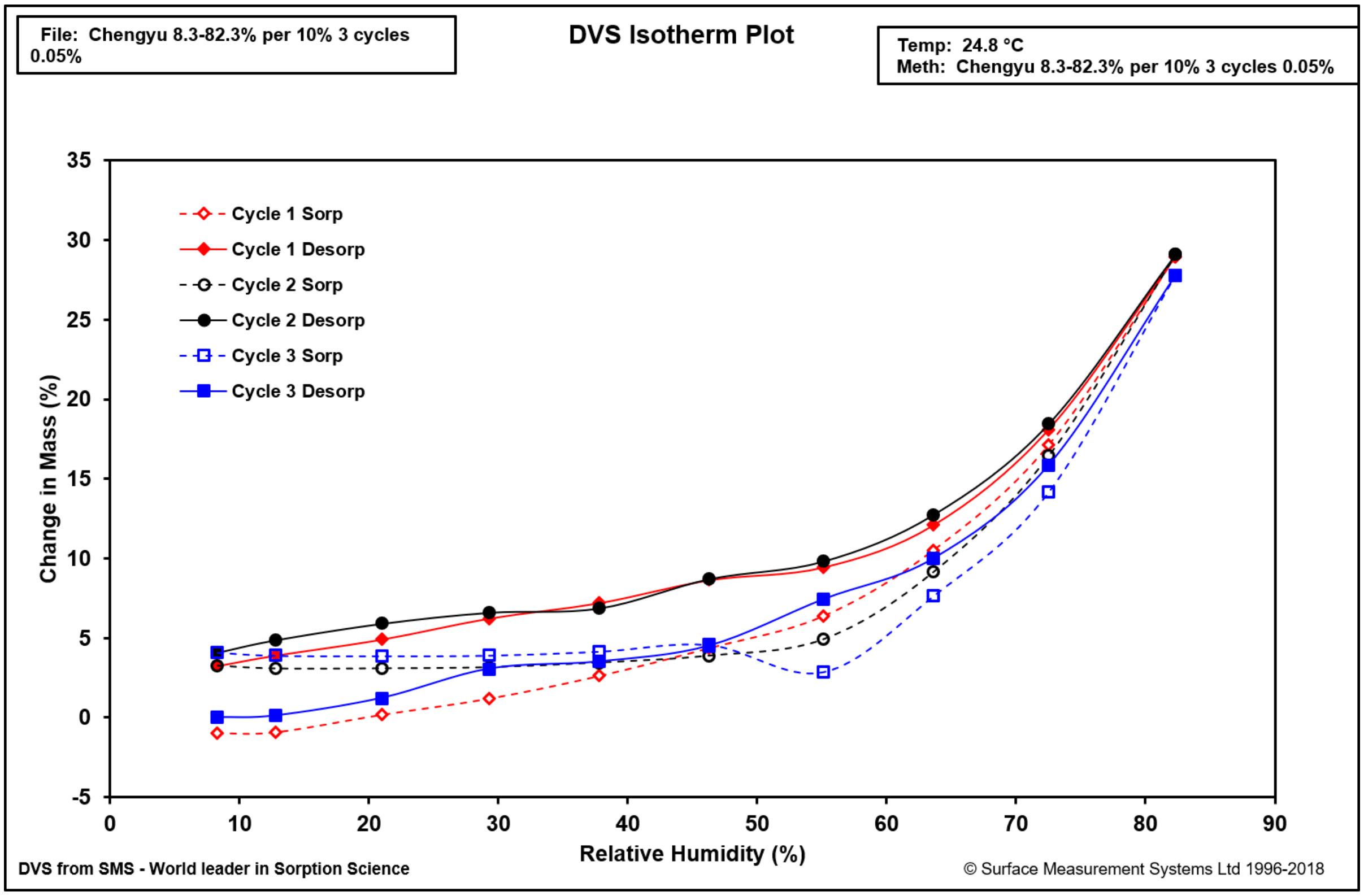

Figure S76. DVS isotherm Plot for poly(*β*-Gal-Thr)$_{23}$-*r*-poly(Cys-H)$_{3}$.

DVS Isotherm Analysis Report

Meth: Chengyu 8.3-82.3% per 10% 3 cycles 0.05%
Temp: 24.8 °C
MRef: 1.8026 from Mass at end of first stage

	Target	Change In Mass (%) - ref		
	% P/Po	Sorption	Desorption	Hysteresis
Cycle 1	8.3	0.14	-0.40	
	12.8	0.27	0.20	-0.07
	21.0	1.05	1.50	0.44
	29.3	1.10	2.75	1.65
	37.8	2.24	3.93	1.69
	46.3	3.78	5.11	1.33
	55.1	5.75	6.63	0.88
	63.6	8.67	9.14	0.47
	72.5	13.30	13.92	0.62
	82.3	20.71	20.71	
Cycle 2	8.3	-0.40	-0.39	
	12.8	-0.36	0.36	0.72
	21.0	0.19	1.61	1.41
	29.3	1.03	2.65	1.62
	37.8	2.13	3.84	1.71
	46.3	3.48	5.22	1.74
	55.1	5.31	6.65	1.34
	63.6	8.27	9.10	0.84
	72.5	12.93	13.89	0.96
	82.3	20.34	20.34	
Cycle 3	8.3	-0.39	0.00	
	12.8	-0.31	0.71	1.02
	21.0	0.25	1.88	1.63
	29.3	1.06	2.87	1.81
	37.8	2.19	3.97	1.78
	46.3	3.34	5.22	1.73
	55.1	5.28	6.65	1.38
	63.6	8.20	9.09	0.89
	72.5	12.88	13.79	0.91
	82.3	20.29	20.29	

DVS from SMS - World leader in Sorption Science

© Surface Measurement Systems Ltd 1996-2018

Figure S77. DVS isotherm analysis report for poly(*β*-Gal-Thr)$_{23}$-*r*-poly(Cys-D)$_3$.

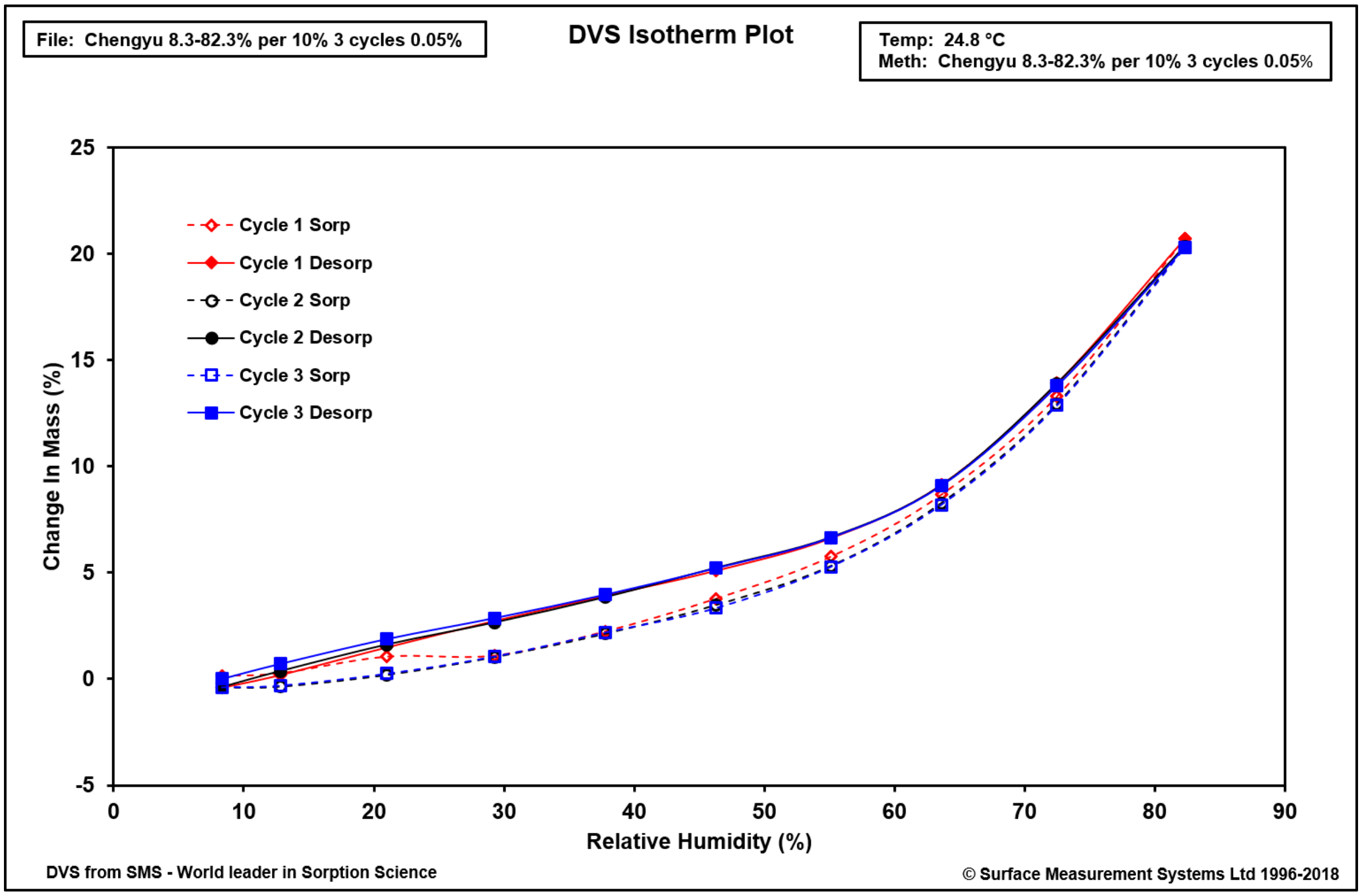

Figure S78. DVS isotherm Plot for poly(β-Gal-Thr)$_{23}$-*r*-poly(Cys-D)$_3$.

DVS Isotherm Analysis Report

Meth: Chengyu 8.3-82.3% per 10% 3 cycles 0.05%
Temp: 24.8 °C
MRef: 0.7814 from Mass at end of first stage

	Target	Change In Mass (%) - ref		
	% P/Po	Sorption	Desorption	Hysteresis
Cycle 1	8.3	1.11	0.72	
	12.8	1.51	1.34	-0.17
	21.0	3.21	1.98	-1.23
	29.3	4.44	3.48	-0.96
	37.8	5.99	4.76	-1.23
	46.3	7.67	7.81	0.14
	55.1	9.85	9.00	-0.86
	63.6	13.85	12.30	-1.55
	72.5	20.00	19.52	-0.49
	82.3	30.64	30.64	
Cycle 2	8.3	0.72	0.76	
	12.8	0.70	1.42	0.72
	21.0	1.23	2.48	1.25
	29.3	1.61	3.35	1.74
	37.8	2.56	4.99	2.43
	46.3	3.70	6.68	3.19
	55.1	5.63	8.72	3.08
	63.6	9.89	12.43	2.53
	72.5	17.26	19.13	1.87
	82.3	29.13	29.13	
Cycle 3	8.3	0.76	0.00	
	12.8	0.55	1.18	0.63
	21.0	0.63	2.67	2.05
	29.3	0.74	3.43	2.69
	37.8	1.38	4.06	2.67
	46.3	2.27	5.64	3.38
	55.1	3.90	7.46	3.56
	63.6	8.11	10.67	2.56
	72.5	15.59	17.32	1.73
	82.3	27.16	27.16	

DVS from SMS - World leader in Sorption Science

© Surface Measurement Systems Ltd 1996-2018

Figure S79. DVS isotherm analysis report for poly(*β*-Gal-Thr)$_{23}$-*r*-poly(Cys-H)$_6$.

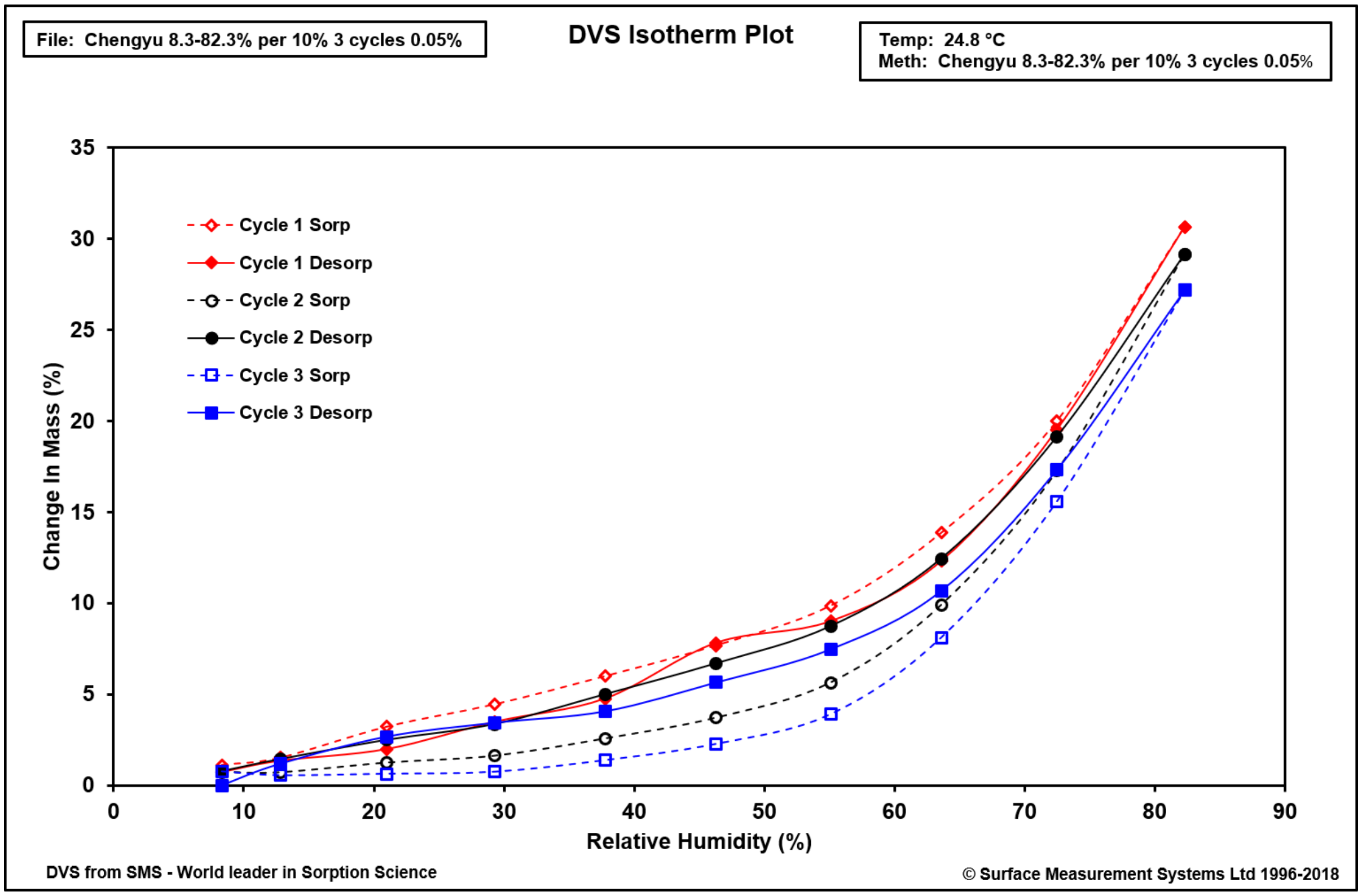

Figure S80. DVS isotherm Plot for poly(β-Gal-Thr)$_{23}$-*r*-poly(Cys-H)$_6$.

DVS Isotherm Analysis Report

Meth: Chengyu 8.3-82.3% per 10% 3 cycles 0.05% 5-20-5
Temp: 24.9 °C
MRef: 1.8026 from Mass at end of first 0.0 P/Po stage

	Target % P/Po	Change In Mass (%) - ref Sorption	Desorption	Hysteresis
Cycle 1	8.3	0.14	-0.40	
	12.8	0.27	0.20	0.07
	21.0	1.05	1.50	-0.44
	29.3	1.10	2.75	-1.65
	37.8	2.24	3.93	-1.69
	46.3	3.78	5.11	-1.33
	55.1	5.75	6.93	-1.18
	63.6	8.61	9.15	-0.55
	72.5	14.30	14.92	-0.62
	82.3	18.71	18.71	
Cycle 2	8.3	-0.50	-0.16	
	12.8	-0.41	0.49	-0.90
	21.0	0.10	1.46	-1.36
	29.3	0.78	2.32	-1.54
	37.8	2.72	3.36	-0.64
	46.3	2.98	4.39	-1.41
	55.1	4.64	5.79	-1.15
	63.6	7.43	8.06	-0.63
	72.5	11.82	12.52	-0.69
	82.3	18.74	18.74	
Cycle 3	8.3	-0.39	0.00	
	12.8	-0.51	0.71	-1.22
	21.0	0.25	1.88	-1.63
	29.3	2.06	2.87	-0.81
	37.8	2.19	3.97	-1.78
	46.3	3.49	5.22	-1.73
	55.1	5.28	6.65	-1.38
	63.6	8.20	9.08	-0.89
	72.5	12.88	13.79	-0.91
	82.3	18.29	18.29	

DVS from SMS - World leader in Sorption Science

© Surface Measurement Systems Ltd 1996-2018

Figure S81. DVS isotherm analysis report for poly(β-Gal-Thr)$_{23}$-*r*-poly(Cys-D)$_{6}$.

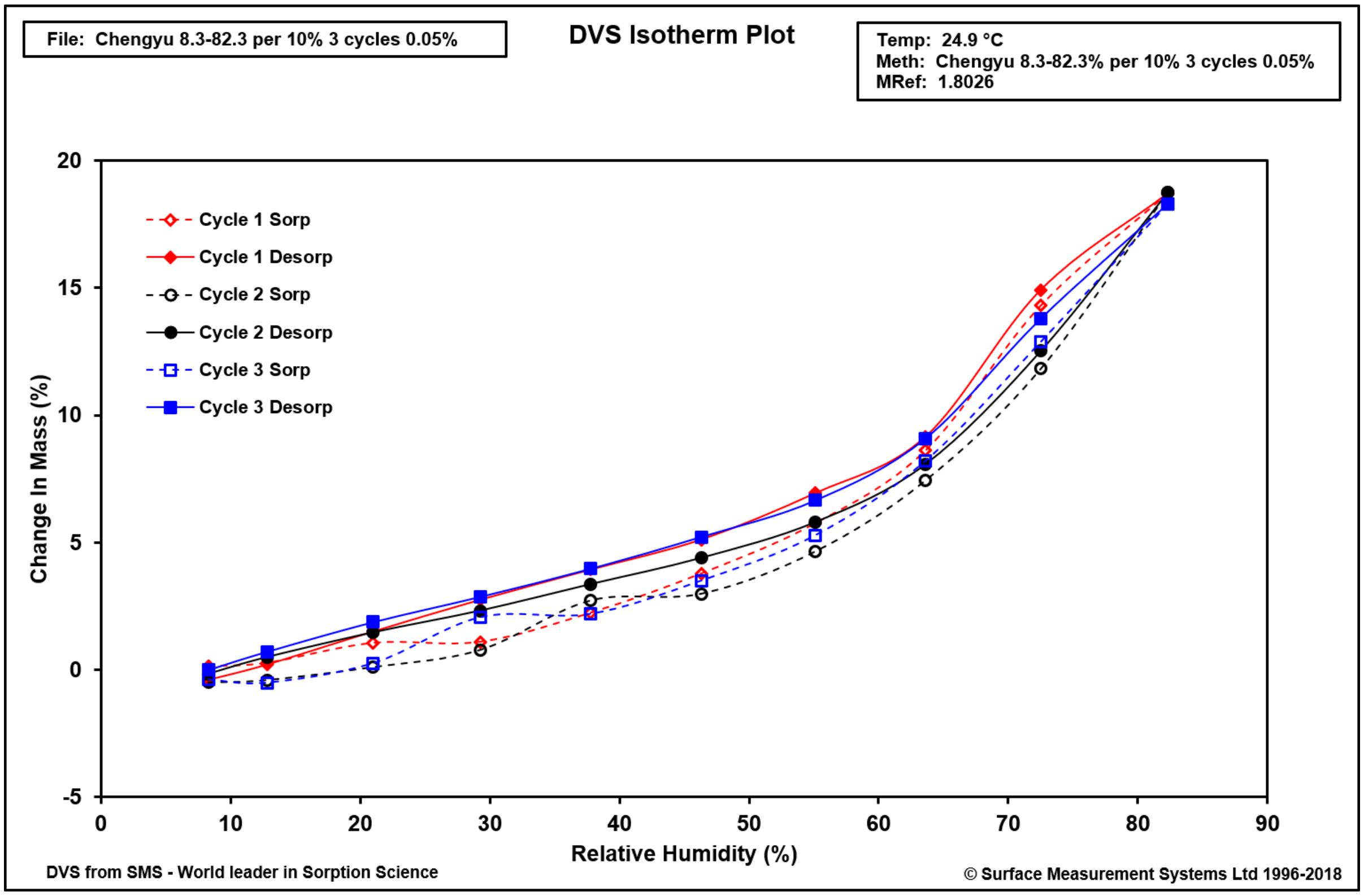

Figure S82. DVS isotherm Plot for poly(β-Gal-Thr)$_{23}$-*r*-poly(Cys-D)$_6$.

DVS Isotherm Analysis Report

Meth: 8.3-82.3% per 10% 3 cycles 0.05%
Temp: 24.8 °C
MRef: 1.0404 from Mass at end of first stage

	Target	Change In Mass (%) - ref		
	% P/Po	Sorption	Desorption	Hysteresis
Cycle 1	8.3	0.00	-0.78	
	12.8	0.03	-0.24	-0.27
	21.0	0.77	0.56	-0.21
	29.3	1.68	2.60	0.92
	37.8	2.98	4.18	1.20
	46.3	6.22	6.65	0.43
	55.1	9.63	10.45	0.82
	63.6	14.44	16.20	1.76
	72.5	22.66	25.82	3.15
	82.3	36.45	36.45	
Cycle 2	8.3	-0.78	-2.04	
	12.8	-0.60	-1.54	-0.94
	21.0	-1.47	-0.07	1.40
	29.3	0.64	1.23	0.59
	37.8	2.44	2.85	0.41
	46.3	5.01	5.46	0.45
	55.1	8.69	9.24	0.55
	63.6	14.03	14.84	0.81
	72.5	22.25	24.51	2.26
	82.3	35.22	35.22	
Cycle 3	8.3	-2.04	-1.81	
	12.8	-1.85	-1.05	0.80
	21.0	-0.76	0.37	1.13
	29.3	0.55	1.72	1.17
	37.8	2.34	3.27	0.93
	46.3	4.82	5.57	0.76
	55.1	8.71	9.50	0.79
	63.6	14.55	15.21	0.65
	72.5	22.71	24.77	2.06
	82.3	35.59	35.59	

DVS from SMS - World leader in Sorption Science

© Surface Measurement Systems Ltd 1996-2018

Figure S83. DVS isotherm analysis report for poly(β-Gal-Thr)$_{33}$-*r*-poly(Cys-H)$_3$.

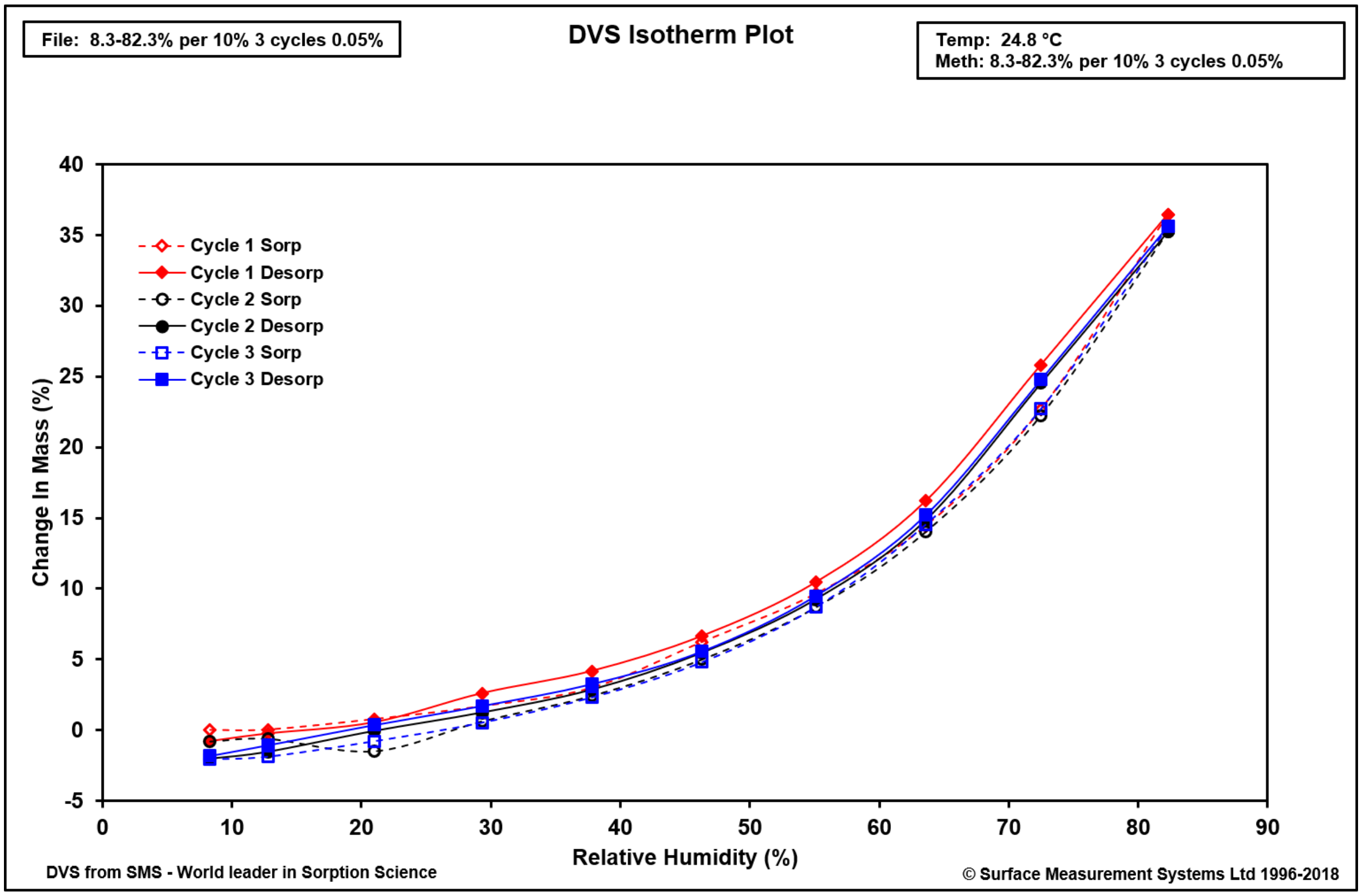

Figure S84. DVS isotherm Plot for poly(β-Gal-Thr)$_{33}$-*r*-poly(Cys-H)$_3$.

DVS Isotherm Analysis Report

Meth: Chengyu 8.3-82.3% per 10% 3 cycles 0.05%
Temp: 24.8 °C
MRef: 1.5503 from Mass at end of first stage

	Target	Change In Mass (%) - ref		
	% P/Po	Sorption	Desorption	Hysteresis
Cycle 1	8.3	0.00	-1.25	
	12.8	-0.07	-0.32	-0.25
	21.0	0.28	1.28	1.00
	29.3	1.01	2.56	1.55
	37.8	1.75	4.13	2.38
	46.3	3.42	5.19	1.77
	55.1	5.59	7.04	1.44
	63.6	8.68	9.93	1.26
	72.5	13.85	15.49	1.64
	82.3	22.51	22.51	
Cycle 2	8.3	-1.25	-1.93	
	12.8	-1.36	-0.89	0.47
	21.0	-0.94	0.88	1.82
	29.3	-0.43	2.32	2.74
	37.8	0.45	3.52	3.08
	46.3	1.95	4.74	2.79
	55.1	4.10	6.33	2.23
	63.6	7.32	8.62	1.30
	72.5	12.80	14.06	1.25
	82.3	21.17	21.17	
Cycle 3	8.3	-1.93	-0.68	
	12.8	-1.72	-0.01	1.71
	21.0	-1.01	1.12	2.13
	29.3	-0.04	2.23	2.27
	37.8	0.50	3.56	3.06
	46.3	2.27	5.06	2.79
	55.1	5.39	6.81	1.43
	63.6	8.80	9.51	0.71
	72.5	13.78	15.13	1.35
	82.3	22.28	22.28	

DVS from SMS - World leader in Sorption Science

© Surface Measurement Systems Ltd 1996-2018

Figure S85. DVS isotherm analysis report for poly(*β*-Gal-Thr)$_{33}$-*r*-poly(Cys-D)$_3$.

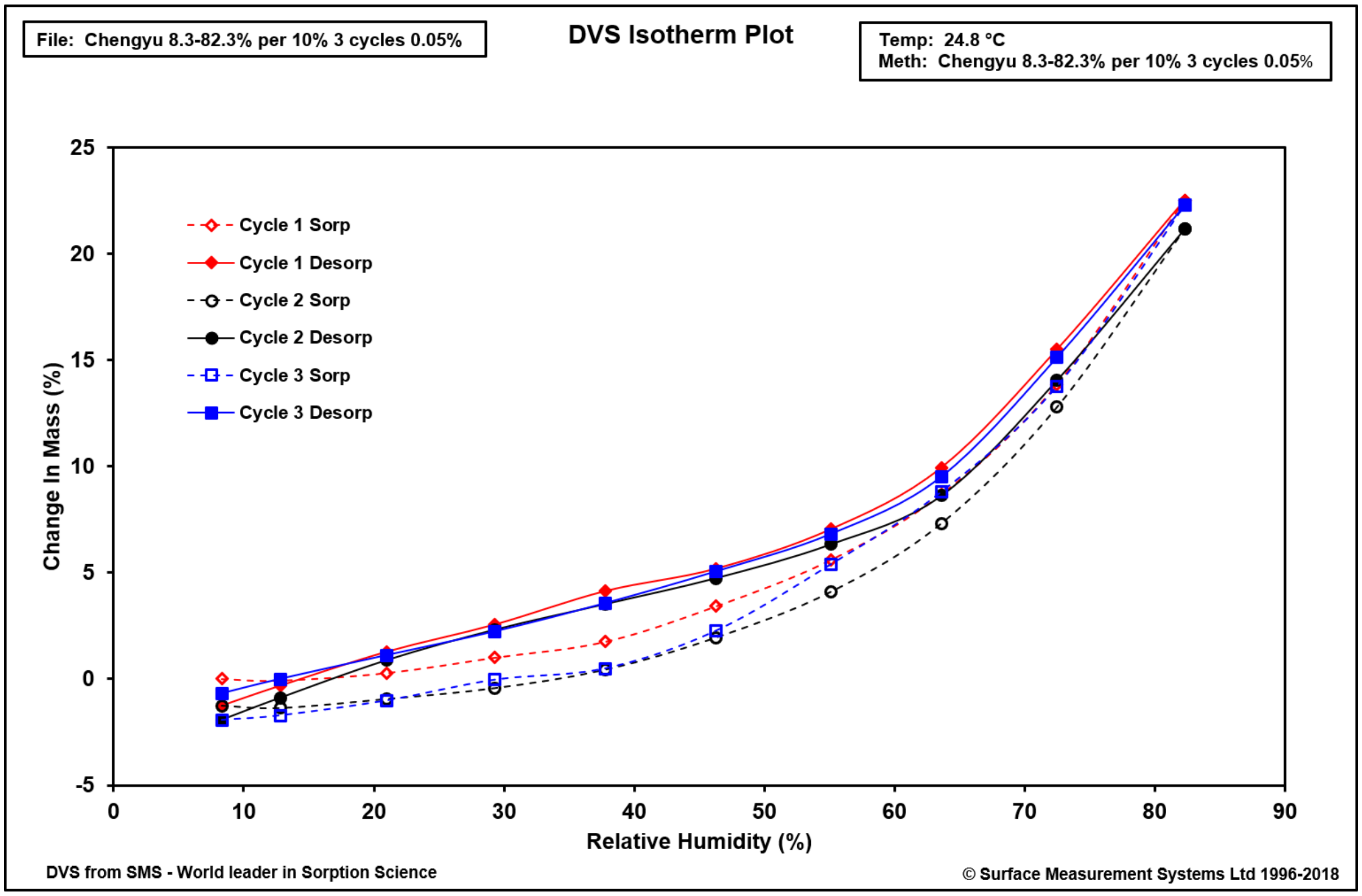

Figure S86. DVS isotherm Plot for poly(β-Gal-Thr)$_{33}$-*r*-poly(Cys-D)$_3$.

DVS Isotherm Analysis Report

Meth: Chengyu 8-82% per 10% 3 cycles 0.05%
Temp: 24.9 °C
MRef: 1.2997 from Mass at end of first stage

	Target % P/Po	Change In Mass (%) - ref Sorption	Desorption	Hysteresis
Cycle 1	0.0	0.00		
	8.3	-4.23	-5.59	-1.36
	12.8	-4.49	-5.23	-0.74
	21.0	-4.08	-4.08	0.00
	29.3	-2.80	5.26	8.06
	37.8	0.85	10.16	9.31
	46.3	12.16	15.63	3.48
	55.1	21.30	22.84	1.54
	63.6	29.71	34.74	5.03
	72.5	42.55	53.73	11.18
	82.3	64.01	64.01	
Cycle 2	8.3	-5.59	-5.32	
	12.8	-5.38	-4.93	0.45
	21.0	-4.17	-3.85	0.32
	29.3	-2.98	5.02	8.00
	37.8	1.70	9.84	8.14
	46.3	13.80	15.13	1.33
	55.1	20.12	22.11	1.99
	63.6	28.39	33.85	5.46
	72.5	41.14	52.21	11.07
	82.3	62.62	62.62	
Cycle 3	8.3	-5.32	-5.06	
	12.8	-5.05	-4.75	0.29
	21.0	-3.92	-3.47	0.45
	29.3	-2.43	5.18	7.61
	37.8	2.25	10.08	7.82
	46.3	13.97	15.35	1.38
	55.1	20.07	22.25	2.19
	63.6	28.31	33.63	5.32
	72.5	40.98	51.80	10.82
	82.3	62.36	62.36	

DVS from SMS - World leader in Sorption Science

© Surface Measurement Systems Ltd 1996-2018

Figure S87. DVS isotherm analysis report for poly(β-Gal-Thr)$_{62}$-*r*-poly(Cys-H)$_3$.

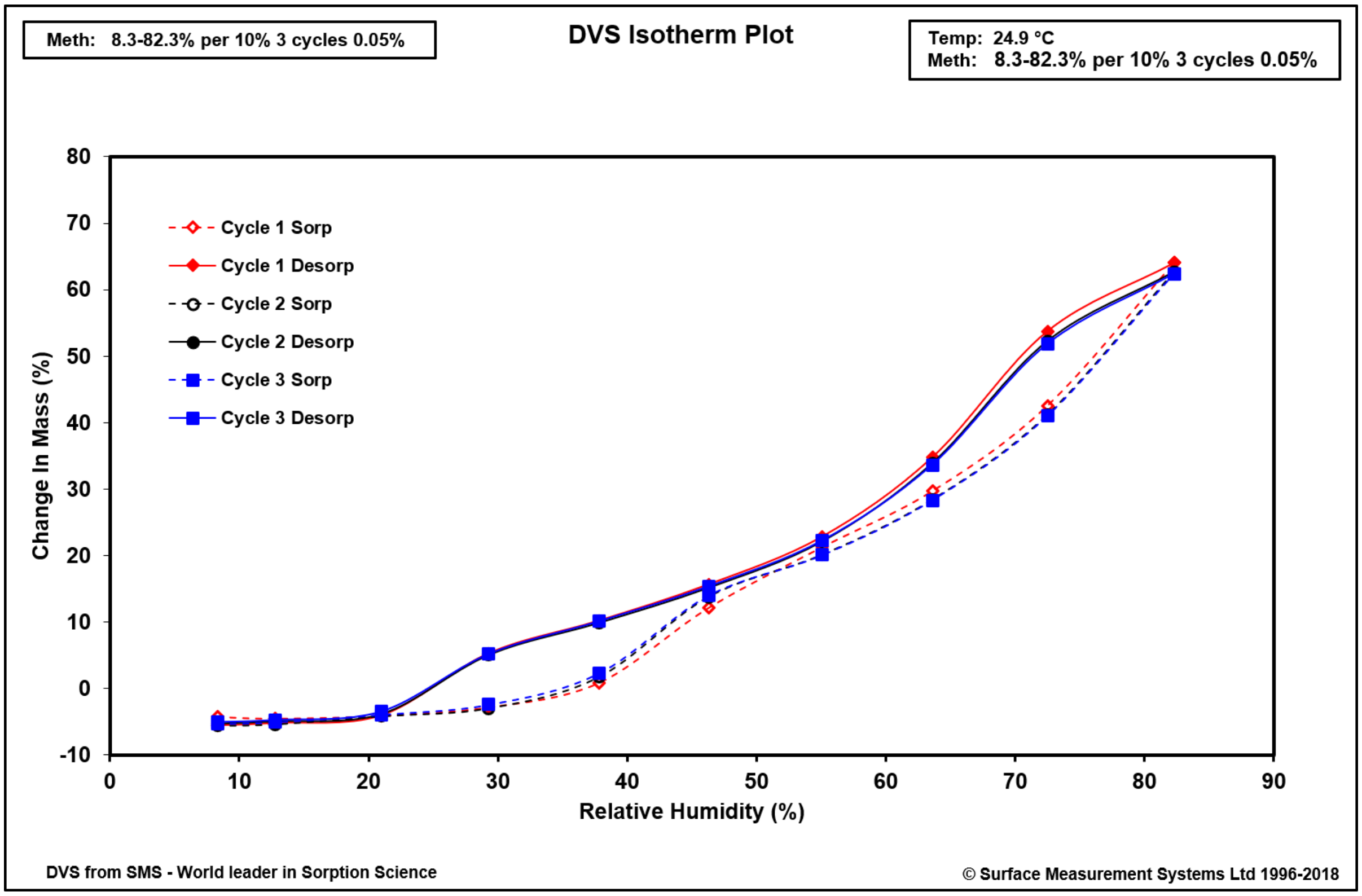

Figure S88. DVS isotherm Plot for poly(β-Gal-Thr)$_{62}$-*r*-poly(Cys-H)$_3$.

DVS Isotherm Analysis Report

Meth: Chengyu 8.3-82.3% per 10% 3 cycles 0.05%
Temp: 24.9 °C

	Target % P/Po	Change In Mass (%) - ref Sorption	Desorption	Hysteresis
Cycle 1	8.3	0.000	-1.840	
	12.8	0.125	-1.483	-1.609
	21.0	0.587	-0.725	-1.312
	29.3	0.389	-0.079	-0.468
	37.8	1.029	0.336	-0.692
	46.3	1.424	0.758	-0.666
	55.1	1.457	1.391	-0.066
	63.6	2.545	2.130	-0.415
	72.5	4.167	4.088	-0.079
	82.3	7.384	7.384	
Cycle 2	8.3	-1.840	-2.505	
	12.8	-1.325	-2.281	-0.956
	21.0	-1.352	-1.800	-0.448
	29.3	-0.976	-1.286	-0.310
	37.8	-0.521	-0.666	-0.145
	46.3	0.073	0.066	-0.007
	55.1	0.607	0.640	0.033
	63.6	1.418	1.424	0.007
	72.5	2.861	3.185	0.323
	82.3	6.191	6.191	
Cycle 3	8.3	-2.505	-3.059	
	12.8	-2.242	-2.809	-0.567
	21.0	-1.497	-1.866	-0.369
	29.3	-0.936	-1.101	-0.165
	37.8	-0.646	-0.574	0.073
	46.3	-0.422	-0.059	0.363
	55.1	0.053	0.877	0.824
	63.6	0.547	1.451	0.903
	72.5	2.532	3.316	0.785
	82.3	5.736	5.736	

DVS from SMS - World leader in Sorption Science © Surface Measurement Systems Ltd 1996-2018

Figure S89. DVS isotherm analysis report for poly(β-Gal-Thr)$_{62}$-*r*-poly(Cys-D)$_3$.

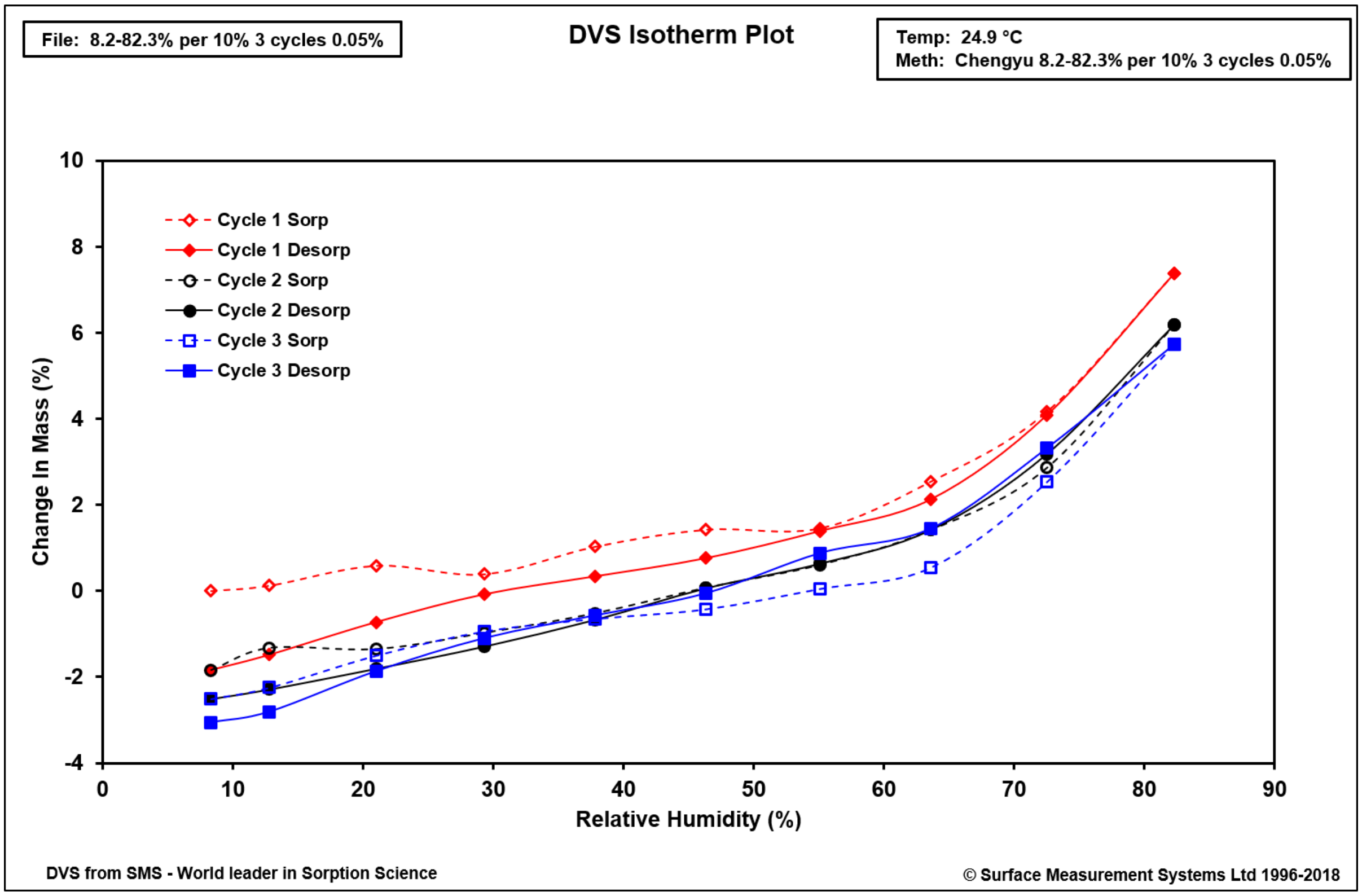

Figure S90. DVS isotherm Plot for poly(β-Gal-Thr)$_{62}$-*r*-poly(Cys-D)$_3$.

DVS Isotherm Analysis Report

Meth: Chengyu 8.3-82.3% per 10% 3 cycles 0.05%
Temp: 24.8 °C
MRef: 2.4795 from Mass at end of first 0.0 P/Po stage

	Target % P/Po	Change In Mass (%) - ref Sorption	Desorption	Hysteresis
Cycle	8.3	3.1	-0.4	
	12.8	3.4	0.1	-3.3
	21.0	4.1	1.5	-2.7
	29.3	5.3	4.0	-1.3
	37.8	7.5	20.7	13.2
	46.3	19.8	27.7	7.9
	55.1	23.9	36.8	12.9
	63.6	30.5	50.2	19.7
	72.5	44.2	69.4	25.2
	82.3	101.4	101.4	
Cycle	8.3	-0.4	0.1	
	12.8	0.0	0.6	0.6
	21.0	1.1	1.9	0.8
	29.3	2.7	4.2	1.6
	37.8	8.7	21.2	12.5
	46.3	22.1	28.3	6.2
	55.1	30.0	37.4	7.4
	63.6	39.2	50.8	11.5
	72.5	57.1	69.6	12.5
	82.3	101.0	101.0	
Cycle	8.3	0.1	0.0	
	12.8	0.4	0.5	0.1
	21.0	1.6	1.9	0.2
	29.3	3.2	4.3	1.1
	37.8	9.4	21.3	11.9
	46.3	22.5	28.4	5.9
	55.1	30.7	37.5	6.8
	63.6	39.9	51.0	11.1
	72.5	57.7	69.9	12.2
	82.3	101.4	101.4	

DVS from SMS - World leader in Sorption Science © Surface Measurement Systems Ltd 1996-2018

Figure S91. DVS isotherm analysis report for poly(β-Gal-Thr)$_{78}$-*r*-poly(Cys-H)$_4$.

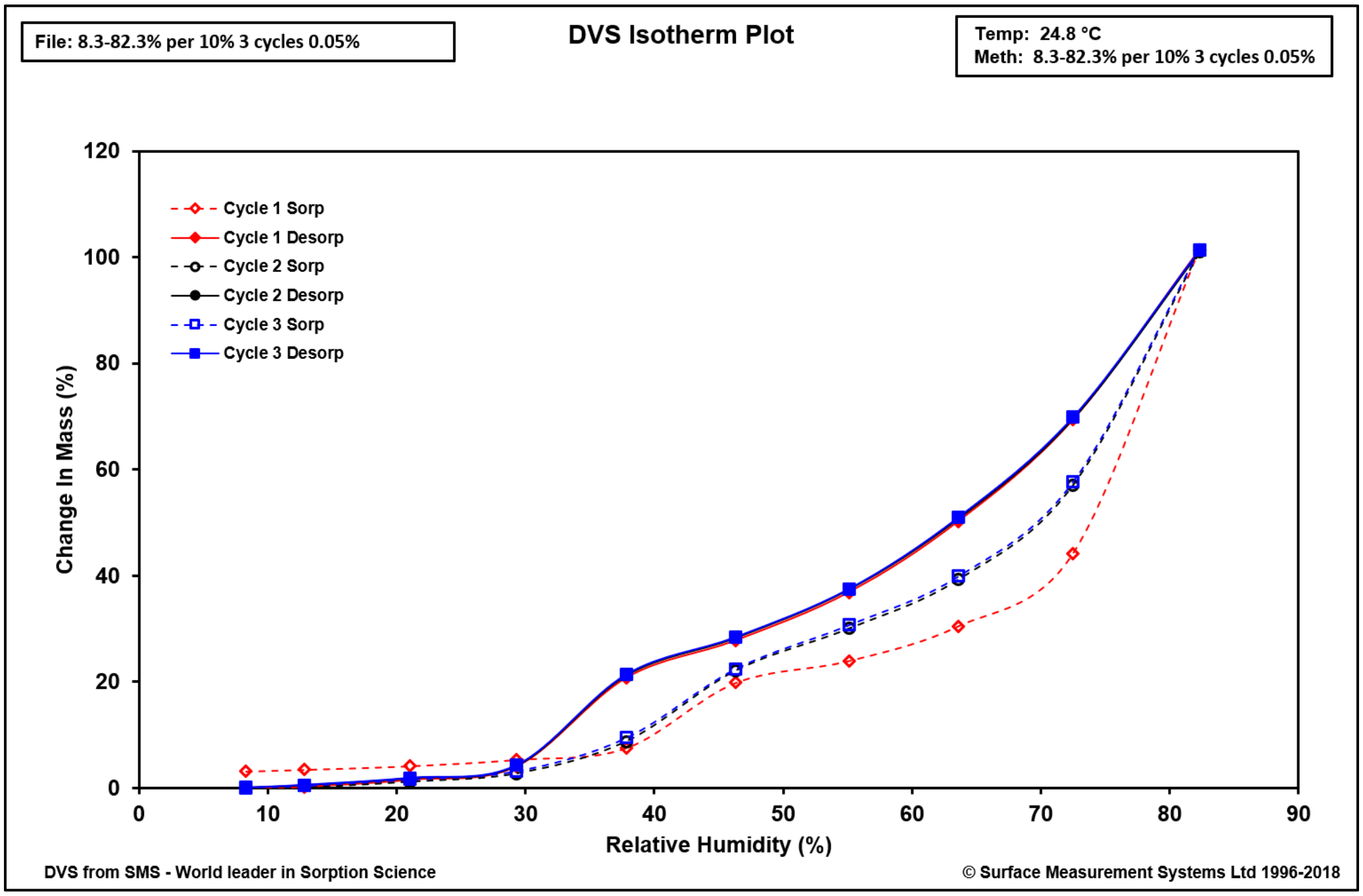

Figure S92. DVS isotherm plot for poly(β-Gal-Thr)$_{78}$-*r*-poly(Cys-H)$_4$.

DVS Isotherm Analysis Report

Meth: Chengyu 8.3-82.3% per 10% 2 cycles 0.05%
Temp: 24.8 °C
MRef: 3.4893 from Mass at end of first stage

	Target	Change In Mass (%) - ref		
	% P/Po	Sorption	Desorption	Hysteresis
Cycle 1	8.3	0.000	0.980	
	12.8	0.003	0.917	-0.914
	21.0	0.092	0.797	-0.705
	29.3	0.060	0.714	-0.653
	37.8	0.155	0.433	-0.278
	46.3	0.433	0.848	-0.416
	55.1	0.496	0.493	0.003
	63.6	0.054	0.691	-0.636
	72.5	0.160	0.100	-0.060
	82.3	0.387	0.387	
Cycle 2	8.3	0.980	1.098	
	12.8	1.017	1.052	-0.034
	21.0	1.003	1.020	-0.017
	29.3	0.992	1.003	-0.011
	37.8	0.934	0.969	-0.034
	46.3	0.874	0.900	-0.026
	55.1	0.820	0.834	-0.014
	63.6	0.676	0.739	-0.063
	72.5	0.559	0.585	-0.026
	82.3	0.332	0.332	

DVS from SMS - World leader in Sorption Science

© Surface Measurement Systems Ltd 1996-2018

Figure S93. DVS isotherm analysis report for Poly(β-Gal-Thr)$_{78}$-*r*-poly(Cys-D)$_4$

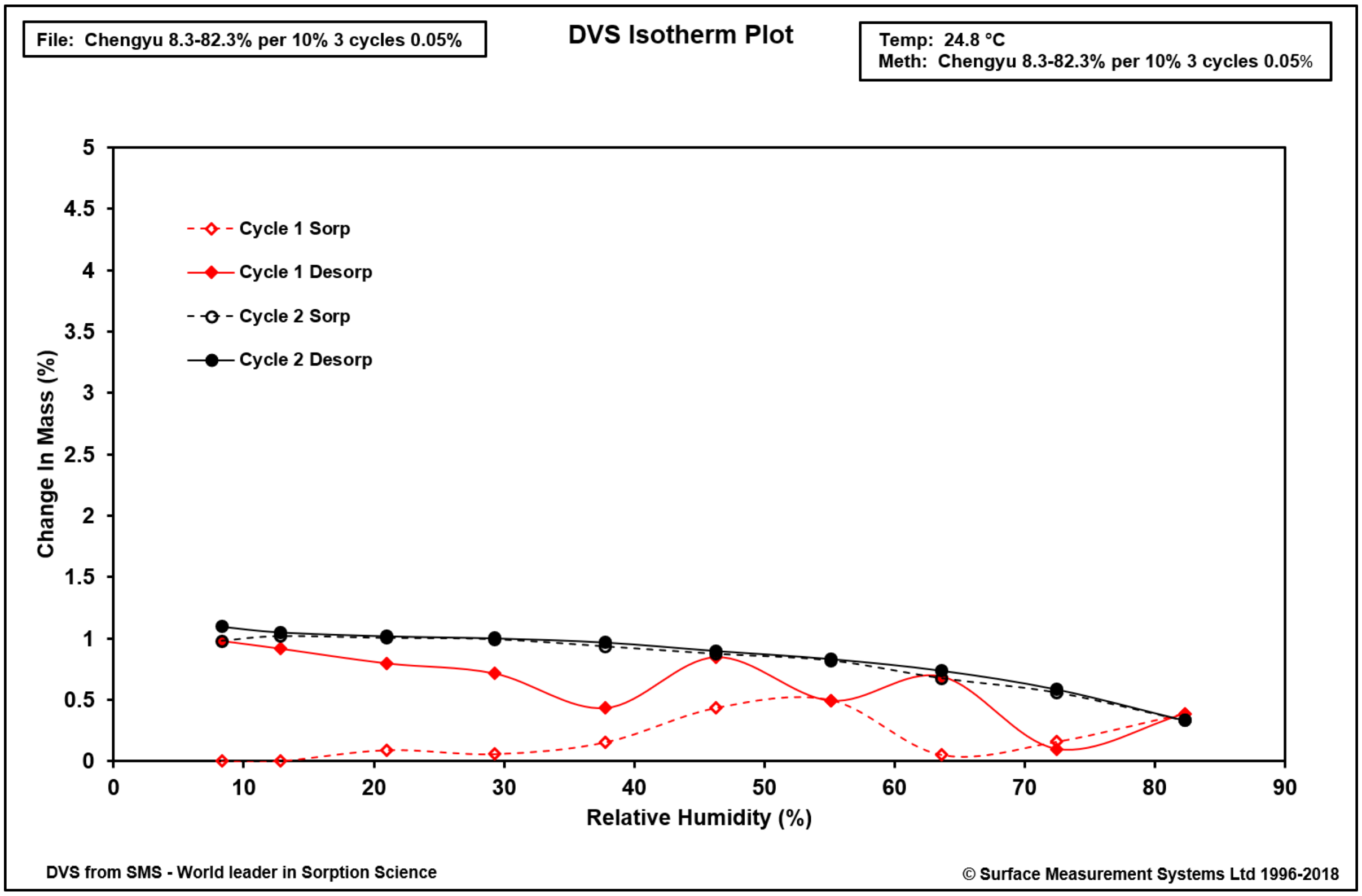

Figure S94. DVS isotherm Plot for poly(*β*-Gal-Thr)$_{78}$-*r*-poly(Cys-D)$_4$.

DVS Isotherm Analysis Report

Meth: Chengyu 8.3-82.3% per 10% 3 cycles 0.05%
Temp: 24.8 °C
MRef: 4.1472 from Mass at end of first stage

	Target	Change In Mass (%) - ref		
	% P/Po	Sorption	Desorption	Hysteresis
Cycle 1	8.3	0.00	-0.73	
	12.8	0.18	-0.33	-0.51
	21.0	0.78	0.86	0.07
	29.3	1.86	2.28	0.42
	37.8	3.03	3.99	0.96
	46.3	4.79	6.56	1.76
	55.1	7.57	10.53	2.96
	63.6	11.29	16.85	5.56
	72.5	17.04	24.12	7.09
	82.3	26.63	26.63	
Cycle 2	8.3	-0.73	-0.64	
	12.8	-0.52	-0.18	0.34
	21.0	0.52	1.05	0.53
	29.3	1.80	2.53	0.74
	37.8	3.34	4.32	0.97
	46.3	5.50	7.13	1.62
	55.1	8.60	11.41	2.81
	63.6	12.83	18.30	5.47
	72.5	19.05	26.24	7.19
	82.3	28.75	28.75	
Cycle 3	8.3	-0.64	-0.32	
	12.8	-0.33	0.35	0.68
	21.0	0.89	1.38	0.49
	29.3	2.28	3.02	0.74
	37.8	4.28	5.14	0.86
	46.3	6.68	8.13	1.45
	55.1	9.98	12.59	2.61
	63.6	14.42	19.65	5.24
	72.5	20.78	27.73	6.95
	82.3	30.49	30.49	

DVS from SMS - World leader in Sorption Science

© Surface Measurement Systems Ltd 1996-2018

Figure S95. DVS isotherm analysis report for Reversable poly(β-Gal-Thr)$_{23}$-*r*-poly(Cys-H)$_6$.

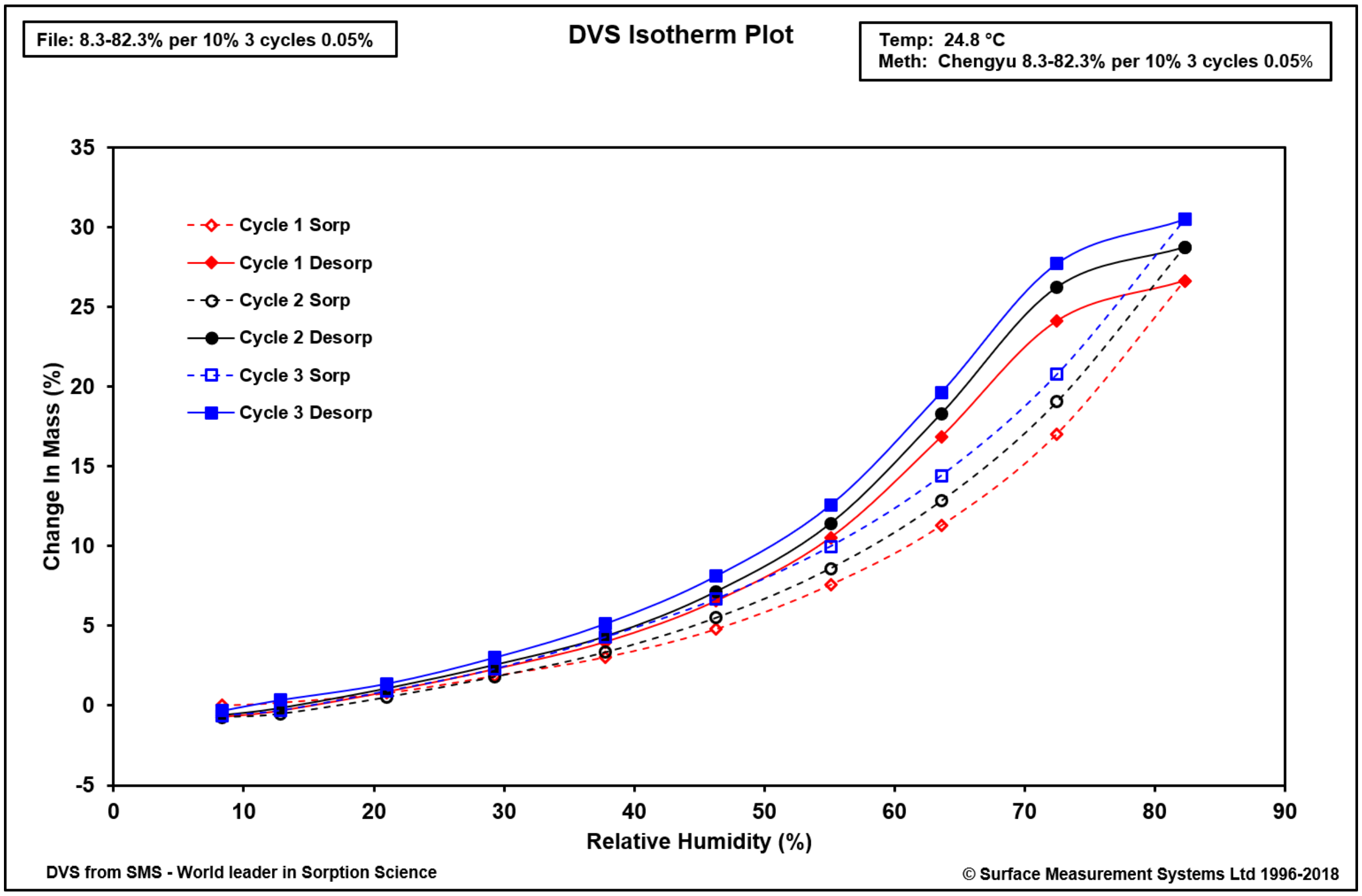

Figure S96. DVS isotherm Plot for Reversable poly(*β*-Gal-Thr)$_{23}$-*r*-poly(Cys-H)$_6$.

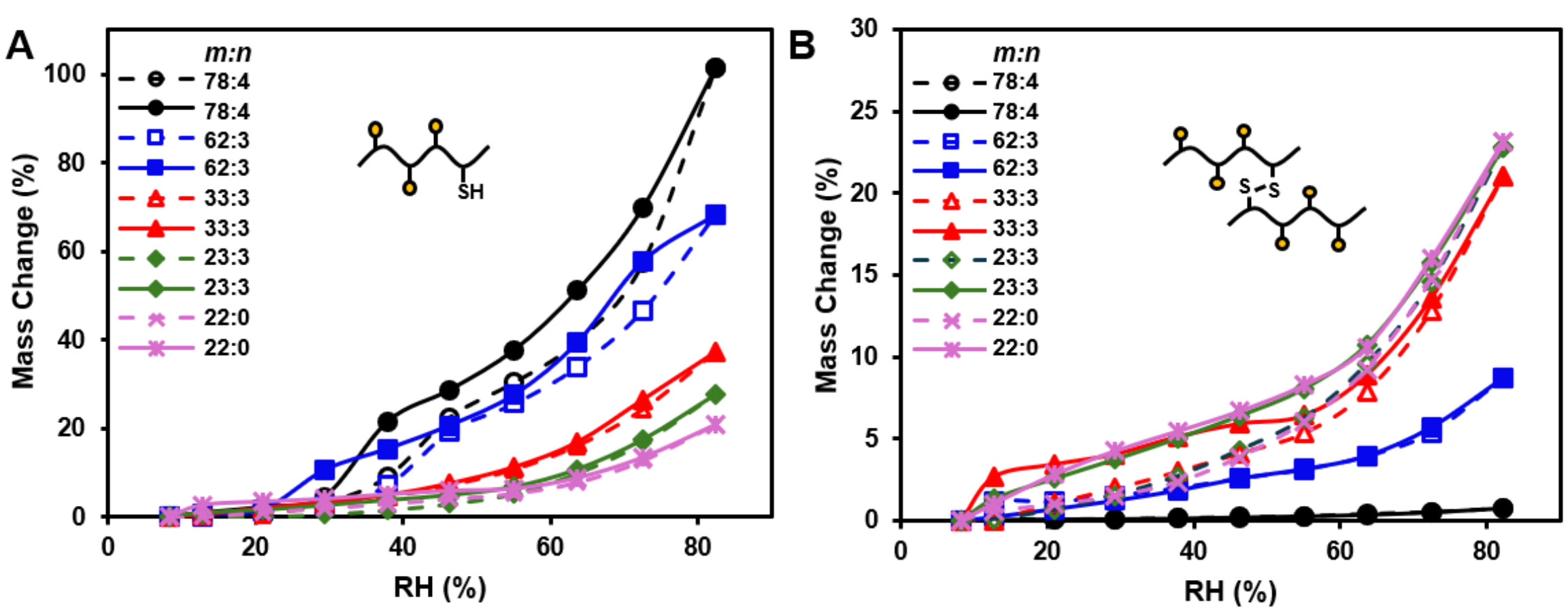

Figure S97. DVS and SEM analysis of SCAMPs. (A-B) Water sorption isotherms of poly(*β*-Gal-Thr)$_m$-*r*-poly(Cys-H)$_n$ and Poly(*β*-Gal-Thr)$_m$-*r*-poly(Cys-D)$_n$. The dashed lines represent adsorption and the solid lines represent desorption.

6. Determination of Young's Modulus (*E*) and effective Work of Adhesion (W) of Polymers

Determination of *E*. The *E* of polymer samples was characterized by atomic force microscopy (AFM) nanoindentation using a customized Multimode 8 AFM (Bruker) operated at 25 °C and 50% RH. Prior to AFM measurements, 2 μL polymer solution (20 mg/mL) was drop-cast onto a silicon wafer and dried overnight at room temperature. The local RH was precisely controlled by adjusting the flow rates of humidified and dry air and was allowed to stabilize at the target value for at least 10 min before measurements. The RH was continuously monitored using a HIH-4021 humidity sensor (Honeywell). AFM nanoindentation measurements were subsequently performed using NCHV probe (Bruker) with a calibrated spring constant of 55.3 N/m. Force–distance curves were acquired at an indentation rate of 1 Hz. For each location, indentation measurements were repeated three times to ensure reproducibility, and at least three different locations were tested for each sample.[9, 10]

The force–distance curves were analyzed using a Hertzian contact model to extract the *E* of the polymer (Eq. 2).

$$F^{\frac{2}{3}} = \left(\frac{4}{3}\frac{E}{(1-v)^2}\sqrt{R}\right)^{\frac{2}{3}} d \qquad \text{(Eq. 2)}$$

where *F* is the indentation force, *v* is the Poisson's ratio (0.3), R is the probe radius (8 nm), and *d* is the indentation depth.[11, 12]

Determination of W. *W* was determined from the area enclosed by the approach and retraction curves in the force–distance curves, and then normalized by the corresponding tip–sample contact area (A) to account for variations in indentation depth and contact geometry (Eq. 3).

$$W = \frac{\oint F(d)\mathrm{d}d}{A} \qquad \text{(Eq. 3)}$$

where $\oint F(d)\,\mathrm{d}d$ is the enclosed area of the force–distance curve, corresponding to the mechanical energy dissipated during one loading–unloading cycle. The A was approximated using the maximum Hertzian contact during loading, $A = \pi R \delta_{\max}$, where R is the tip radius and $\delta_{\max}$ is the maximum indentation depth. The *W* shows a dissipative, nonequilibrium work of adhesion rather than a purely thermodynamic surface energy. It captures energy losses arising from viscoelastic dissipation within the polymer–water network, as well as additional contributions from kinetically limited bond rupture and molecular rearrangements.[13-15]

Table 2: *E* and *W* of poly(β-Gal-Thr)$_m$-*r*-poly(Cys-H)$_n$ and poly(β-Gal-Thr)$_m$-*r*-poly(Cys-D)$_n$ at 50% RH.

	SH					SS				
m:n	DVS 82 % RH	Hysteresis[a] at 38 % RH	Hysteresis[a] 72 % RH	*E*[b] (GPa)	*W*[b] (J/m^2)	DVS 82 % RH	Hysteresis[a] at 38 % RH	Hysteresis[a] at 72 % RH	*E*[b] (GPa)	*W*[b] (J/m^2)
22:0	22.7	2.11	0.750	4.70	2.34	22.7	2.11	0.750	4.70	2.34
23:1	25.4	3.43	2.01	0.730	20.3	21.7	1.58	1.21	6.67	6.18
23:2	27.7	2.43	1.86	0.470	2.20	22.5	2.32	1.17	2.20	1.20
23:3	29.0	2.37	0.807	1.76	0.500	20.3	1.70	0.959	2.75	1.80
23:6	29.1	1.75	1.88	0.400	0.880	18.7	1.56	0.694	6.60	12.0
33:3	35.2	0.413	2.26	1.10	13.7	21.2	3.07	1.25	2.86	7.30
62:3	62.6	8.14	11.1	7.10	15.0	6.19	0.145	0.324	7.50	3.20
78:4	101	12.5	12.5	0.870	9.80	0.332	0.034	0.026	4.80	1.52
23:6	28.8[c]	0.972[c]	7.47[c]							

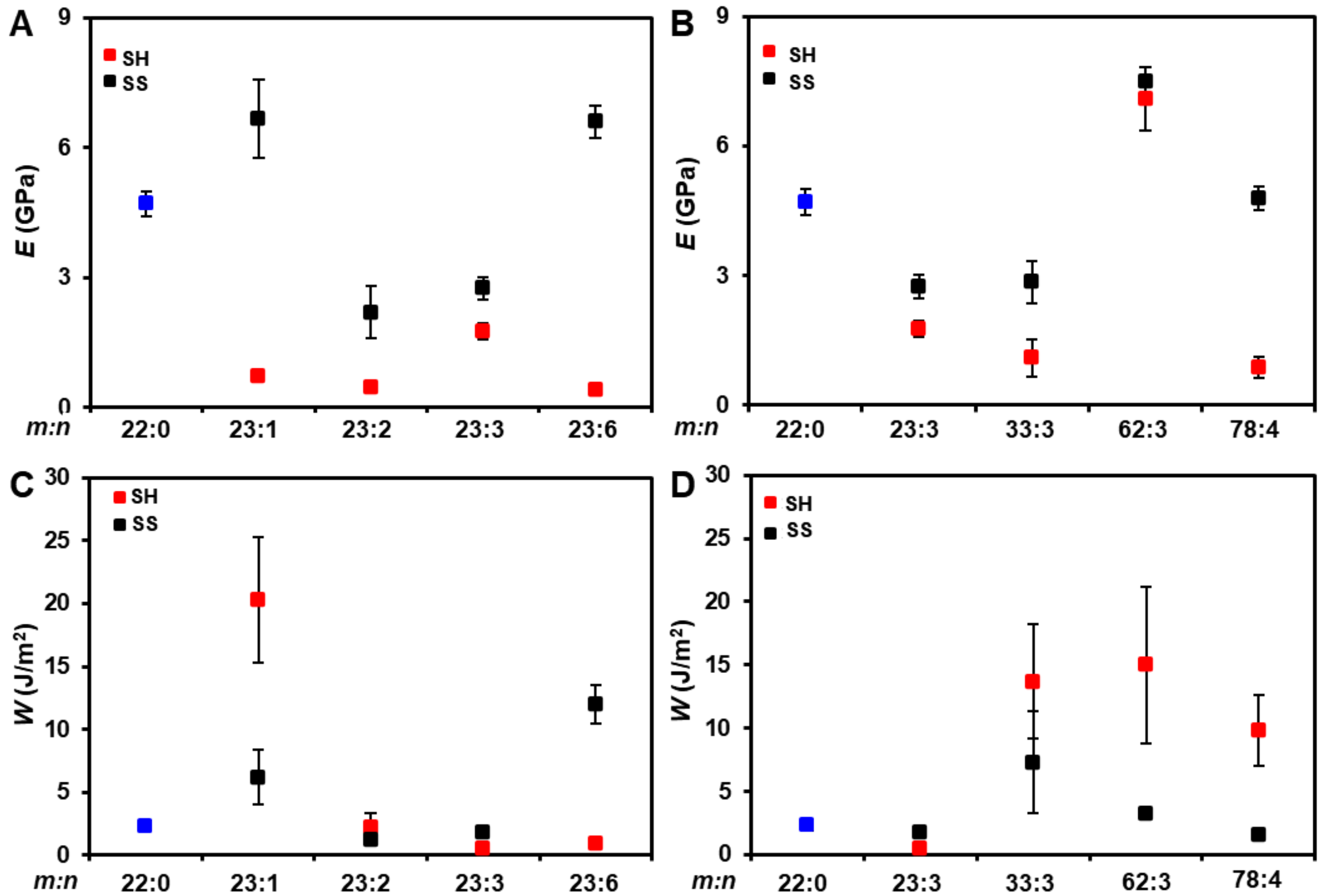

Figure S98. *E* and *W* of poly(β-Gal-Thr)$_m$-*r*-poly(Cys-H)$_n$ and poly(β-Gal-Thr)$_m$-*r*-poly(Cys-D)$_n$ at 50% RH. (A) *E* for poly(β-Gal-Thr)$_m$-*r*-poly(Cys-H)$_n$ (red) and poly(β-Gal-Thr)$_m$-*r*-poly(Cys-D)$_n$ (black) with constant *m*, varying *n* and poly(β-Gal-Thr)$_{22}$ (blue). (B) *E* for poly(β-Gal-Thr)$_m$-*r*-poly(Cys-H)$_n$ (red) and poly(β-Gal-Thr)$_m$-*r*-poly(Cys-D)$_n$ (black) with constant *n*, varying *m* and poly(β-Gal-Thr)$_{22}$ (blue). (C) *W* for poly(β-Gal-Thr)$_m$-*r*-poly(Cys-H)$_n$ (red) and poly(β-Gal-Thr)$_m$-*r*-poly(Cys-D)$_n$ (black) with constant *m*, varying *n* and poly(β-Gal-Thr)$_{22}$ (blue). (D) *W* for poly(β-Gal-Thr)$_m$-*r*-poly(Cys-H)$_n$ (red) and poly(β-Gal-Thr)$_m$-*r*-poly(Cys-D)$_n$ (black) with constant *n*, varying *m* and poly(β-Gal-Thr)$_{22}$ (blue).

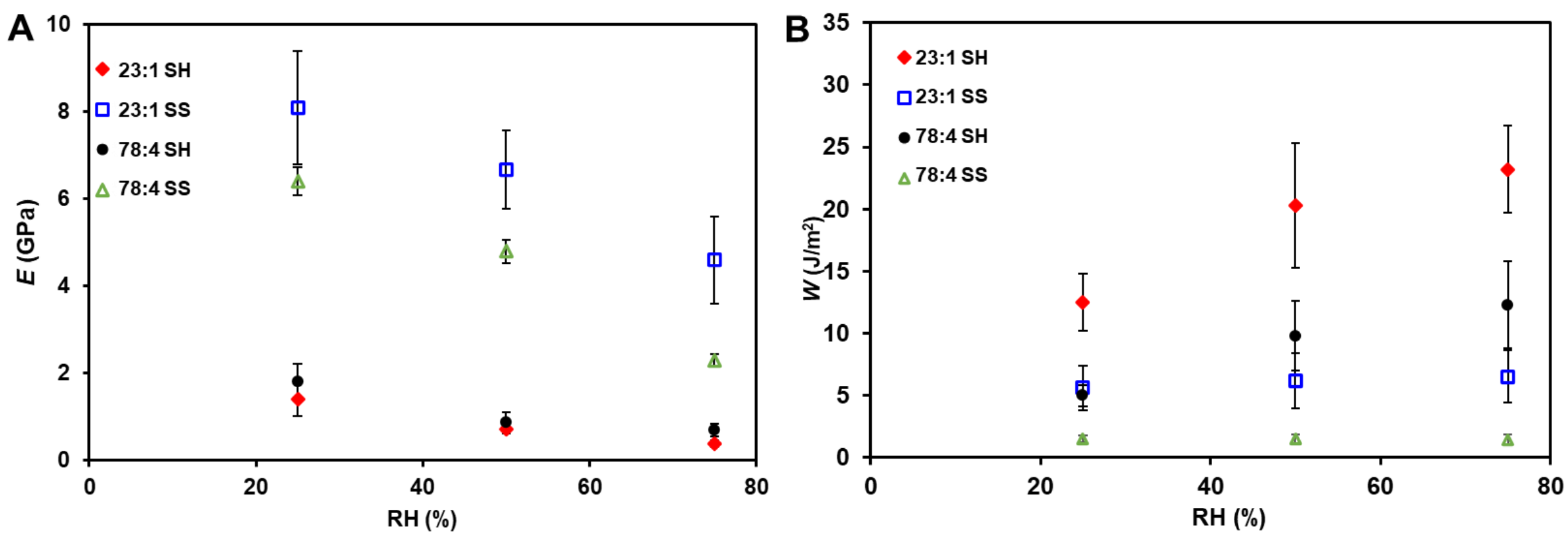

Figure S99. *E* and *W* of poly(*β*-Gal-Thr)$_m$-*r*-poly(Cys-H)$_n$ and poly(*β*-Gal-Thr)$_m$-*r*-poly(Cys-D)$_n$ at 25%, 50%, 75% RH. (A) *E* for poly(*β*-Gal-Thr)$_{23}$-*r*-poly(Cys-H)$_1$ (red), poly(*β*-Gal-Thr)$_{23}$-*r*-poly(Cys-D)$_1$ (blue), poly(*β*-Gal-Thr)$_{78}$-*r*-poly(Cys-H)$_4$ (black), and poly(*β*-Gal-Thr)$_{78}$-*r*-poly(Cys-D)$_4$ (green). (B) *W* for *E* for poly(*β*-Gal-Thr)$_{23}$-*r*-poly(Cys-H)$_1$ (red), poly(*β*-Gal-Thr)$_{23}$-*r*-poly(Cys-D)$_1$ (blue), poly(*β*-Gal-Thr)$_{78}$-*r*-poly(Cys-H)$_4$ (black), and poly(*β*-Gal-Thr)$_{78}$-*r*-poly(Cys-D)$_4$ (green).

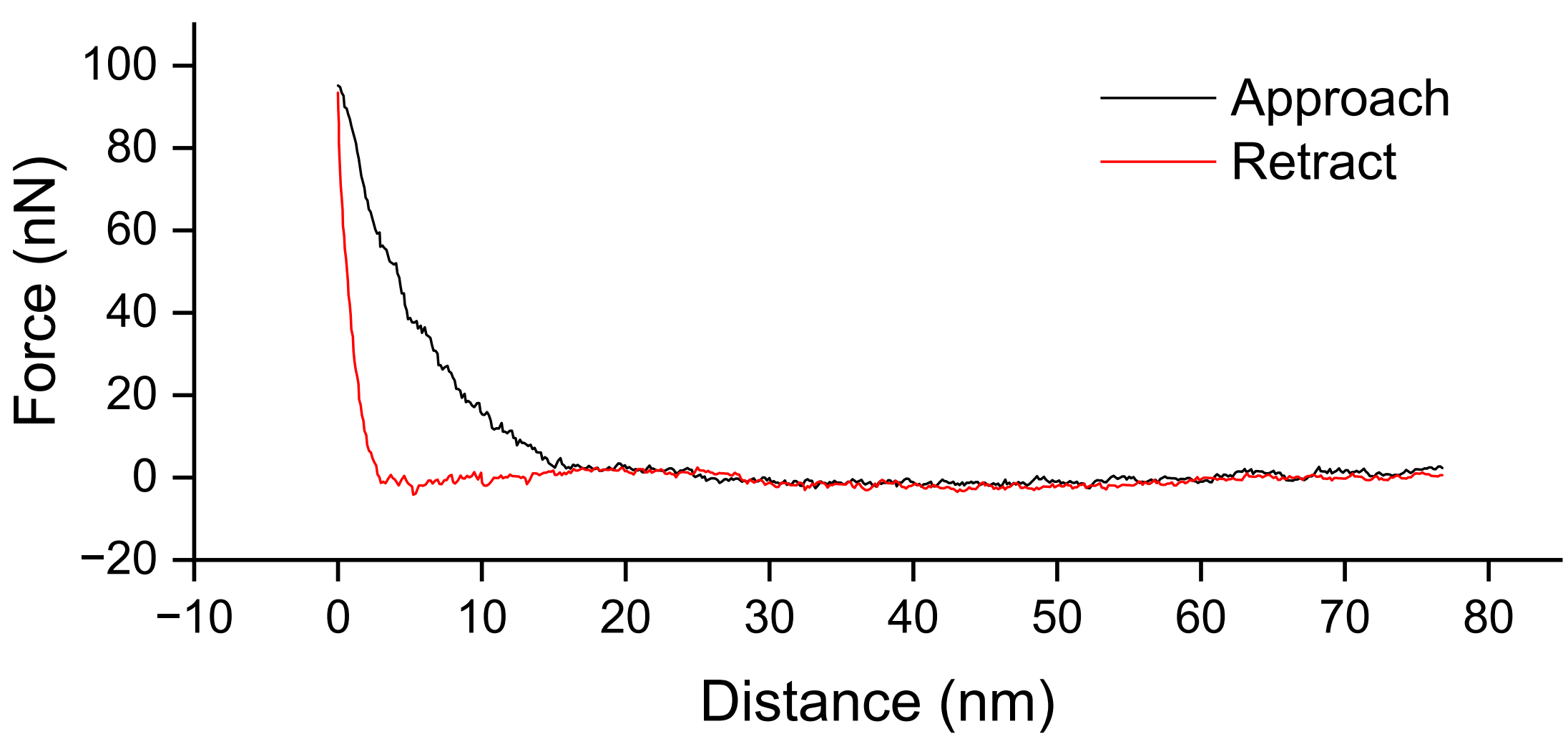

Figure S100. The force-distance curve of poly(*β*-Gal-Thr)$_{22}$ at 50% RH

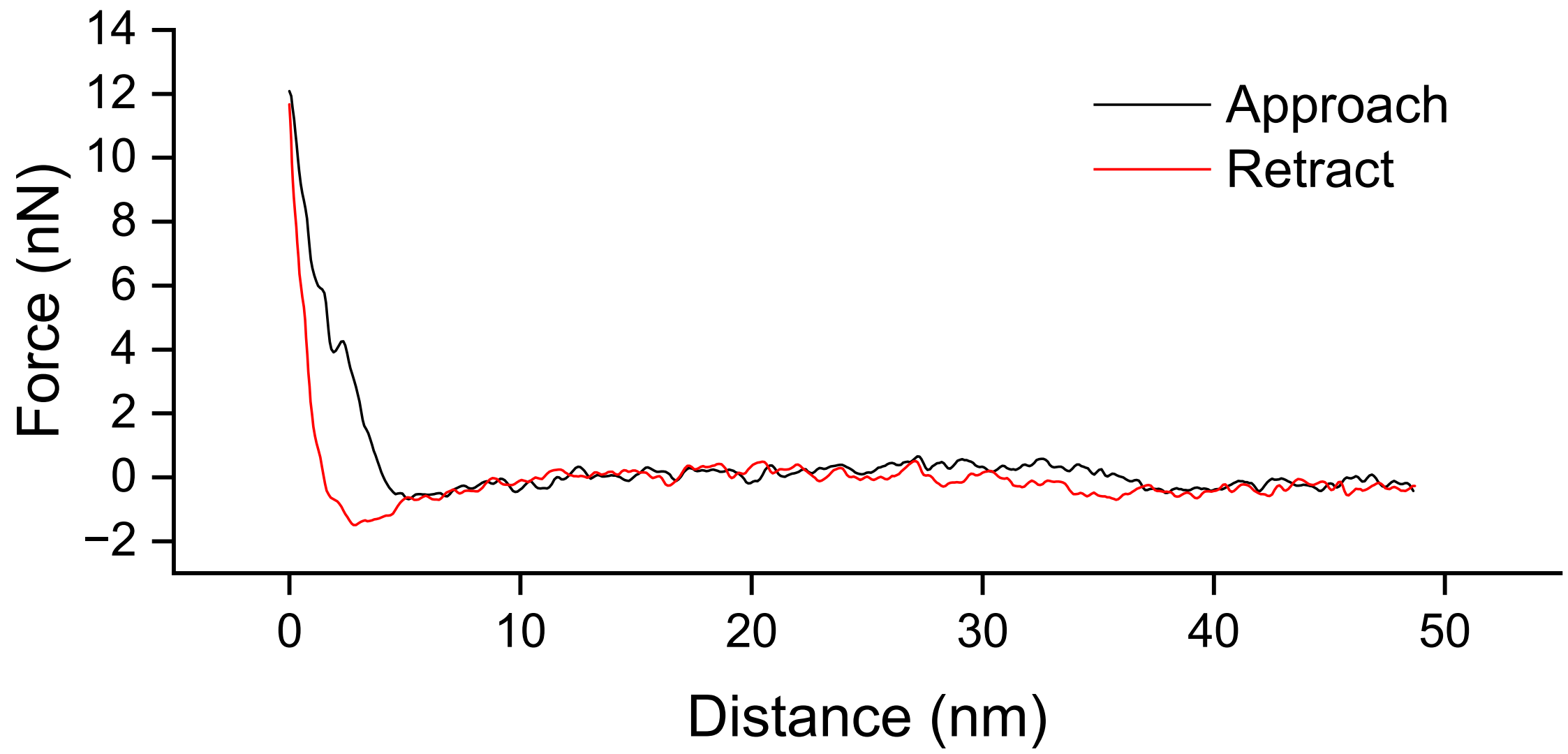

Figure S101. The force-distance curve of poly(β-Gal-Thr)$_{23}$-*r*-poly(Cys-H)$_1$ at 25% RH.

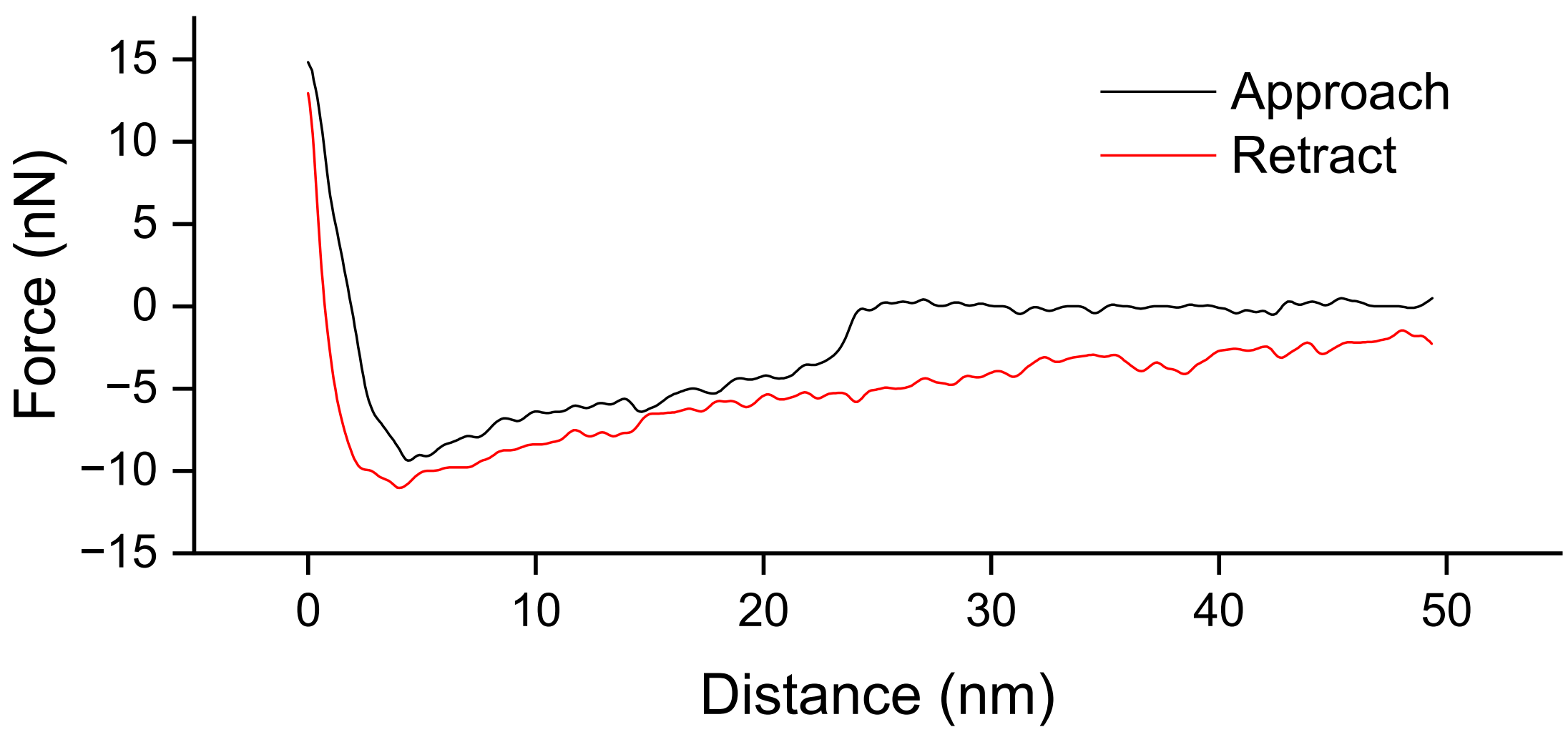

Figure S102. The force-distance curve of poly(β-Gal-Thr)$_{23}$-*r*-poly(Cys-H)$_1$ at 50% RH.

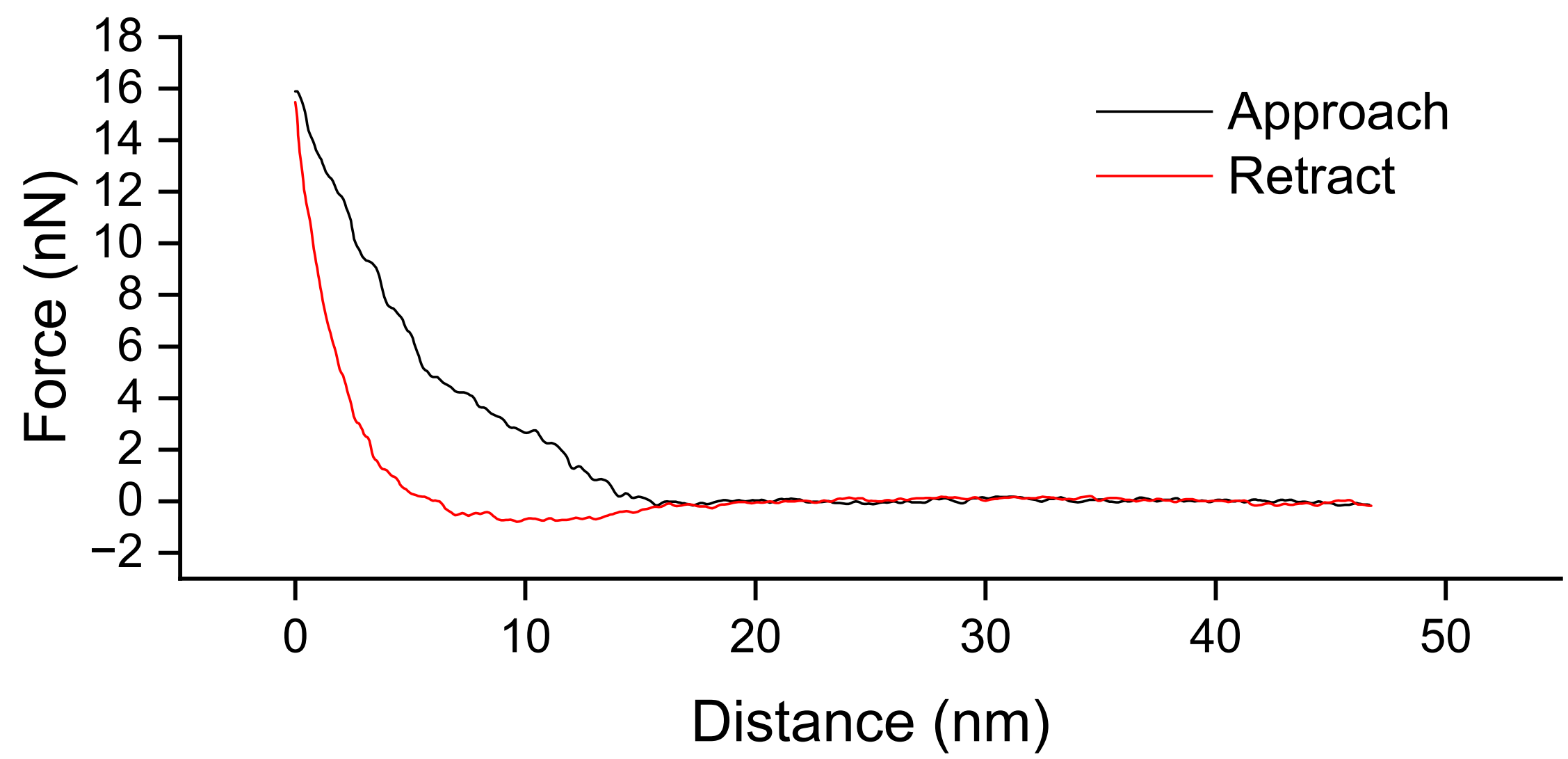

Figure S103. The force-distance curve of poly(*β*-Gal-Thr)$_{23}$-*r*-poly(Cys-H)$_1$ at 75% RH.

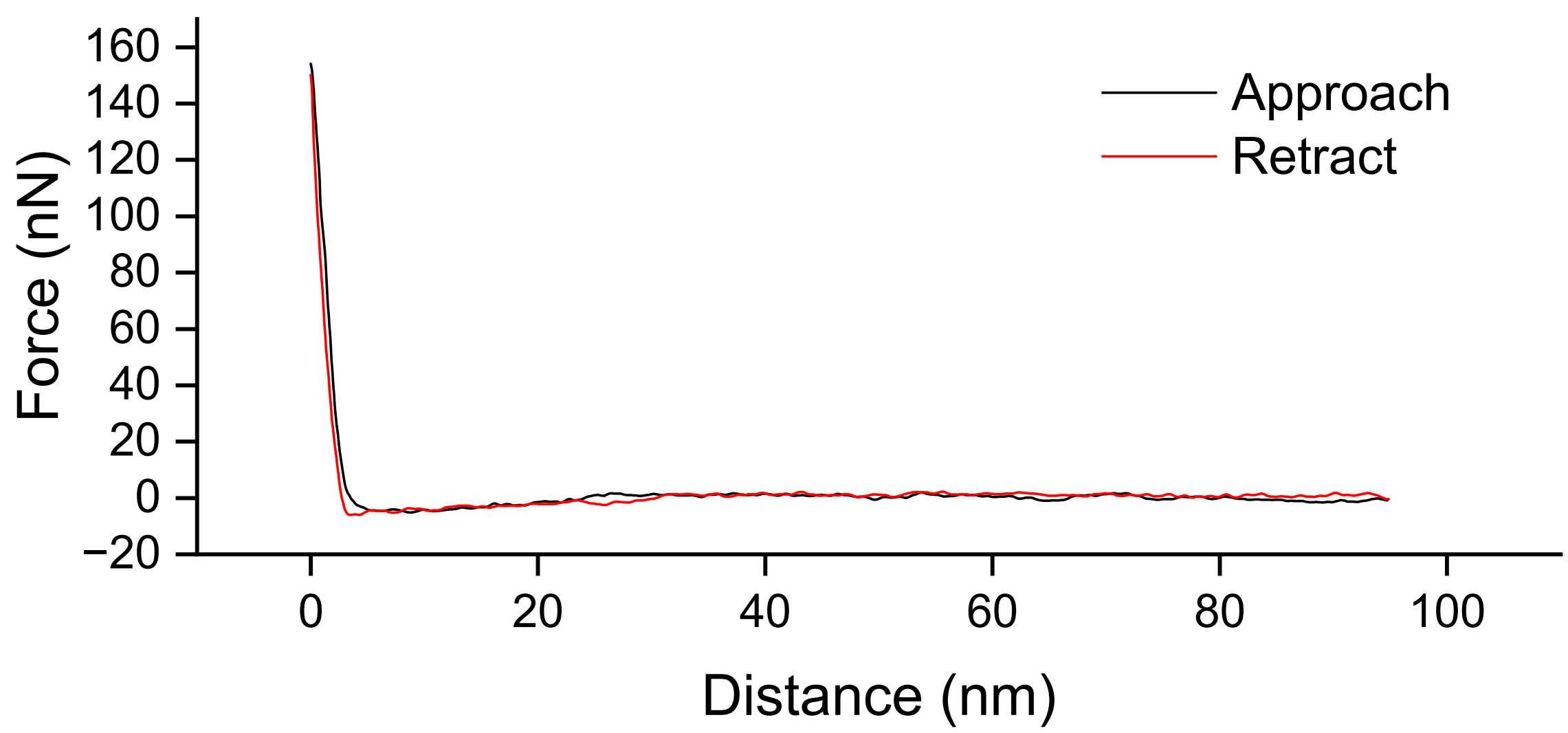

Figure S104. The force-distance curve of poly(*β*-Gal-Thr)$_{23}$-*r*-poly(Cys-D)$_1$ at 25% RH.

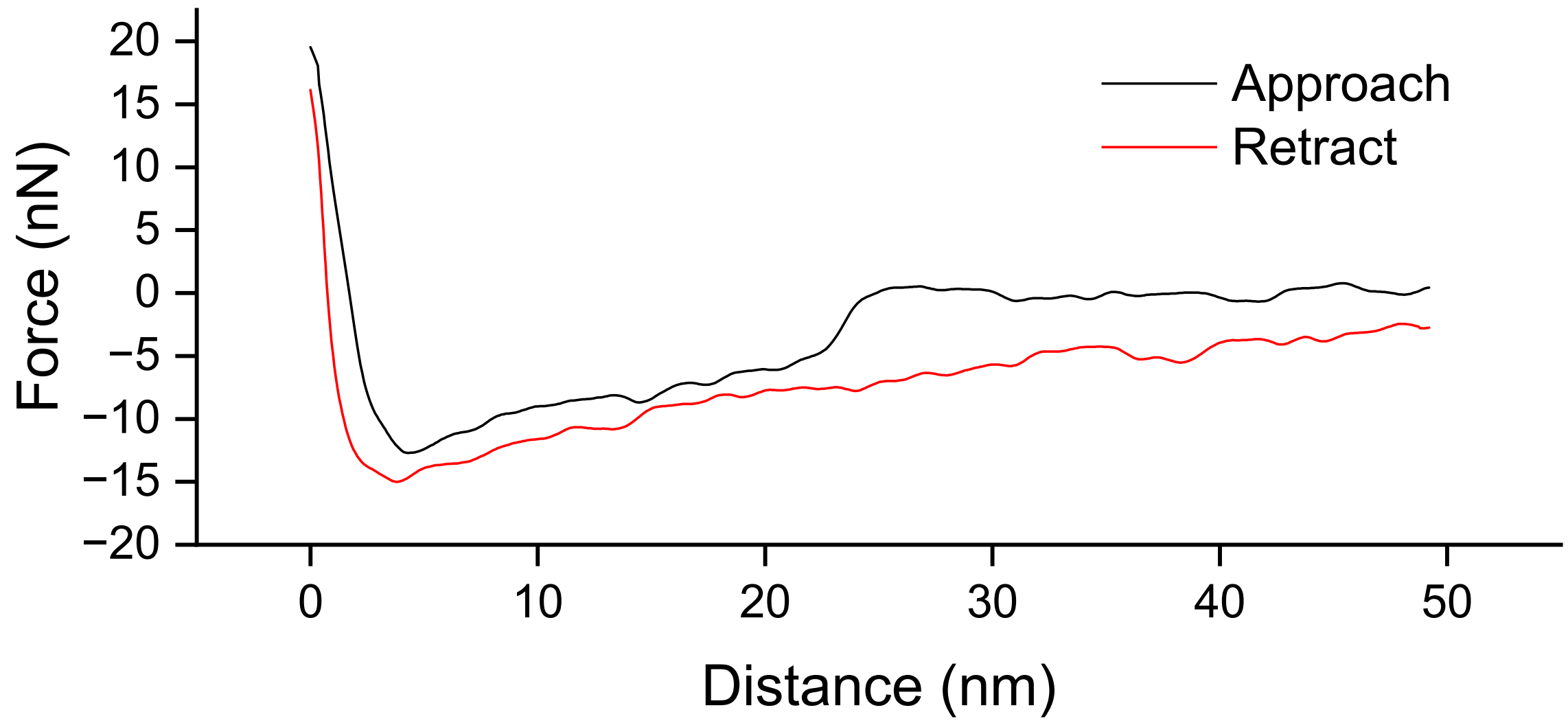

Figure S105. The force-distance curve of poly(β-Gal-Thr)$_{23}$-*r*-poly(Cys-D)$_1$ at 50% RH.

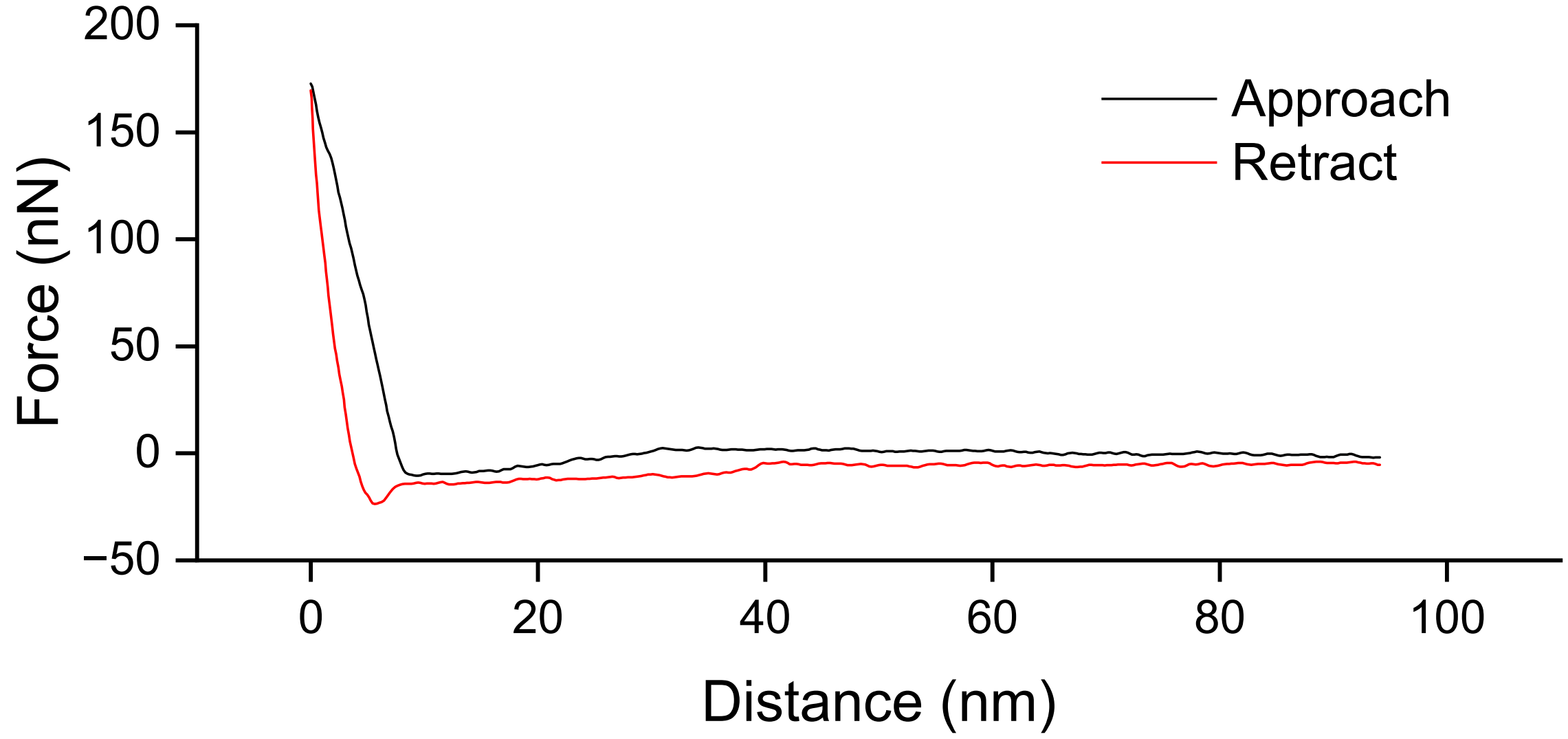

Figure S106. The force-distance curve of poly(β-Gal-Thr)$_{23}$-*r*-poly(Cys-D)$_1$ at 75% RH.

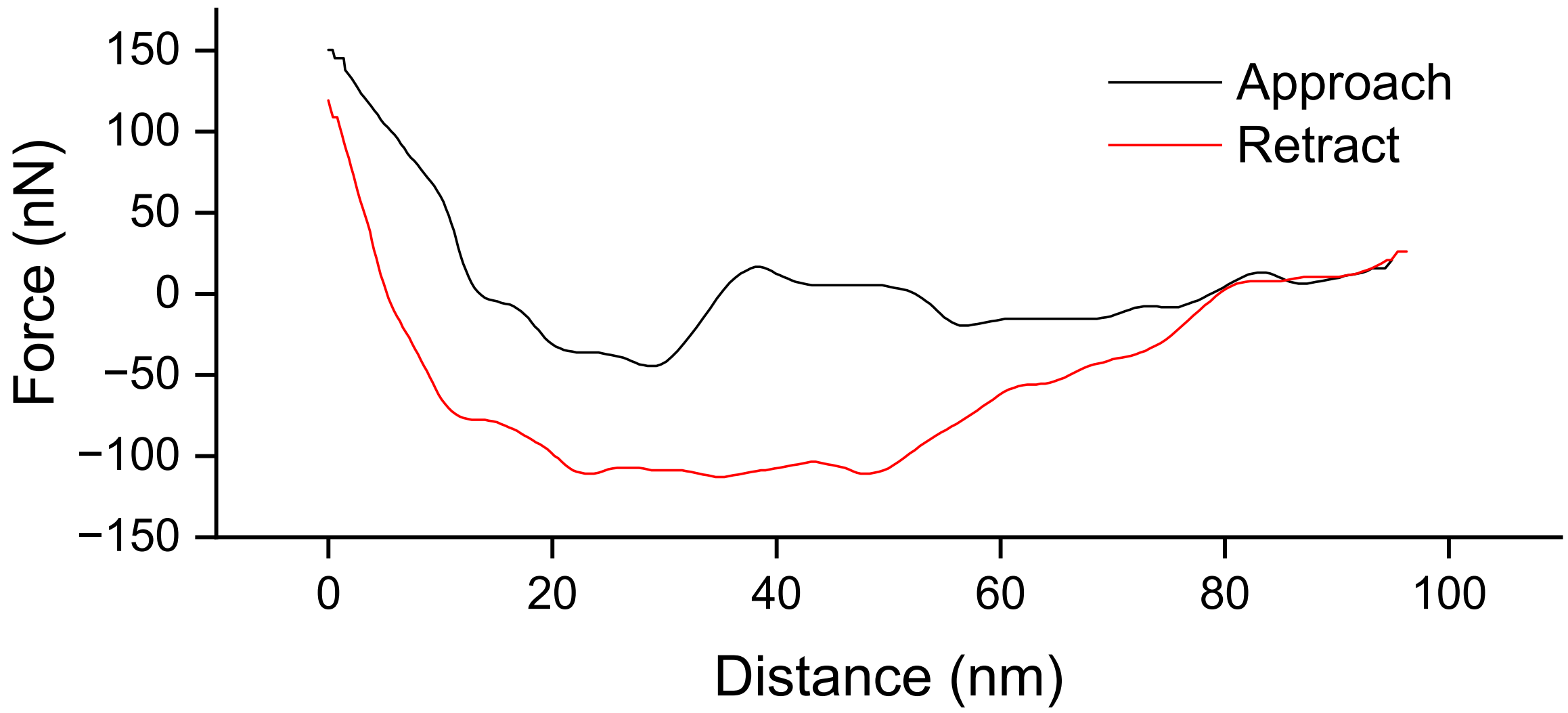

Figure S107. The force-distance curve of poly(β-Gal-Thr)$_{23}$-r-poly(Cys-H)$_2$ at 50% RH.

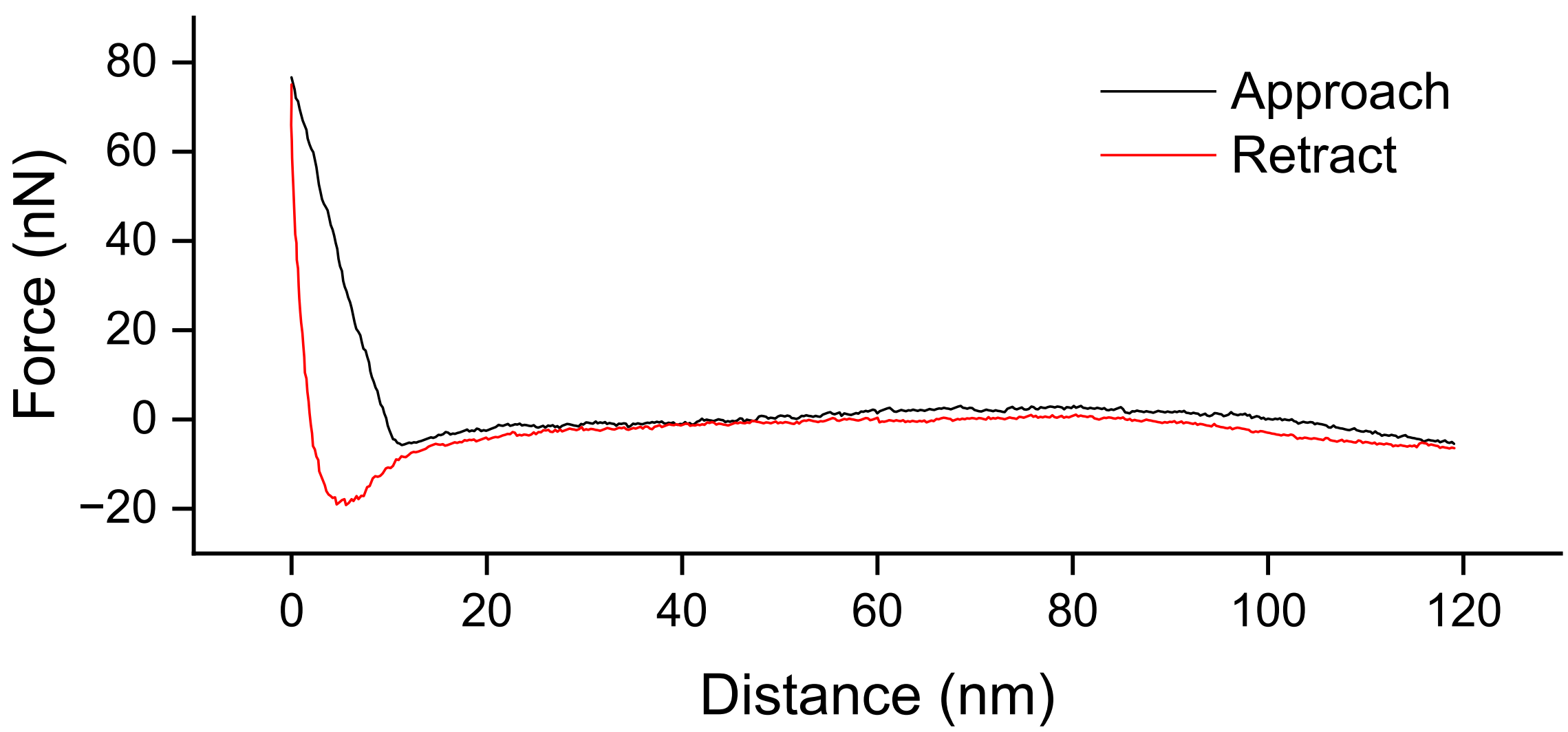

Figure S108. The force-distance curve of poly(β-Gal-Thr)$_{23}$-r-poly(Cys-D)$_2$ at 50% RH.

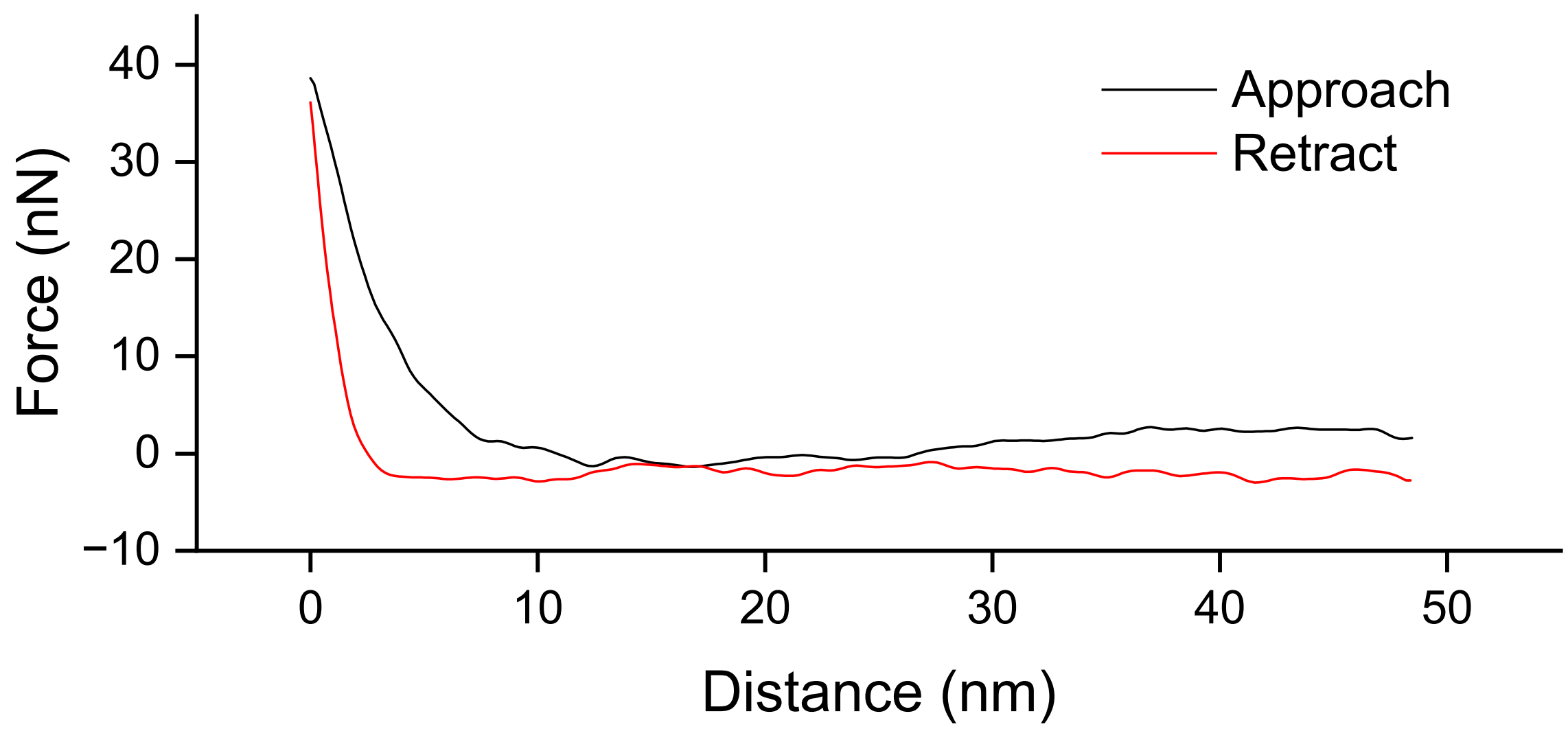

Figure S109. The force-distance curve of poly(β-Gal-Thr)$_{23}$-*r*-poly(Cys-H)$_3$ at 50% RH.

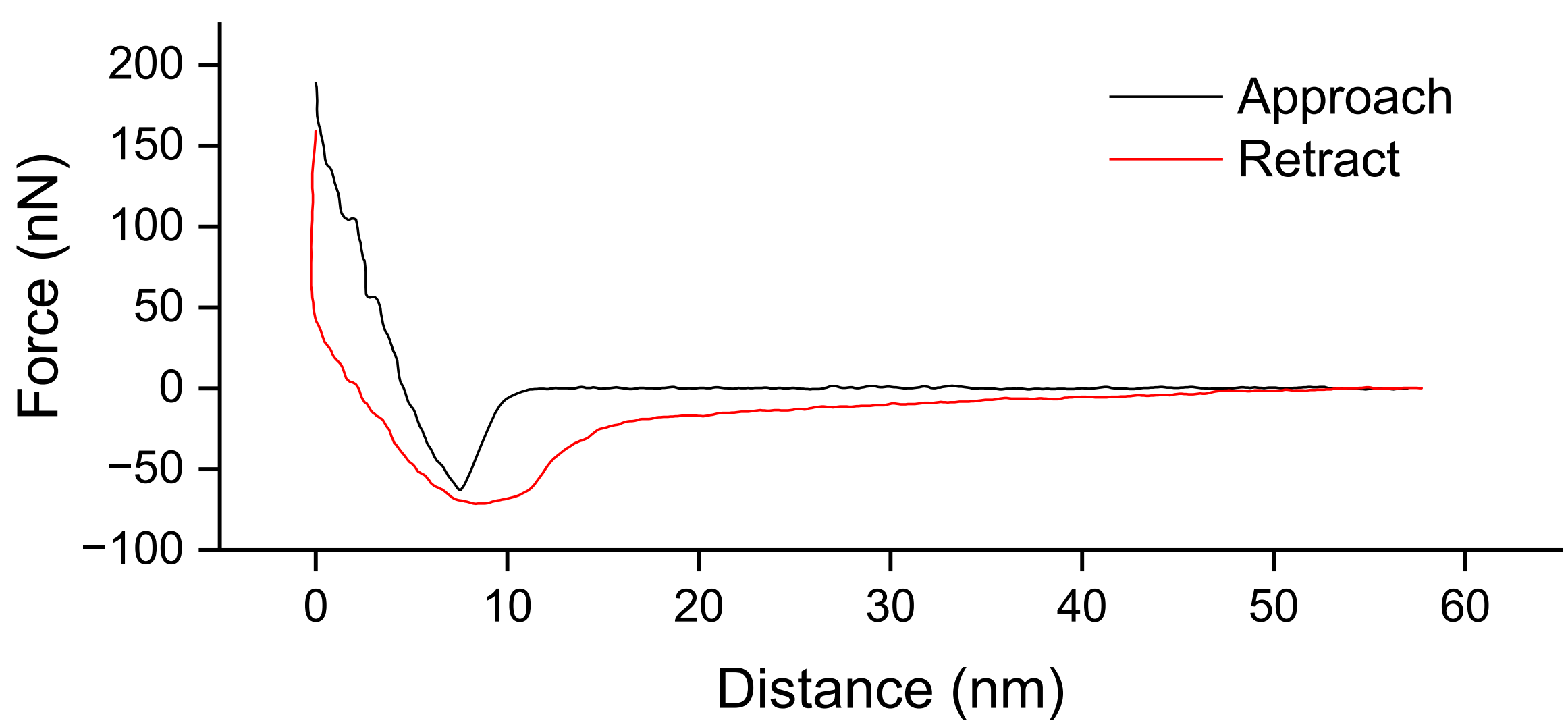

Figure S110. The force-distance curve of poly(β-Gal-Thr)$_{23}$-*r*-poly(Cys-D)$_3$ at 50% RH.

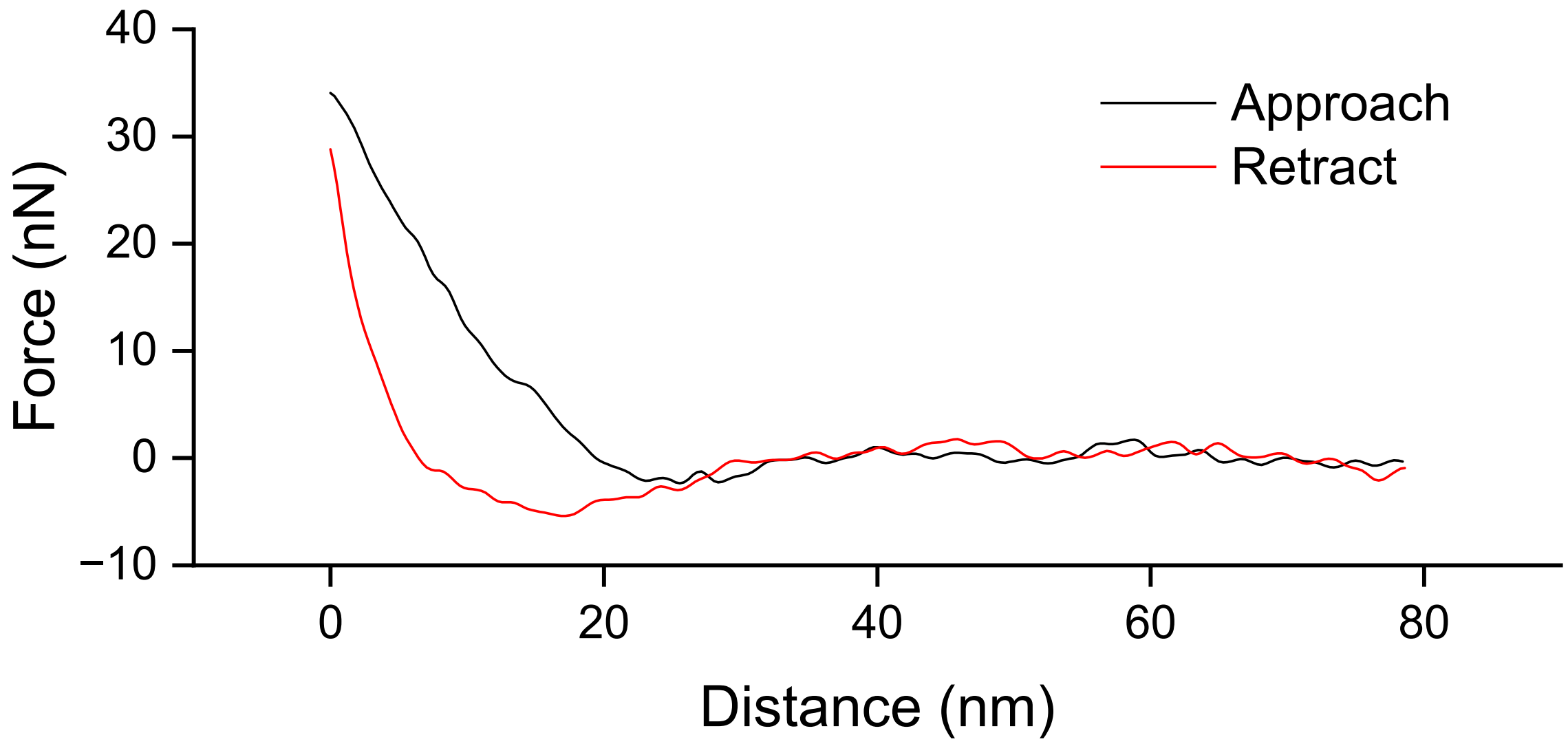

Figure S111. The force-distance curve of poly(β-Gal-Thr)$_{23}$-*r*-poly(Cys-H)$_6$ at 50% RH.

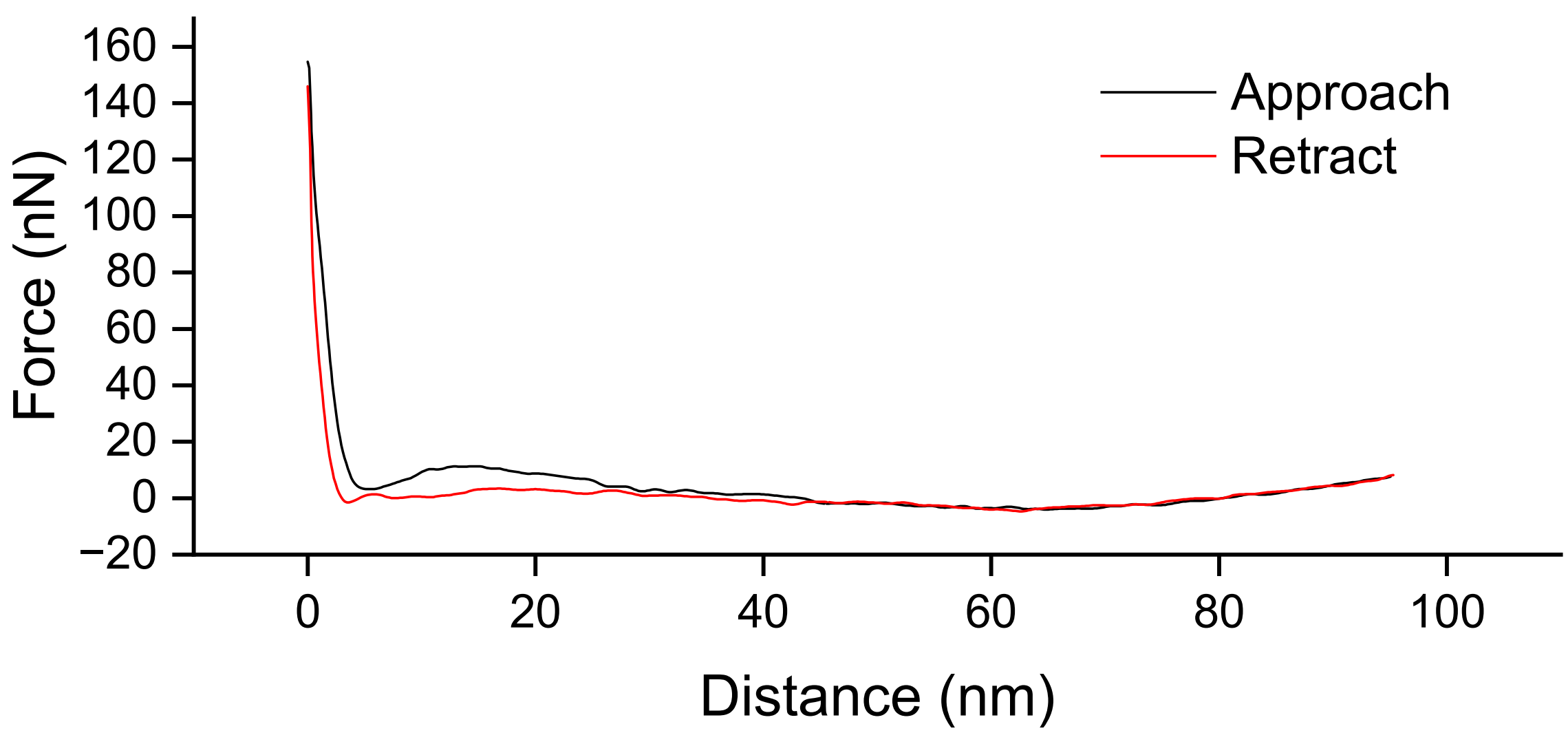

Figure S112. The force-distance curve of poly(β-Gal-Thr)$_{23}$-*r*-poly(Cys-D)$_6$ at 50% RH.

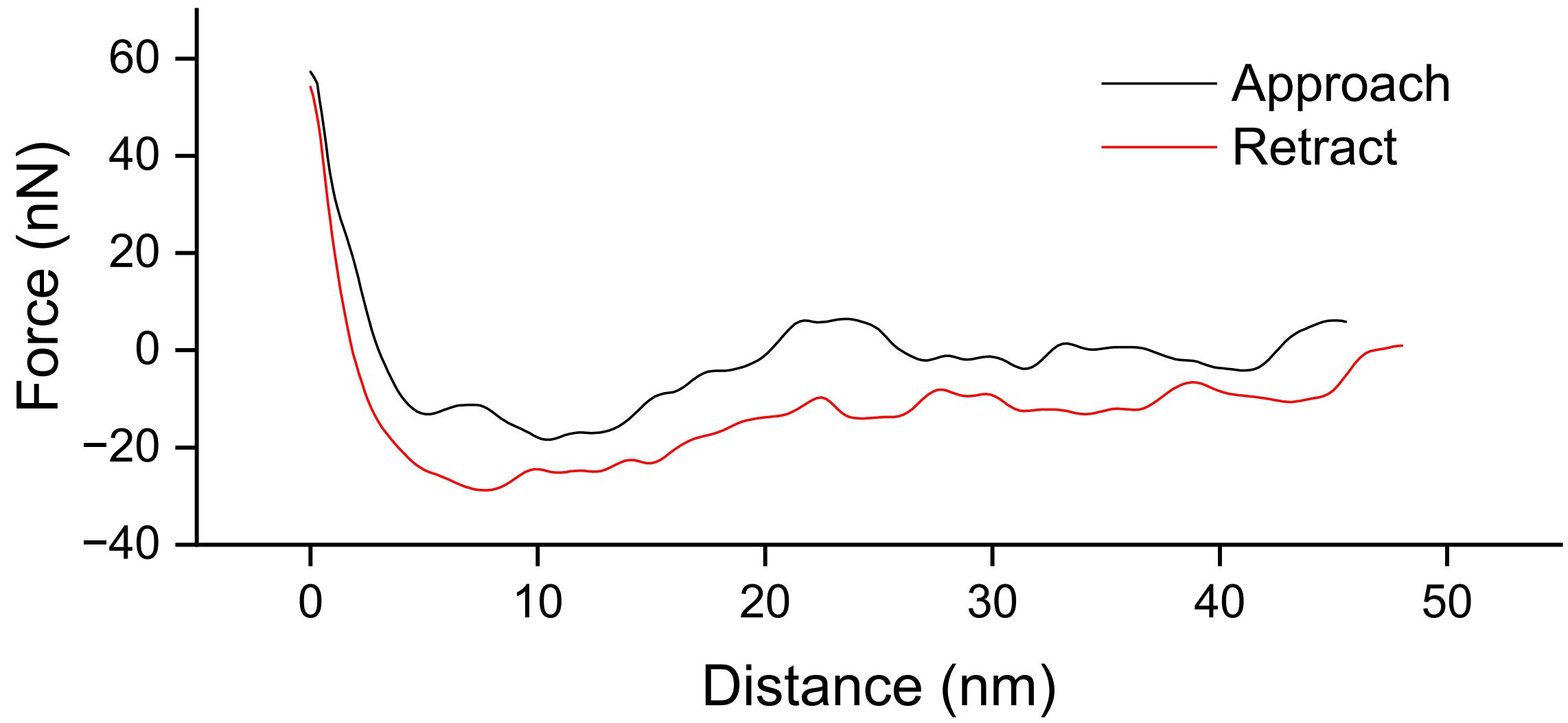

Figure S113. The force-distance curve of poly(β-Gal-Thr)$_{33}$-*r*-poly(Cys-H)$_3$ at 50% RH.

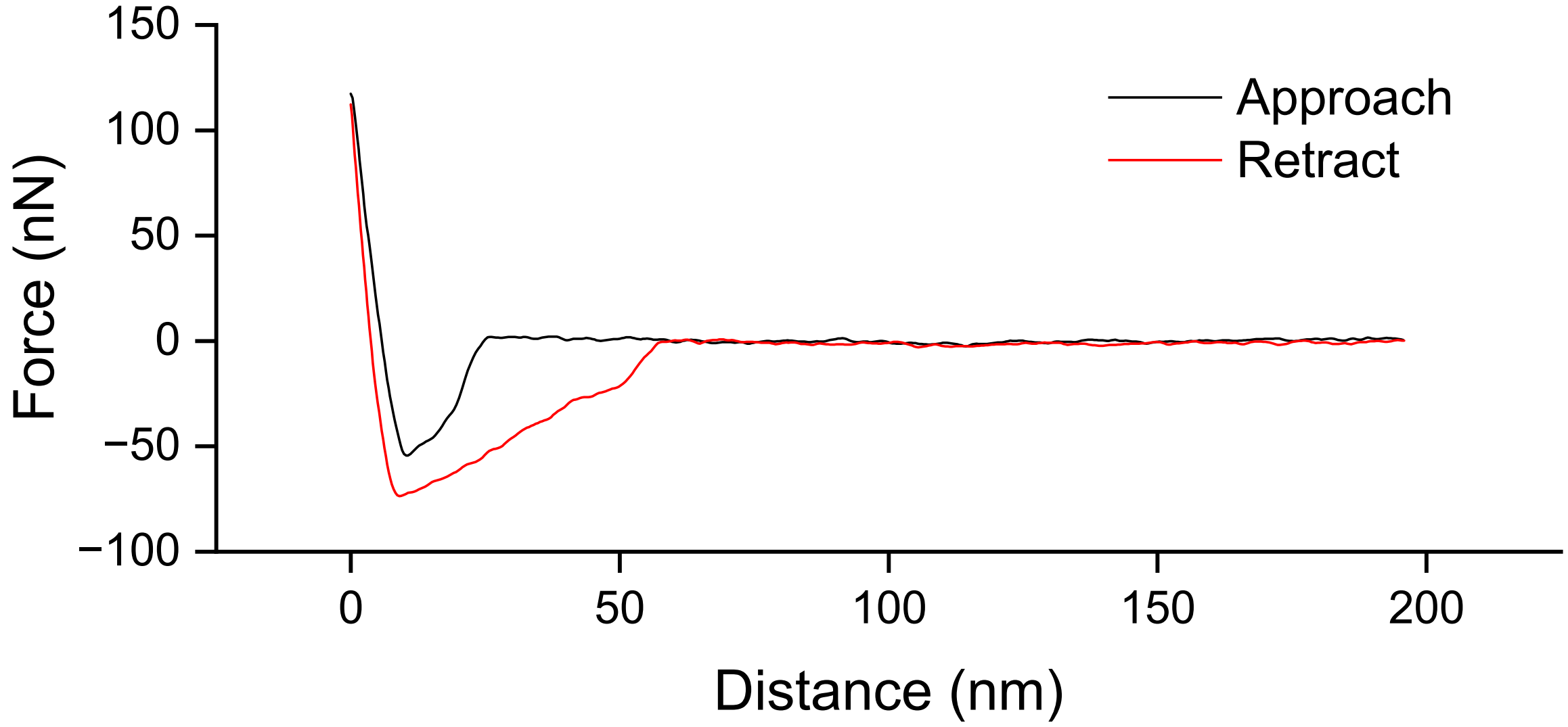

Figure S114. The force-distance curve of poly(β-Gal-Thr)$_{33}$-*r*-poly(Cys-D)$_3$ at 50% RH.

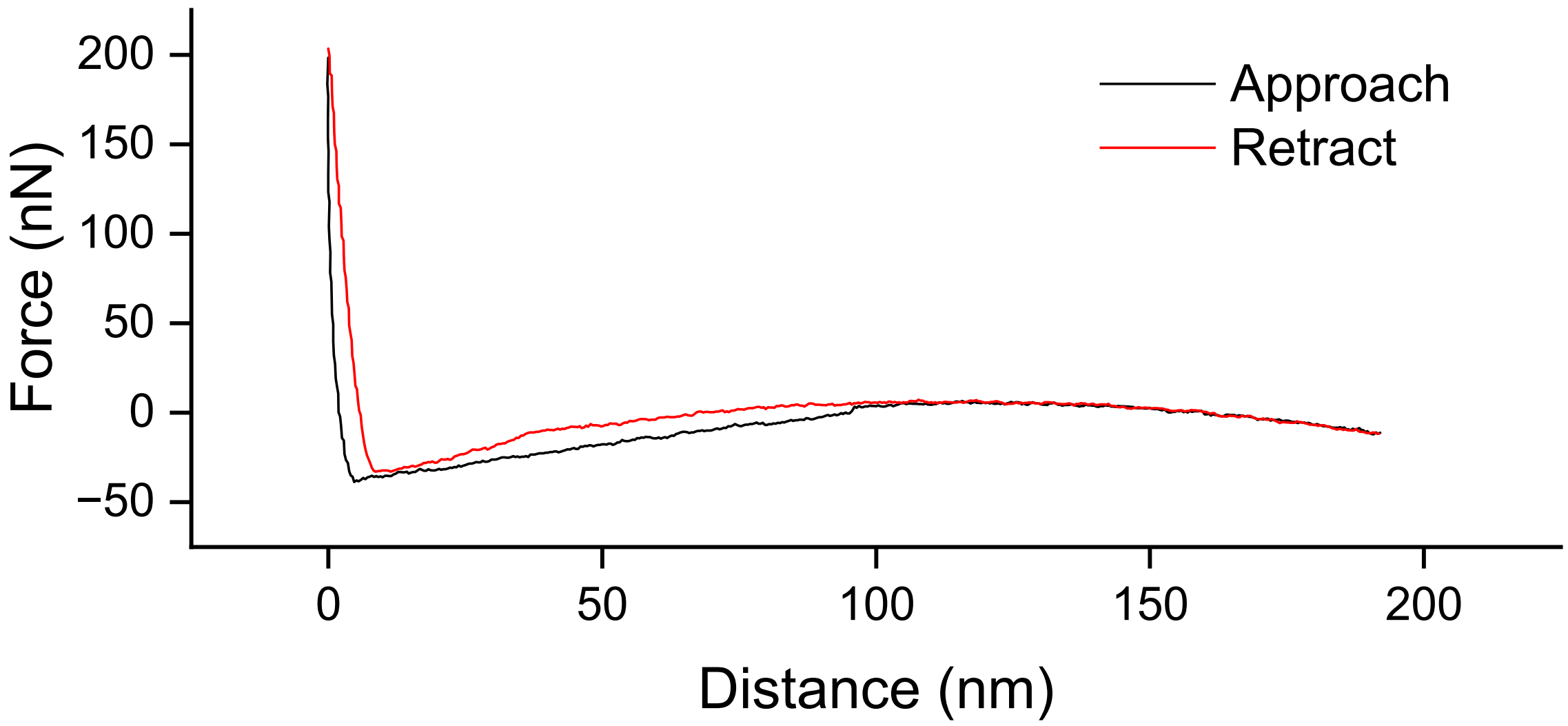

Figure S115. The force-distance curve of poly(β-Gal-Thr)$_{62}$-*r*-poly(Cys-H)$_3$ at 50% RH.

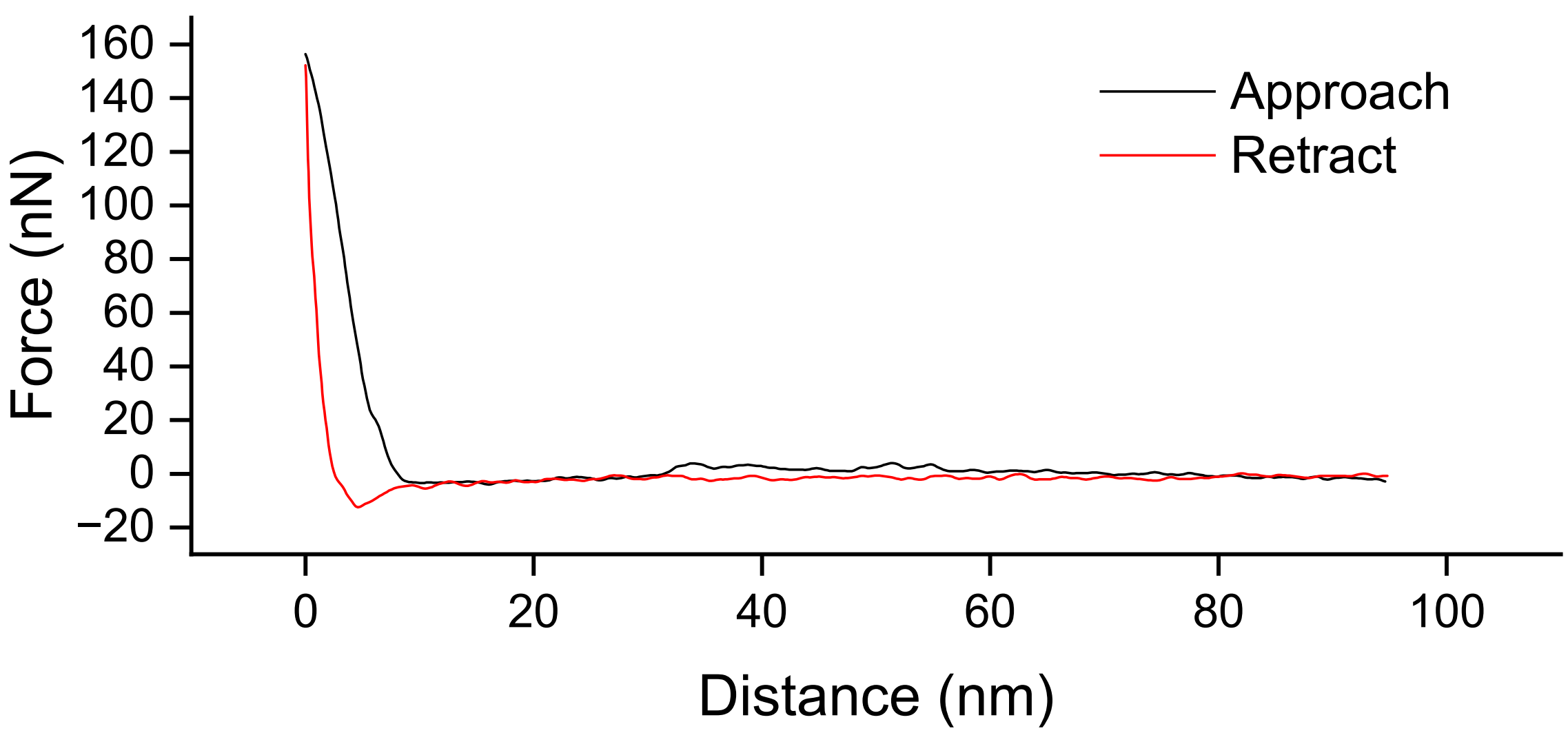

Figure S116. The force-distance curve of poly(β-Gal-Thr)$_{62}$-*r*-poly(Cys-D)$_3$ at 50% RH.

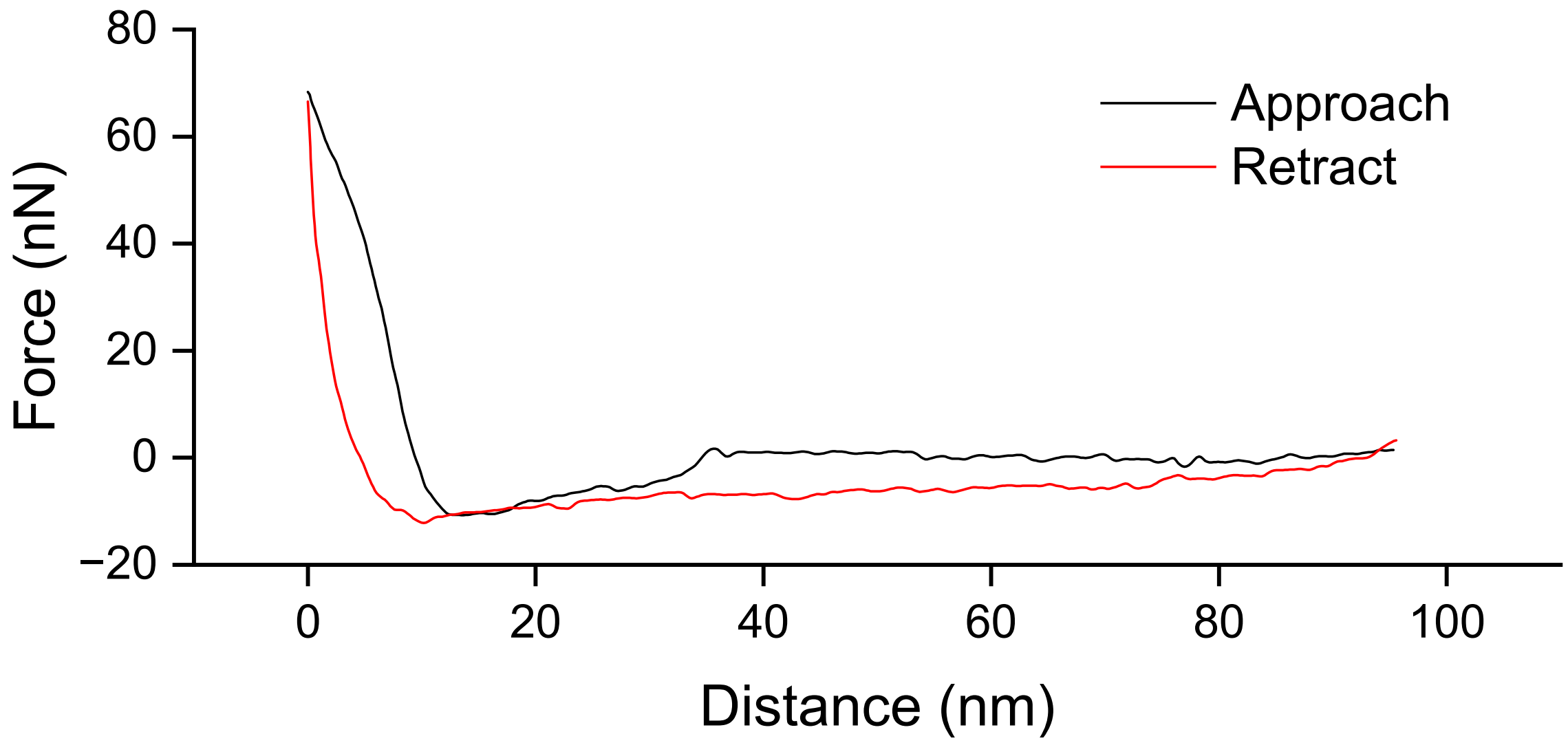

Figure S117. The force-distance curve of poly(β-Gal-Thr)$_{78}$-*r*-poly(Cys-H)$_4$ at 25% RH.

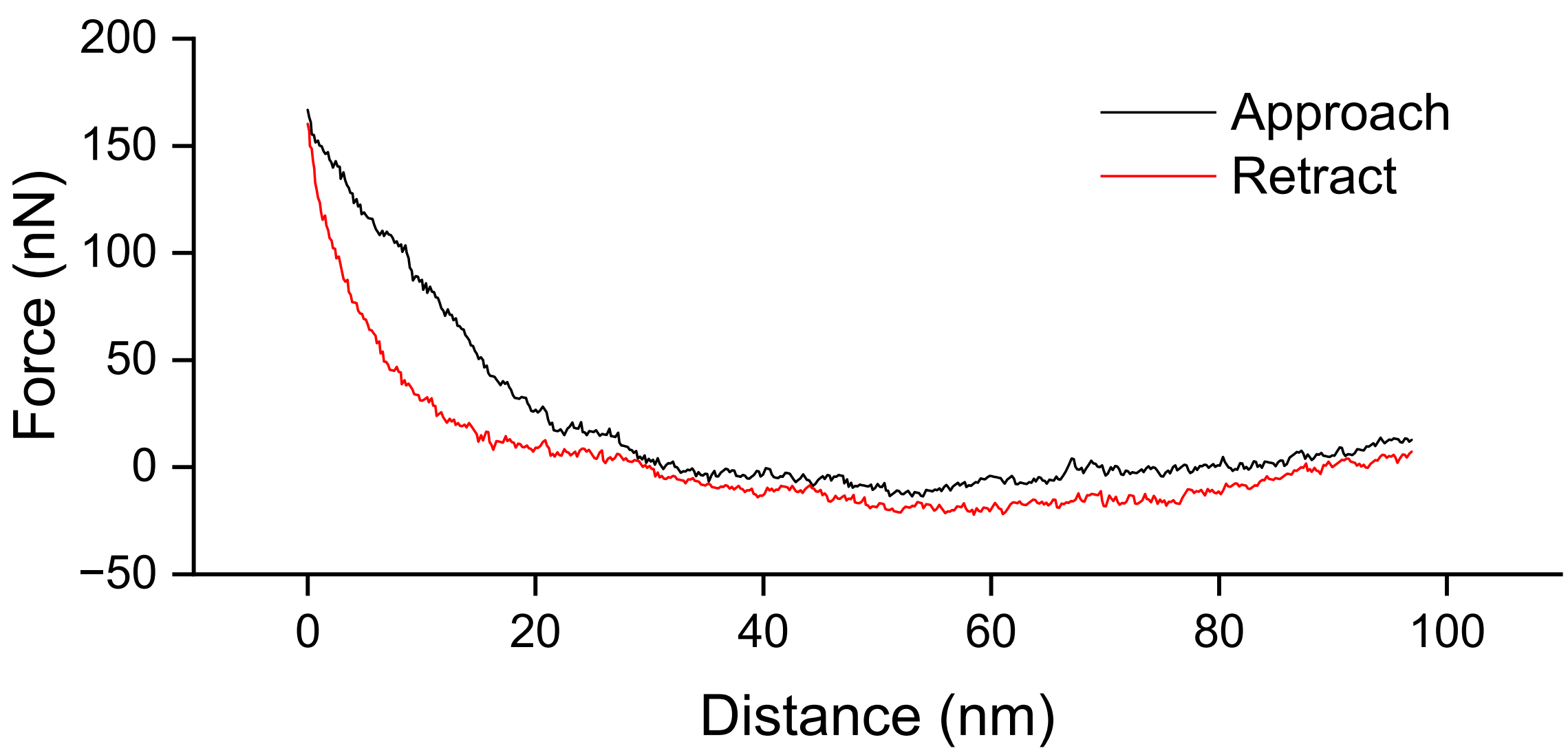

Figure S118. The force-distance curve of poly(β-Gal-Thr-H)$_{78}$-*r*-poly(Cys-H)$_4$ at 50% RH

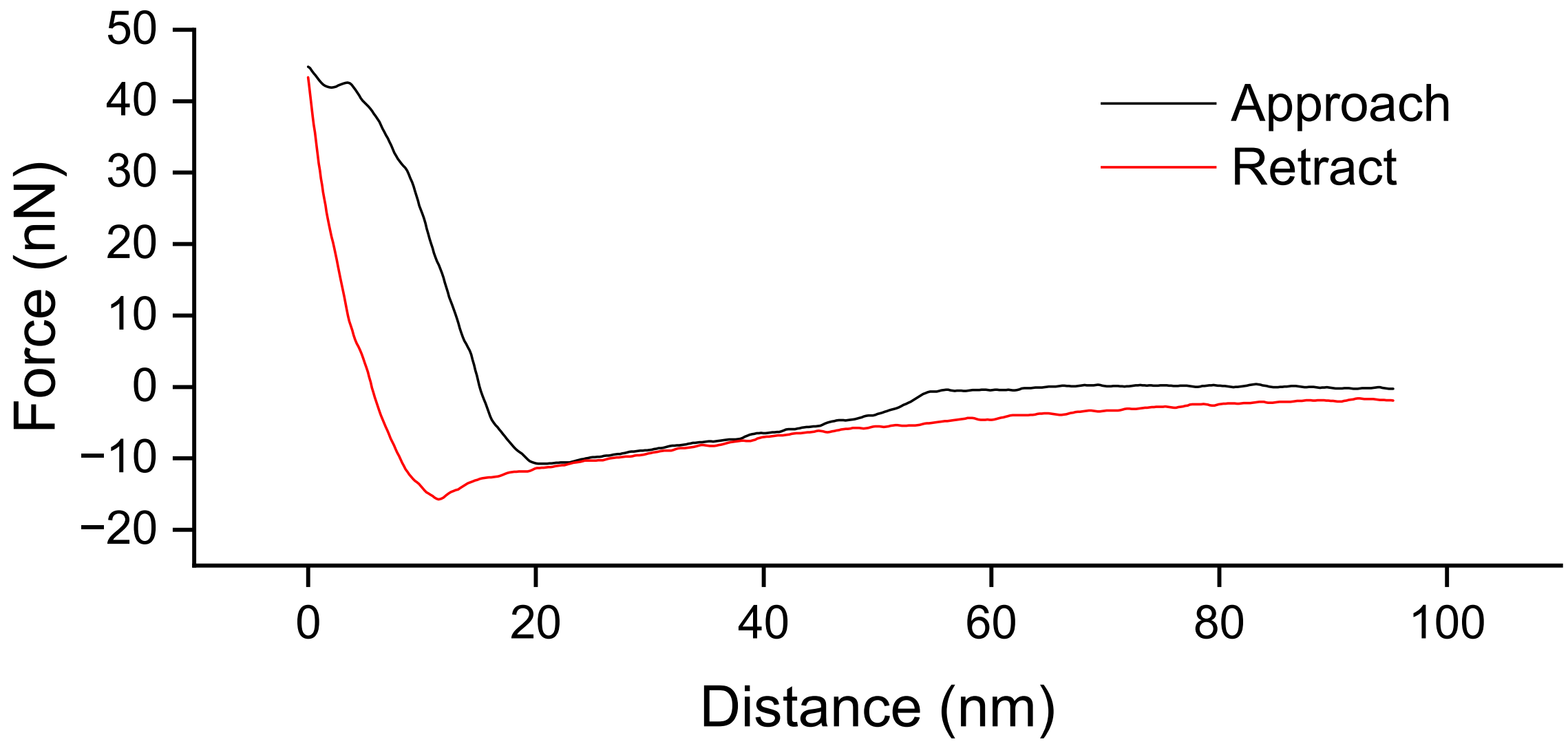

Figure S119. The force-distance curve of poly(β-Gal-Thr)$_{78}$-*r*-poly(Cys-H)$_4$ at 75% RH.

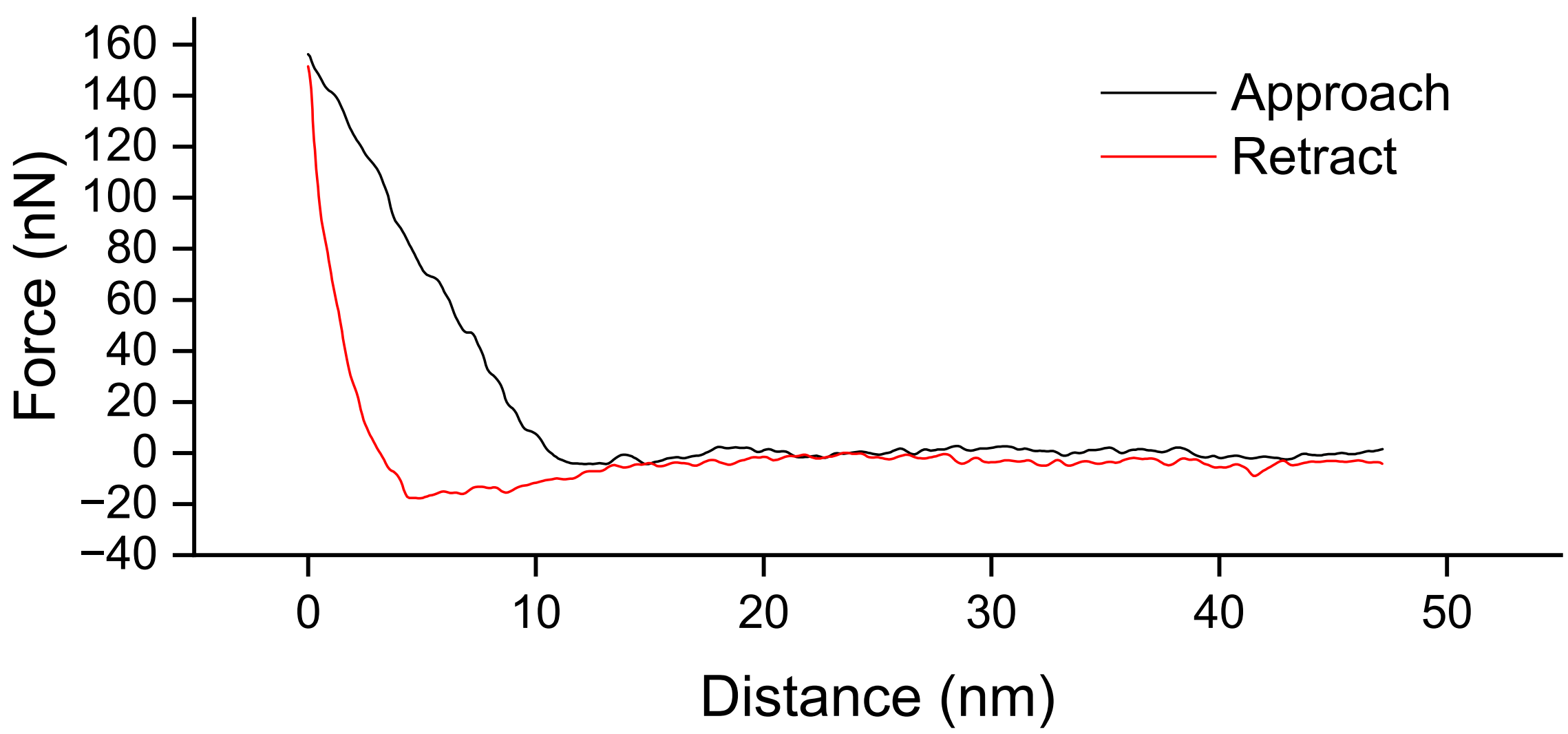

Figure S120. The force-distance curve of poly(β-Gal-Thr)$_{78}$-*r*-poly(Cys-D)$_4$ at 25% RH.

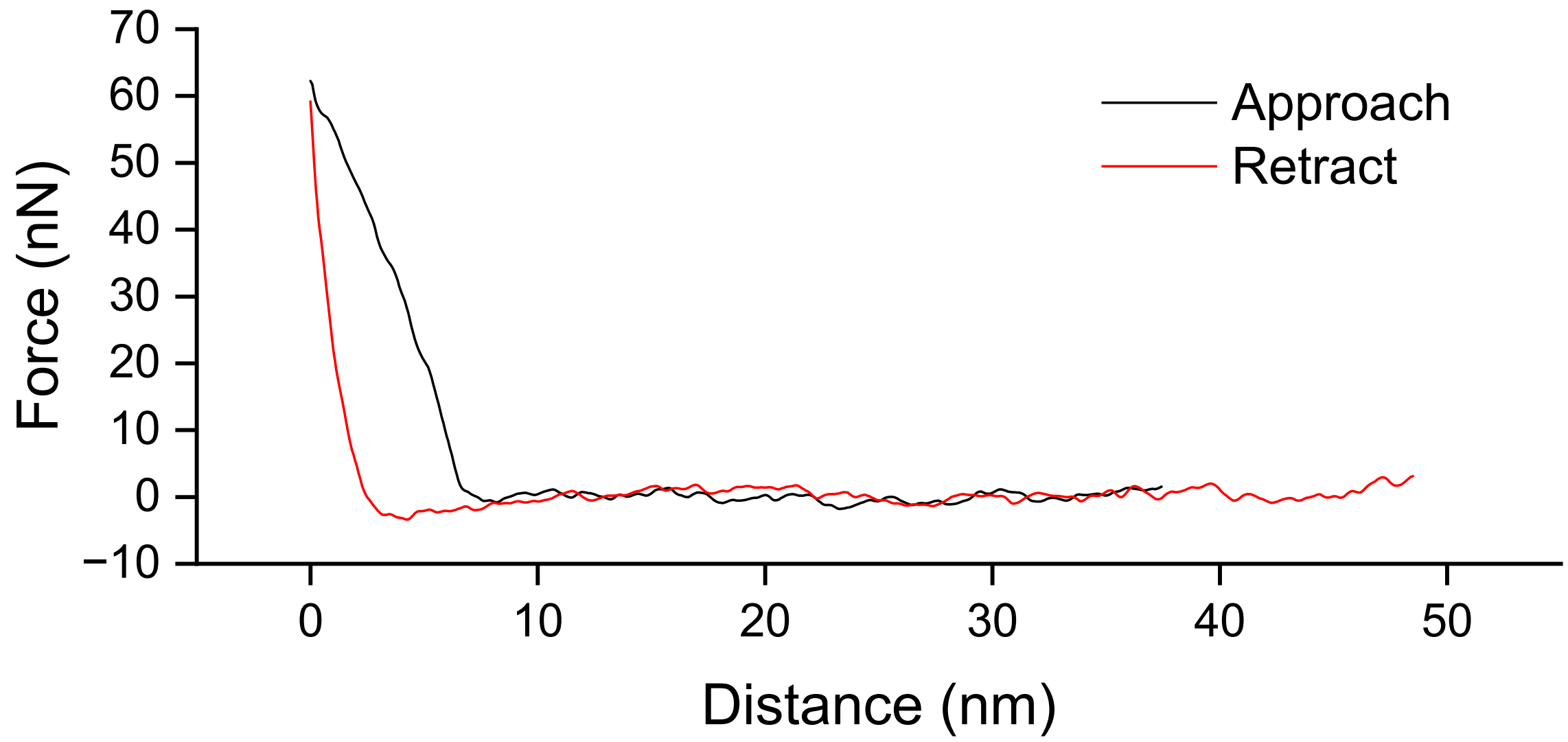

Figure S121. The force-distance curve of poly(β-Gal-Thr)$_{78}$-*r*-poly(Cys-D)$_4$ at 50% RH

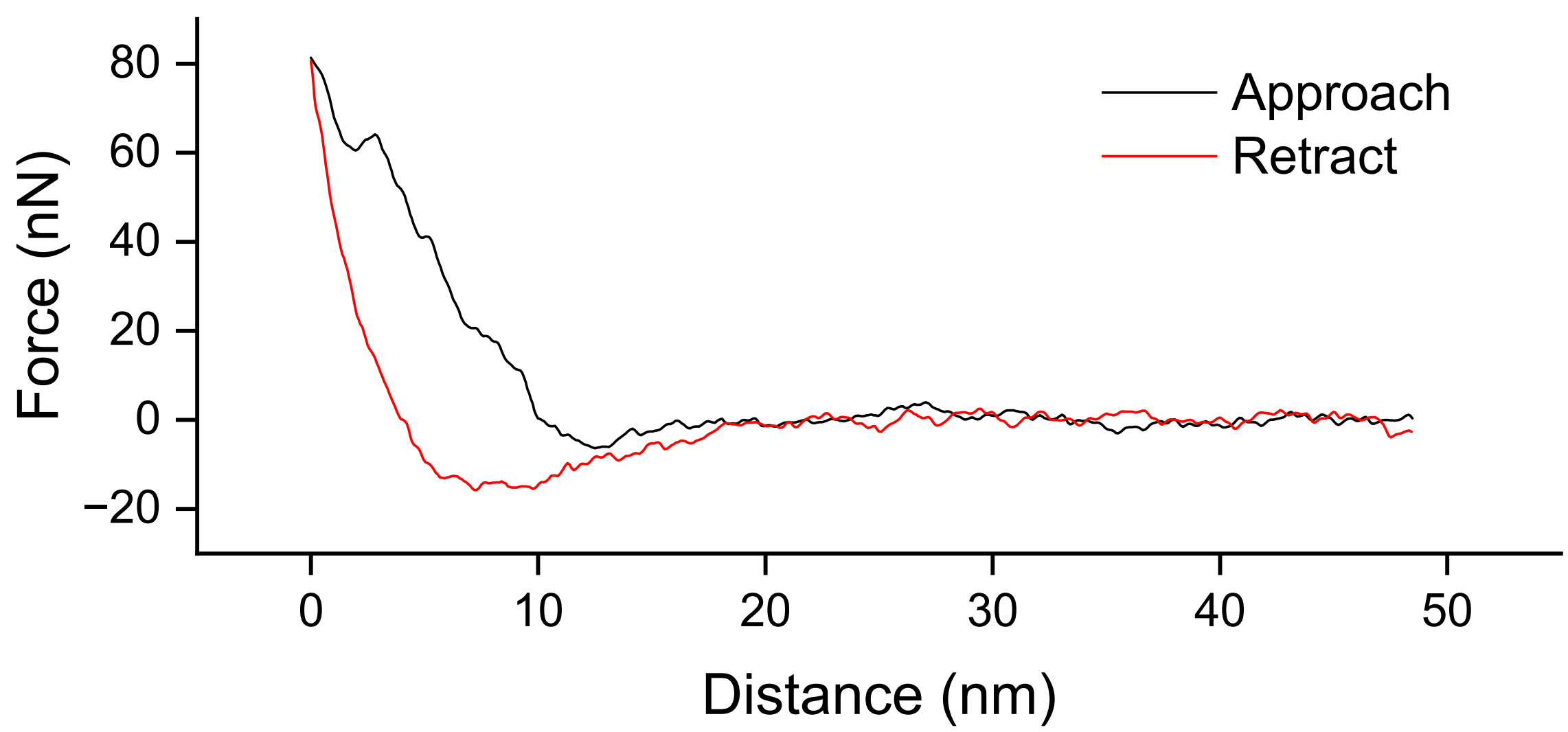

Figure S122. The force-distance curve of poly(β-Gal-Thr)$_{78}$-*r*-poly(Cys-D)$_4$ at 75% RH.

7. References

(1) Lema, M. A.; Nava-Medina, I. B.; Cerullo, A. R.; Abdelaziz, R.; Jimenez, S. M.; Geldner, J. B.; Abdelhamid, M.; Kwan, C.-S.; Kharlamb, L.; Neary, M. C.; Braunschweig, A. B. Scalable Preparation of Synthetic Mucins via Nucleophilic Ring-Opening Polymerization of Glycosylated *N*-Carboxyanhydrides. *Macromolecules* **2022**, *55* (11), 4710-4720.

(2) Habraken, G. J.; Koning, C. E.; Heuts, J. P.; Heise, A. Thiol chemistry on well-defined synthetic polypeptides. *Chem. Commun.* **2009**, (24), 3612-3614.

(3) Jager, S.; Meyer, P.; Feichtner, K. S.; Henkel, S.; Schwaab, G. W.; Gessner, V. H.; Havenith, M. Reaction of lithium hexamethyldisilazide (LiHMDS) with water at ultracold conditions. *Phys. Chem. Chem. Phys.* **2022**, *24* (39), 24089-24094.

(4) Nagao, M.; Matsubara, T.; Hoshino, Y.; Sato, T.; Miura, Y. Topological Design of Star Glycopolymers for Controlling the Interaction with the Influenza Virus. *Bioconjug. Chem.* **2019**, *30* (4), 1192-1198

(5) Held,; Kilz, P. Qualification of GPC/GFC/SEC Data and Results. Quantification in LC and GC: A Practical Guide to Good Chromotographic Data. *Wiley-VCH* **2009**, 271-305.

(6) Burgess, S.; Pinard, P. AZtec Wave – a New Way to Achieve Combined EDS and WDS Capability on SEM. *Microsc. Microanal.* **2020**, *26* (S2), 114-115.

(7) Wang, H.; Liu, Z. L.; Lao, J.; Zhang, S.; Abzalimov, R.; Wang, T.; Chen, X. High Energy and Power Density Peptidoglycan Muscles through Super-Viscous Nanoconfined Water. *Adv. Sci. (Weinh)* **2022**, *9* (15), e2104697.

(8) Podbevsek, D.; Jung, Y.; Khan, M. K.; Yu, H.; Tu, R. S.; Chen, X. The role of water mobility on water-responsive actuation of silk. *Nat. Commun.* **2024**, *15* (1), 8287.

(9) Liu, Z. L.; Chen, X. Water-Content-Dependent Morphologies and Mechanical Properties of Bacillus subtilis Spores' Cortex Peptidoglycan. *ACS Biomater. Sci. Eng.* **2022**, *8* (12), 5094-5100.

(10) Gao, Y.; Kim, S.; Zhou, S.; Chiu, H. C.; Nelias, D.; Berger, C.; Heer, W.; Polloni, L.; Sordan, R.; Bongiorno, A.; Riedo, E. Elastic coupling between layers in two-dimensional materials. *Nat. Mater.* **2015**, *14* (7), 714-720.

(11) Harrellson, S. G.; DeLay, M. S.; Chen, X.; Cavusoglu, A. H.; Dworkin, J.; Stone, H. A.; Sahin, O. Hydration solids. *Nature* **2023**, *619* (7970), 500-505.

(12) Gao, Y.; Cao, T.; Cellini, F.; Berger, C.; Heer, W. A.; Tosatti, E.; Riedo, E.; Bongiorno, A. Ultrahard carbon film from epitaxial two-layer graphene. *Nat. Nanotechnol.* **2018**, *13* (2), 133-138.

(13) Maghsoudy-Louyeh, S.; Kropf, M.; Tittmann, B. R. Review of Progress in Atomic Force Microscopy. *Open Neuroimaging J.* **2018**, 7, *12*-86.

(14) Butt, H. J.; Cappella, B.; Kappl, M. Force measurements with the atomic force microscope: Technique, interpretation and applications. *Surf. Sci. Rep.* **2005**, *59* (1-6), 1-152.
(15) Doorn, J. M. v. On the verge: Mechanics in the limit of vanishing strength and stiffness. Ph.D. Thesis, *Wageningen University and Research* **2019**, 1-262.
